# Toward a Comprehensive Anatomical Matrix for Crown Birds: Phylogenetic Insights from the Pectoral Girdle and Forelimb Skeleton

**DOI:** 10.1093/iob/obaf029

**Published:** 2025-07-24

**Authors:** A Chen, E M Steell, R B J Benson, D J Field

**Affiliations:** Department of Earth Sciences, University of Cambridge, Cambridge CB2 3EQ, UK; Department of Earth Sciences, University of Cambridge, Cambridge CB2 3EQ, UK; Girton College, Cambridge CB3 0JG, UK; American Museum of Natural History, NY, NY 10024, USA; Department of Earth Sciences, University of Cambridge, Cambridge CB2 3EQ, UK; Museum of Zoology, University of Cambridge, Cambridge CB2 3EJ, UK

## Abstract

Phylogenetic analyses of phenotypic characters in crown-group birds often recover results that are strongly incongruous with the findings of recent phylogenomic analyses. Furthermore, existing morphological datasets for crown birds are frequently limited by restricted taxon or character sampling, inconsistent character construction, incorrect scoring, or a combination of several of these factors. As part of an effort to address these limitations, in this study we focus on identifying phylogenetically informative characters of the avian pectoral girdle and forelimb skeleton, elements of which are commonly preserved as avian fossils. We assembled and vetted a dataset of 203 characters, which were then scored for a phylogenetically diverse range of 75 extant avian taxa and incorporated into phylogenetic analyses. Analyses run without topological constraints exhibited notable conflicts with the results of recent phylogenomic studies, possibly due to functional convergence and rapid cladogenesis in the early evolutionary history of crown birds. Qualitative anatomical comparisons and quantitative metrics of homoplasy further highlighted the fact that similar morphologies in pectoral girdle and forelimb elements have evolved repeatedly in distantly related groups of birds, representing a major confounding factor in avian morphological phylogenetics. However, the implementation of molecular scaffolds allowed the identification of diagnostic character combinations for numerous avian clades previously only recognized through molecular data, such as Phaethontimorphae, Aequornithes, and Telluraves. Although large morphological datasets may not guarantee increased congruence with molecular phylogenetic studies, they can nonetheless be valuable tools for identifying anatomical synapomorphies of key clades, placing fossils into phylogenetic context, and studying macroevolutionary patterns within major groups of organisms.

## Chinese abstract

分析表型特徵判斷冠群鳥類的譜系時經常獲得與近期譜系基因組學研究強烈不一致的結果。此外, 現有的冠群鳥類形態特徵資料集常受到分類單元或特徵採樣受限、特徵構建不一致、特徵評分錯誤等因素組合的限制。為了減少這些不足之處, 本研究注重於辨識鳥類肩帶和前肢骨骼中具有譜系資訊的特徵;此身體區域包含一些較常保存為鳥類化石的骨頭。我們根據75 種現存鳥類整理並審查出 203 個骨骼特徵的資料集, 對其進行譜系分析。在樹形未約束的情況下分析所產生的結果與近期譜系基因組學研究有明顯衝突, 這可能由冠群鳥類早期演化史中功能形態趨同演化及快速支序分枝所導致。定性解剖學上的比較和定量的同源性指標進一步強調在親緣關係遠的鳥類支序群中, 肩帶和前肢骨骼反覆演化出相似形態的模式, 代表鳥類形態譜系學中的重要混雜因素。不過, 在樹形根據分子譜系學的結果約束之下, 我們能夠對於許多先前僅透過分子分析識別的鳥類支序群判斷出形態上的共衍徵組合, 例如鸏型態類 (Phaethontimorphae)、水鳥類(Aequornithes)和陸鳥類 (Telluraves)。雖然大型的形態特徵資料集未必能確保與分子譜系學研究的一致性, 但仍然有識別關鍵支序群的形態共衍徵、將化石納入譜系分析以及研究主要生物類群的宏觀進化模式的價值。

## Introduction

Recent large-scale phylogenomic studies have greatly clarified the phylogenetic interrelationships of crown birds (Jarvis et al. 2014; Prum et al. 2015; Reddy et al. 2017; Kuhl et al. 2021; Stiller et al. 2024; Wu et al. 2024b). However, areas of contention remain, particularly concerning the branching topology at the base of Neoaves, a clade encompassing over 95% of extant bird diversity (Reddy et al. 2017; Braun et al. 2019; Braun and Kimball 2021; Bravo et al. 2021; Gatesy and Springer 2022; Wang et al. 2022; Stiller et al. 2024; Wu et al. 2024b; Chen et al. 2025). Furthermore, analyses of existing avian anatomical datasets (e.g., Mayr and Clarke 2003; Livezey and Zusi 2007; Worthy et al. 2017; Musser and Cracraft 2019) frequently fail to recover well-supported clades found by phylogenomic studies. As morphological data are essential for assessing the affinities of most extinct taxa and identifying suitable fossil calibrations for estimating divergence times (Giribet 2015; Lee and Palci 2015), this incongruence between molecular and morphological phylogenetic analyses severely hampers our understanding of morphological evolution in the early history of crown birds.

Several outstanding shortcomings of existing avian morphological phylogenetic datasets have been identified. The largest such dataset to date, presented in Livezey and Zusi (2006), has been criticized for its limited sampling of fossil taxa (Mayr 2007a) and incorrect or doubtful character scorings (Mayr 2008a). Furthermore, most morphological datasets for crown birds are relatively limited in anatomical and phylogenetic scope, often incorporating few characters compared to the total anatomical disparity exhibited by the sampled taxa or targeted toward resolving the affinities of narrow avian subgroups instead of broader clades. Although Mayr (2008a) also interpreted the results of Livezey and Zusi (2007) as evidence that large morphological datasets potentially contain little phylogenetic signal, this contention has not been tested for crown bird phylogenetics using independent datasets constructed with the explicit aim of correcting the shortcomings of previous studies. In any case, the production of morphological datasets that encompass a truly representative sample of crown bird character and taxic diversity remains underexplored.

Despite the frequently conflicting phylogenetic signals arising from molecular and morphological sources of data, some recent research has suggested that analyses of avian osteological data can potentially produce more congruent phylogenetic topologies with molecular analyses than those of soft tissue characters (Sansom and Wills 2017; Callender-Crowe and Sansom 2022; though see Ericson and Qu 2025). Furthermore, past reassessments of avian morphological data considering strongly supported clades recovered by molecular analyses have often been successful in identifying previously overlooked character support for said groupings (e.g., Mayr 2004a; Manegold 2006; Mayr 2011a; Chen et al. 2019; McInerney et al. 2019; Mayr 2019a; Steell et al. 2023). The production of a comprehensive, improved morphological phylogenetic dataset for crown birds could therefore provide a robust independent avenue with which to assess avian phylogenetic relationships, as well as facilitate congruence between morphological and molecular sources of phylogenetic information.

As part of an initiative to assemble such a dataset, we present a new morphological dataset for crown birds focusing on skeletal characters from the pectoral girdle and forelimb. Long recognized as critical to understanding the evolutionary origins of the modern avian bauplan (e.g., Ostrom 1974; Olson and Feduccia 1979; Feduccia 1986; Jenkins 1993; Ostrom 1995; Middleton and Gatesy 2000; Gishlick 2001; Senter 2006; Dececchi and Larsson 2009; Nesbitt et al. 2009; Sullivan et al. 2010; Heers and Dial 2012; Zheng et al. 2012; Benson and Choiniere 2013; Botelho et al. 2014; Xu et al. 2014; Zheng et al. 2014; O'Connor et al. 2015; Heers 2016; Heers et al. 2016; Mayr 2017a; Nebreda et al. 2020; Novas et al. 2020; Cau et al. 2021; Heers et al. 2021; Karoullas and Nudds 2021; Novas et al. 2021; Lo Coco et al. 2022; Pittman et al. 2022; Wang et al. 2022; Eliason et al. 2023; Lowi-Merri et al. 2023; Wang and Zhou 2023; Widrig et al. 2023; Wu et al. 2024a; Field et al. 2025; Napoli et al. 2025; Nebreda et al. 2025; Wu et al. 2025), this anatomical region is of particular interest in avian morphological phylogenetics for several reasons. For one, though the fossil record of birds is often regarded as relatively sparse, some elements of the avian pectoral girdle and forelimb, such as the coracoid, are commonly recovered in fossil assemblages that preserve remains of birds and their close relatives (e.g., Longrich 2009; Longrich et al. 2011). In addition, several elements from this anatomical region have been assessed for phylogenetic signals in previous ornithological studies (Fürbringer 1888; Höfling and Alvarenga 2001; Wang and Clarke 2014; Mayr 2014a; Shatkovska and Ghazali 2017; Steell et al. 2023; Shimizu and Anezaki 2024), and characters from the pectoral girdle and forelimb account for over 30% of the characters used in some phylogenetic datasets focused on crown birds and near-crown stem-birds (Brocklehurst et al. 2012).

## Methods and materials

### Phylogenetic dataset

We assembled a phylogenetic dataset of 203 morphological characters from the pectoral girdle and forelimb skeleton. Characters were sourced from direct anatomical observations as well as numerous previous studies (primarily Mayr and Clarke 2003; Livezey and Zusi 2006; Smith 2010; Worthy et al. 2017; Ksepka et al. 2019; Musser and Cracraft 2019; Musser and Clarke 2020), and modified based on guidelines for character construction put forth by Hawkins et al. (1997), Sereno (2007), and Simões et al. (2017). We recognize the potential methodological problems and challenges of discretizing characters founded on continuous morphological variation, especially in the absence of well-understood state delimitations (e.g., Goloboff et al. 2006; Randle and Sansom 2017; Simões et al. 2017), but have chosen to retain some characters of this nature in our dataset (e.g., chars. 9, 15, 35, 52, 53, 74, 75, 81, 99, 162, 170,176, 180, 181, 201) because discretized continuous characters remain widely used in vertebrate morphological phylogenetics and these traits contain potential phylogenetic signal. Characters from nearly all bony elements of the avian pectoral girdle and forelimb were included, with the exception of the smaller manual phalanges, which are frequently lost in fossil specimens and prepared skeletons of extant species. Complete character descriptions are available as Supplementary Material S1 and measurements used for scoring char. 99 are included in Supplementary Material S2.

**Fig. 1:**
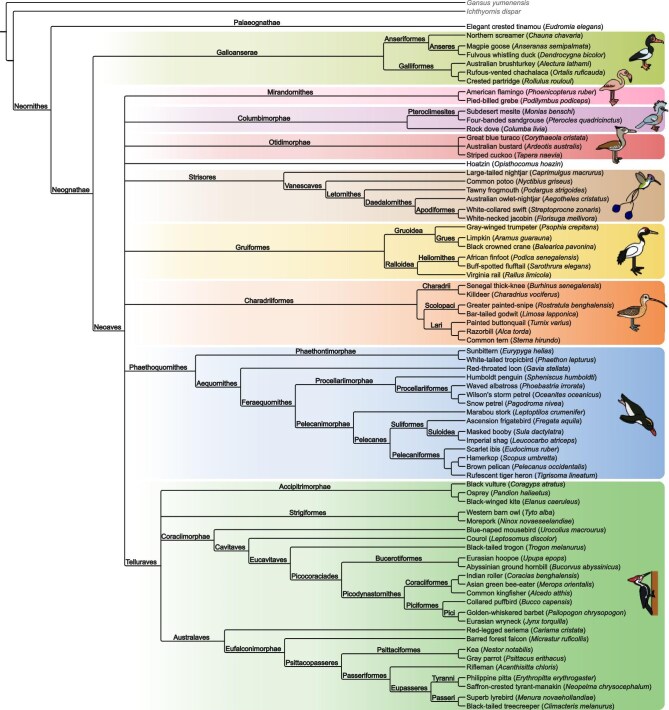
Phylogenetic consensus tree of the species sampled in this study, summarized based on recent phylogenomic studies (Jarvis et al. 2014; Prum et al. 2015; Kuhl et al. 2021; Stiller et al. 2024; Wu et al. 2024b) and labeled with names of higher-order clades. English names largely follow Gill et al. (2024), though the name “courol” is used for *Leptosomus discolor* instead of “cuckoo-roller” to emphasize its phylogenetic distinctiveness from true rollers (Coraciidae). Illustrations depict representatives of major clades, but not necessarily the precise species examined in the present study.

Our dataset includes 77 taxa, including the Cretaceous stem-birds *Gansus* and *Ichthyornis* as well as 75 extant species broadly sampling crown bird diversity (Fig. 1). Only taxa that use the forelimbs in propulsive locomotion (powered aerial flight or wing-propelled diving) were considered for this study, as the convergent reduction of pectoral and forelimb elements in flightless birds has well-known confounding effects on phylogenetic reconstruction (Worthy and Scofield 2012; Worthy et al. 2017). Taxa were scored primarily based on personal examination of computed-tomography (CT) scans published by Bjarnason and Benson (2021) and Benito et al. (2022a), with the exception of *Gansus*, which was scored based on published fossil descriptions (You et al. 2006; Li et al. 2011; Wang et al. 2016). A complete list of specimens examined is provided in Supplementary Material S3. Homology of structures in the highly modified humerus and antebrachial bones of *Spheniscus* with those of other birds was inferred based on muscular arrangements described by Watanabe et al. (2021). For some extant taxa, the unfused carpals and/or manual phalanges were absent from the specimens we examined and the characters pertaining to these elements were scored as unknown. Our complete phylogenetic matrix is available as Supplementary Material S4.

**Fig. 2:**
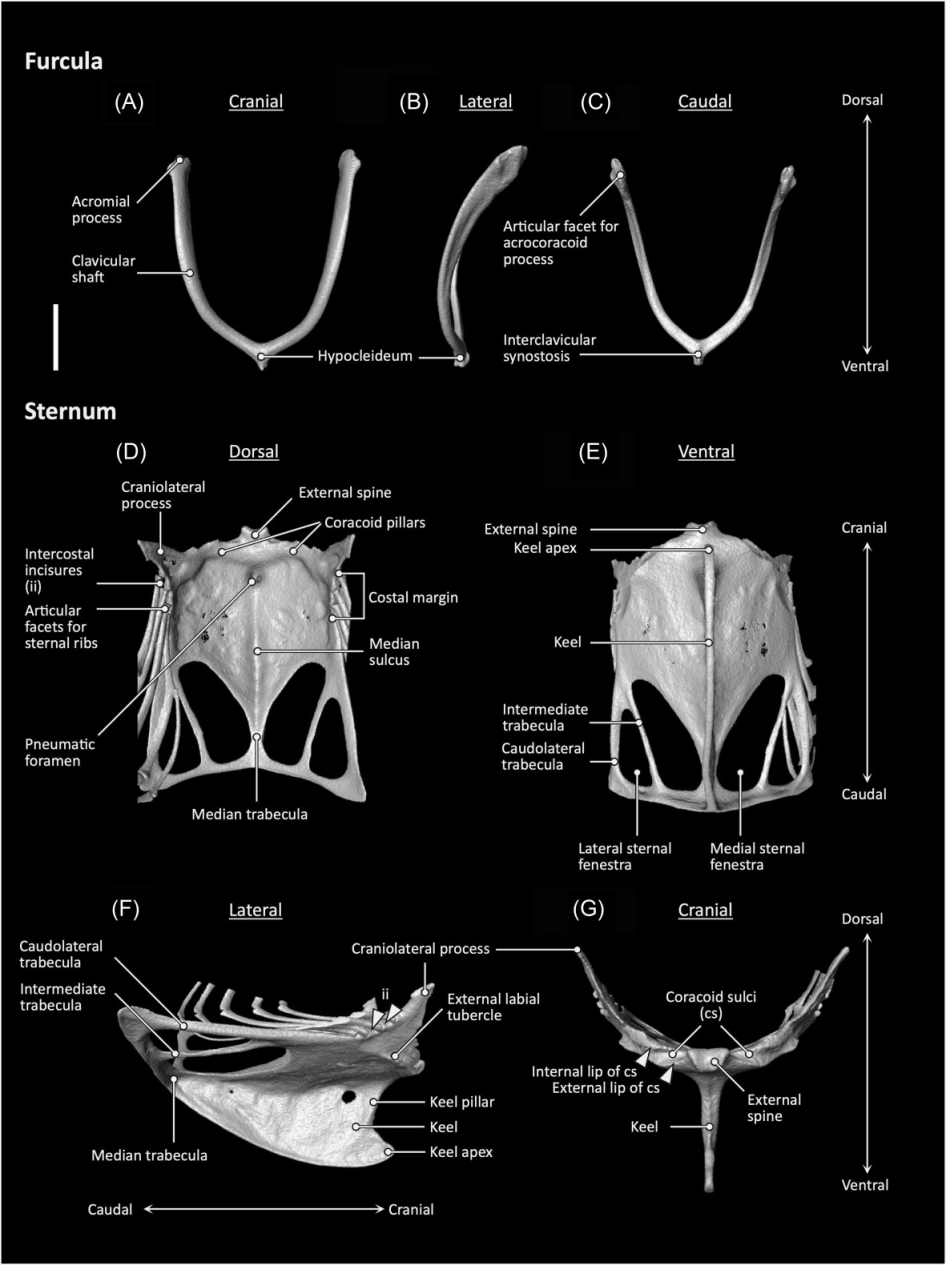
Furcula (**A–C**) and sternum (**D–G**) anatomy of *Aegotheles cristatus* (Australian owlet-nightjar, YPM 124258). Scale bar = 5 mm.

**Fig. 3:**
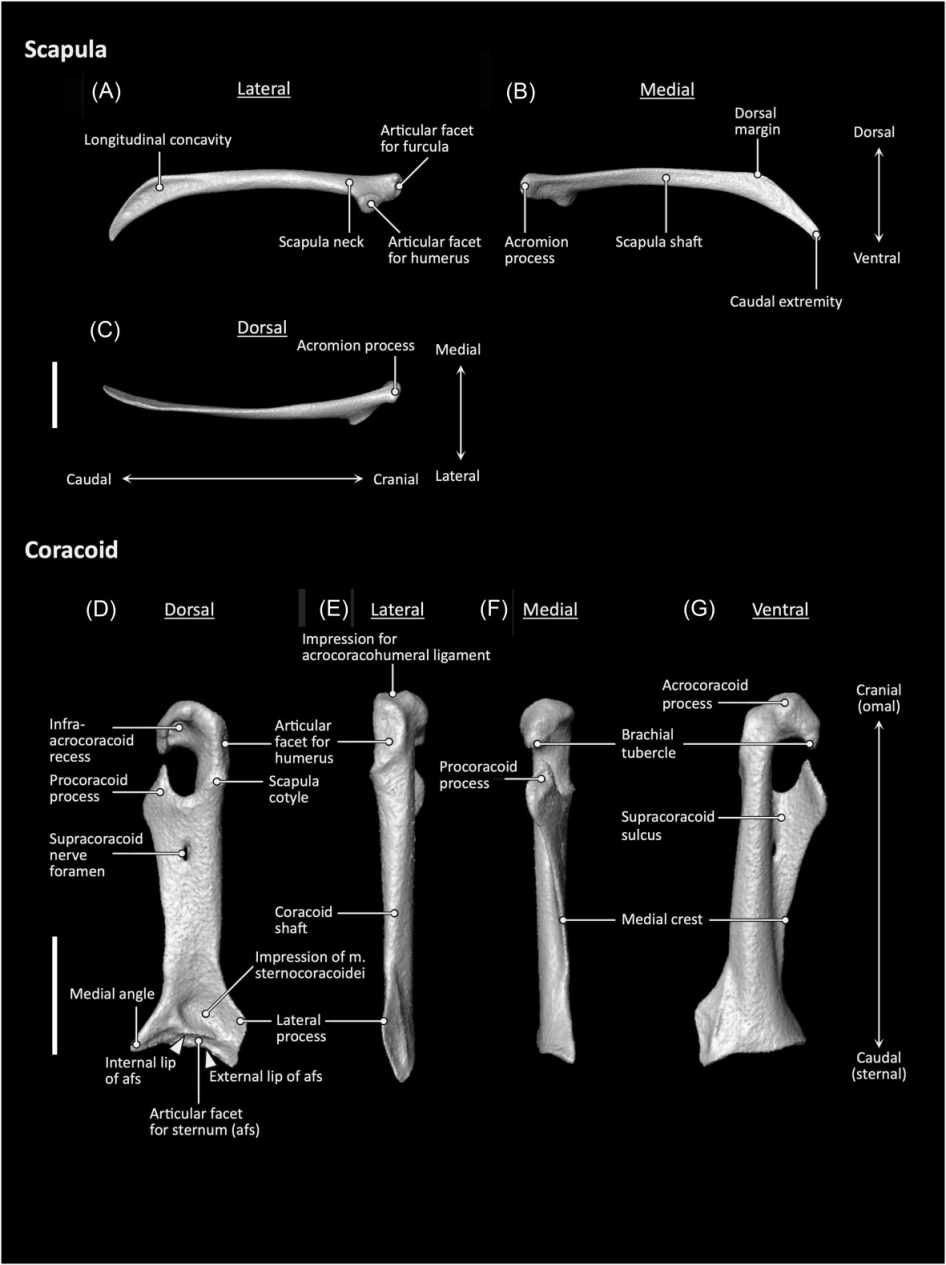
Scapula (**A–C**) and coracoid (**D–G**) anatomy of *Aegotheles cristatus* (Australian owlet-nightjar, YPM 124258). Scale bars = 5 mm.

**Fig. 4:**
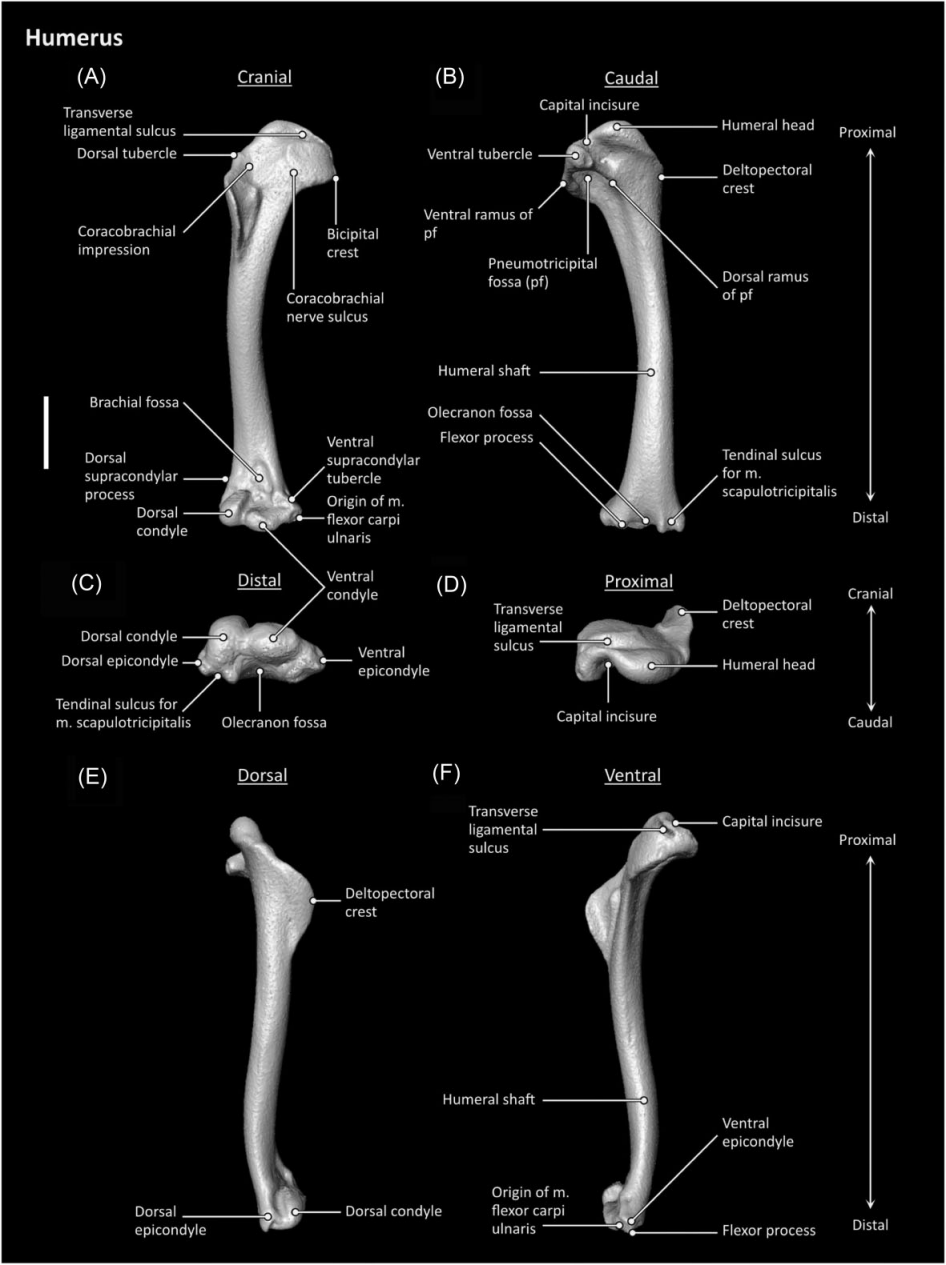
Humerus anatomy of *Aegotheles cristatus* (Australian owlet-nightjar, YPM 124258). Scale bar = 5 mm.

**Fig. 5:**
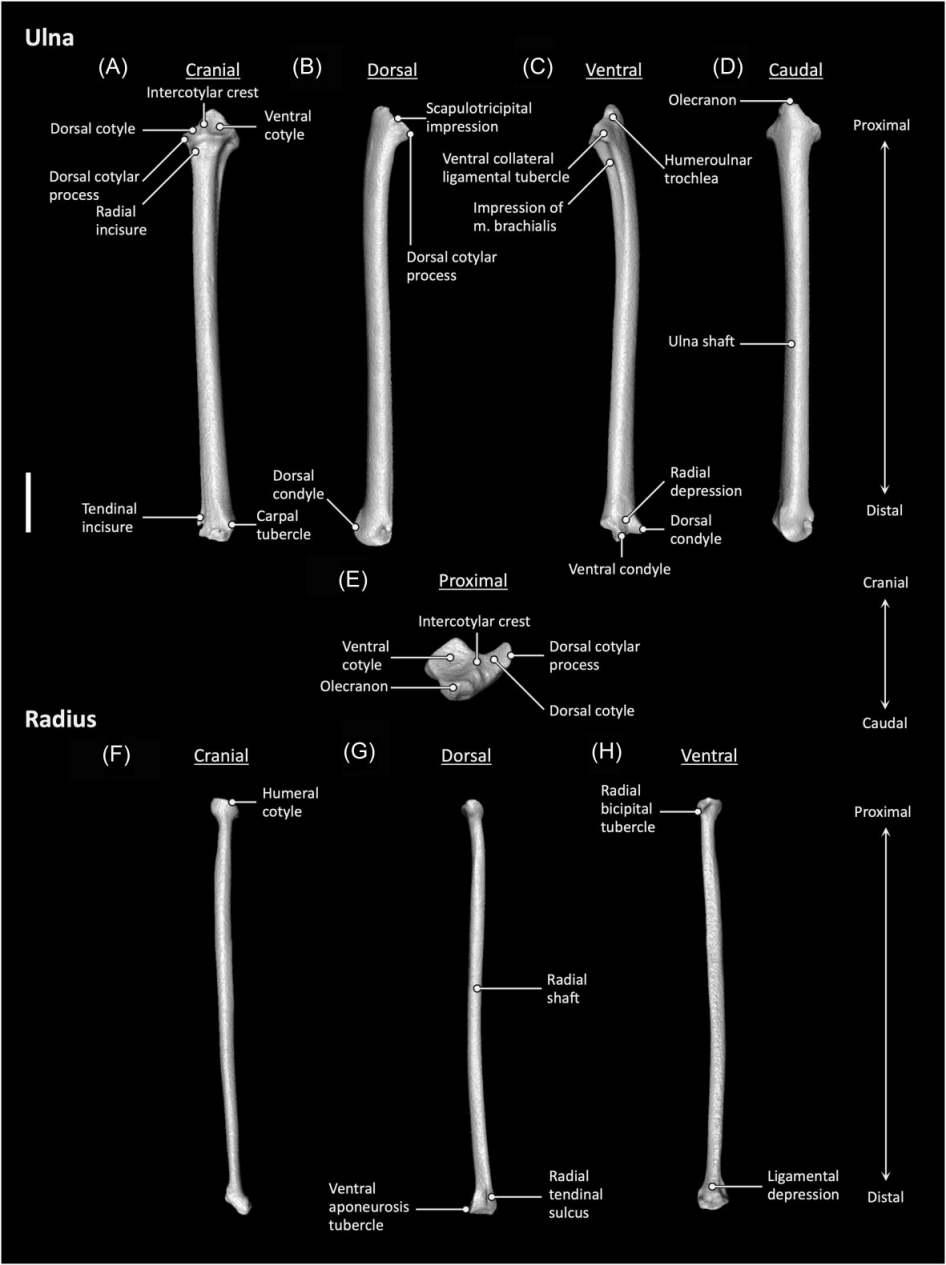
Ulna (**A–E**) and radius (**F–H**) anatomy of *Aegotheles cristatus* (Australian owlet-nightjar, YPM 124258). Scale bar = 5 mm.

**Fig. 6:**
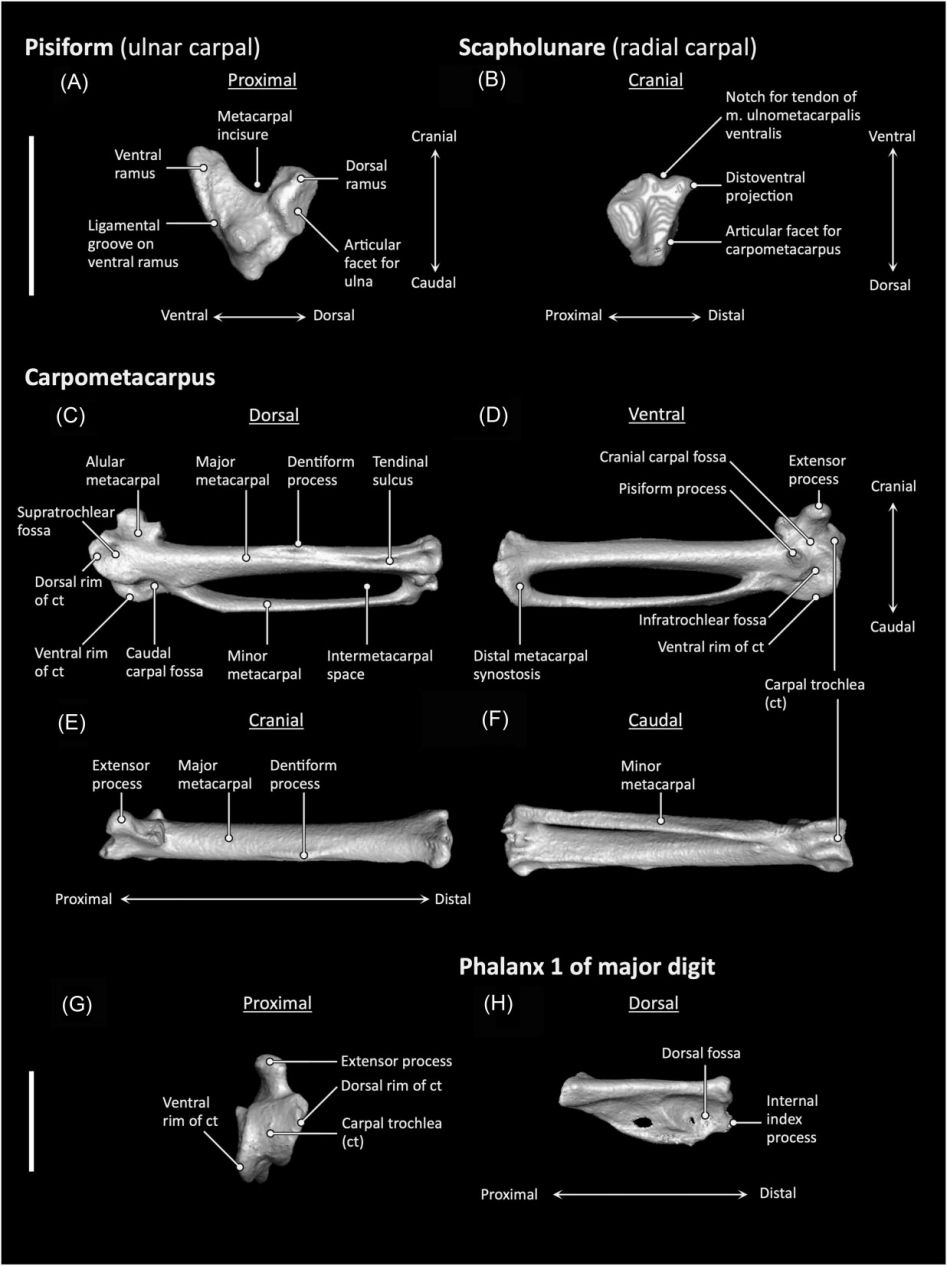
Pisiform (**A**), scapholunare (**B**), carpometacarpus (**C–G**), and manual phalanx II-1 (**H**) anatomy of *Aegotheles cristatus* (Australian owlet-nightjar, YPM 124258). Scale bars = 5 mm.

Anatomical terminology follows the English equivalents of terms used by Baumel and Witmer (1993), with some exceptions as follows. “Lateral trabeculae” of the sternum are called “caudolateral trabeculae” here, following Livezey and Zusi (2006) to avoid confusion with the apomorphic, laterally projecting sternal processes found in galliforms, which we term “lateral branches of the caudolateral trabeculae.” The radial carpal (“radiale”) and ulnar carpal (“ulnare”) are referred to here as the “scapholunare” and “pisiform” respectively, following the recommendations of Botelho et al. (2014). Anatomical features referenced within this study are illustrated in Figs. 2–6.

Where applicable, taxon names for major bird clades follow the phylogenetic definitions of Sangster (2020a), Sangster (2020b), Chen and Field (2020), Mindell (2020), Sangster and Mayr (2021), and Sangster et al. (2022). A phylogenetic consensus tree and nomenclatural framework for the taxa included in this study are shown in Fig. 1.

### Phylogenetic analyses

**Fig. 7:**
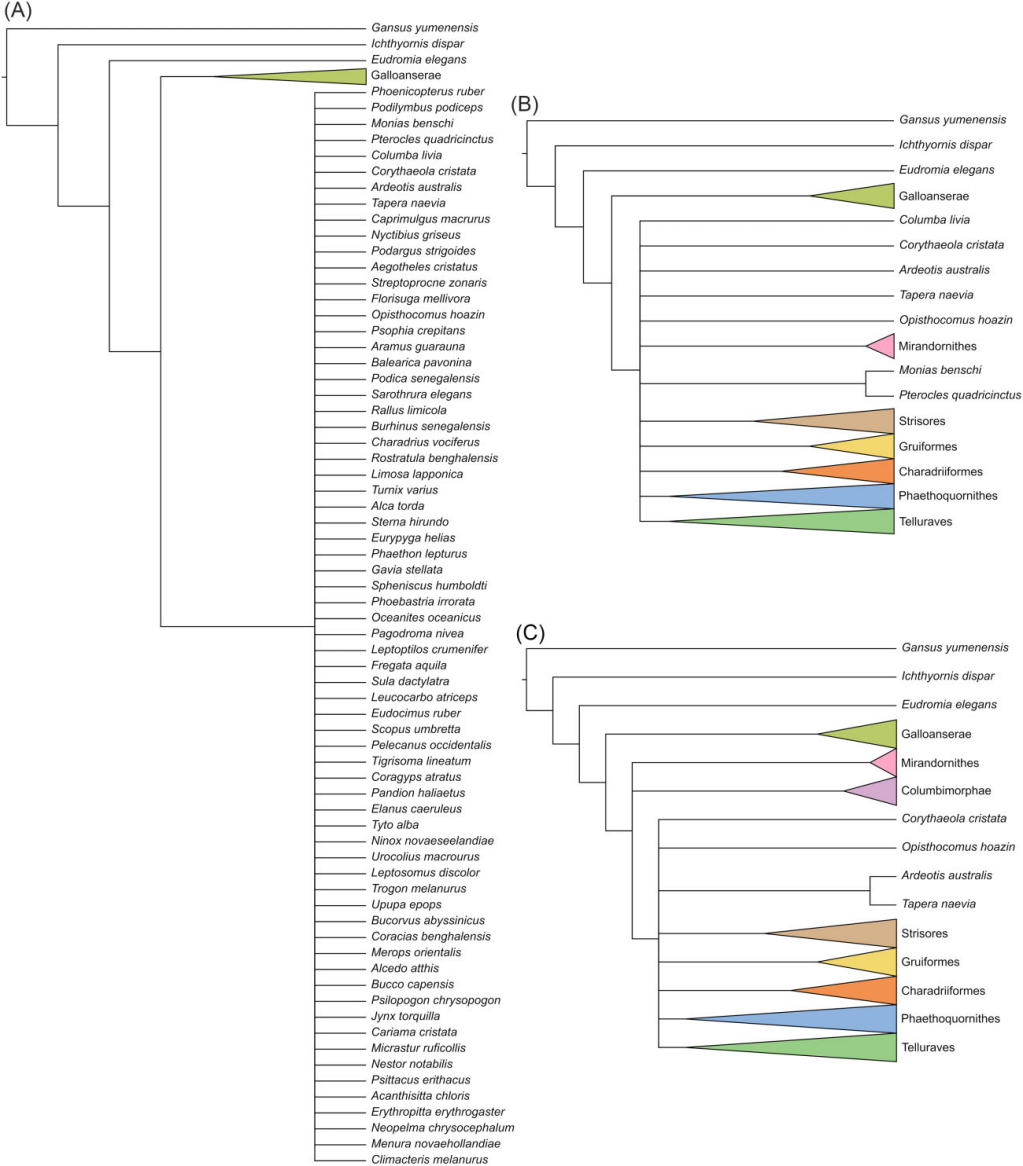
Topological constraints used for Analyses 4–6 (**A**), Analyses 7–9 (**B**), and Analyses 10–12 (**C**). Where constrained to be monophyletic, select major avian clades are collapsed and highlighted following the color coding shown in Fig. 1. Internal relationships of collapsed groups can be assumed to follow those shown in Fig. 1. Further details of analytical parameters are discussed in the main text.

Twelve different analyses were run under alternative topological constraints and search parameters, as described in Table 1. Details of topological constraints are visualized in Fig. 7.

**Table 1:** Parameters for phylogenetic analyses run in the present study

	Analytical method	Topological constraints
Analysis 1	Equal weights parsimony	None
Analysis 2	Implied weights parsimony	None
Analysis 3	Bayesian inference (Mkv model)	None
Analysis 4	Equal weights parsimony	Outgroup relationships to Neoaves constrained based on current phylogenetic consensus (Fig. 7A)
Analysis 5	Implied weights parsimony	Outgroup relationships to Neoaves constrained based on current phylogenetic consensus
Analysis 6	Bayesian inference (Mkv model)	Outgroup relationships to Neoaves constrained based on current phylogenetic consensus
Analysis 7	Equal weights parsimony	Strict consensus of results from recent phylogenomic studies (Fig. 7B; Jarvis et al. 2014; Prum et al. 2015; Kuhl et al. 2021; Stiller et al. 2024; Wu et al. 2024b)
Analysis 8	Implied weights parsimony	Strict consensus of results from recent phylogenomic studies
Analysis 9	Bayesian inference (Mkv model)	Strict consensus of results from recent phylogenomic studies
Analysis 10	Equal weights parsimony	Consensus tree proposed by Braun and Kimball (2021) (Fig. 7C)
Analysis 11	Implied weights parsimony	Consensus tree proposed by Braun and Kimball (2021)
Analysis 12	Bayesian inference (Mkv model)	Consensus tree proposed by Braun and Kimball (2021)

Maximum parsimony analyses were conducted in TNT v.1.5 (Goloboff and Catalano 2016). After increasing the maximum number of trees to 99,999, a new technology search was run in which a minimum length tree was found in 1000 replicates and default parameters were set for sectorial search, ratchet, tree drift and tree fusion. Implied weights analyses were run with a *k* (concavity constant) value of 12, following Goloboff et al. (2018). Absolute bootstrap frequencies were obtained from 1000 replicates under a traditional search with default parameters.

Bayesian phylogenetic analyses were conducted in MrBayes (Ronquist et al. 2012) using the CIPRES Science Gateway (Miller et al. 2010). Data were analyzed under the Mkv model (Lewis 2001), and gamma-distributed rate variation was assumed to allow for evolutionary rate heterogeneity across characters. Analyses were conducted using four chains and two independent runs for 30,000,000 generations, with a tree sampled every 4000 generations and a burn-in of 25%. Analytical convergence was assessed using standard diagnostics provided in MrBayes (average standard deviation of split frequencies <0.01, potential scale reduction factors = 1, effective sample sizes >200). Results of independent runs of the same analyses were summarized using the sump and sumt commands. To facilitate comparisons with fully resolved topologies, the “contype = allcompat” command was used to display clades that were present in less than 50% of postburnin trees but were compatible with the 50% majority-rule consensus tree.

### Comparisons with molecular topologies

As a measure of tree distance, the Robinson–Foulds (RF) index comparing the most parsimonious trees and the “allcompat” consensus trees recovered by analyses of the morphological dataset with the molecular phylogenetic results of Jarvis et al. (2014), Prum et al. (2015), Kuhl et al. (2021), Stiller et al. (2024), and Wu et al. (2024b) were calculated in R 4.3.1 (R Core Team 2023) using the package phangorn (Schliep 2011). The data and R code used for calculating RF indices is available as Supplementary Material S5. Synapomorphies of major clades recovered by molecular phylogenetic analyses were inferred under maximum parsimony by mapping the morphological dataset onto the aforementioned molecular topologies in TNT and ASADO v.1.61 (Nixon 2002). Tree lengths based on the inferred number of morphological changes under molecular topologies were also calculated in TNT.

To visualize the structure of the morphological dataset relative to molecular topologies, heatmaps of similarity matrices were produced from our phylogenetic matrix following the procedure outlined by Evers and Benson (2019). Similarity matrices were calculated for the complete morphological dataset as well as partitions of that dataset pertaining to individual elements for which over ten characters were coded, and colors were assigned to the similarity values of each matrix using the package gclus (Hurley 2019). *Monias benschi* was excluded from the similarity matrix calculated for furcular characters due to its lack of an ossified furcula. The data and R code for calculating similarity and producing the heatmaps are available as Supplementary Material S6.

### Measuring homoplasy

We applied parsimony-based indices (consistency index [Kluge and Farris 1969], retention index [Farris 1989] and relative homoplasy index [Steell et al. 2025]) to quantify homoplasy in the present dataset with the same molecular constraint topologies as above. For comparison, we also estimated homoplasy in additional published extant bird matrices covering several taxonomic scales: Mayr and Clarke (2003), Livezey and Zusi (2007), Field et al. (2020), and Musser and Clarke (2020) representing general Neornithes matrices; Ksepka et al. (2023a) representing Galliformes; Chen et al. (2019) representing Strisores; Ksepka et al. (2023b) representing Sphenisciformes; Ksepka et al. (2019) representing Telluraves; and Steell et al. (2023) representing Passeriformes. Recent molecular topological constraints were applied to each matrix, and when applicable, multiple constraints were applied based on the differing topologies of Jarvis et al. (2014), Prum et al. (2015), and Stiller et al. (2024). For the galliform-specific dataset of Ksepka et al. (2023a), molecular constraints followed Kimball et al. (2021), and for the sphenisciform-specific dataset of Ksepka et al. (2023b), molecular constraints followed Pan et al. (2019) and Vianna et al. (2020). Analyses of the datasets from Chen et al. (2019) and Steell et al. (2023) followed scaffolds employed in those respective studies. Where appropriate, we removed all fossil taxa from comparison matrices prior to analysis.

We applied the relative homoplasy index (RHI) (Steell et al. 2025) to all applicable molecular topologies per matrix. The RHI measures the amount of homoplasy in a matrix with a given topology against a null distribution of topologies with an estimated maximum amount of homoplasy, therefore enabling accurate comparisons across datasets with different taxon and character samples (Steell et al. 2025). RHI calculations and matrix preparation were carried out in R 4.3.1 (R Core Team 2023). All code and custom functions used are available from Supplementary Material S7. The main dependencies are the packages phangorn (Schliep 2011), APE (Paradis and Schliep 2019), and phytools (Revell 2024). In many cases, the removal of fossil taxa produced invariant characters which were removed prior to analysis. See Supplementary Material S7 for specific characters removed in each case, except for the Livezey and Zusi (2007) dataset, in which 659 characters were removed due to the large number of nonavian reptile taxa in that matrix. For simplicity, all characters were treated as unordered, polymorphisms, and uncertainties were treated the same with the most parsimonious state in the polymorphism being used in the step count, and “gaps” (“-”) were treated as ambiguities (“?”). In addition to RHI, consistency (CI), and retention index (RI) were also calculated using phangorn.

## Results

### Phylogenetic analyses

**Fig. 8:**
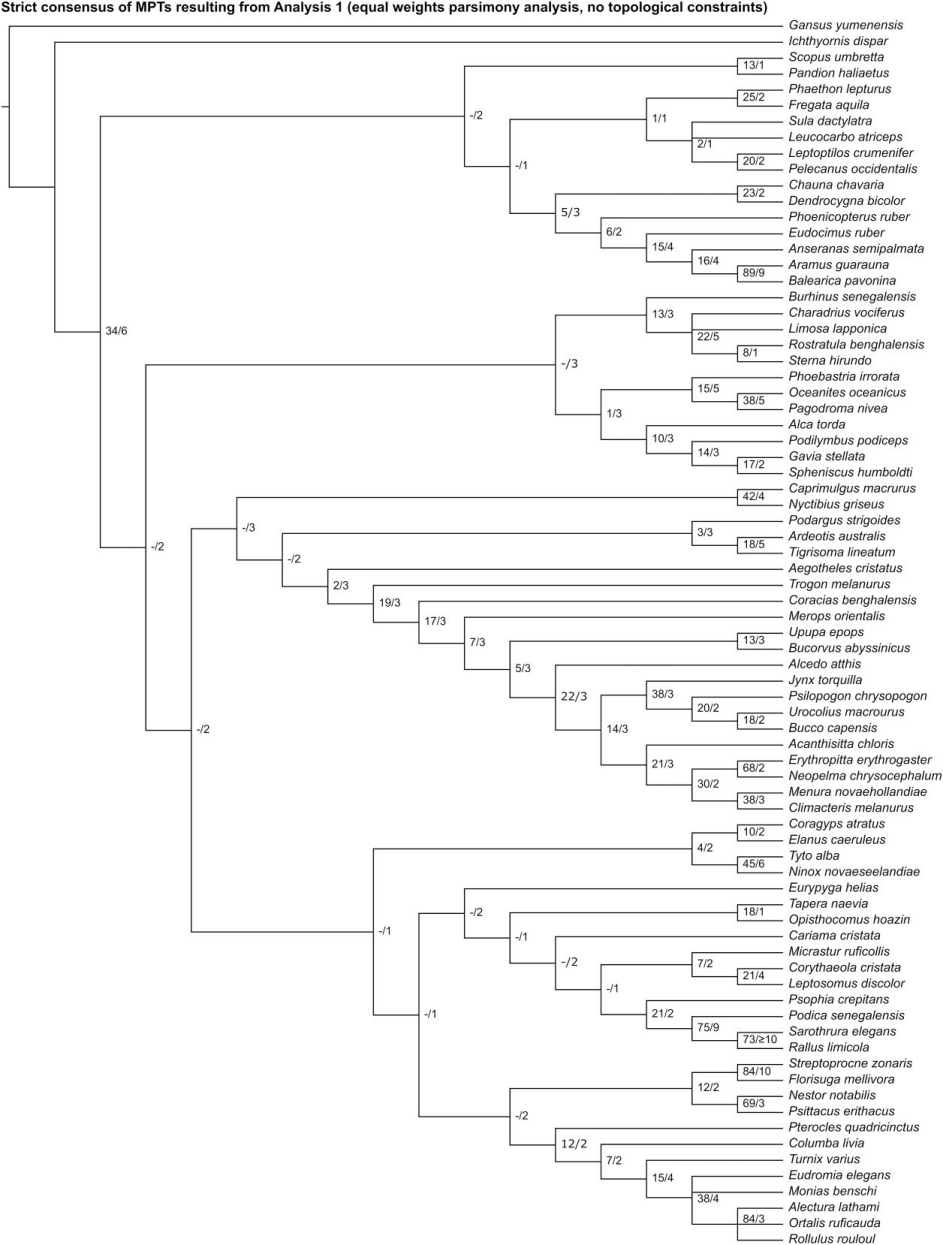
Strict consensus of most parsimonious trees resulting from Analysis 1 (equal weights parsimony analysis without topological constraints). Numbers on nodes represent absolute bootstrap frequencies (left of bar) and Bremer support values (right of bar). A hyphen (-) denotes values of 0.

Analysis 1 recovered six most parsimonious trees (MPT) of 2483 steps (Fig. 8), with a consistency index (CI) of 0.098 and a retention index (RI) of 0.421. The strict consensus of all MPTs bears little topological resemblance to recent phylogenetic consensus. Instead of the universally accepted split between paleognaths and neognaths, a clade uniting an assemblage of mainly aquatically adapted taxa (including *Phoenicopterus, Phaethon, Pandion*, and members of Anseriformes, Grues, and Pelecanimorphae) was recovered as the sister group to all other crown birds. Fourteen nodes consistent with current phylogenetic consensus were resolved: the exclusion of *Ichthyornis* from Neornithes, Galliformes (*Alectura* + *Ortalis* + *Rollulus*), Apodiformes (*Streptoprocne* + *Florisuga*), Grues (*Aramus* + *Balearica*), Ralloidea (*Podica* + *Sarothrura* + *Rallus*), Procellariiformes (*Phoebastria* + *Oceanites* + *Pagodroma*), *Oceanites* + *Pagodroma*, Strigiformes (*Tyto* + *Ninox*), Bucerotiformes (*Upupa* + *Bucorvus*), Psittaciformes (*Nestor* + *Psittacus*), Passeriformes (*Acanthisitta* + *Erythropitta* + *Neopelma* + *Menura* + *Climacteris*), Eupasseres (*Erythropitta* + *Neopelma* + *Menura* + *Climacteris*), Tyranni (*Erythropitta* + *Neopelma*), and Passeri (*Menura* + *Climacteris*).

**Fig. 9:**
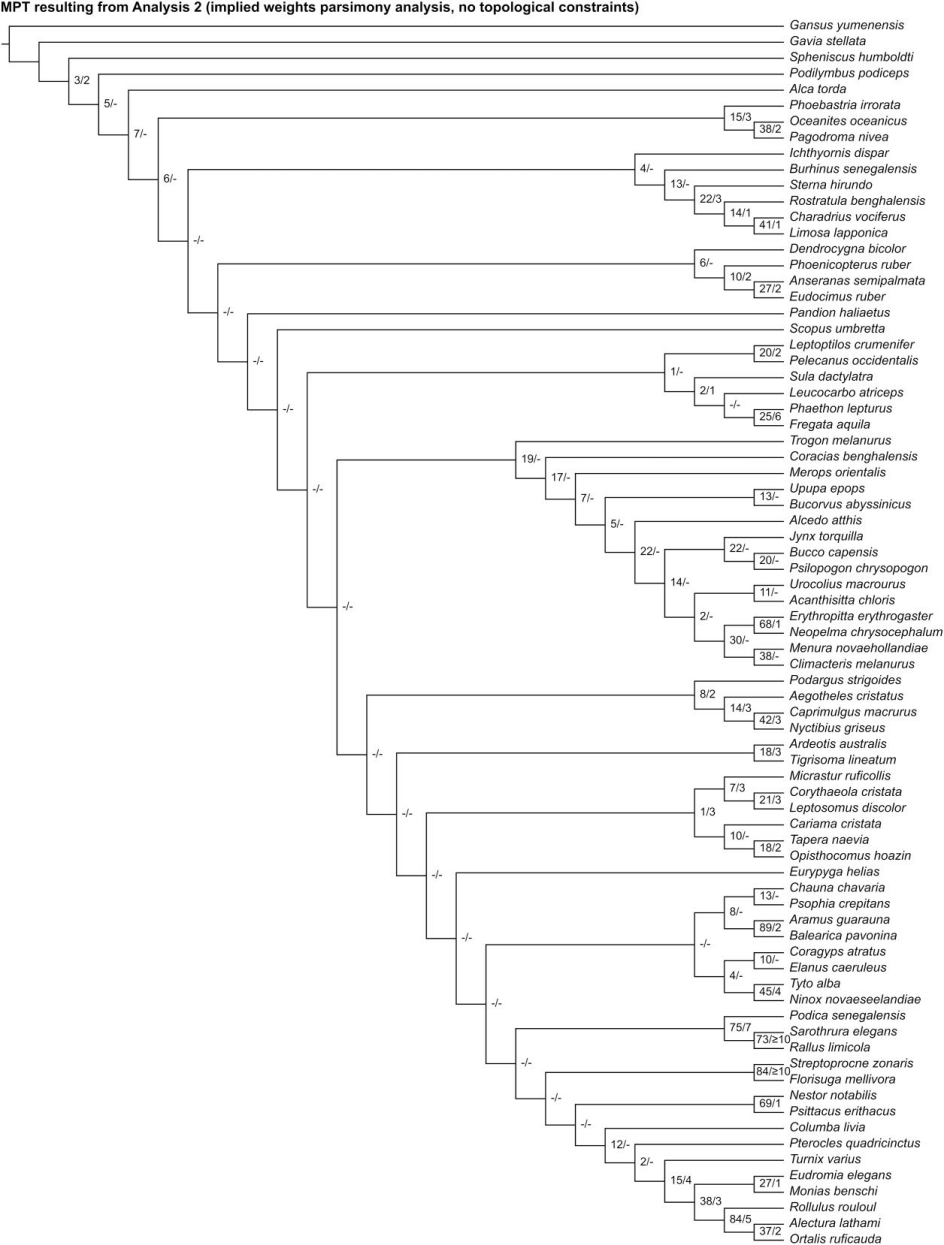
The most parsimonious tree resulting from Analysis 2 (implied weights parsimony analysis without topological constraints). Numbers on nodes represent absolute bootstrap frequencies (left of bar) and Bremer support values (right of bar). A hyphen (-) denotes values of 0.

Analysis 2 recovered one MPT of 2498 steps (Fig. 9), with a CI of 0.097 and a RI of 0.417. This tree is also highly incongruent with current knowledge of avian phylogenetic relationships. *Gavia* was recovered as the sister group to all other crown birds and *Ichthyornis* was recovered within the avian crown group, closely related to a clade comprising several members of Charadriiformes. Thirteen nodes consistent with current phylogenetic consensus were resolved: Galliformes (*Alectura* + *Ortalis* + *Rollulus*), Apodiformes (*Streptoprocne* + *Florisuga*), Grues (*Aramus* + *Balearica*), Ralloidea (*Podica* + *Sarothrura* + *Rallus*), Procellariiformes (*Phoebastria* + *Oceanites* + *Pagodroma*), *Oceanites* + *Pagodroma*, Strigiformes (*Tyto* + *Ninox*), Bucerotiformes (*Upupa* + *Bucorvus*), Piciformes (*Bucco* + *Psilopogon* + *Jynx*), Psittaciformes (*Nestor* + *Psittacus*), Eupasseres (*Erythropitta* + *Neopelma* + *Menura* + *Climacteris*), Tyranni (*Erythropitta* + *Neopelma*), and Passeri (*Menura* + *Climacteris*).

**Fig. 10:**
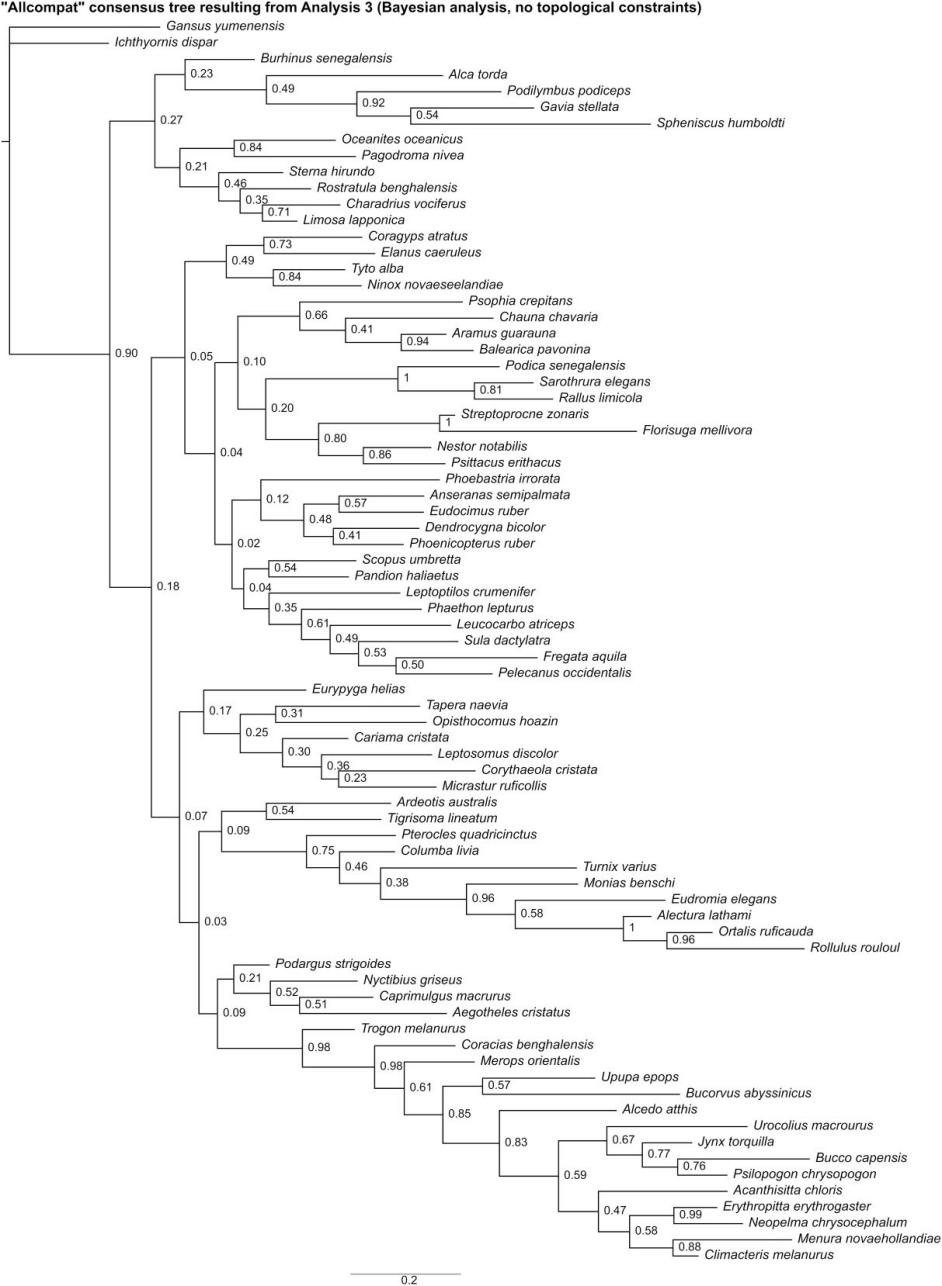
The “allcompat” consensus tree resulting from Analysis 3 (Bayesian analysis without topological constraints). Numbers on nodes represent posterior probabilities.

In the “allcompat” consensus tree recovered by Analysis 3 (Fig. 10), a mainly aquatically adapted clade consisting of *Podilymbus, Gavia, Spheniscus*, and members of Charadriiformes and Procellariiformes was placed as the sister group to all other crown birds, though posterior probability for this relationship was well below 50%. Fourteen nodes consistent with current phylogenetic consensus were resolved with posterior probabilities ≥50%: the exclusion of *Ichthyornis* from Neornithes, Galliformes (*Alectura* + *Ortalis* + *Rollulus*), *Ortalis* + *Rollulus*, Apodiformes (*Streptoprocne* + *Florisuga*), Grues (*Aramus* + *Balearica*), Ralloidea (*Podica* + *Sarothrura* + *Rallus*), *Oceanites* + *Pagodroma*, Strigiformes (*Tyto* + *Ninox*), Bucerotiformes (*Upupa* + *Bucorvus*), Piciformes (*Bucco* + *Psilopogon* + *Jynx*), Psittaciformes (*Nestor* + *Psittacus*), Eupasseres (*Erythropitta* + *Neopelma* + *Menura* + *Climacteris*), Tyranni (*Erythropitta* + *Neopelma*), and Passeri (*Menura* + *Climacteris*).

**Fig. 11:**
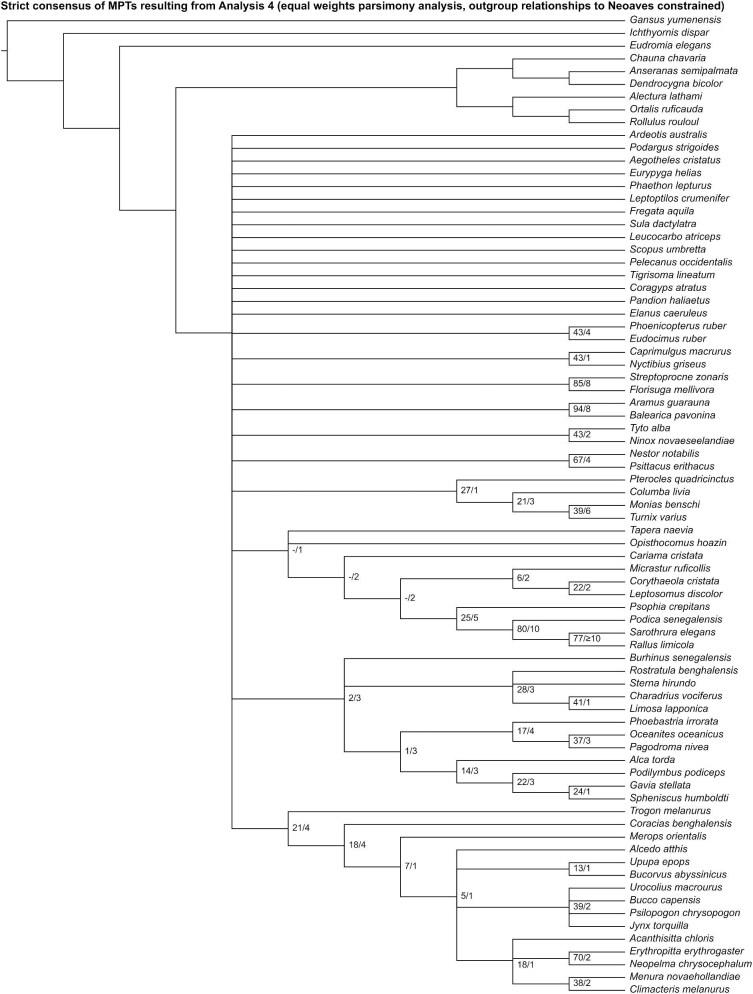
Strict consensus of most parsimonious trees resulting from Analysis 4 (equal weights parsimony analysis run with outgroup relationships to Neoaves constrained). Numbers on nodes represent absolute bootstrap frequencies (left of bar) and Bremer support values (right of bar). A hyphen (-) denotes values of 0. Nodes lacking support values were constrained to be monophyletic.

Analysis 4 recovered 15 MPTs of 2524 steps (Fig. 11), with a CI of 0.096 and a RI of 0.411. The strict consensus of all trees was poorly resolved, but 11 unconstrained nodes consistent with current phylogenetic consensus were recovered: Apodiformes (*Streptoprocne* + *Florisuga*), Grues (*Aramus* + *Balearica*), Ralloidea (*Podica* + *Sarothrura* + *Rallus*), Procellariiformes (*Phoebastria* + *Oceanites* + *Pagodroma*), *Oceanites* + *Pagodroma*, Strigiformes (*Tyto* + *Ninox*), Bucerotiformes (*Upupa* + *Bucorvus*), Psittaciformes (*Nestor* + *Psittacus*), Passeriformes (*Acanthisitta* + *Erythropitta* + *Neopelma* + *Menura* + *Climacteris*), Tyranni (*Erythropitta* + *Neopelma*), and Passeri (*Menura* + *Climacteris*). Other results that broadly align with those of recent phylogenetic studies include a clade uniting *Monias, Pterocles*, and *Columba* (though *Turnix* was also recovered as a member of this clade).

**Fig. 12:**
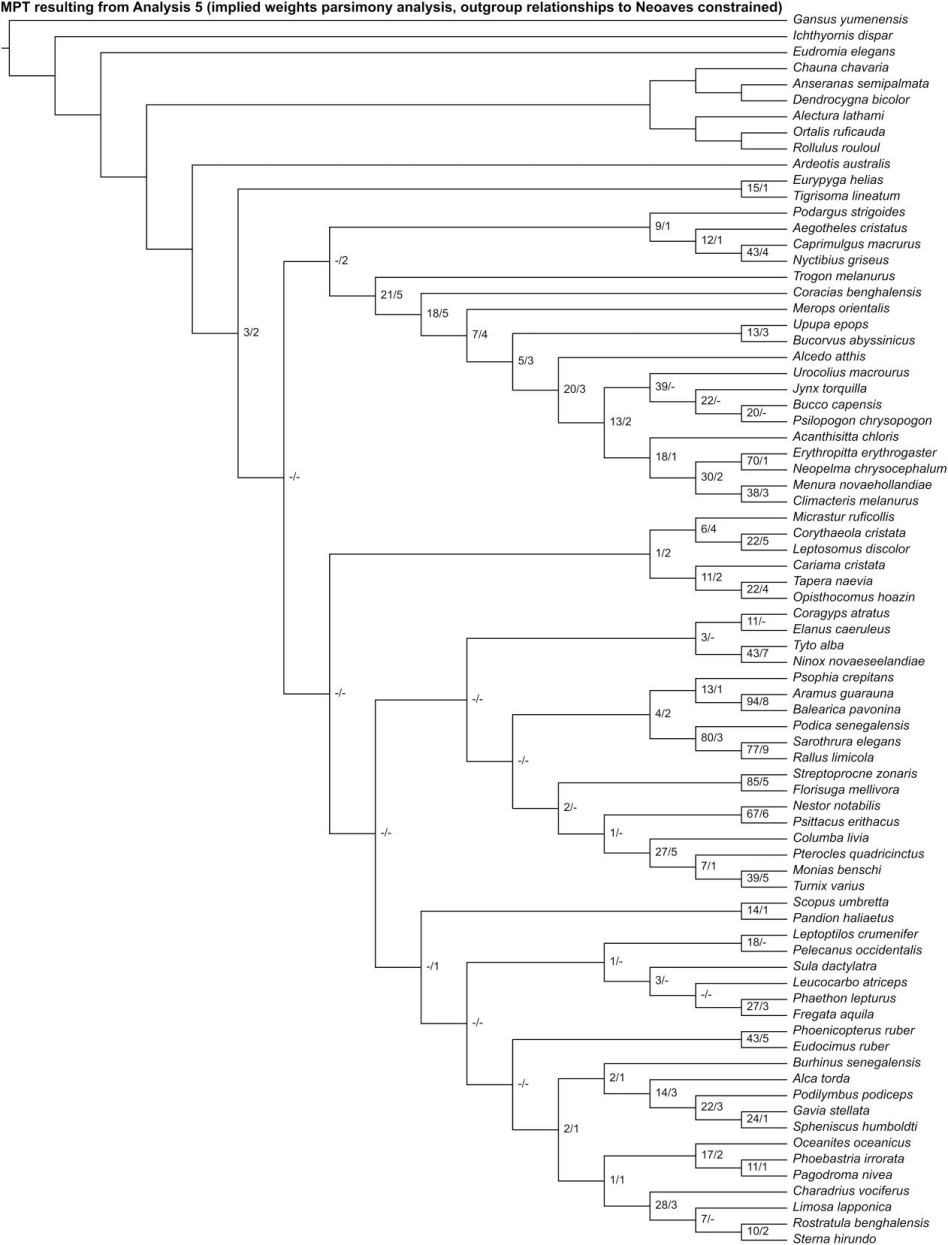
The most parsimonious tree resulting from Analysis 5 (implied weights parsimony analysis run with outgroup relationships to Neoaves constrained). Numbers on nodes represent absolute bootstrap frequencies (left of bar) and Bremer support values (right of bar). A hyphen (-) denotes values of 0. Nodes lacking support values were constrained to be monophyletic.

Analysis 5 recovered one MPT of 2544 steps (Fig. 12), with a CI of 0.096 and a RI of 0.407. *Ardeotis* was recovered as the sister group to all other neoavians. Fourteen unconstrained nodes consistent with current phylogenetic consensus were resolved: Apodiformes (*Streptoprocne* + *Florisuga*), Gruiformes (*Psophia* + *Aramus* + *Balearica* + *Podica* + *Sarothrura* + *Rallus*), Gruoidea (*Psophia* + *Aramus* + *Balearica*), Grues (*Aramus* + *Balearica*), Ralloidea (*Podica* + *Sarothrura* + *Rallus*), Procellariiformes (*Phoebastria* + *Oceanites* + *Pagodroma*), Strigiformes (*Tyto* + *Ninox*), Bucerotiformes (*Upupa* + *Bucorvus*), Piciformes (*Bucco* + *Psilopogon* + *Jynx*), Psittaciformes (*Nestor* + *Psittacus*), Passeriformes (*Acanthisitta* + *Erythropitta* + *Neopelma* + *Menura* + *Climacteris*), Eupasseres (*Erythropitta* + *Neopelma* + *Menura* + *Climacteris*), Tyranni (*Erythropitta* + *Neopelma*), and Passeri (*Menura* + *Climacteris*). Other results that broadly align with those of recent phylogenetic studies include a close relationship among *Monias, Pterocles*, and *Columba* (though *Turnix* was also recovered as a member of this clade).

**Fig. 13:**
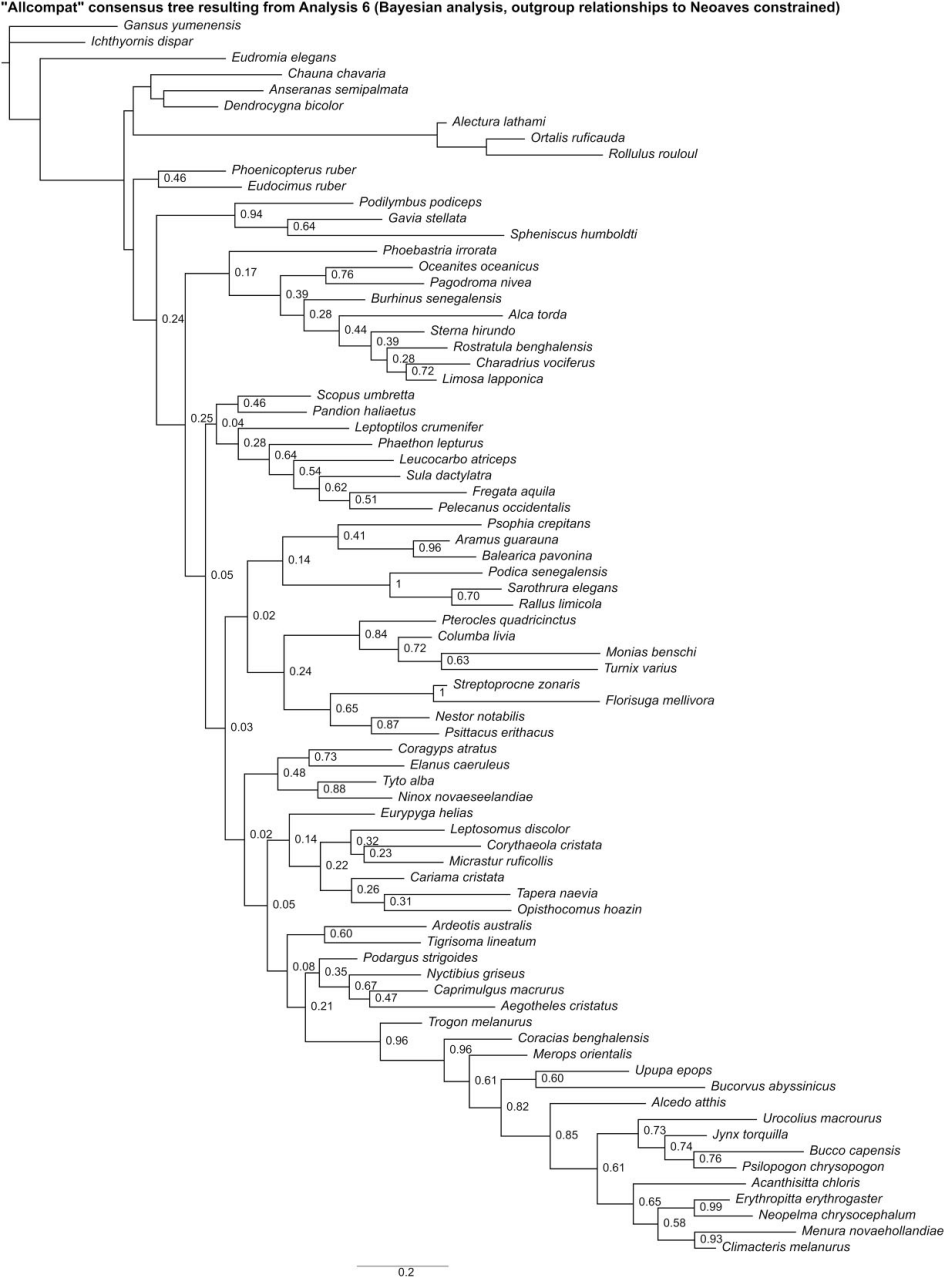
The “allcompat” consensus tree resulting from Analysis 6 (Bayesian analysis run with outgroup relationships to Neoaves constrained). Numbers on nodes represent posterior probabilities. Nodes lacking support values were constrained to be monophyletic.

In the “allcompat” consensus tree recovered by Analysis 6 (Fig. 13), a clade uniting *Phoenicopterus* and *Eudocimus* was placed as the sister group to all other neoavians, though posterior probability for this relationship was well below 50%. Twelve unconstrained nodes consistent with current phylogenetic consensus were resolved with posterior probabilities ≥50%: Apodiformes (*Streptoprocne* + *Florisuga*), Grues (*Aramus* + *Balearica*), Ralloidea (*Podica* + *Sarothrura* + *Rallus*), *Oceanites* + *Pagodroma*, Strigiformes (*Tyto* + *Ninox*), Bucerotiformes (*Upupa* + *Bucorvus*), Piciformes (*Bucco* + *Psilopogon* + *Jynx*), Psittaciformes (*Nestor* + *Psittacus*), Passeriformes (*Acanthisitta* + *Erythropitta* + *Neopelma* + *Menura* + *Climacteris*), Eupasseres (*Erythropitta* + *Neopelma* + *Menura* + *Climacteris*), Tyranni (*Erythropitta* + *Neopelma*), and Passeri (*Menura* + *Climacteris*). Other results that broadly align with those of recent phylogenetic studies include a clade uniting *Monias, Pterocles*, and *Columba* (though *Turnix* was also recovered as a member of this clade).

**Fig. 14:**
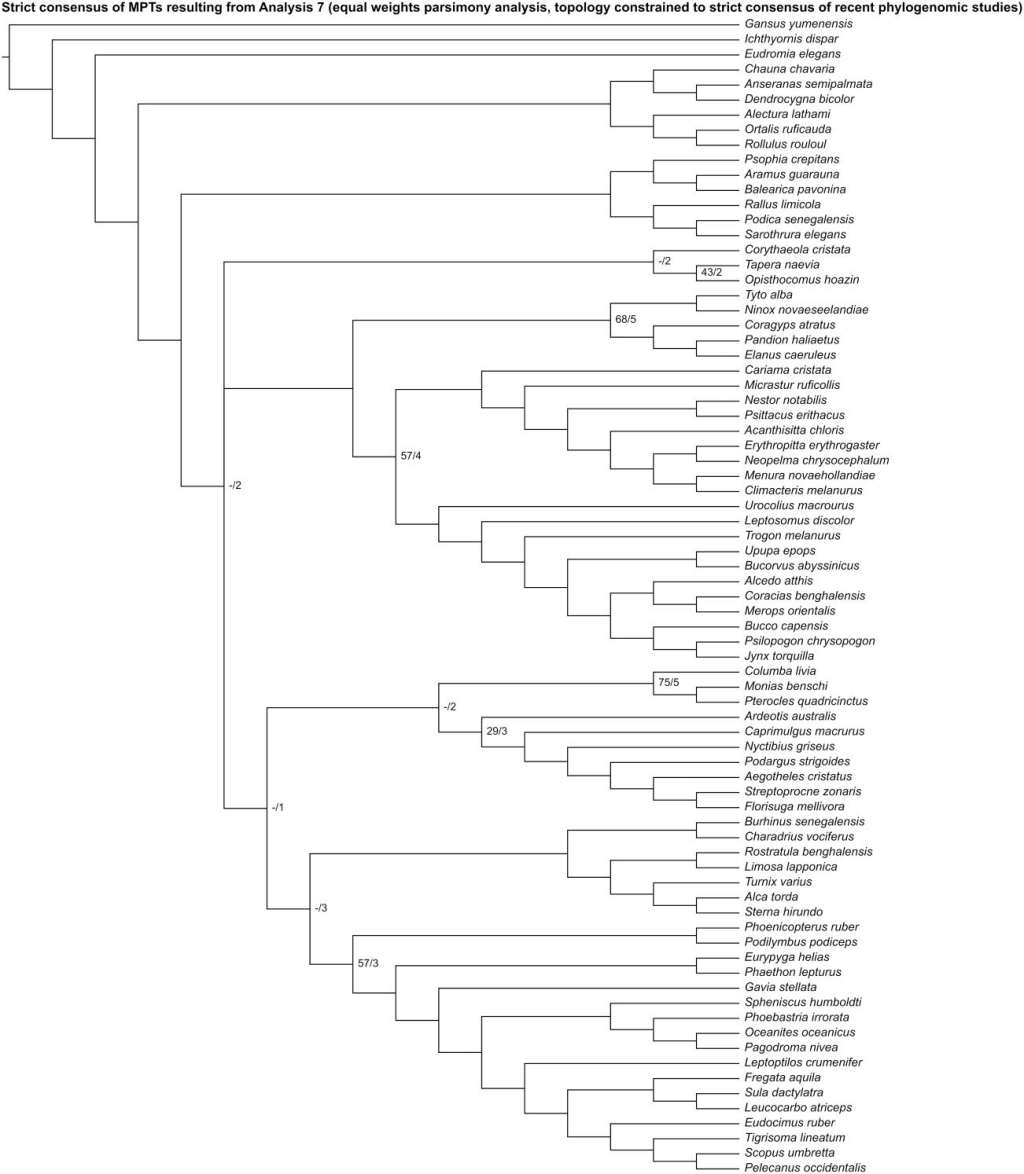
Strict consensus of most parsimonious trees resulting from Analysis 7 (equal weights parsimony analysis run with the topology constrained to a strict consensus of results from recent phylogenomic studies). Numbers on nodes represent absolute bootstrap frequencies (left of bar) and Bremer support values (right of bar). A hyphen (-) denotes values of 0. Nodes lacking support values were constrained to be monophyletic.

Analysis 7 recovered 2 MPTs of 2739 steps (Fig. 14), with a CI of 0.089 and a RI of 0.355. In the strict consensus of both MPTs, the interrelationships within Neoaves were poorly resolved, but Gruiformes was recovered as the sister group to other neoavians. The remaining neoavians were divided into 3 groups: Telluraves, a small clade consisting of *Corythaeola, Tapera*, and *Opisthocomus*, and a large clade consisting of Columbimorphae, *Ardeotis*, Strisores, Charadriiformes, Mirandornithes, and Phaethoquornithes. Within Telluraves, Accipitrimorphae was recovered as the sister group to Strigiformes, forming Hieraves; this clade was in turn recovered as the sister group to remaining telluravians.

**Fig. 15:**
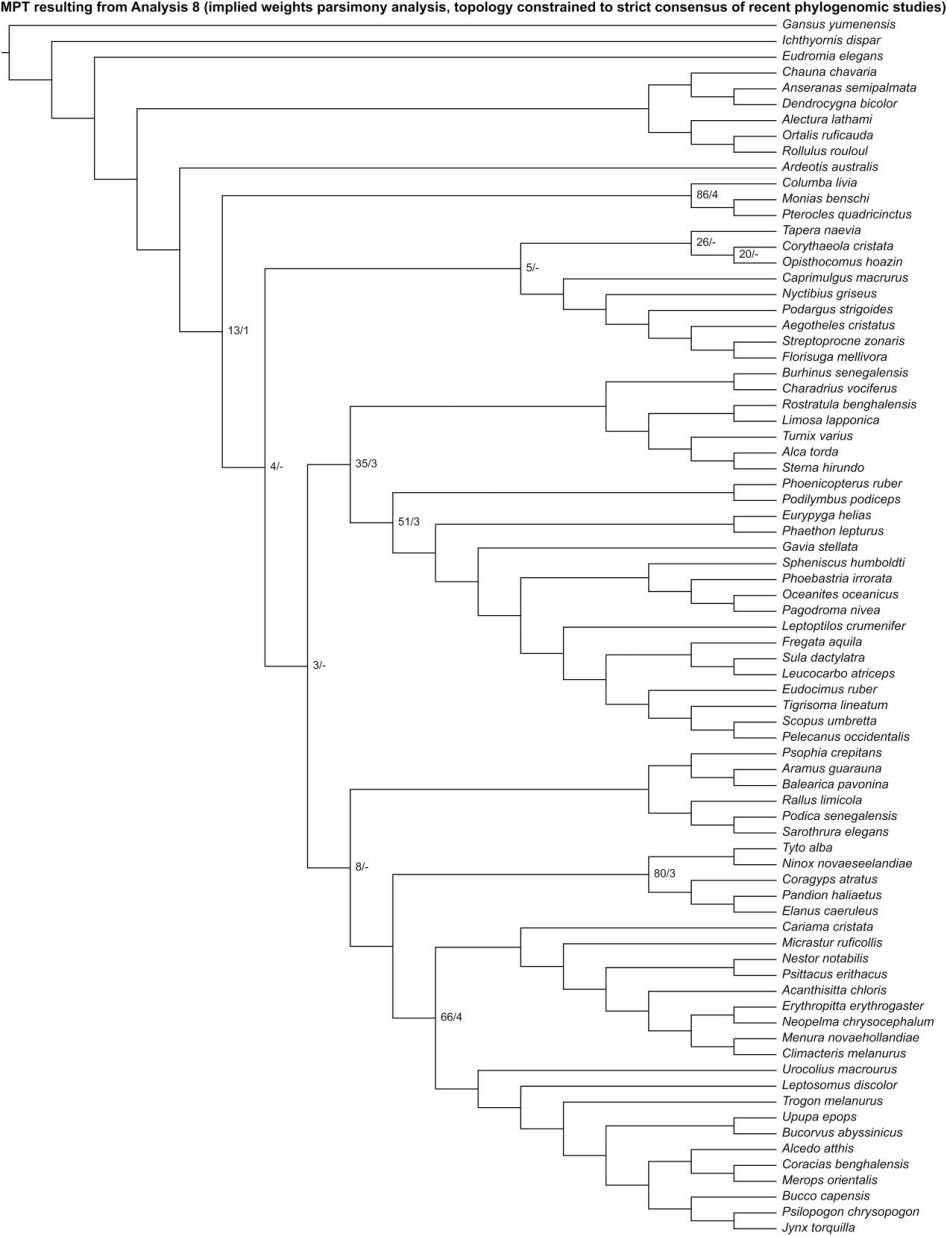
The most parsimonious tree resulting from Analysis 8 (implied weights parsimony analysis run with the topology constrained to a strict consensus of results from recent phylogenomic studies). Numbers on nodes represent absolute bootstrap frequencies (left of bar) and Bremer support values (right of bar). A hyphen (-) denotes values of 0. Nodes lacking support values were constrained to be monophyletic.

Analysis 8 recovered one MPT of 2744 steps (Fig. 15), with a CI of 0.089 and a RI of 0.354. *Ardeotis* was recovered as the sister group to all other neoavians. Columbimorphae, *Tapera* + *Corythaeola* + *Opisthocomus* + Strisores, Charadriiformes + Mirandornithes + Phaethoquornithes, and Gruiformes were recovered as successively closer relatives to Telluraves. Within Telluraves, Accipitrimorphae was recovered as the sister group to Strigiformes, forming Hieraves; this clade was in turn recovered as the sister group to remaining telluravians.

**Fig. 16:**
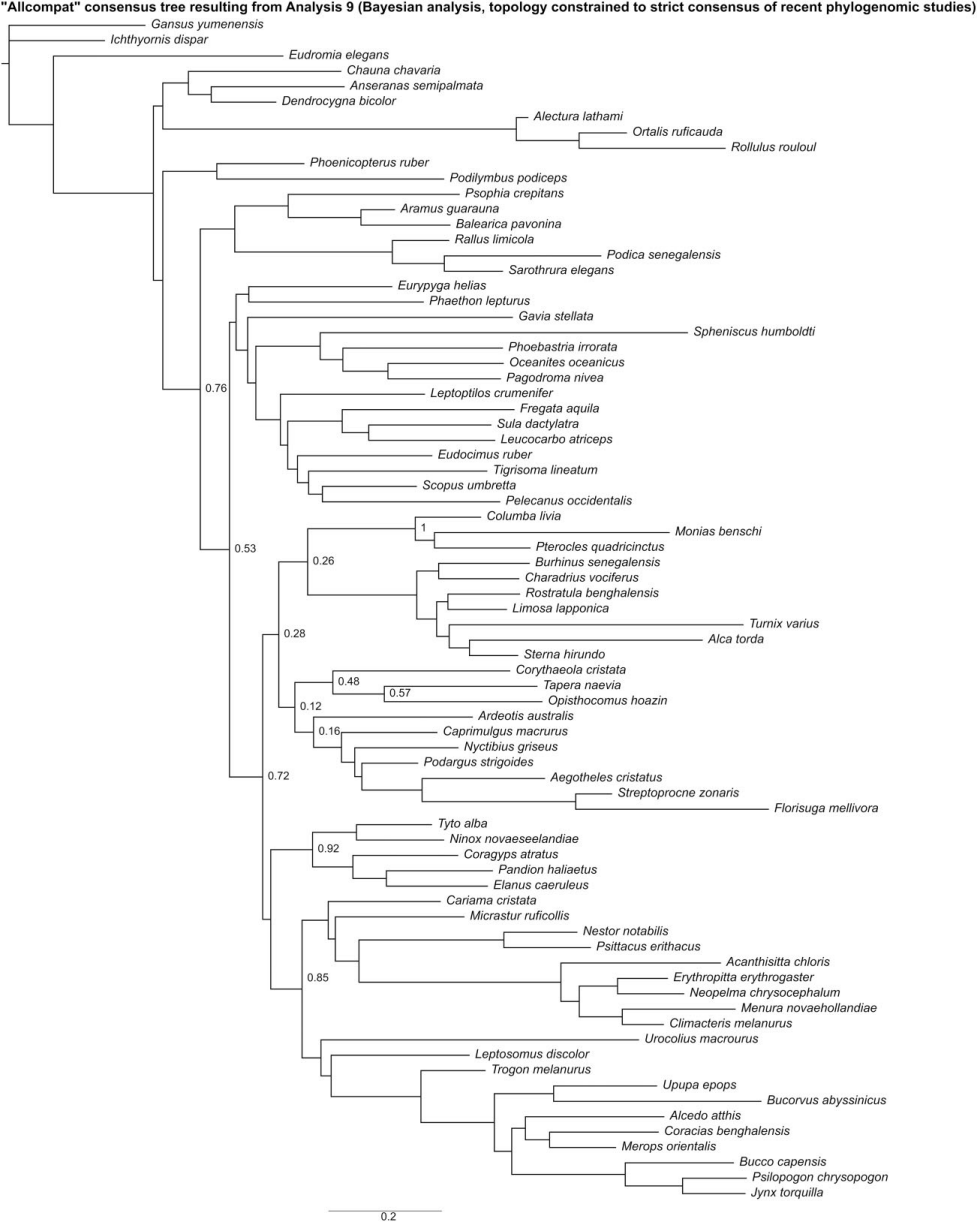
The “allcompat” consensus tree resulting from Analysis 9 (Bayesian analysis run with the topology constrained to a strict consensus of results from recent phylogenomic studies). Numbers on nodes represent posterior probabilities. Nodes lacking support values were constrained to be monophyletic.

In the “allcompat” consensus tree recovered by Analysis 9 (Fig. 16), Mirandornithes was placed as the sister group to all other neoavians. Gruiformes and Phaethoquornithes were recovered as successively closer to remaining neoavians with posterior probabilities ≥50%. Within Telluraves, Accipitrimorphae was recovered as the sister group to Strigiformes, forming Hieraves; this clade was in turn recovered as the sister group to remaining telluravians.

**Fig. 17:**
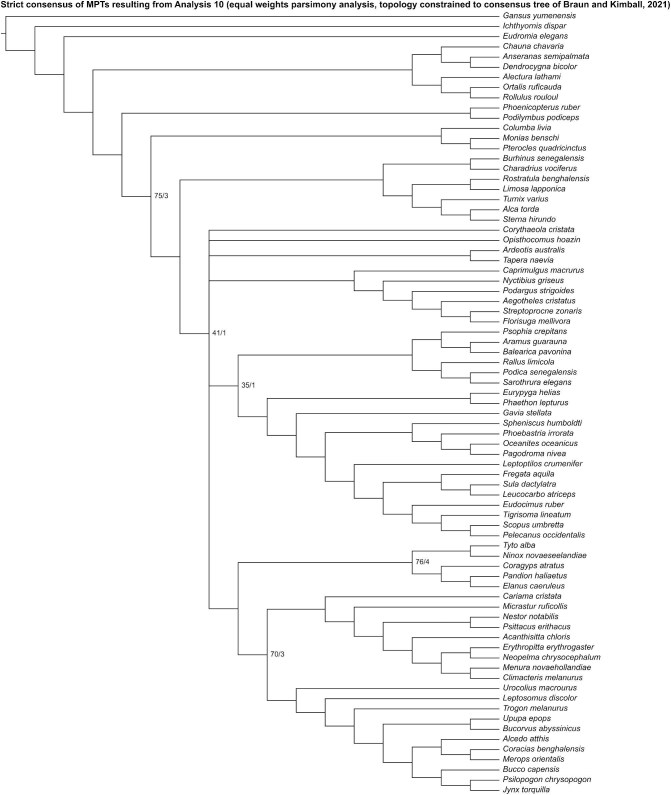
Strict consensus of most parsimonious tree resulting from Analysis 10 (equal weights parsimony analysis run with the topology constrained to the consensus tree proposed by Braun and Kimball 2021). Numbers on nodes represent absolute bootstrap frequencies (left of bar) and Bremer support values (right of bar). A hyphen (-) denotes values of 0. Nodes lacking support values were constrained to be monophyletic.

Analysis 10 recovered 2 MPTs of 2754 steps (Fig. 17), with a CI of 0.088 and a RI of 0.351. In the strict consensus of both MPTs, Mirandornithes was recovered as the sister group to the rest of Neoaves. Charadriiformes was recovered as the sister group to all other neoavians besides Mirandornithes and Columbimorphae, and Gruiformes was positioned as the sister group to Phaethoquornithes, but interrelationships among other major neoavian groups were otherwise poorly resolved. Within Telluraves, Strigiformes was recovered as the sister group to Accipitrimorphae, forming Hieraves, with both being excluded from a clade containing all other telluravians.

**Fig. 18:**
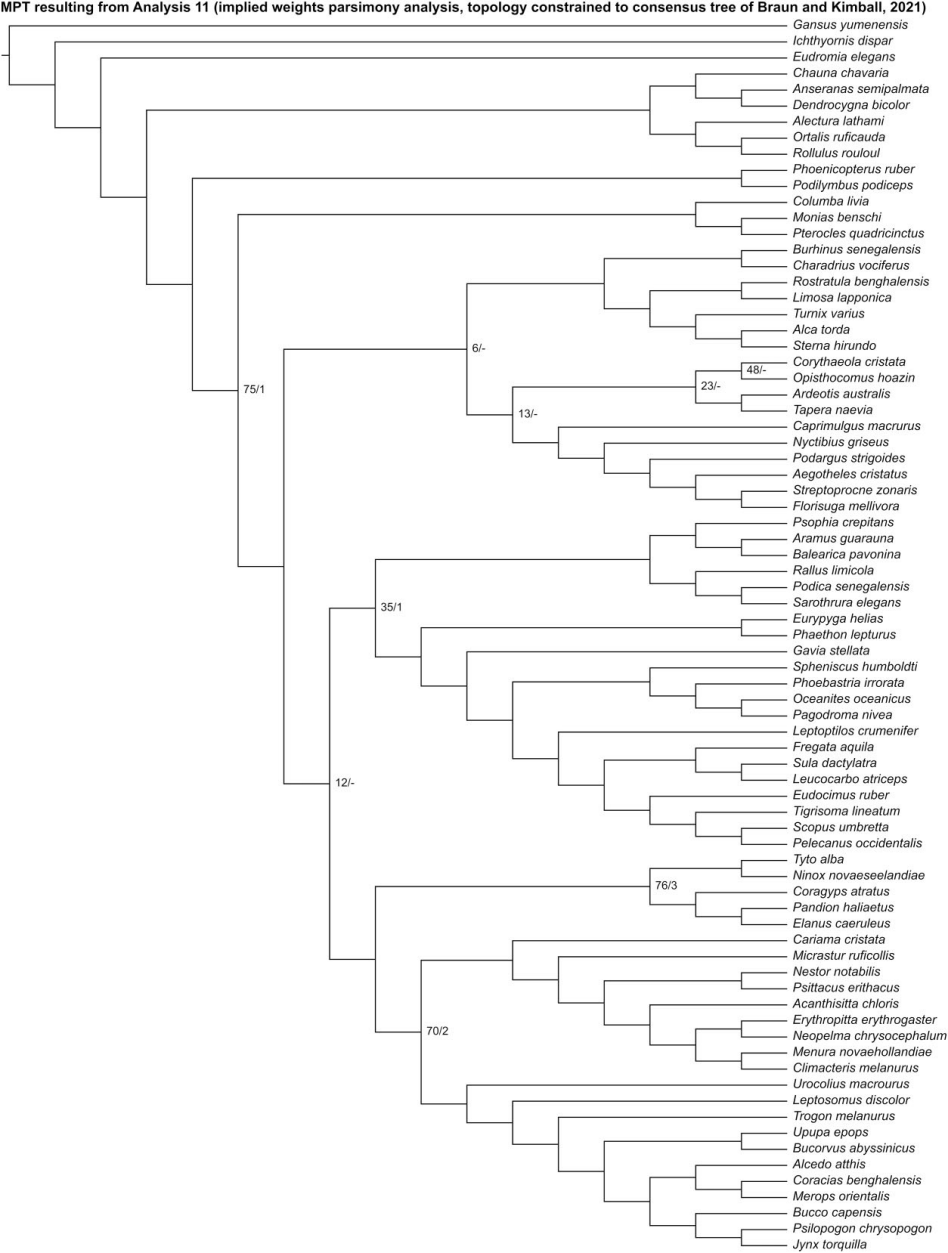
The most parsimonious tree resulting from Analysis 11 (implied weights parsimony analysis run with the topology constrained to the consensus tree proposed by Braun and Kimball 2021). Numbers on nodes represent absolute bootstrap frequencies (left of bar) and Bremer support values (right of bar). A hyphen (-) denotes values of 0. Nodes lacking support values were constrained to be monophyletic.

Analysis 11 recovered one MPT of 2755 steps (Fig. 18), with a CI of 0.088 and a RI of 0.351. Mirandornithes was recovered as the sister group to all other neoavians. Charadriiformes + *Ardeotis* + *Tapera* + *Corythaeola* + *Opisthocomus* + Strisores and Gruiformes + Phaethoquornithes were resolved as successively closer relatives to Telluraves. Within Telluraves, Strigiformes was recovered as the sister group to Accipitrimorphae, forming Hieraves, with both being excluded from a clade containing all other telluravians.

**Fig. 19:**
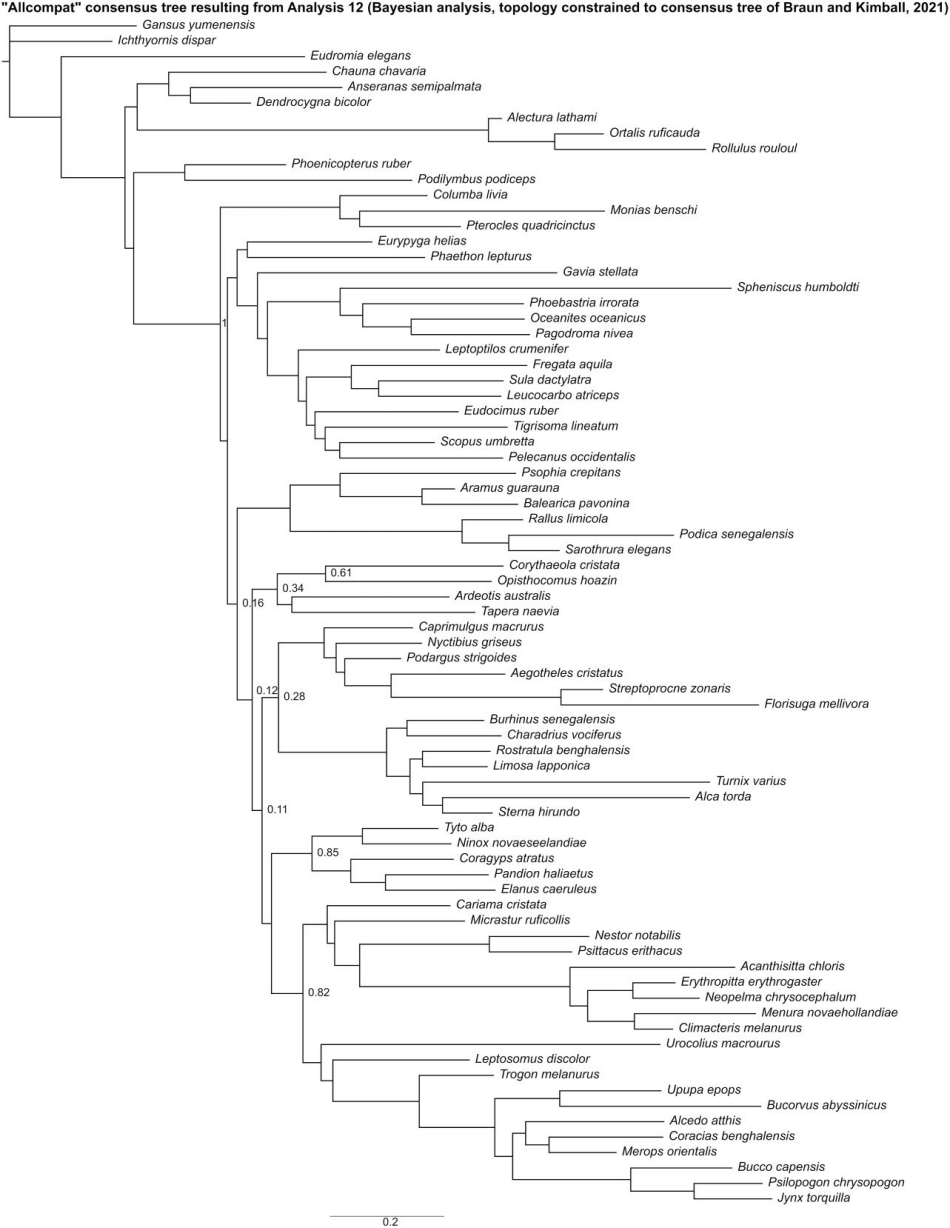
The “allcompat” consensus tree resulting from Analysis 12 (Bayesian analysis run with the topology constrained to the consensus tree proposed by Braun and Kimball 2021). Numbers on nodes represent posterior probabilities. Nodes lacking support values were constrained to be monophyletic.

In the “allcompat” consensus tree recovered by Analysis 12 (Fig. 19), Mirandornithes was placed as the sister group to all other neoavians with high posterior probability (= 1), but posterior probabilities for interrelationships inferred among other major neoavian groups were generally low. Within Telluraves, Strigiformes was recovered as the sister group to Accipitrimorphae, forming Hieraves, with both being excluded from a clade containing all other telluravians.

### Comparisons with molecular topologies

Values for tree distance metrics comparing the most parsimonious trees and “allcompat” consensus trees recovered by the morphological analyses in the present study to topologies recovered by recent avian phylogenomic studies are provided in Table 2. In general, analyses constrained to the consensus tree of Braun and Kimball (2021) (Analyses 10–12) produced trees with the shortest distances from molecular topologies. Under analyses where no topological constraints were applied (Analyses 1–3), results of Bayesian inference exhibited shorter average distances from molecular topologies than those of parsimony analyses. Under analyses where only outgroup relationships were constrained (Analyses 4–6), the results of both implied weights parsimony and Bayesian inference exhibited shorter distances than equal weights parsimony analysis. However, in analyses implementing more extensive topological constraints, implied weights parsimony and Bayesian analyses often yielded values for RF distances comparable to or exceeding those based on equivalent equal weights parsimony analyses.

**Table 2:** Values for RF distances comparing the most parsimonious trees and “allcompat” consensus trees recovered by the morphological analyses in the present study to topologies recovered by recent avian phylogenomic studies

	Jarvis et al. (2014)	Prum et al. (2015)	Kuhl et al. (2021)	Stiller et al. (2024)	Wu et al. (2024b)
T1: Unconstrained topology analyzed under equal weights parsimony	118–120 (mean = 118.7)	118–120 (mean = 118.7)	118–120 (mean = 118.7)	118–120 (mean = 118.7)	118–120 (mean = 118.7)
T2: Unconstrained topology analyzed under implied weights parsimony	122	122	122	122	122
T3: Unconstrained topology analyzed under Bayesian inference	118	118	118	118	118
T4: Outgroups constrained and analyzed under equal weights parsimony	106–108 (mean = 107.2)	106–108 (mean = 107.2)	106–108 (mean = 107.2)	106–108 (mean = 107.2)	106–108 (mean = 107.2)
T5: Outgroups constrained and analyzed under implied weights parsimony	104	104	104	104	104
T6: Outgroups constrained and analyzed under Bayesian inference	104	104	104	104	104
T7: Constrained to strict consensus of all reference trees and analyzed under equal weights parsimony	22 (mean = 22)	20 (mean = 20)	24 (mean = 24)	20 (mean = 20)	20 (mean = 20)
T8: Constrained to strict consensus of all reference trees and analyzed under implied weights parsimony	22	20	24	20	20
T9: Constrained to strict consensus of all reference trees and analyzed under Bayesian inference	22	22	22	18	20
T10: Constrained to consensus tree of Braun and Kimball and analyzed under equal weights parsimony	20 (mean = 20)	20 (mean = 20)	22 (mean = 22)	16 (mean = 16)	20 (mean = 20)
T11: Constrained to consensus tree of Braun and Kimball and analyzed under implied weights parsimony	20	20	22	16	20
T12: Constrained to consensus tree of Braun and Kimball and analyzed under Bayesian inference	20	20	22	16	20

The molecular topologies studied did not differ from each other substantially in distance from each morphological tree recovered, but on average the Stiller et al. (2024) topology exhibited slightly shorter RF distances from morphological trees than the others when results were constrained to consensus trees of molecular studies, whereas the Kuhl et al. (2021) topology exhibited slightly higher RF distances.

When topologies recovered by recent phylogenomic analyses were enforced under the current dataset, the Wu et al. (2024b) topology resulted in the longest tree and the lowest RI. The Jarvis et al. (2014), Kuhl et al. (2021), and Wu et al. (2024b) topologies were tied for the lowest CI and highest RHI. The Prum et al. (2015) and Stiller et al. (2024) topologies resulted in the highest RI and CI, with the Stiller et al. (2024) topology corresponding to the shortest tree and the Prum et al. (2015) topology exhibiting the lowest RHI. Specific values for these metrics are reported in Table 3.

**Table 3:** Characterization of trees resulting from enforcing the results of recent avian phylogenomic studies on the current morphological dataset with ordered characters included

	Jarvis et al. (2014)	Prum et al. (2015)	Kuhl et al. (2021)	Stiller et al. (2024)	Wu et al. (2024b)
Tree length	2780 steps	2771 steps	2778 steps	2769 steps	2781 steps
CI	0.087	0.088	0.087	0.088	0.087
RI	0.345	0.347	0.345	0.347	0.344
RHI	0.795	0.791	0.795	0.792	0.795

**Fig. 20:**
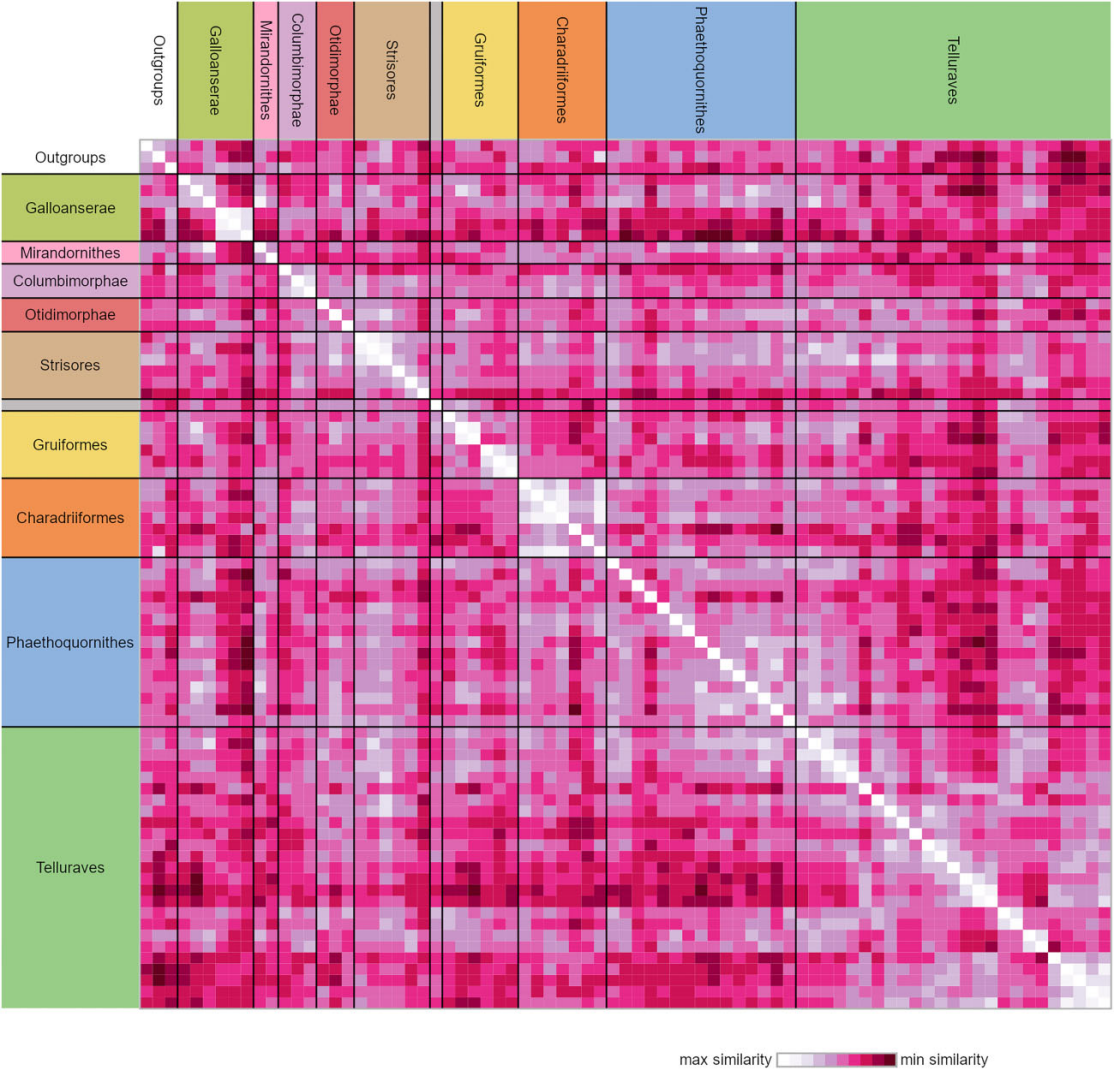
Heatmap of symmetric pairwise-taxon similarity matrix using our complete character dataset (*n* = 203). The unlabeled monotypic group positioned between Strisores and Gruiformes represents *Opisthocomus*.

The heatmap generated from the similarity matrix incorporating all characters in the present study suggests that our morphological dataset contains a relatively low phylogenetic signal (Fig. 20). Although relatively high intra-group similarity is seen in some clades (e.g., Anseriformes, Galliformes, Columbimorphae, some Strisores, Grues, Ralloidea, most Charadriiformes, Accipitrimorphae, Strigiformes, Piciformes, Psittaciformes, and Passeriformes), many species exhibit comparable or greater similarity to distantly related taxa in comparison with their closest relatives included in the dataset.

**Fig. 21:**
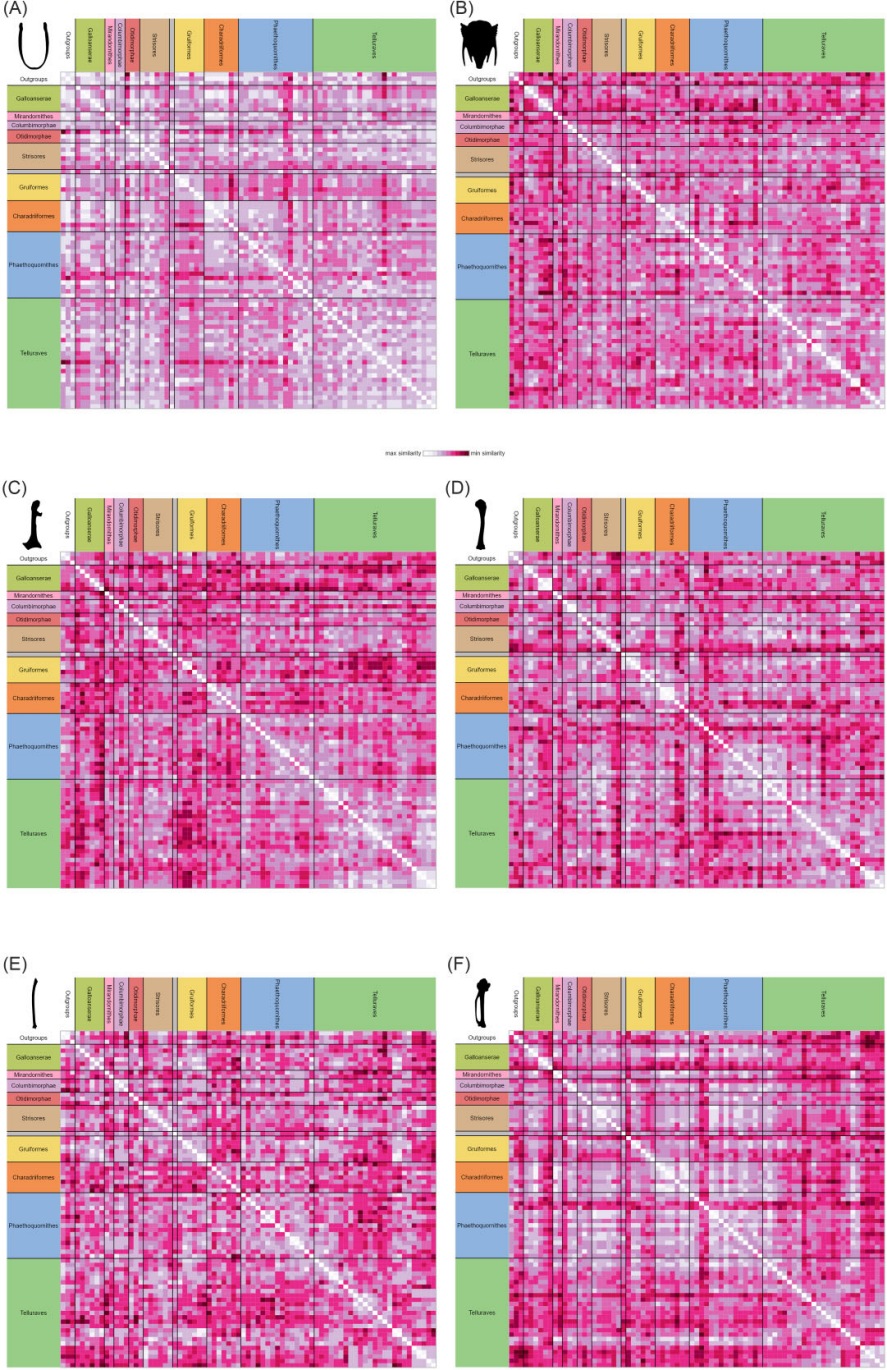
Heatmaps of symmetric pairwise-taxon similarity matrices of subsets of our character dataset partitioned by individual element, including the furcula (**A**, *n* = 13), sternum (**B**, *n* = 49), coracoid (**C**, *n* = 26), humerus (**D**, *n* = 45), ulna (**E**, *n* = 14), and carpometacarpus (**F**, *n* = 30). The unlabeled monotypic group positioned between Strisores and Gruiformes represents *Opisthocomus*.

This general pattern is also seen in heatmaps generated from subsets of our character dataset partitioned by individual skeletal elements (Fig. 21). Of note, however, is that characters from the humerus and carpometacarpus appear to more closely reflect phylogenetic relationships compared to those of the other elements analyzed.

### Homoplasy across avian morphological matrices

**Fig. 22:**
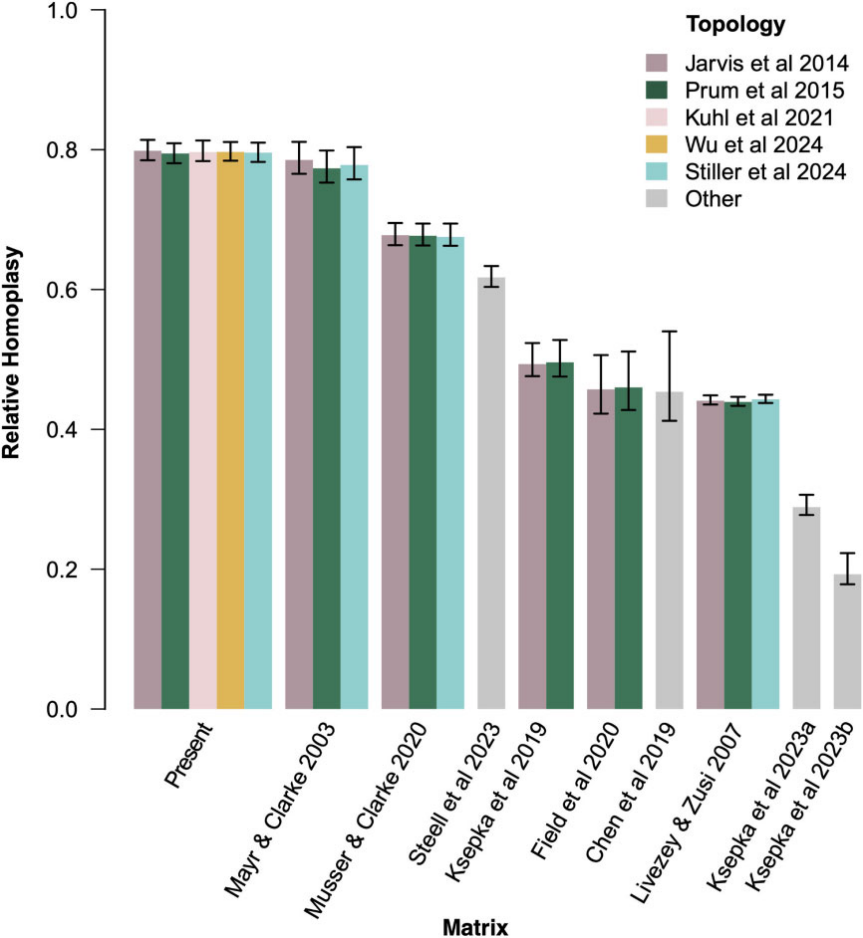
Bar plot showing relative homoplasy index values for the present matrix and published matrices of extant birds for a range of molecular topologies. Error bars indicate the 5% and 95% quantile ranges. A value of 1 indicates maximum possible homoplasy. Where topology is denoted as “other,” details of molecular reference trees are indicated in the main text and Table 4.

Our comparative homoplasy analysis shows that the present matrix is highly homoplastic with an RHI value of 0.79–0.80 when all characters are treated as unordered (Fig. 22, Table 4). A value of zero or close to zero would indicate no or little homoplasy within the dataset, and a value of one or close to one would indicate high levels of homoplasy comparable to a matrix with no phylogenetic signal present with regards to the empirical tree. Indeed, when states within the empirical pectoral matrix are permuted without replacement within characters (therefore representing a matrix with no phylogenetic signal with respect to any external tree), RHI values range between 0.99 and 1.00 for the published topological constraints. We find no significant difference in values across different topological constraints per matrix with near-identical RHI values and overlapping confidence intervals for all datasets sampled (Table 4). The Mayr and Clarke (2003) crown bird dataset exhibits comparable levels of homoplasy to the present matrix (Fig. 22, Table 4), and together both matrices are the most homoplastic out of the datasets sampled. The Ksepka et al. (2023b) sphenisciform dataset shows the lowest relative homoplasy with an RHI of 0.19.

**Table 4:** Summary of the relative homoplasy results for published extant avian datasets in addition to consistency and retention indices.

Study	Taxa	No. taxa	No. Characters	Constraint	Tree length	RHI	RHI 5%	RHI 95%	CI	RI
Present	Neornithes	75	202	Jarvis et al. 2014	2736	0.80	0.78	0.81	0.09	0.34
				Prum et al. 2015	2727	0.79	0.78	0.81	0.09	0.34
				Kuhl et al. 2021	2733	0.80	0.78	0.81	0.09	0.34
				Wu et al. 2024b	2738	0.80	0.78	0.81	0.09	0.34
				Stiller et al. 2024	2727	0.79	0.78	0.81	0.09	0.34
Mayr and Clarke 2003	Neornithes	43	145	Jarvis et al. 2014	824	0.79	0.76	0.81	0.20	0.33
				Prum et al. 2015	813	0.77	0.75	0.80	0.20	0.35
				Stiller et al. 2024	817	0.78	0.76	0.80	0.20	0.34
Livezey and Zusi 2007	Neornithes	150	2295	Jarvis et al. 2014	17967	0.44	0.44	0.45	0.19	0.61
				Prum et al. 2015	17929	0.44	0.43	0.45	0.19	0.61
				Stiller et al. 2024	18046	0.44	0.44	0.45	0.19	0.61
Field et al. 2020	Neornithes	26	293	Jarvis et al. 2014	1094	0.45	0.42	0.50	0.36	0.63
				Prum et al. 2015	1100	0.46	0.43	0.51	0.36	0.63
Musser and Clarke 2020	Neornithes	52	692	Jarvis et al. 2014	5247	0.68	0.66	0.70	0.16	0.45
				Prum et al. 2015	5246	0.68	0.66	0.70	0.16	0.45
				Stiller et al. 2024	5237	0.68	0.66	0.69	0.16	0.45
Chen et al. 2019	Strisores	28	114	Chen et al. 2019	233	0.46	0.41	0.55	0.54	0.63
Ksepka et al. 2019	Telluraves	34	144	Jarvis et al. 2014	548	0.49	0.47	0.53	0.33	0.57
				Prum et al. 2015	549	0.50	0.48	0.53	0.33	0.57
Ksepka et al. 2023a	Galliformes	61	135	Kimball et al. 2021	442	0.29	0.28	0.31	0.34	0.75
Ksepka et al. 2023b	Sphenisciformes	25	245	Pan et al. 2019 and Vianna et al. 2020	422	0.19	0.18	0.22	0.76	0.83
Steell et al. 2023	Passeriformes	142	49	Steell et al. 2023	990	0.62	0.63	0.60	0.06	0.51

Note that RHI measures homoplasy so that zero indicates no homoplasy and one indicates maximum possible homoplasy, whereas CI and RI indicate the opposite.

### Optimization of synapomorphies onto molecular topologies

Potential synapomorphies from the pectoral and forelimb skeleton were recovered for most avian clades strongly supported by molecular phylogenetic analyses, though many of these characters appear to be homoplastic across crown bird phylogeny and some were not inferred to be synapomorphic under all alternative molecular topologies. Specific characters optimized as synapomorphies for major clades and their phylogenetic distributions are detailed below and in Supplementary Material S8.

## Discussion

### Distribution of optimized osteological synapomorphies for well-supported clades

Although potential morphological synapomorphies could be optimized for most of the avian clades recognized by molecular phylogenetic analyses, not all of these character states are equally convincing as synapomorphies when their distribution among crown birds and relevant fossil taxa is considered. Here, remarks on character state distributions in fossil taxa are limited to characters that can be assessed based on previously published fossil descriptions. Synapomorphies optimizing onto specific molecular phylogenetic topologies are denoted below as J14 (for Jarvis et al. 2014), P15 (for Prum et al. 2015), K21 (for Kuhl et al. 2021), S24 (for Stiller et al. 2024), and W24 (for Wu et al. 2024b).

#### Neornithes

Eleven character states were optimized as potential synapomorphies of Neornithes (the avian crown group), of which eight were consistently inferred as such across all alternative molecular topologies studied.

- Four articular facets on costal margin of sternum or fewer (char. 36: 2 > 1, reversed in Anseriformes, Mirandornithes, *Monias, Corythaeola, Podargus*, Apodiformes, *Opisthocomus*, Gruiformes, Charadriiformes, Phaethoquornithes, Accipitrimorphae, and Australaves); P15.
- Ventromedial intermuscular line on sternum prominent (char. 39: 0 > 1, reversed in *Chauna, Corythaeola, Ardeotis, Tapera*, Strisores, *Opisthocomus, Rallus, Turnix*, Phaethontimorphae, *Gavia, Pelecanus, Elanus, Ninox*, Coraciimorphae, and Passeriformes); J14, P15, K21, S24, W24.
- Single pair of caudal fenestrae in sternum (char. 51: 2 > 1, reversed in *Pterocles, Columba, Corythaeola, Ardeotis, Tapera*, Vanescaves, *Opisthocomus*, Charadriiformes, Phaethontimorphae, *Pagodroma, Eudocimus, Coragyps, Ninox*, Coraciimorphae, and *Acanthisitta*); K21, S24.
- Median trabecula of sternum extends caudal to caudolateral trabeculae (char. 62: 1 > 0, reversed in *Dendrocygna*, Mirandornithes, Strisores, Heliornithes, *Phaethon, Spheniscus, Phoebastria*, Suliformes, *Scopus, Pelecanus, Pandion* + *Elanus, Tyto*, Pici, *Micrastur*, and *Nestor*); J14, P15, K21, S24, W24.
- Scapular cotyle on coracoid a shallow concavity (char. 68: 0 > 1, reversed in *Chauna, Dendrocygna, Phoenicopterus, Pterocles, Ardeotis*, Apodiformes, *Opisthocomus*, Charadriiformes, *Gavia, Leptoptilos, Pelecanus*, and *Tigrisoma*); J14, P15, K21, S24, W24.
- Impression of m. sternocoracoidei on coracoid shallow or hard to discern (char. 78: 1 > 0, reversed in *Chauna, Phoenicopterus, Aegotheles, Opisthocomus*, Grues, *Sarothrura, Rallus, Charadrius, Turnix, Sterna, Spheniscus, Phoebastria, Coragyps, Coracias*, and *Cariama*); J14, P15, K21, S24, W24.
- Lateral process of coracoid unhooked or weakly hooked (char. 82: 1 > 0, reversed in *Anseranas, Rollulus, Ardeotis, Tapera*, Strisores, Grues, *Burhinus, Sterna, Phaethon*, Procellariiformes, *Leptoptilos, Fregata, Leucocarbo, Scopus* + *Pelecanus, Pandion, Trogon, Bucrovus, Coracias, Bucco*, and *Psittacus*); J14, P15, K21, S24, W24.
- Pneumatic foramen in humeral pneumotricipital fossa (char. 109: 0 > 1, reversed in *Podilymbus*, Ralloidea, Charadriiformes, *Gavia, Spheniscus, Oceanites, Pagodroma, Leucocarbo, Urocolius*, and *Acanthisitta*); J14, P15, K21, S24, W24.
- Deltopectoral crest a small eminence, extending less than one third the total length of the humerus (char. 122: 0 > 1, reversed in *Corythaeola, Ardeotis, Nyctibius, Podargus, Spheniscus, Leptoptilos, Leucocarbo, Eudocimus, Scopus*, Accipitrimorphae, Strigiformes, Cavitaves, *Micrastur*, and Passeri); J14, P15, K21, S24, W24.
- Radial depression on ulna weak (char. 161: 1 > 0, reversed in *Chauna, Dendrocygna, Podilymbus, Corythaeola, Streptoprocne, Charadrius, Limosa, Alca* + *Sterna, Phaethon, Leptoptilos, Fregata, Pelecanus, Pandion* + *Elanus, Leptosomus, Bucorvus*, Picodynastornithes, *Psittacus*, and *Climacteris*); J14, P15, K21, S24, W24.
- Minor metacarpal strongly bowed (char. 173: 0 > 1, reversed in *Dendrocygna*, Mirandornithes, Apodiformes, Charadriiformes, *Phaethon, Gavia*, Procellariimorphae, *Fregata, Eudocimus, Pandion*, Picodynastornithes, and Psittacopasseres); J14, P15, S24, W24.

However, many of these features are absent in the extinct Lithornithidae, which have been recovered as stem-paleognaths (Nesbitt and Clarke 2016; Worthy et al. 2017) or near-crown stem-birds (Livezey and Zusi 2007; Hartman et al. 2019) in recent phylogenetic analyses. Based on previous descriptions (Houde 1988; Bourdon and Lindow 2015; Nesbitt and Clarke 2016; Mayr and Kitchener 2025a), lithornithids typically lack prominent intermuscular lines on the sternum (char. 39: 0), sternal incisures (char. 51: 0) and have a deep, cup-like scapular cotyle of the coracoid (char. 68: 0), a deep impression of the m. sternocoracoidei on the coracoid (char. 78: 1), a sharply hooked lateral process of the coracoid (char. 82: 1), a well-developed deltopectoral crest (char. 122: 0), and a weakly bowed minor metacarpal (char. 173: 0). This distribution of character states might reflect a position of lithornithids outside of crown-group birds, but it is also plausible that character optimization at this node may be confounded by convergent acquisition of character states in tinamous (Tinamidae) and galliforms, perhaps correlated with specialization in these groups for burst flight (Houde 1988; Widrig et al. 2023). Based on comparisons with fossil crown birds and near-crown stem-birds, Mayr (2021a) previously inferred that a deep, cup-like scapular cotyle is likely plesiomorphic for crown birds, despite the presence of a shallow or flattened facet in extant tinamids and galliforms. Of the above characters, lithornithids do exhibit a pneumatic foramen in the pneumotricipital fossa of the humerus.

Other pectoral and forelimb features that have been considered potential synapomorphies of crown birds include pneumatic pores in the intercostal incisures of the sternum and a pneumatized coracoid (Clarke 2004; Turner et al. 2012; Benito et al. 2022b). However, minute and possibly pneumatic pores have subsequently been identified in the intercostal incisures of *Ichthyornis* (Benito et al. 2022a), and as a result, this character state was not optimized as a synapomorphy of Neornithes in our analyses. Coracoid pneumatization was not coded as a single character in the present study, as the positioning of pneumatic foramina in the coracoid differs among crown birds with pneumatic coracoids. In lithornithids (Houde 1988) and tinamids (Bertelli et al. 2014; Suzuki et al. 2014; Widrig et al. 2023) the coracoid is dorsally penetrated by one or more pneumatic foramina near the scapular cotyle, whereas in many galloanserans and some neoavians the pneumatic foramen is positioned near the sternal facet.

#### Neognathae

Seven character states were optimized as potential synapomorphies of Neognathae, of which four were consistently inferred as such across all alternative molecular topologies studied.

- Dorsolateral intermuscular line on sternum prominent (char. 38: 0 > 1, reversed in *Corythaeola, Tapera, Caprimulgus, Nyctibius, Aegotheles, Sarothrura, Rallus, Phoebastria, Fregata, Sula, Pelecanus*, and *Bucorvus*); J14, K21, S24, W24.
- Median trabecula of sternum squared off at caudal termination (char. 61: 0 > 1, reversed in *Ortalis* + *Rollulus, Monias, Columba, Florisuga*, Gruiformes, *Turnix, Alca, Eurypyga*, Aequornithes, Accipitrimorphae, *Tyto, Urocolius, Bucorvus, Bucco*, Psittaciformes, and *Menura)*; J14, P15, K21, S24, W24.
- Length of coracoid, between three and four times the width of articular facet for sternum (char. 74: 1 > 2, reversed in *Anseranas, Phoenicopterus, Corythaeola, Nyctibius*, Grues, Charadrii, *Limosa, Phaethon, Gavia*, Procellariiformes, *Fregata, Eudocimus*, and Accipitriformes); J14, P15, K21, S24, W24.
- Olecranon fossa on humerus deep (char. 127: 0 > 1, reversed in *Anseranas*, Mirandornithes, *Monias, Ardeotis, Nyctibius, Opisthocomus*, Grues, *Alca, Gavia, Leptoptilos*, and *Eudocimus*); J14, P15, W24.
- Tendinal sulcus of m. scapulotricipitalis on humerus present (char. 128: 0 > 1, reversed in *Nyctibius, Podargus, Phoebastria, Pagodroma*, and *Menura*); J14, P15, K21, S24, W24.
- Proximal third of minor metacarpal more than half as thick of major metacarpal dorsoventrally (char. 178: 0 > 1, reversed in *Anseranas, Ardeotis, Nyctibius, Podargus*, Apodiformes, *Sula*, and *Pelecanus*); J14, P15, K21, S24, W24.
- Extensor process on carpometacarpus surpasses distal articular facet for alular digit by the width of the facet or more (char. 181: 1 > 2, reversed in Mirandornithes, *Monias, Corythaeola, Opisthocomus, Charadrius, Phaethon, Gavia, Spheniscus*, Bucerotiformes, *Nestor*, and *Climacteris*); J14, P15, W24.

In lithornithids, the extensor process on the carpometacarpus surpasses the distal articular facet for the alular digit by approximately the width of the facet (Nesbitt and Clarke 2016), so this character may be synapomorphic for a more inclusive clade than Neognathae.

Although a moderately elongate coracoid is found in stem-galliforms (Mourer-Chauviré 1992a, 2000; Mayr and Weidig 2004; Mayr and Kitchener 2023a) and the putative stem-anseriform (Houde et al. 2023) or stem-galliform (Mayr et al. 2023a) *Danielsavis*, stockier coracoids (in which the total length of the bone is only two to three times the width of the sternal facet) are present in *Anachronornis* (Houde et al. 2023), *Nettapterornis* (Olson 1999), and Presbyornithidae (Howard 1955; Ericson 1999; Worthy et al. 2023), which are potential stem-anseriforms (Tambussi et al. 2019; Field et al. 2020; Houde et al. 2023; Crane et al. 2025). A similarly stocky coracoid is found in *Juncitarsus* (Ericson 1999), a putative stem member of Mirandornithes (Mayr 2014b), which is a clade that has been recovered as the sister group to all other neoavians in some molecular analyses (Braun and Kimball 2021; Kuhl et al. 2021; Stiller et al. 2024). Therefore, a more elongate coracoid may have been convergently acquired by several neognath clades.

The presence of a tendinal sulcus on the humerus for the m. scapulotricipitalis as a synapomorphy of Neognathae is consistent with previously described fossil evidence, as this feature is absent in most near-crown stem-birds (Clarke 2004; Clarke et al. 2006; Wang et al. 2016) and lithornithids (Nesbitt and Clarke 2016), whereas it is present (though inconspicuous) in *Anachronornis* (Houde et al. 2023), presbyornithids (Ericson 2000; Worthy et al. 2023), and the stem-galliform *Paraortygoides messelensis* (Mayr 2000a). However, this sulcus is lacking in *Danielsavis* (Houde et al. 2023). A deep olecranon fossa is similarly absent in near-crown stem-birds (Clarke 2004; Wang et al. 2016) but present in *Anachronornis* (Houde et al. 2023), presbyornithids (Worthy et al. 2023), and the possible stem-anseriform *Conflicto* (Tambussi et al. 2019). This character is absent in Mirandornithes, however, and was therefore not optimized as a neognath synapomorphy under the topologies of Kuhl et al. (2021) and Stiller et al. (2024).

#### Galloanserae

One character state was optimized as a potential synapomorphy of Galloanserae, which was consistently inferred as such across all alternative molecular topologies studied.

- Large pneumatic foramen in impression of m. sternocoracoidei in coracoid (char. 79: 0 > 1, reversed in *Dendrocygna*; also found in *Pterocles, Opisthocomus, Psophia, Balearica*, and *Phoebastria*); J14, P15, K21, S24, W24.

Previously reported morphological synapomorphies of Galloanserae have been largely limited to features of the skull (Cracraft and Clarke 2001; Mayr 2011a; Field et al. 2020; Crane et al. 2025), so the identification of a potential pectoral synapomorphy is noteworthy. A large pneumatic foramen in the impression of the m. sternocoracoidei is present in non-anatid crown anseriforms, many crown galliforms, *Nettapterornis* (Olson 1999), and the stem-galliform *Ameripodius* (Mourer-Chauviré 2000), but is absent in *Anachronornis, Danielsavis* (Houde et al. 2023), presbyornithids (Howard 1955; Worthy et al. 2023), and the stem-galliforms *Gallinuloides* (Mayr and Weidig 2004), *Paraortygoides* (Mayr 2006a; Mayr and Kitchener 2023a), *Waltonortyx* (Mayr and Kitchener 2023a), *Paraortyx*, and *Quercymegapodius* (Mourer-Chauviré 1992a). Whether this character was present in the last common ancestor of Galloanserae is therefore uncertain. However, an evolutionary tendency toward the formation of a pneumatic foramen near the sternal end of the coracoid may plausibly be an underlying synapomorphy of Galloanserae, as suggested by Mayr (2006a).

The presence of several prominent muscle scars that diagonally traverse the dorsal surface of the coracoid may be a synapomorphy of Galloanserae, as this feature is found in most crown anseriforms, presbyornithids (Ericson 1997), *Danielsavis* (Houde et al. 2023), *Gallinuloides* (Mayr and Weidig 2004), *Paraortygoides* (Mayr 2006a), *Waltonortyx* (Mayr and Kitchener 2023a), and *Ameripodius* (Mourer-Chauviré 2000). Although this character was included in the present study (char. 76: 1), the extant-only taxon sampling for crown birds prevented it from being optimized as a galloanseran synapomorphy, as it is absent in most crown galliforms.

#### Neoaves

Six character states were optimized as potential synapomorphies of Neoaves, but none of these were consistently inferred as such across all alternative molecular topologies studied. No unambiguous synapomorphies for this clade were inferred under the Stiller et al. (2024) topology.

- Ventrolateral sulcus on sternum distinct (char. 34: 0 > 1, reversed in Mirandornithes, *Caprimulgus, Aegotheles, Sterna*, Gruoidea, *Eurypyga, Phoebastria, Leptoptilos*, Pelecaniformes, *Bucorvus*, and *Nestor*; also found in *Rollulus*); W24.
- Coracoid keeled at ventromedial margin of supracoracoid sulcus (char. 71: 0 > 1, reversed in Mirandornithes, *Pterocles, Ardeotis, Tapera, Aegotheles, Streptoprocne*, Gruiformes, Aequornithes, *Bucorvus, Nestor*, and *Acanthisitta*; also found in *Dendrocygn*a and *Ortalis* + *Rollulus*); P15, W24.
- Little expansion of external lip of coracoid (char. 86: 1 > 0, reversed in Columbimorphae, *Ardeotis, Tapera*, Grues, *Rostratula, Alca, Sterna, Oceanites*, Suliformes, *Scopus, Pelecanus, Coragyps, Pandion, Leptosomus, Upupa*, Pici, and *Nestor*); K21.
- Prominent expansion of internal lip of coracoid (char. 88: 0 > 1, reversed in *Podilymbus, Pterocles, Caprimulgus, Psophia*, Ralloidea, *Charadrius, Rostratula, Turnix*, and *Urocolius*; also found in *Anseranas* and *Alectura*); J14, W24.
- Ridge ventral to impression of m. pectoralis on humerus prominent (char. 114: 0 > 1, reversed in Mirandornithes, *Monias, Tapera*, Apodiformes, *Sarothrura, Rallus, Charadrius, Limosa, Alca, Gavia, Oceanites* + *Pagodroma*, Pelecanimorphae, *Pandion* + *Elanus, Urocolius*, Pici, *Nestor*, and *Acanthisitta*; also found in *Anseranas* and *Ortalis*); P15, W24.
- Internal index on phalanx 1 of major digit well developed (char. 200: 0 > 1, reversed in *Podilymbus, Monias, Corythaeola, Opisthocomus*, Gruiformes, *Turnix, Spheniscus, Tigrisoma, Ninox, Urocolius, Bucorvus, Merops*, Piciformes, and Australaves; also found in *Ichthyornis* and Anseres); J14.

Neoavians exhibit a great degree of anatomical disparity, and morphological synapomorphies characterizing the entire clade have therefore been challenging to identify, with previously proposed synapomorphies being largely limited to cranial and soft tissue traits (Livzey and Zusi 2007; Mayr 2008a; Mayr 2011a). The characters listed above are highly variable within Neoaves and can also be found in non-neoavian birds, so their identification as potential synapomorphies should be considered tentative.

#### Mirandornithes

Twenty-one character states were optimized as potential synapomorphies of Mirandornithes, of which four were consistently inferred as such across all alternative molecular topologies studied.

**Fig. 23:**
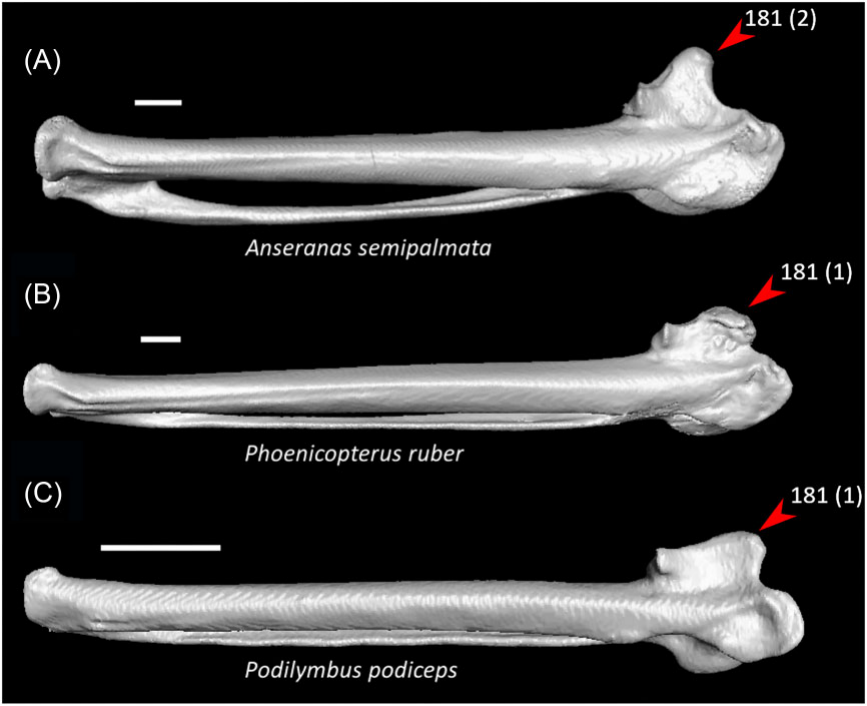
Carpometacarpi of *Anseranas semipalmata* (**A**, NHMUK 1852.7.22.1, left element), *Phoenicopterus ruber* (**B**, UMZC 346.B, left element), and *Podilymbus podiceps* (**C**, UMMZ 205087, right element, mirrored) in dorsal view. Scale bars = 5 mm. Arrows indicate the extensor process. *Anseranas* exhibits state 2 for character 181 (extensor process surpassing the distal articular facet for alular digit by the width of the facet or more), whereas *Phoenicopterus* and *Podilymbus* exhibit state 1 (extensor process on the carpometacarpus surpassing the distal articular facet by less than the width of the facet, optimized as a synapomorphy of Mirandornithes).

- Strong craniocaudal curvature of furcula (char. 2: 0 > 1, also found in Anseres, *Nyctibius*, Charadriiformes, Aequornithes, *Pandion, Trogon*, and *Coracias* + *Merops*); J14, K21, S24, W24.
- Ventrolateral sulcus on sternum indistinct (char. 34: 1 > 0, also found in non-neoavian birds, *Caprimulgus, Aegotheles*, Gruoidea, *Sterna, Eurypyga, Phoebastria, Leptoptilos*, Pelecaniformes, *Bucorvus*, and *Nestor*); P15, W24.
- Pneumatic pores in intercostal incisures of sternum absent (char. 37: 1 > 0, also found in *Rollulus, Caprimulgus, Aegotheles*, Heliornithes, Charadriiformes, *Gavia, Spheniscus, Oceanites, Pagodroma, Leucocarbo, Urocolius*, Piciformes, *Neopelma*, and *Menura*); J14, K21, S24, W24.
- Caudolateral trabeculae of sternum extend caudal to median trabecula (char. 62: 0 > 1, also found in *Gansus, Ichthyornis, Dendrocygna*, Strisores, Heliornithes, *Phaethon, Spheniscus, Phoebastria*, Suliformes, *Scopus, Pelecanus, Pandion* + *Elanus, Tyto*, Pici, *Micrastur*, and *Nestor*); J14, P15, K21, S24, W24.
- Impression for acrocoracohumeral ligament on coracoid deep (char. 66: 0 > 1, also found in *Eudromia, Chauna, Dendrocygna, Streptoprocne, Rallus, Rostratula, Turnix, Pagodroma, Leptoptilos*, Suloidea, *Eudocimus, Pelecanus*, Accipitrimorphae, Strigiformes, *Urocolius*, Eucavitaves, and Psittacopasseres); J14, P15, W24.
- Coracoid rounded and relatively thick at the ventromedial margin of supracoracoid sulcus (char. 71: 1 > 0, also found in non-neoavian birds, *Pterocles, Ardeotis, Tapera, Aegotheles, Streptoprocne*, Gruiformes, Aequornithes, *Bucorvus, Nestor*, and *Acanthisitta*); P15, W24.
- Strong lateral curvature of scapular shaft (char. 98: 0 > 1, also found in *Ichthyornis*, Anseriformes, *Charadrius, Limosa, Eurypyga*, Aequornithes, and *Leptosomus*); J14.
- Ventral tubercle on humerus distal to dorsal tubercle (char. 103: 1 > 0, also found in *Eudromia, Dendrocygna*, Galliformes, Pteroclimesites, *Florisuga, Turnix, Alca, Gavia, Spheniscus, Urocolius*, Piciformes, Tyranni, and *Climacteris*); P15, W24.
- Dorsal tubercle of humerus pointed (char. 104: 0 > 1, also found in Anseriformes, *Nyctibius, Florisuga, Aramus*, Ralloidea, Phaethoquornithes, *Pandion*, Strigiformes, *Trogon, Upupa, Coracias*, and Passeri); J14, K21, S24.
- Ridge ventral to impression of m. pectoralis on humerus absent or weak (char. 114: 1 > 0, also found in non-neoavian birds, *Monias, Tapera*, Apodiformes, *Sarothrura, Rallus, Charadrius, Limosa, Alca, Gavia, Oceanites* + *Pagodroma*, Pelecanimorphae, *Pandion* + *Elanus, Urocolius*, Pici, *Nestor*, and *Acanthisitta*); P15, W24.
- Deltopectoral crest rounded dorsally (char. 121: 1 > 0, also found in *Gansus, Chauna, Anseranas, Ardeotis, Aegotheles, Opisthocomus*, Gruiformes, *Charadrius, Rostratula, Alca, Eudocimus*, Eucavitaves, and Australaves); J14, P15, K21, S24.
- Humeral shaft straight (char. 125: 0 > 1, also found in *Pterocles, Columba*, Apodiformes, *Opisthocomus*, Charadriiformes, *Phaethon*, Procellariimorphae, *Fregata, Leucocarbo, Urocolius, Psilopogon, Psittacus, Neopelma*, and Passeri); K21, S24.
- Olecranon fossa on humerus shallow (char. 127: 1 > 0, also found in non-neognath birds, *Anseranas, Monias, Ardeotis, Nyctibius, Opisthocomus*, Grues, *Alca, Gavia, Leptoptilos*, and *Eudocimus*); J14, P15.
- Proximal margin of dorsal condyle on humerus pointed (char. 139: 0 > 1, also found in *Dendrocygna, Podargus, Aegotheles, Balearica, Charadrius, Alca*, Procellariiformes, *Leptoptilos*, Suloidea, *Scopus* + *Pelecanus*, Accipitrimorphae, *Leptosomus, Trogon, Coracias, Micrastur, Psittacus*, and Eupasseres); J14, P15, K21, S24, W24.
- Proximal margin of dorsal condyle on humerus separated from brachial fossa by smooth area of bone (char. 140: 1 > 0, also found in *Gansus, Eudromia*, Anseres, *Ardeotis, Nyctibius, Podargus, Aramus, Burhinus, Alca, Phaethon*, Aequornithes, Strigiformes, *Leptosomus, Trogon, Bucorvus*, and *Coracias*); J14.
- Radial incisure on ulna prominent (char. 155: 0 > 1, also found in *Ichthyornis, Eudromia, Chauna, Anseranas*, Mirandornithes, *Ardeotis, Nyctibius*, Grues, Charadriiformes, *Phaethon, Phoebastria, Pagodroma, Leptoptilos, Fregata, Sula, Scopus* + *Pelecanus*, Accipitrimorphae, and Psittaciformes); W24.
- Scapulotricipital impression on ulna deep (char. 156: 0 > 1, also found in Columbimorphae, Strisores, *Rallus*, Charadriiformes, *Phaethon, Gavia*, Procellariiformes, Suliformes, *Eudocimus, Scopus, Pandion*, Strigiformes, *Trogon, Bucco, Psilopogon*, and Psittacopasseres); S24.
- Minor metacarpal weakly bowed (char. 173: 1 > 0, also found in *Gansus, Ichthyornis, Dendrocygna*, Apodiformes, Charadriiformes, *Phaethon, Gavia*, Procellariimorphae, *Fregata, Eudocimus, Pandion*, Picodynastornithes, and Psittacopasseres); J14, S24.
- Minor metacarpal prominently narrows distally in caudal view (char. 179: 0 > 1, also found in *Ichthyornis*, Anseres, Columbimorphae, *Aegotheles*, Gruiformes, Charadriiformes, Phaethontimorphae, Pelecanimorphae, *Leptosomus, Coracias*, Accipitrimorphae, Strigiformes, and Australaves); W24.
- Extensor process on carpometacarpus surpasses distal articular facet for alular digit by less than width of facet (Fig. 23, char. 181: 2 > 1, also found in *Ichthyornis, Eudromia, Monias, Corythaeola, Opisthocomus, Charadrius, Phaethon*, Bucerotiformes, *Nestor*, and *Climacteris*); J14, P15, K21, S24, W24.
- Distal synostosis of major and minor metacarpals longer than craniocaudal width (char. 197: 0 > 1, also found in *Ichthyornis, Eudromia*, Anseriformes, *Monias, Psophia*, Ralloidea, *Charadrius*, Scolopaci, *Sterna, Gavia, Eudocimus*, and *Cariama*); W24.
- Phalanx 1 of major digit elongate (char. 201: 0 > 1, also found in *Fregata*); J14, P15, K21, S24, W24.

That only a few of these characters were recovered as synapomorphies across all alternative topologies indicates that their optimization is highly sensitive to the placement of Mirandornithes within Neoaves. An elongate phalanx 1 of the major digit was previously considered a synapomorphy of Mirandornithes by Mayr (2004a). Another mirandornithean synapomorphy identified by Mayr (2004a) was the presence of an oval depression on the humerus at the insertion site of the m. scapulohumeralis cranialis. This character state was included in the present study (char. 108: 1), but it was not optimized as a synapomorphy of this clade because it happens to be absent or indistinct in *Phoenicopterus ruber* (Mayr 2004a, 2014b), the species of flamingo (Phoenicopteridae) used in our dataset. In general, this feature is less prominent in extant phoenicopterids than in grebes (Podicipedidae), stem-phoenicopterids, and *Juncitarsus* (Mayr 2014b), though a distinct depression has been reported in *Phoenicopterus chilensis* (Mayr 2004a). The recognition of sternal caudolateral trabeculae that caudally surpass the median trabecula, a rounded deltopectoral crest, and an extensor process of the carpometacarpus only slightly surpassing the distal articular facet for the alular digit as potential synapomorphies of Mirandornithes is, to our knowledge, novel.

Most of the above characters cannot be assessed in published descriptions of *Juncitarsus* (Olson and Feduccia 1980a; Peters 1987; Ericson 1999; Mayr 2014b), though Mayr (2014b) noted that *Juncitarsus* lacks an elongate phalanx 1 of the major digit and interpreted this as evidence supporting its placement outside of crown-group Mirandornithes.

#### Columbimorphae

Twelve character states were optimized as potential synapomorphies of Columbimorphae, but none of these were consistently inferred as such across all alternative molecular topologies studied. Three character states were optimized as potential synapomorphies under all molecular reference topologies except Kuhl et al. (2021), in which cuckoos (Cuculidae) are included in this clade.

**Fig. 24:**
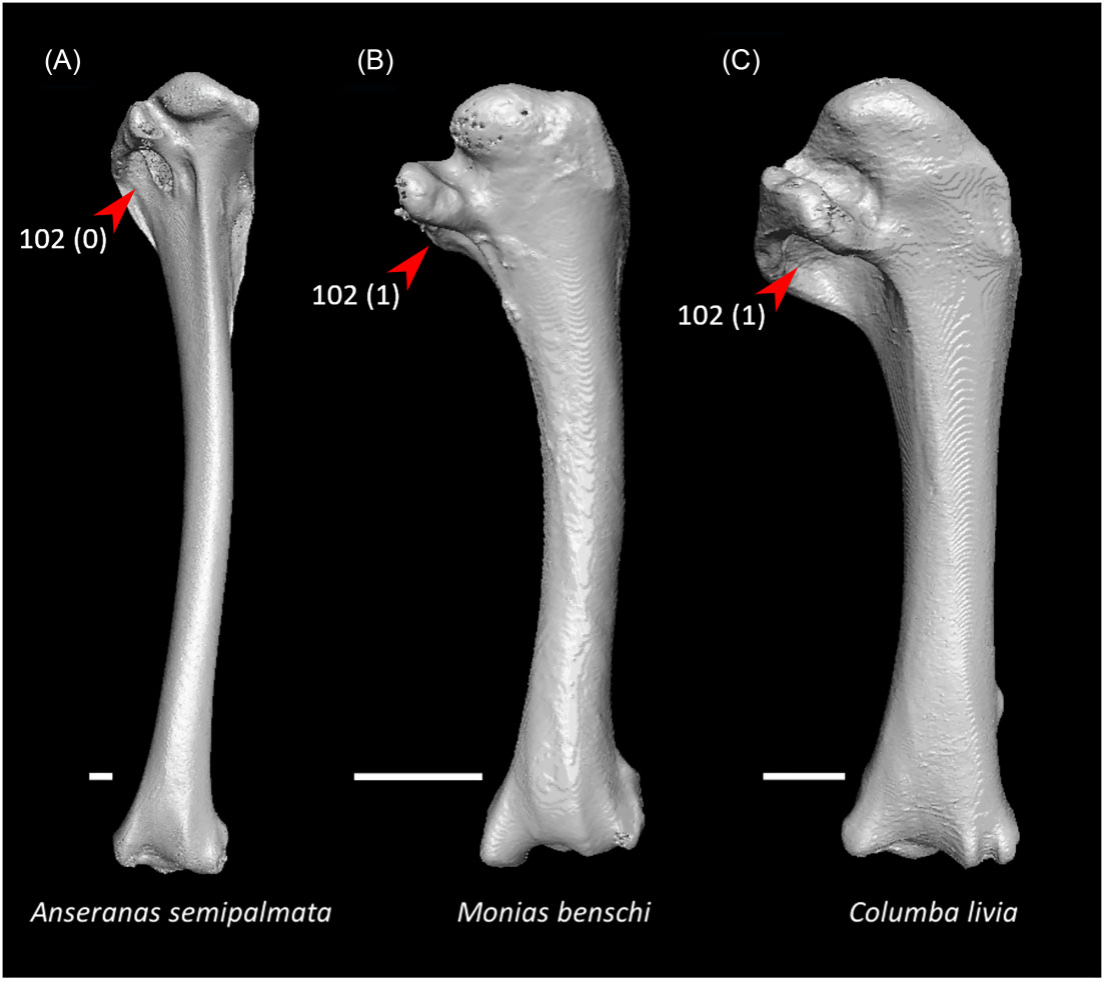
Humeri of *Anseranas semipalmata* (**A**, NHMUK 1852.7.22.1, left element, mirrored), *Monias benschi* (**B**, NHMUK 1924.11, right element), and *Columba livia* (**C**, FMNH 347273, left element, mirrored) in caudal view. Scale bars = 5 mm. Arrows indicate the pneumotricipital fossa. *Anseranas* exhibits state 0 for character 102 (pneumotricipital fossa largely exposed in caudal view), whereas *Monias* and *Columba* exhibit state 1 (pneumotricipital fossa largely concealed by the ventral tubercle in caudal view, optimized as a synapomorphy of Columbimorphae).

- Pair of cranially extending flanges absent from dorsal lip of coracoid sulci on sternum (char. 22: 1 > 0, also found in *Ichthyornis, Eudromia, Alectura, Ortalis*, Apodiformes, *Phoebastria, Fregata, Urocolius*, and Psittaciformes); P15, S24, W24.
- Cranial extent of lateral fenestrae greater than one-third of total sternum length (char. 52: 0 > 1, also found in *Eudromia, Rollulus, Caprimulgus*, Letornithes, *Sarothrura, Rallus, Limosa, Turnix, Gavia, Spheniscus, Urocolius*, Piciformes, and *Acanthisitta*); J14, P15, S24, W24.
- Transverse expansion on caudolateral trabecula of sternum (char. 54: 0 > 1, also found in *Rollulus, Podilymbus, Tapera, Aegotheles, Opisthocomus, Turnix, Coragyps, Ninox, Urocolius*, Eucavitaves, *Acanthisitta, Erythropitta*, and *Climacteris*); P15, K21, S24.
- Lateral extent of lateral process on coracoid half the width of sternal facet or greater (char. 81: 0 > 1, also found in *Corythaeola, Tapera, Caprimulgus, Podargus*, Charadriiformes, *Phoebastria, Pagodroma, Fregata, Sula, Scopus, Pelecanus, Elanus, Tyto, Leptosomus, Upupa, Coracias* + *Merops*, and *Bucco*); J14.
- Ventral tubercle on humerus largely concealing pneumotricipital fossa (Fig. 24, char. 102: 0 > 1, also found in *Gansus, Florisuga, Turnix, Alca, Spheniscus, Pagodroma, Urocolius, Alcedo*, Piciformes, *Micrastur, Neopelma*, and *Climacteris*); J14, P15, S24, W24.
- Dorsal tubercle on humerus proximodistally expanded (char. 105: 0 > 1, also found in *Streptoprocne, Sarothrura, Turnix, Alca, Spheniscus, Oceanites*, and Psittaciformes); J14, P15, S24, W24.
- Capital shaft ridge on humerus absent (char. 106: 1 > 0, also found in *Eudromia*, Galliformes, *Corythaeola, Caprimulgus*, Daedalornithes, *Opisthocomus*, Gruoidea, *Alca, Eurypyga, Spheniscus, Oceanites, Leptoptilos, Eudocimus, Tigrisoma, Urocolius, Cariama, Micrastur*, and *Acanthisitta*); J14, W24.
- Deltopectoral crest caudally convex (char. 124: 0 > 1, also found in *Eudromia*, Galliformes, *Podilymbus, Ardeotis*, Daedalornithes, Ralloidea, *Rostratula, Turnix, Alca, Spheniscus, Trogon, Alcedo*, Psittaciformes, and *Acanthisitta*); P15, S24, W24.
- Brachial fossa on humerus shallow (char. 134: 1 > 0, also found in *Gansus, Eudromia*, Galliformes, *Podilymbus, Tapera, Sarothrura, Rallus, Rostratula, Turnix, Alca, Eurypyga, Phoebastria, Oceanites, Tigrisoma, Elanus, Ninox, Upupa, Alcedo, Cariama, Psittacus*, and *Erythropitta*); P15, K21.
- Scapulotricipital impression on ulna deep (char. 156: 0 > 1, also found in Mirandornithes, Strisores, *Rallus*, Charadriiformes, *Phaethon, Gavia*, Procellariiformes, Suliformes, *Eudocimus, Scopus, Pandion*, Strigiformes, *Trogon, Bucco, Psilopogon*, and Psittacopasseres); P15, S24.
- Minor metacarpal prominently narrows distally in caudal view (char. 179: 0 > 1, also found in *Ichthyornis*, Anseres, Mirandornithes, *Aegotheles*, Gruiformes, Charadriiformes, Phaethontimorphae, Pelecanimorphae, *Leptosomus, Coracias*, Accipitrimorphae, Strigiformes, and Australaves); W24.
- Cranial projection on distal end of major metacarpal weak (char. 196: 1 > 0, also found in *Gansus, Ortalis* + *Rollulus, Corythaeola, Tapera, Opisthocomus, Psophia, Aramus*, Heliornithes, *Rostratula, Turnix, Spheniscus, Tigrisoma*, Strigiformes, *Upupa, Merops, Bucco, Cariama*, and Passeriformes); J14.

Sangster et al. (2022) previously inferred a proximodistally elongated dorsal tubercle on the humerus as a potential synapomorphy of this clade. The fossil record of all potential columbimorph groups is extremely poorly known, and few stem members of the major constituent clades have been convincingly identified (Mayr 2017b, 2022a). The stem-sandgrouse (stem-Pteroclidae) *Archaeoganga larvatus* and *Leptoganga* do, however, exhibit a ventral tubercle that largely conceals the pneumotricipital fossa, the absence of a capital ridge shaft, and a shallow brachial fossa on the humerus (Pl. 2 in Mourer-Chauviré 1993).

#### Pteroclimesites

Eight character states were optimized as potential synapomorphies of Pteroclimesites, of which one was consistently inferred as such across all alternative molecular topologies studied.

**Fig. 25:**
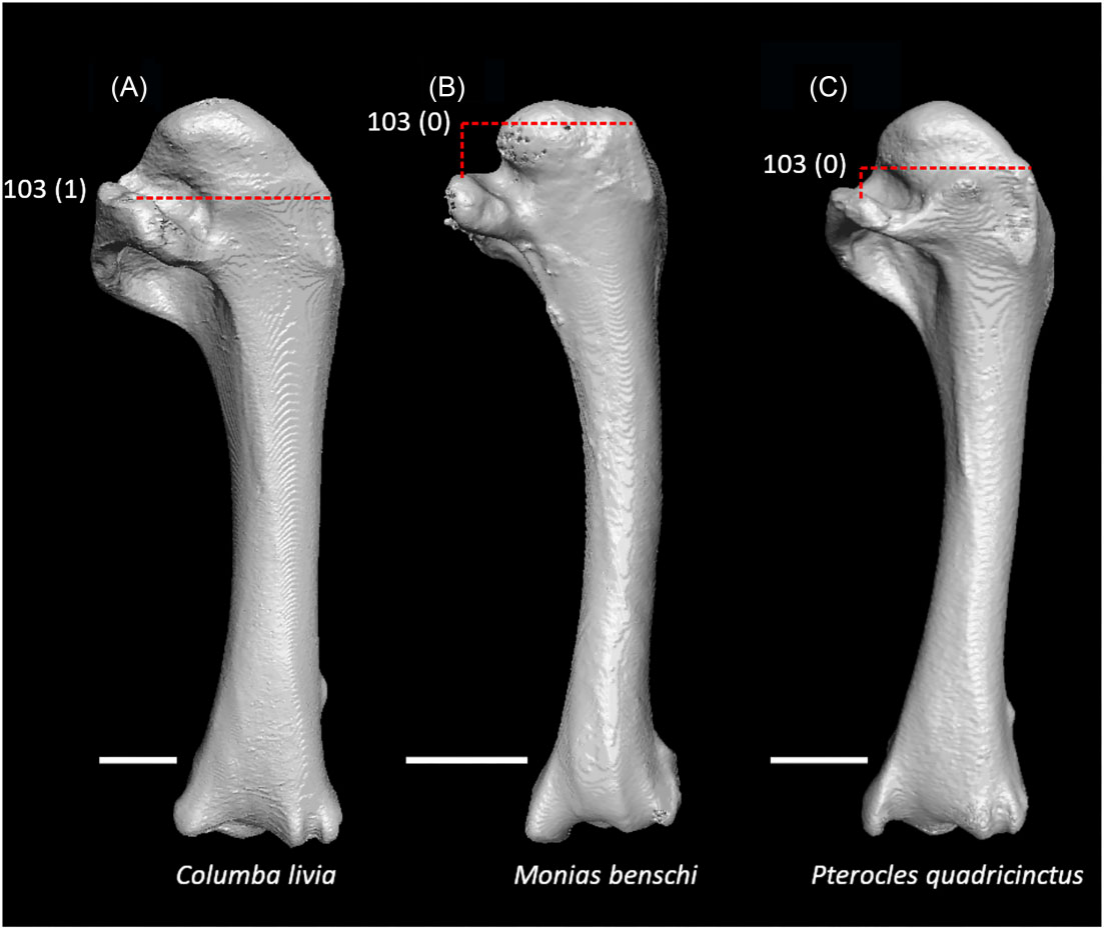
Humeri of *Columba livia* (**A**, FMNH 347273, left element, mirrored), *Monias benschi* (**B**, NHMUK 1924.11, right element), and *Pterocles quadricinctus* (**C**, FMNH 319937, left element, mirrored) in caudal view. Scale bars = 5 mm. Dotted lines indicate the proximal extent of the dorsal tubercle relative to that of the ventral tubercle. *Columba* exhibits state 1 for character 103 (ventral tubercle subequal in elevation to dorsal tubercle), whereas *Monias* and *Pterocles* exhibit state 0 (ventral tubercle distal to dorsal tubercle, optimized as a synapomorphy of Pteroclimesites).

- External spine on sternum distinctly tapered (char. 16: 0 > 1, also found in *Ardeotis, Caprimulgus, Nyctibius, Balearica, Turnix, Phaethon, Phoebastria, Leptoptilos, Tigrisoma, Ninox, Urocolius, Alcedo*, Piciformes, and Passeriformes); J14, P15, S24, W24.
- Keel sulcus present on sternum (char. 41: 0 > 1, also found in *Eudromia, Dendrocygna*, Galliformes, *Phoenicopterus, Tapera, Psophia, Rallus, Charadrius, Limosa, Eurypyga, Pagodroma, Leptoptilos, Coragyps*, and *Micrastur*); J14, P15, S24, W24.
- Longitudinal concavity of scapula prominent (char. 95: 0 > 1, also found in *Gansus, Anseranas*, Galliformes, *Tapera, Opisthocomus, Aramus*, Heliornithes, *Eudocimus, Urocolius, Leptosomus, Trogon*, Pici, *Cariama, Micrastur*, and Tyranni); J14, P15, S24, W24.
- Ventral tubercle on humerus distal to dorsal tubercle (Fig. 25, char. 103: 1 > 0, also found in *Eudromia, Dendrocygna*, Galliformes, Mirandornithes *Florisuga, Turnix, Alca, Gavia, Spheniscus, Urocolius*, Piciformes, Tyranni, and *Climacteris*); P15, K21, S24, W24.
- Ventral epicondyle on humerus equal or distal to ventral condyle (char. 130: 0 > 1, also found in Galliformes, *Phoenicopterus, Tapera*, Letornithes, *Opisthocomus, Psophia, Aramus, Sarothrura, Charadrius*, Scolopaci, *Alca, Gavia*, Procellariimorphae, *Fregata, Pelecanus* + *Tigrisoma, Elanus, Ninox*, Coraciimorphae, and Australaves); J14, P15, S24.
- Ventral aponeurosis tubercle on radius rounded (char. 148: 1 > 0, also found in *Nyctibius, Streptoprocne, Opisthocomus*, Grues, Heliornithes, *Turnix, Eurypyga, Phoebastria, Fregata, Elanus, Tyto, Urocolius, Leptosomus, Merops*, Piciformes, and Australaves); J14, P15, K21, S24, W24.
- Distinct ridge connecting caudal end of minor metacarpal and pisiform process (char. 172: 0 > 1, also found in *Tapera, Psophia, Rallus, Tyto, Leptosomus, Upupa, Cariama, Micrastur*, and *Acanthisitta*); J14, P15, S24, W24.
- Pneumatic foramen in infratrochlear fossa on carpometacarpus absent (char. 190: 1 > 0, also found in Mirandornithes, Strisores, *Opisthocomus, Rallus*, Scolopaci + Lari, Procellariimorphae, *Leucocarbo, Eudocimus, Tigrisoma, Ninox, Urocolius, Merops, Jynx, Pandion, Nestor*, and Passeriformes); K21.

Most of these characters are difficult to assess in previously identified stem-pteroclid fossils based on published descriptions, though *Archaeoganga* and *Leptoganga* do have a ventral tubercle distal to the dorsal tubercle and a ventral epicondyle equal or distal to the ventral condyle on the humerus (Pl. 2 in Mourer-Chauviré 1993).

Using the phylogenetic matrix of Livezey and Zusi (2006), Sangster et al. (2022) optimized a sternum with a median trabecula equal in length or longer than the caudolateral trabeculae, which in turn are equal in length or longer than the intermediate trabeculae, as a potential synapomorphy of Pteroclimesites. However, this character is not strictly applicable to mesites (Mesitornithidae), which only have a single pair of sternal incisures (and thus lack intermediate trabeculae).

#### Strisores

Thirteen character states were optimized as potential synapomorphies of Strisores, of which two were consistently inferred as such across all alternative molecular topologies studied.

- Hypocleideum less than 5% the total length of the furcula (char. 9: 1 > 0, reversed in *Streptoprocne*; also found in *Podilymbus*, Scolopaci, *Alca*, Phaethoquornithes, *Coragyps, Bucorvus, Alcedo, Cariama*, and *Menura*); J14, P15, K21.
- External spine of sternum less than 5% total sternum length (char. 15: 1 > 0, also found in *Chauna, Pterocles, Ardeotis*, Gruiformes, *Alca, Phaethon, Gavia, Phoebastria, Leptoptilos, Leucocarbo, Eudocimus, Coragyps*, and *Leptosomus*); J14, P15.
- Costal margin less than 25% of total sternum length (char. 35: 1 > 0, reversed in *Streptoprocne*; also found in *Gansus, Eudromia*, Galliformes, Mirandornithes, Columbimorphae, *Ardeotis, Tapera, Sarothrura, Burhinus, Rostratula, Turnix, Tigrisoma, Ninox, Urocolius*, Eucavitaves, and Eupasseres); S24.
- Ventromedial intermuscular line on sternum absent or weak (char. 39: 1 > 0, reversed in Apodiformes; also found in *Gansus, Ichthyornis, Chauna, Corythaeola, Ardeotis, Tapera, Opisthocomus, Rallus, Turnix*, Phaethontimorphae, *Gavia, Pelecanus, Elanus, Ninox*, Coraciimorphae, and Passeriformes); P15, W24.
- Dorsoventral orientation of acrocoracoid process essentially coplanar with main craniocaudal axis of coracoid (char. 64: 0 > 1, also found in Galliformes, *Pterocles, Columba, Psophia*, Heliornithes, Charadriiformes, *Urocolius*, Eucavitaves, Tyranni, and *Climacteris*); J14, P15, K21, S24.
- Lateral process of coracoid strongly hooked (char. 82: 0 > 1, reversed in Daedalornithes; also found in *Gansus, Ichthyornis, Anseranas, Rollulus, Ardeotis, Tapera*, Grues, *Burhinus, Sterna, Phaethon*, Procellariiformes, *Leptoptilos, Fregata, Leucocarbo, Scopus* + *Pelecanus, Pandion, Trogon, Bucorvus, Coracias, Bucco*, and *Psittacus*); P15, K21, W24.
- Transverse sulcus on humerus intermediate in length (char. 115: 0 > 1, reversed in *Podargus*; also found in *Anseranas, Balearica, Charadrius, Gavia, Oceanites* + *Pagodroma*, Pelecanimorphae, *Coragyps, Elanus, Tyto, Coracias*, and Psittaciformes); J14, P15, K21, W24.
- Ventral epicondyle on humerus proximal to ventral condyle (char. 130: 1 > 0, reversed in Letornithes; also found in *Ichthyornis, Eudromia*, Anseriformes, *Podilymbus, Columba, Corythaeola, Ardeotis, Balearica, Podica, Rallus, Burhinus, Turnix, Sterna, Phaethon, Oceanites, Leptoptilos*, Suloidea, *Eudocimus, Coragyps, Pandion, Tyto*, and *Nestor*); S24.
- Radius craniocaudally curved (char. 144: 0 > 1, reversed in Apodiformes; also found in Anseriformes, *Phoenicopterus, Ardeotis*, Grues, Charadrii, Feraequornithes, *Coragyps, Pandion*, Strigiformes, Cavitaves, *Micrastur*, Tyranni, and *Climacteris*); J14, P15, K21, S24, W24.
- Apex of dorsal cotylar process of ulna approximately coplanar with dorsal surface of ulnar main body (char. 149: 0 > 1, reversed in *Aegotheles* and *Streptoprocne*; also found in *Ichthyornis*, Anseriformes, *Podilymbus, Corythaeola, Opisthocomus*, Charadriiformes, *Urocolius*, Cavitaves, *Nestor*, and Passeriformes); J14.
- Scapulotricipital impression on ulna deep (char. 156: 0 > 1, reversed in Daedalornithes; also found in Mirandornithes, Columbimorphae, *Rallus*, Charadriiformes, *Phaethon, Gavia*, Procellariiformes, Suliformes, *Eudocimus, Scopus, Pandion*, Strigiformes, *Trogon, Bucco, Psilopogon*, and Psittacopasseres); J14, P15.
- Ventral collateral ligamental tubercle on ulna strongly developed (char. 157: 0 > 1, also found in *Ichthyornis, Ortalis, Corythaeola, Tapera, Psophia*, Charadriiformes, Procellariiformes, *Fregata, Pelecanus, Elanus*, Strigiformes, Coraciimorphae, *Nestor*, and Passeriformes); P15, K21, S24.
- Phalanx 1 of major digit fenestrated (char. 203: 1, reversed in *Podargus*; also found in *Pterocles, Sterna, Phaethon, Fregata, Elanus*, and *Tyto*); J14, P15, K21, S24, W24.

Using the phylogenetic matrix of Livezey and Zusi (2006), Ericson and Qu (2025) also inferred a spatulate articulation with the furcula on the acrocoracoid process of the coracoid as a potential synapomorphy of this clade. A prominent transverse sulcus on the humerus was considered a possible synapomorphy of Strisores by Cracraft (1988) and Chen et al. (2019). This feature has been documented in a wide range of fossil strisoreans, such as the putative stem-nightjar (stem-Caprimulgidae) clade Archaeotrogonidae (Mourer-Chauviré 1980; Mayr 2021b), the stem-potoo (stem-Nyctibiidae) *Paraprefica kelleri* (Mayr 1999a), the putative stem-apodiforms *Aegialornis* and *Eocypselus vincenti*, and the stem-swift (stem-Apodidae) *Scaniacypselus wardi* (Harrison 1984). Archaeotrogonids additionally exhibit a short external spine on the sternum (Mayr 1998a), and a deep scapulotricipital impression and prominently projecting (though small) ventral collateral tubercle on the ulna (Mayr 2021b). The hypocleideum is short in the archaeotrogonid *Archaeodromus* (Fig. 2i in Mayr and Kitchener 2024a); *Fluvioviridavis* (Fig. 8 in Mayr and Kitchener 2024a), which has been proposed to be a stem-frogmouth (stem-Podargidae; Nesbitt et al. 2011) or stem-oilbird (stem-Steatornithidae; Chen et al. 2019); *Eocypselus rowei* (Ksepka et al. 2013); and the stem-apodiform *Primapus* (Mayr and Kitchener 2024b), but altogether absent in the archaeotrogonid *Hassiavis* (Mayr 1998a). *Fluvioviridavis* also possesses a sharply hooked lateral process on the coracoid (Fig. 8b in Mayr and Kitchener 2024a), but has a more elongate external spine on the sternum than do extant strisoreans (Mayr and Kitchener 2024a).

A pair of depressions on the dorsal surface of phalanx 1 of the major digit was suggested by Mayr (2002a) to be a synapomorphy of a putative clade uniting Caprimulgidae, Nyctibiidae, owlet-nightjars (Aegothelidae), and Apodiformes. Although the monophyly of this assemblage has not been upheld by recent molecular phylogenetic analyses (Prum et al. 2015; Chen et al. 2019; White and Braun 2019; Kuhl et al. 2021; Stiller et al. 2024; Wu et al. 2024b), at least some of the features proposed to characterize this group may be synapomorphies of Strisores as a whole that have undergone secondary reversals in the oilbird (*Steatornis caripensis*) and podargids (Chen et al. 2019). Possessing a pair of dorsal depressions on phalanx 1 of the major digit (char. 203: 1) is itself a widespread trait among crown birds, but the present study optimizes fenestrae in these depressions as a potential strisorean synapomorphy. The presence of these fenestrae is variable in fossil strisoreans, being present in *Archaeodromus* (Mayr 2021b), the stem-steatornithid *Prefica* (Mayr 1999a), *Aegialornis* (Mourer-Chauviré 1988a), the stem-hummingbird (stem-Trochilidae) *Argornis* (Karhu 1999), and some species of *Fluvioviridavis* (Mayr and Kitchener 2024a), but absent in *Hassiavis* (Mayr 2021b), *Paraprefica* (Mayr 2005a), *Fluvioviridavis platyrhamphus* (Mayr and Kitchener 2024a), *Eocypselus* (Mayr 2010a), and the stem-trochilids *Parargornis* (Mayr 2003a) and *Eurotrochilus noniewiczi* (Bochenski and Bochenski 2008). Nonetheless, the widespread distribution of this feature within Strisores may suggest that it is an underlying synapomorphy of the clade.

Other pectoral and forelimb characters that have been proposed as putative synapomorphies of Strisores include an elongated ventral ramus of the pisiform (Mayr 2010b; char. 165: 0), an unhooked acrocoracoid process of the coracoid (Chen et al. 2019; char. 65: 0), and a caudally prominent ventral tubercle of the humerus (Ericson and Qu 2025; char. 101: 1) but these were not optimized as unambiguous synapomorphies for this clade under the topologies studied here.

#### Gruiformes

Eleven character states were optimized as potential synapomorphies of Gruiformes, of which five were consistently inferred as such across all alternative molecular topologies studied.

**Fig. 26:**
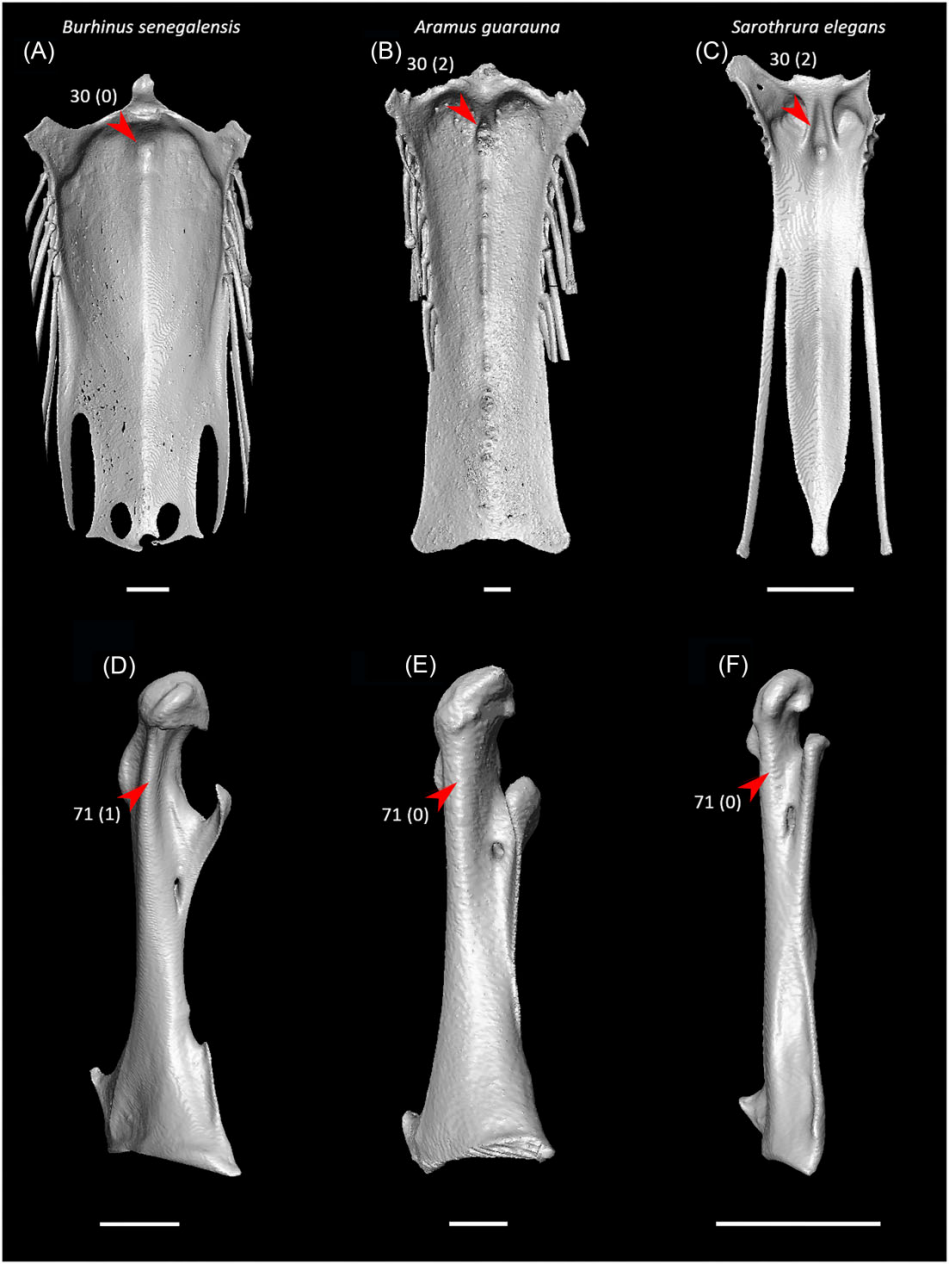
Sterna of *Burhinus senegalensis* (**A**, FMNH 313704), *Aramus guarauna* (**B**, FMNH 376078), and *Sarothrura elegans* (**C**, NHMUK S1997.34.2) in dorsal view and coracoids of *B. senegalensis* (**D**, FMNH 313704, left element, mirrored), *A. guarauna* (**E**, FMNH 376078, right element), and *S. elegans* (**F**, NHMUK S1997.34.2, right element) in ventromedial view. Scale bars = 5 mm. Arrows indicate the region immediately caudal to the cranial margin of the sternum (**A–C**) and the ventromedial margin of supracoracoid sulcus (**D–F**). The right craniolateral process of the sternum in *Sarothrura* is broken. *Burhinus* exhibits state 0 for character 30 (laminae absent from region immediately caudal to the cranial margin of the sternum), whereas *Aramus* and *Sarothrura* exhibit state 2 (two laminae in region immediately caudal to the cranial margin of the sternum, optimized as a synapomorphy of Gruiformes). *Burhinus* also exhibits state 1 for character 71 (coracoid compressed and keeled at ventromedial margin of supracoracoid sulcus), whereas *Aramus* and *Sarothrura* exhibit state 0 (coracoid rounded and relatively thick at ventromedial margin of supracoracoid sulcus, optimized as a synapomorphy of Gruiformes).

- Hypocleideum ventrocranially deflected from furcular shafts (char. 10: 1 > 2, also found in *Pterocles*, Apodiformes, *Oceanites, Leptoptilos, Coragyps, Psittacus*, and *Menura*); J14, P15, K21, S24, W24.
- External spine of sternum less than 5% total sternum length (char. 15: 1 > 0, reversed in *Podica*; also found in *Chauna, Pterocles, Ardeotis*, Strisores, *Alca, Phaethon, Gavia, Phoebastria, Leptoptilos, Leucocarbo, Eudocimus, Coragyps*, and *Leptosomus*); J14, P15, K21.
- Two laminae on dorsal surface of sternum immediately caudal to cranial margin (Fig. 26, char. 30: 0 > 2, reversed in *Podica*; also found in *Chauna, Monias, Columba*, Apodiformes, *Eurypyga, Upupa*, and *Coracias*); J14, P15, K21, S24, W24.
- Six or more articular facets on costal margin of sternum (char. 36: 2 > 3, reversed in *Sarothrura*; also found in Anseriformes, *Phoenicopterus, Alca, Sterna, Phaethon, Gavia*, Procellariimorphae, *Sula, Eudocimus*, Scopus, *Pandion* + *Elanus*, and Psittaciformes); J14, P15, K21, S24, W24.
- Median trabecula of sternum rounded at caudal termination (char. 61: 1 > 0, reversed in *Aramus*; also found in non-neognath birds, *Ortalis* + *Rollulus, Monias, Columba, Florisuga, Turnix, Alca, Eurypyga*, Aequornithes, Accipitrimorphae, *Tyto, Urocolius, Bucorvus, Bucco*, Psittaciformes, and *Menura*); J14, P15, K21, S24, W24.
- Coracoid rounded and relatively thick at ventromedial margin of supracoracoid sulcus (Fig. 26, char. 71: 1 > 0, also found in non-neoavian birds, Mirandornithes, *Pterocles, Ardeotis, Tapera, Aegotheles, Streptoprocne*, Aequornithes, *Bucorvus, Nestor*, and *Acanthisitta*); J14, P15, K21, S24, W24.
- Supracoracoid nerve foramen in coracoid present (char. 72: 1 > 2, also found in *Ichthyornis, Anseranas, Phoenicopterus, Corythaeola*, Daedalornithes, Charadrii, *Alca, Sterna, Phaethon*, Procellariiformes, *Leptoptilos, Eudocimus, Pelecanus*, Accipitrimorphae, Strigiformes, *Leptosomus*, and *Micrastur*); P15.
- Pronounced ventral curvature of scapula (char. 94: 0 > 1, reversed in *Balearica*; also found in *Caprimulgus, Nyctibius, Florisuga, Opisthocomus, Eurypyga, Spheniscus, Oceanites* + *Pagodroma, Leptoptilos, Tigrisoma, Coragyps, Pandion, Tyto, Merops, Alcedo, Psilopogon, Nestor, Acanthisitta*, and Tyranni); K21.
- Deltopectoral crest rounded dorsally (char. 121: 1 > 0, reversed in *Podica*; also found in *Gansus, Chauna, Anseranas*, Mirandornithes, *Ardeotis, Aegotheles, Opisthocomus, Charadrius, Rostratula, Alca, Eudocimus*, Eucavitaves, and Australaves); P15, K21, W24.
- Humeral shaft width approximately constant throughout length (char. 126: 2 > 0, reversed in *Podica*; also found in *Gansus, Eudromia*, Anseriformes, *Ortalis*, Mirandornithes, *Monias, Ardeotis, Limosa, Alca, Phaethon, Gavia, Phoebastria, Pagodroma*, Suliformes, *Eudocimus, Coragyps, Elanus*, Strigiformes, *Urocolius, Merops, Alcedo, Bucco, Micrastur*, and *Acanthisitta*); S24.
- Internal index on phalanx 1 of major digit poorly developed (char. 200: 1 > 0, reversed in *Balearica*; also found in *Gansus, Eudromia, Chauna, Alectura, Rollulus, Podilymbus, Monias, Corythaeola, Opisthocomus, Rostratula, Turnix, Spheniscus, Tigrisoma, Ninox, Urocolius, Bucorvus, Merops*, Piciformes, and Australaves); K21, W24.

The optimization of six or more costal facets as a synapomorphy of this clade is consistent with the presence of six costal facets on the sternum of the messelornithids *Messelornis nearctica* (Hesse 1992) and *Songzia acutunguis* (Wang et al. 2012), which recent studies suggest are stem members of Ralloidea (Mayr 2004b; Musser et al. 2019). Messelornithids possess a supracoracoid nerve foramen (Hesse 1988; Mourer-Chauviré 1995; Mayr and Kitchener 2025b), but have a dorsocaudally deflected hypocleideum (Hesse 1988, 1990), and a well-developed internal index process on phalanx 1 of the major digit (Hesse 1990).

#### Charadriiformes

Twelve character states were optimized as potential synapomorphies of Charadriiformes, of which one was consistently inferred as such across all alternative molecular topologies studied. Five character states were inferred as synapomorphies under all molecular reference topologies except for Prum et al. (2015), in which Charadriiformes was recovered as the extant sister group of Mirandornithes instead of Gruiformes.

- Strong craniocaudal curvature of furcula (char. 2: 0 > 1, reversed in *Turnix*; also found in Anseres, Mirandornithes, *Nyctibius*, Aequornithes, *Pandion, Trogon*, and *Coracias* + *Merops*); J14, K21, S24, W24.
- Pointed acromial processes on furcula (char. 5: 0 > 1, also found in *Ichthyornis*, Anseriformes, *Phoenicopterus, Pterocles, Nyctibius*, Apodiformes, *Sarothrura, Eurypyga, Gavia*, Procellariimorphae, *Sula, Coragyps, Pandion, Urocolius*, and *Acanthisitta*); J14, K21, S24, W24.
- Hypocleideum dorsocaudally deflected from furcular shafts (char. 10: 1 > 0, reversed in *Limosa* and *Sterna*; also found in *Ichthyornis, Rollulus, Podilymbus, Podargus*, Phaethontimorphae, *Phoebastria, Sula, Scopus, Tigrisoma, Urocolius, Bucorvus*, Coraciiformes, *Acanthisitta*, and *Erythropitta*); J14, K21, S24, W24.
- Pneumatic pores in intercostal incisures of sternum absent (char. 37: 1 > 0, also found in *Rollulus*, Mirandornithes, *Caprimulgus, Aegotheles*, Heliornithes, *Gavia, Spheniscus, Oceanites, Pagodroma, Leucocarbo, Urocolius*, Piciformes, *Acanthisitta, Erythropitta*, and *Climacteris*); J14, K21, S24, W24.
- Dorsoventral orientation of acrocoracoid process essentially coplanar with main craniocaudal axis of coracoid (char. 64: 0 > 1, reversed in *Limosa* and *Alca*; also found in Galliformes, *Pterocles, Columba*, Strisores, *Psophia*, Heliornithes, *Urocolius*, Eucavitaves, Tyranni, and *Climacteris*); P15.
- Acrocoracoid process on coracoid forms pronounced hook (char. 65: 0 > 1, also found in *Ichthyornis, Eudromia, Alectura, Rollulus, Podilymbus, Pterocles, Columba, Corythaeola, Tapera, Aegotheles, Streptoprocne*, Heliornithes, Phaethontimorphae, Procellariimorphae, Cavitaves, *Cariama*, Psittaciformes, and Eupasseres); S24, W24.
- Scapular cotyle on coracoid deep and cup-like (char. 68: 1 > 0, reversed in *Alca*; also found in *Gansus, Ichthyornis, Chauna, Dendrocygna, Phoenicopterus, Pterocles*, Apodiformes, *Opisthocomus, Eurypyga, Gavia, Leptoptilos, Pelecanus*, and *Tigrisoma*); K21, W24.
- Strong ventral curvature of sternal articular facet of coracoid (char. 85: 0 > 1, also found in *Chauna, Rollulus*, Gruoidea, *Sarothrura, Spheniscus, Oceanites, Coragyps*, Psittaciformes, and *Acanthisitta*); P15.
- Moderate ventral curvature of scapula (char. 94: 1 > 0, also found in most birds outside of Gruiformes, *Opisthocomus*, and some Strisores, Phaethoquornithes, and Telluraves); S24.
- Apex of dorsal cotylar process of ulna approximately coplanar with dorsal surface of ulnar main body (char. 149: 0 > 1, reversed in *Limosa*; also found in *Ichthyornis*, Anseriformes, *Podilymbus, Corythaeola*, Strisores, *Opisthocomus, Urocolius*, Cavitaves, *Nestor*, and Passeriformes); K21.
- Radial incisure on ulna prominent (char. 155: 0 > 1, reversed in *Turnix*; also found in *Ichthyornis, Eudromia, Chauna, Anseranas*, Mirandornithes, *Ardeotis, Nyctibius*, Grues, *Phaethon, Phoebastria, Pagodroma, Leptoptilos, Fregata, Sula, Scopus* + *Pelecanus*, Accipitrimorphae, and Psittaciformes); J14, K21, S24, W24.
- Tubercle at insertion point of humerocarpal ligament on pisiform (char. 166: 0 > 1, also found in *Pterocles, Columba, Aegotheles, Florisuga*, Pelecanimorphae, *Coragyps, Tyto, Micrastur, Erythropitta*, and Passeri); J14, P15, K21, S24, W24.

Using the phylogenetic matrix of Livezey and Zusi (2006), Ericson and Qu (2025) also inferred a caudoventrally placed pneumotricipital fossa on the humerus in distal view as a potential synapomorphy of this clade. A hooked acrocoracoid process of the coracoid and the presence of a tubercle at the insertion point of the humerocarpal ligament on the pisiform have been considered characteristic of Charadriiformes by previous studies (Mayr and Knopf 2007; Mayr 2008a, 2011b; Mayr and Kitchener 2023b). Additionally, strong cranial curvature of the sternal articular facet (char. 84: 1) of the coracoid as well as a long transverse sulcus (char. 115: 2) and a prominent supracondylar process (char. 136: 2) on an apneumatic (char. 109: 0) humerus are distinctive features widely found in charadriiforms (Mayr 2000b; Mayr and Knopf 2007; Mayr 2008a, 2011b, 2016a; Hood et al. 2019; Mayr et al. 2022; Burton et al. 2023; Mayr and Kitchener 2023b, 2025c), but their optimization as synapomorphies of this clade is likely sensitive to ingroup taxon sampling and outgroup interrelationships.

Pectoral girdle and forelimb elements are mostly unknown in the most completely preserved putative stem-charadriiforms, *Scandiavis* (Bertelli et al. 2013; Heingård et al. 2021) and *Nahmavis* (Musser and Clarke 2020), though Mayr and Kitchener (2023b) assigned a distal humerus from the London Clay Formation to *Scandiavis* and tentatively referred additional pectoral girdle and forelimb material from the same locality to a closely related taxon. Similarities of these specimens to crown-group charadriiforms include a hooked acrocoracoid process and the presence of a dorsal supracondylar tubercle on the humerus, though this is weakly developed (Mayr and Kitchener 2023b).

#### Phaethoquornithes

Three character states were optimized as potential synapomorphies of Phaethoquornithes, but none of these were consistently inferred as such across all alternative molecular topologies studied.

- Hypocleideum less than 5% the total length of the furcula (char. 9: 1 > 0, reversed in *Oceanites*; also found in *Podilymbus*, Strisores, Scolopaci, *Alca, Coragyps, Bucorvus, Alcedo, Cariama*, and *Menura*); J14, K21.
- Dorsal tubercle of humerus pointed (char. 104: 0 > 1, reversed in Procellariimorphae and *Tigrisoma*; also found in Anseriformes, Mirandornithes, *Nyctibius, Florisuga, Aramus*, Ralloidea, *Pandion*, Strigiformes, *Trogon, Upupa, Coracias*, and Passeri); J14, K21, S24.
- Prominent ligamental groove on ventral ramus of pisiform (char. 167: 0 > 1, reversed in *Phoebastria, Oceanites, Eudocimus*, and *Tigrisoma*; also found in *Ichthyornis, Dendrocygna, Columba, Tapera, Nyctibius, Streptoprocne*, Grues, Ralloidea, *Charadrius*, Scolopaci, *Sterna, Pandion*, Strigiformes, *Upupa, Coracias, Alcedo, Psittacus, Erythropitta*, and Passeri); J14, P15, K21, W24.

A short hypocleideum is visible in the stem-loon (stem-Gaviidae) *Nasidytes* (Fig. 3F in Mayr and Kitchener 2022), whereas a small projection for articulation with the sternum can be seen in this region of the furcula in the stem-tropicbird (stem-Phaethontidae) *Prophaethon* (Mayr 2015a). *Prophaethon* additionally exhibits a prominent ligamental groove on the ventral ramus of the pisiform (Fig. 5n in Mayr 2015a).

#### Phaethontimorphae

Six character states were optimized as potential synapomorphies of Phaethontimorphae, of which one was consistently inferred as such across all alternative molecular topologies studied.

**Fig. 27:**
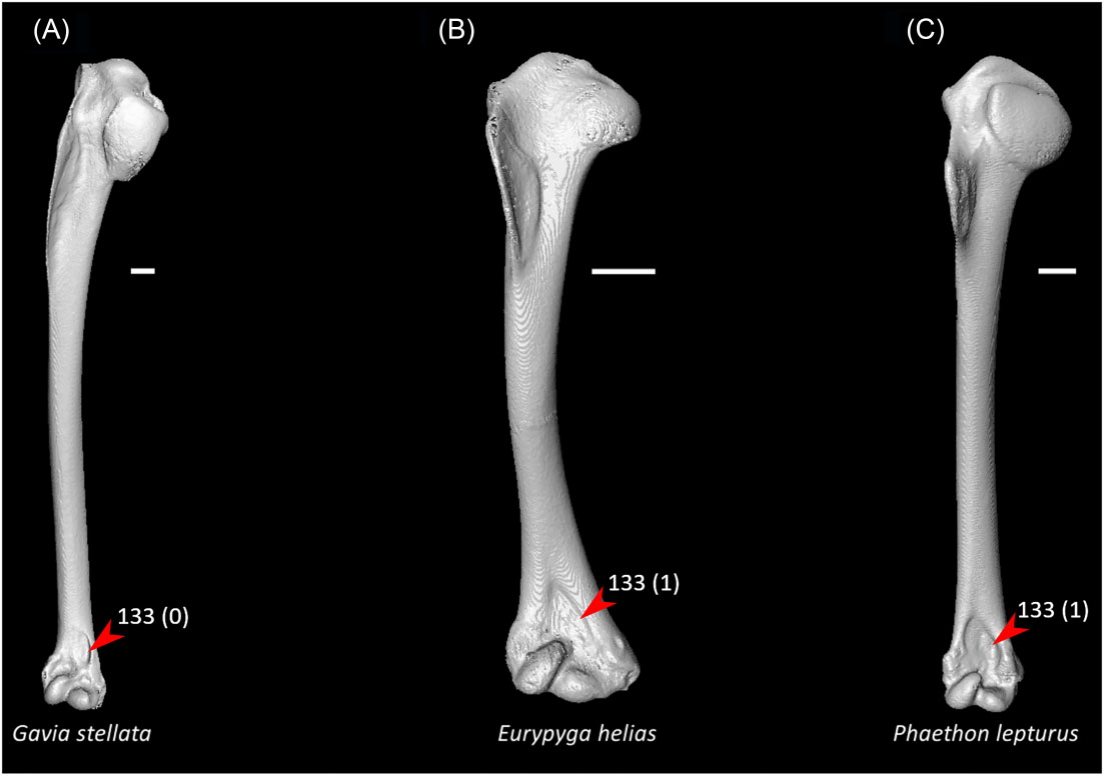
Humeri of *Gavia stellata* (**A**, NHMUK 1891.7.20.132, right element), *Eurypyga helias* (**B**, FMNH 317341, right element), and *Phaethon lepturus* (**C**, NHMUK 1876.3.16.3, right element) in cranial view. Scale bars = 5 mm. Arrows indicate the brachial fossa. *Gavia* exhibits state 0 for character 133 (brachial fossa positioned ventrally on humeral shaft), whereas *Eurypyga* and *Phaethon* exhibit state 1 (brachial fossa positioned medially on humeral shaft, optimized as a synapomorphy of Phaethontimorphae).

1. Hypocleideum dorsocaudally deflected from clavicular shafts (char. 10: 1 > 0, also found in *Ichthyornis, Rollulus, Podilymbus, Podargus*, Charadriiformes, *Phoebastria, Sula, Scopus, Tigrisoma, Urocolius, Bucorvus*, Coraciiformes, *Acanthisitta*, and *Erythropitta*); J14, K21, S24, W24.
2. Coracoid sulci on sternum crossed (char. 25: 1 > 2, also found in *Ichthyornis, Phoenicopterus, Corythaeola*, Grues, *Burhinus*, Procellariimorphae, Pelecaniformes, *Elanus, Leptosomus*, and *Micrastur*); J14, P15, K21, S24, W24.
3. Acrocoracoid process on coracoid forms pronounced hook (char. 65: 0 > 1, also found in *Ichthyornis, Eudromia, Alectura, Rollulus, Podilymbus, Pterocles, Columba, Corythaeola, Tapera, Aegotheles, Streptoprocne*, Heliornithes, Charadriiformes, Procellariimorphae, Cavitaves, *Cariama*, Psittaciformes, and Eupasseres); S24, W24.
4. Olecranon fossa on humerus deep (char. 127: 0 > 1, also found in Procellariimorphae, Suliformes, *Scopus* + *Pelecanus*, and most non-phaethoquornithean neognaths); W24.
5. Brachial fossa on humerus positioned medially on shaft (Fig. 27, char. 133: 0 > 1, also found in Anseres, *Ardeotis, Nyctibius, Opisthocomus*, Gruoidea, Procellariiformes, *Tyto*, and *Leptosomus*); J14, P15, K21, S24.
6. Minor metacarpal prominently narrows distally in caudal view (char. 179: 0 > 1, also found in *Ichthyornis*, Anseres, Mirandornithes, Columbimorphae, *Aegotheles*, Gruiformes, Charadriiformes, Pelecanimorphae, *Leptosomus, Coracias*, Accipitrimorphae, Strigiformes, and Australaves); W24.

The identification of potential morphological synapomorphies for Phaethontimorphae has been challenging (Mayr 2014c, 2019b, 2022a; Sangster et al. 2022; Mayr and Kitchener 2024c) and no well-corroborated fossils of total-group Eurypygiformes have been formally described (Mayr 2022a). Nonetheless, some of the above characters have been documented in stem-phaethontid fossils—*Prophaethon* exhibits dorsocaudally-directed projections on the furcular symphysis (Mayr 2015a), and both *Prophaethon* and *Lithoptila* exhibit a medially positioned brachial fossa on the humerus (Bourdon et al. 2008; Fig. S2b in Mayr 2015a Fig. 1; in Mayr and Kitchener 2024c). Of note is that messelornithids, which were originally interpreted as stem-eurypygids (Hesse 1990), exhibit a ventrally placed humeral brachial fossa (Mourer-Chauviré 1995), consistent with the recent consensus that they are stem-ralloids.

The crossed coracoid sulci on the sternum and medially positioned brachial fossa on the humerus distinguish phaethontimorphs from members of Suliformes, which have traditionally been thought to be closely related to phaethontids (e.g., Cracraft 1985; Livezey and Zusi 2007). However, some recent morphological analyses proposed a closer relationship between Phaethontidae and Procellariiformes (Mayr 2003b; Smith 2010), which is consistent with the fact that all of these potential phaethontimorph synapomorphies also appear to be homoplastically present in at least some procellariiforms.

One of the two extant species of Eurypygiformes, the kagu (*Rhynochetos jubatus*), lacks a caudal projection on the furcular symphysis and crossed coracoid sulci (Parker 1869). However, this species is essentially flightless (Hunt 1996) and is likely to possess a highly autapomorphic pectoral girdle and forelimb skeleton as a result.

#### Aequornithes

Four character states were optimized as potential synapomorphies of Aequornithes, of which two were consistently inferred as such across all alternative molecular topologies studied.

**Fig. 28:**
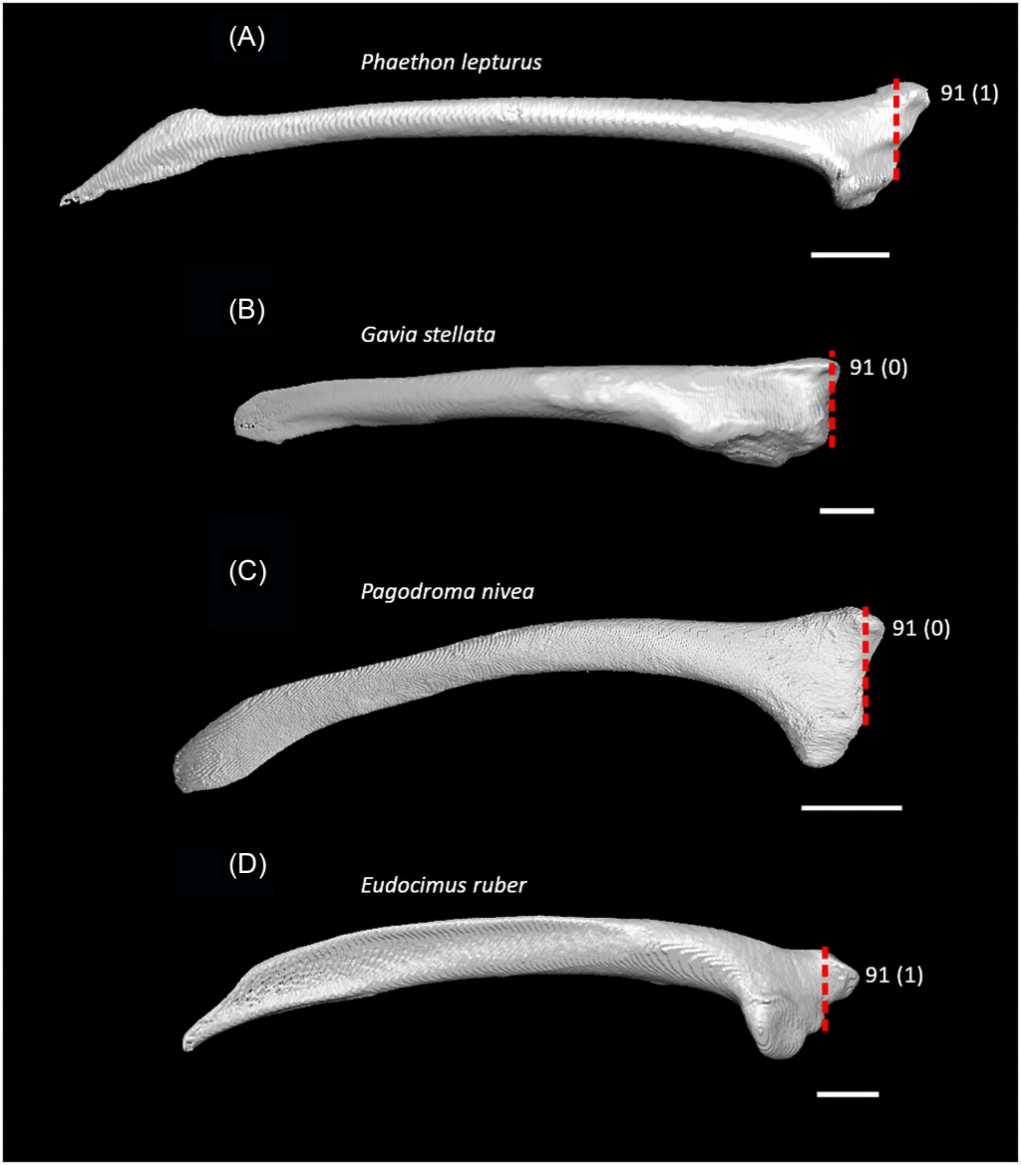
Scapulae of *Phaethon lepturus* (**A**, NHMUK 1876.3.16.3, left element, mirrored), *Gavia stellata* (**B**, NHMUK 1891.7.20.132, right element), *Pagodroma nivea* (**C**, NHMUK S1998.55, right element), and *Eudocimus ruber* (**D**, NHMUK S1999.8.1, right element) in lateral (**A, C–D**) or craniolateral (**B**) view. Scale bars = 5 mm. Dotted lines indicate cranial extent of the coracoid tubercle. *Phaethon* exhibits state 1 for character 91 (acromion extends distinctly cranial to coracoid tubercle), whereas *Gavia* and *Pagodroma* exhibit state 0 (acromion subequal or caudal to coracoid tubercle, optimized as a synapomorphy of Aequornithes). *Eudocimus* also exhibits state 1 for character 89, a reversal to the plesiomorphic condition (optimized as a synapomorphy of Pelecanes).

- Strong craniocaudal curvature of furcula (char. 2: 0 > 1, reversed in *Phoebastria, Fregata, Leucocarbo, Pelecanus*, and *Tigrisoma*; also found in Anseres, Mirandornithes, *Nyctibius*, Charadriiformes, *Pandion, Trogon*, and *Coracias* + *Merops*); J14, K21, S24, W24.
- Single pair of caudal fenestrae in sternum (char. 51: 2 > 1, reversed in *Pagodroma* and *Eudocimus*; also found in *Eudromia*, Anseres, Galliformes, Mirandornithes, *Monias, Caprimulgus, Balearica*, Ralloidea, *Turnix, Pandion* + *Elanus, Tyto*, Bucerotiformes, and Australaves); J14, S24.
- Coracoid rounded and relatively thick at ventromedial margin of supracoracoid sulcus (char. 71: 1 > 0, reversed in *Phoebastria, Pagodroma, Leucocarbo*, and *Tigrisoma*; also found in non-neoavian birds, Mirandornithes, *Pterocles, Ardeotis, Tapera, Aegotheles, Streptoprocne*, Gruiformes, *Bucorvus, Nestor*, and *Acanthisitta*); J14, P15, K21, S24, W24.
- Scapular acromion subequal or caudal to coracoid tubercle (Fig. 28, char. 91: 1 > 0, reversed in *Phoebastria* and Pelecanes; also found in *Ichthyornis, Chauna, Anseranas, Ardeotis, Nyctibius, Florisuga, Alca*, and *Sterna*); J14, P15, K21, S24, W24.

A scapular acromion equal or caudal to the coracoid tubercle is found in Paleocene stem-penguins (stem-Spheniscidae) (Mayr et al. 2017a, 2017b; Blokland et al. 2019; Mayr et al. 2021) and the possible stem-procellariiform *Rupelornis* (Mayr et al. 2002; De Pietri et al. 2010; Mayr and Smith 2012). *Rupelornis* (Mayr et al. 2002) and the stem-gaviid *Nasidytes* (Mayr and Kitchener 2022) are known to have had a single pair of caudal incisions in the sternum, as does the stem-ibis (stem-Threskiornithidae) *Rhynchaeites* (Mayr 2002b), which is noteworthy given that crown threskiornithids have a four-notched sternum. Conversely, the stem-frigatebird (stem-Fregatidae) *Limnofregata* has two pairs of caudal sternal incisions (Olson 1977), perhaps representing another reversal to this condition within Aequornithes. *Nasidytes* exhibits a more elongate scapular acromion and a less craniocaudally curved furcula compared to extant gaviids (Mayr and Kitchener 2022), which may suggest either their apomorphic presence in *Nasidytes* or multiple losses of these traits among Aequornithes.

#### Feraequornithes

Three character states were optimized as potential synapomorphies of Feraequornithes, of which two were consistently inferred as such across all alternative molecular topologies studied.

**Fig. 29:**
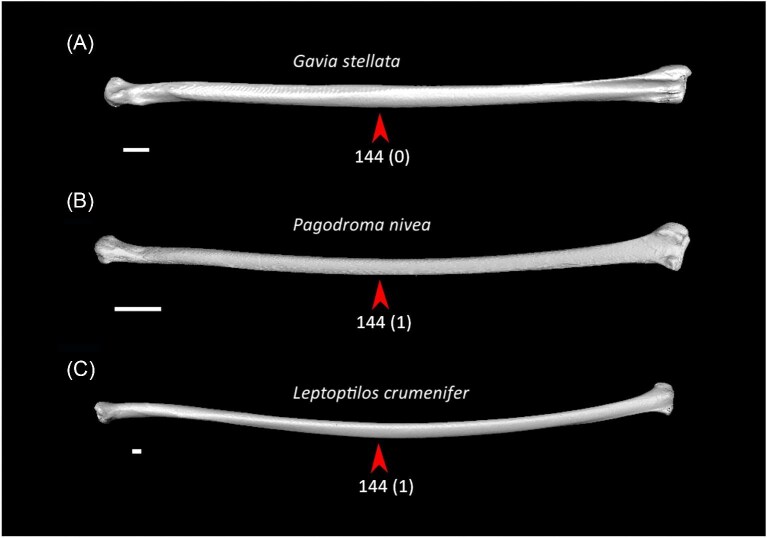
Radii of *Gavia stellata* (**A**, NHMUK 1891.7.20.132, right element), *Pagodroma nivea* (**B**, NHMUK S1998.55, right element), and *Leptoptilos crumenifer* (**C**, NHMUK S1952.3.182, right element) in dorsal view. Scale bars = 5 mm. Arrows indicate the radial shaft. *Gavia* exhibits state 0 for character 144 (radial shaft craniocaudally straight), whereas *Pagodroma* and *Leptoptilos* exhibit state 1 (radial shaft craniocaudally curved, optimized as a synapomorphy of Feraequornithes).

- Ventromedial intermuscular line on sternum prominent (char. 39: 0 > 1, reversed in *Pelecanus*; also found in many non-phaethoquornithean birds); S24, W24.
- Median trabecula of sternum distinctly tapered (char. 60: 0 > 1, reversed in *Leucocarbo* and *Pelecanus*; also found in *Gansus, Alectura, Ortalis, Monias, Ardeotis*, Letornithes, *Sarothrura, Rallus, Charadrius, Turnix, Coragyps, Ninox*, Coraciimorphae, and *Cariama*); J14, P15, K21, S24, W24. Radius craniocaudally curved (Fig. 29, char. 144: 0 > 1, reversed in *Oceanites*; also found in Anseriformes, *Phoenicopterus, Ardeotis*, Strisores, Grues, Charadrii, *Coragyps, Pandion*, Strigiformes, Cavitaves, *Micrastur*, Tyranni, and *Climacteris*); J14, P15, K21, S24, W24.

The inference of a craniocaudally curved radius as a synapomorphy of this clade is consistent with the straight radius in the stem-gaviid *Colymboides* (Storer 1956; Cheneval 1984) and the strongly curved radius in Paleocene stem-spheniscids (Mayr et al. 2017a; Blokland et al. 2019; Mayr et al. 2020a). Previously recognized morphological synapomorphies of Feraequornithes have been limited to cranial characters (Sangster and Mayr 2021).

#### Procellariimorphae

Seven character states were optimized as potential synapomorphies of Procellariimorphae, of which three were consistently inferred as such across all alternative molecular topologies studied.

- Acrocoracoid process on coracoid forms pronounced hook (char. 65: 0 > 1, also found in *Ichthyornis, Eudromia, Alectura, Rollulus, Podilymbus, Pterocles, Columba, Corythaeola, Tapera, Aegotheles, Streptoprocne*, Heliornithes, Charadriiformes, Phaethontimorphae, Cavitaves, *Cariama*, Psittaciformes, and Eupasseres); S24, W24.
- Mound-shaped tuberosity absent from lateral surface of scapula (char. 96: 1 > 0, also found in *Phaethon, Scopus, Tigrisoma*, and most non-phaethoquornithean birds); P15.
- Distal portion of scapula spatulate (char. 97: 1 > 0, also found in *Rollulus* and *Pterocles*); J14, P15. K21, S24, W24.
- Dorsal tubercle of humerus rounded (char. 104: 1 > 0, reversed in *Pagodroma*; also found in *Tigrisoma* and many non-phaethoquornithean birds); J14, P15, K21, S24, W24.
- Humeral shaft straight (char. 125: 0 > 1, reversed in *Oceanites*; also found in Mirandornithes, *Pterocles, Columba*, Apodiformes, *Opisthocomus*, Charadriiformes, *Phaethon, Fregata, Leucocarbo, Urocolius, Psilopogon, Psittacus, Neopelma*, and Passeri); J14, P15, K21, S24.
- Olecranon fossa on humerus deep (char. 127: 0 > 1, also found in Phaethontimorphae, Suliformes, *Scopus* + *Pelecanus*, and most non-phaethoquornithean neognaths); W24.
- Pneumatic foramen in infratrochlear fossa on carpometacarpus absent (char. 190: 1 > 0, reversed in *Pagodroma*; also found in Mirandornithes, Pteroclimesites, Strisores, *Opisthocomus, Rallus*, Scolopaci + Lari, *Leucocarbo, Eudocimus, Tigrisoma, Ninox, Urocolius, Merops, Jynx, Pandion, Nestor*, and Passeriformes); J14, P15, K21, S24, W24.

Sangster et al. (2022) previously inferred a straight humeral shaft as a synapomorphy of this clade, based on the phylogenetic dataset of Livezey and Zusi (2006). However, as with the present study, only extant members of Procellariimorphae were sampled in that dataset. Whereas this character occurs in *Rupelornis* (Mayr et al. 2002), Paleocene stem-spheniscids consistently exhibit a more strongly curved humeral shaft than crown spheniscids (Slack et al. 2006; Jadwiszczak et al. 2013; Mayr et al. 2017a; Blokland et al. 2019; Ksepka et al. 2023b), suggesting that this feature evolved independently in crown-group spheniscids and procellariiforms. Many of the above characters are difficult to assess in fossil procellariimorphs based on preservation and available descriptions, and detailed examination of relevant specimens will likely be necessary to confidently infer potential synapomorphies of this clade.

#### Pelecanimorphae

Six character states were optimized as potential synapomorphies of Pelecanimorphae, of which three were consistently inferred as such across all alternative molecular topologies studied.

**Fig. 30:**
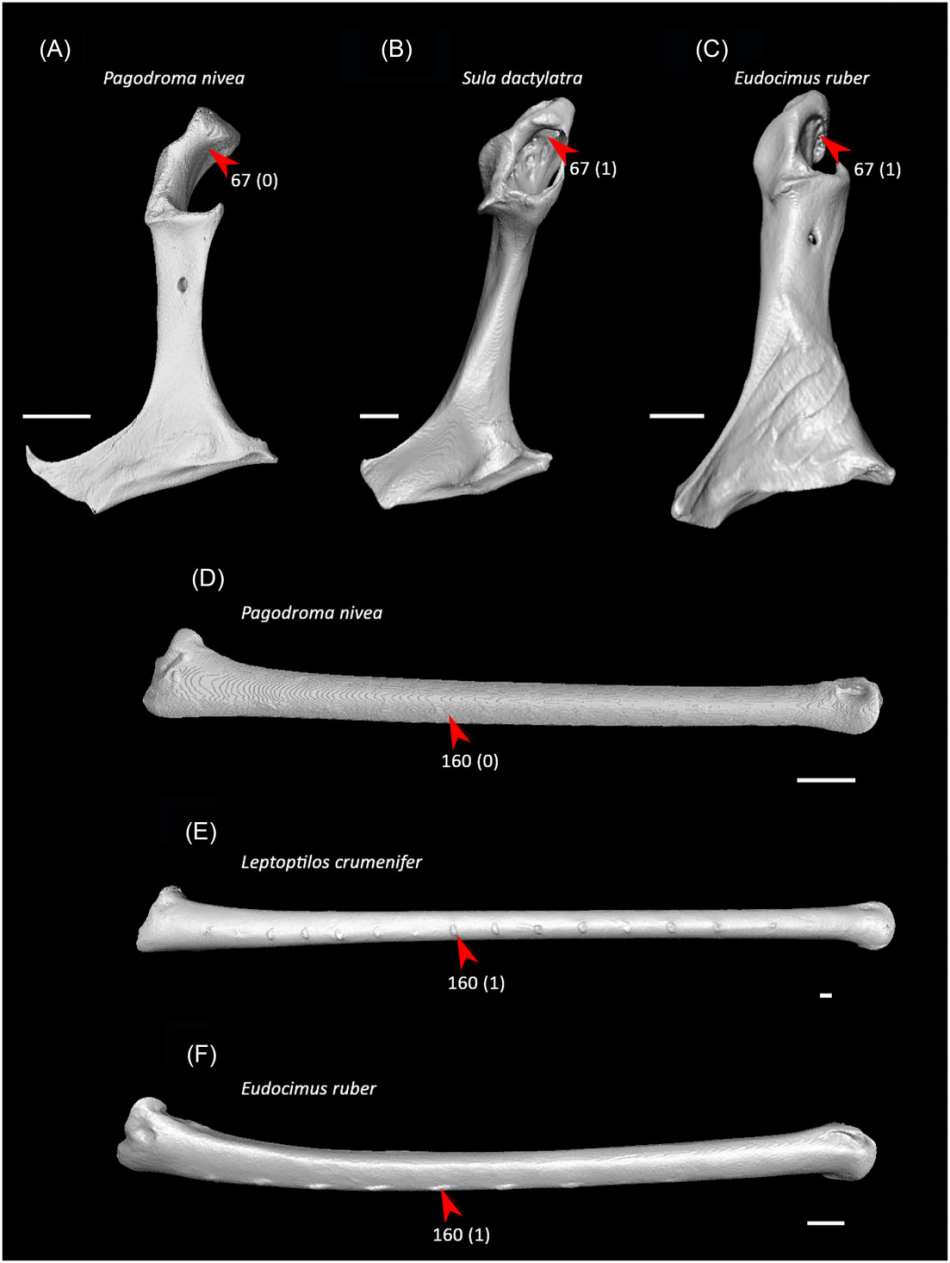
Coracoids of *Pagodroma nivea* (**A**, NHMUK S1998.55, right element, mirrored), *Sula dactylatra* (**B**, NHMUK 1890.11.3.10, right element, mirrored), and *Eudocimus ruber* (**C**, NHMUK S1999.8.1, left element) in dorsal view, and ulnae of *P. nivea* (**D**, NHMUK S1998.55, right element), *Leptoptilos crumenifer* (**E**, NHMUK S1952.3.182, right element), and *E. ruber* (**F**, NHMUK S1999.8.1, left element, mirrored) in caudal view. Scale bars = 5 mm. Arrows indicate the infra-acrocoracoid recess (**A–C**) and the ulnar shaft (**D–F**). *Pagodroma* exhibits state 0 for character 67 (infra-acrocoracoid recess shallow or absent), whereas *Sula* and *Eudocimus* exhibit state 1 (infra-acrocoracoid recess deep, optimized as a synapomorphy of Pelecanimorphae). *Pagodroma* also exhibits state 0 for character 160 (quill knobs absent or weakly developed), whereas *Leptoptilos* and *Eudocimus* exhibit state 1 (quill knobs strongly developed, optimized as a synapomorphy of Pelecanimorphae).

- Rounded acromial processes on furcula (char. 5: 1 > 0, reversed in *Sula*; also found in *Phaethon* and many non-phaethoquornithean birds); P15.
- Pneumatic foramen in median sulcus of sternum immediately caudal to cranial margin (char. 29: 0 > 1, reversed in *Leucocarbo, Scopus*, and *Pelecanus*; also found in *Ichthyornis, Dendrocygna, Alectura, Ortalis, Phoenicopterus, Pterocles, Ardeotis, Tapera*, Strisores, *Opisthocomus*, Grues, *Podica, Phaethon, Coragyps*, Strigiformes, Picocoraciades, *Micrastur*, Psittaciformes, *Neopelma*, and *Menura*); J14, P15, K21, S24.
- Infra-acrocoracoid recess in coracoid deep (Fig. 30, char. 67: 0 > 1, reversed in *Leucocarbo* and *Tigrisoma*; also found in *Ichthyornis, Pterocles, Columba, Nyctibius, Aegotheles, Streptoprocne, Psophia*, Charadriiformes, *Phaethon, Pandion* + *Elanus, Ninox*, Cavitaves, and Australaves); J14, P15, K21, S24, W24.
- Quill knobs on ulna strongly developed (Fig. 30, char. 160: 0 > 1, reversed in *Sula*; also found in *Anseranas, Podilymbus, Monias, Columba, Corythaeola, Ardeotis, Tapera, Caprimulgus, Aramus, Charadrius*, Scolopaci + Lari, *Oceanites*, Accipitrimorphae, Picocoraciades, and Passeriformes); J14, P15, K21, S24, W24.
- Tubercle at insertion point of humerocarpal ligament on pisiform (char. 166: 0 > 1, reversed in *Leucocarbo* and *Scopus*; also found in *Pterocles, Columba, Aegotheles, Florisuga*, Charadriiformes, *Coragyps, Tyto, Micrastur, Erythropitta*, and Passeri); J14, P15, K21, S24, W24.
- Minor metacarpal prominently narrows distally in caudal view (char. 179: 0 > 1, reversed in Suloidea and *Pelecanus*; also found in *Ichthyornis*, Anseres, Mirandornithes, Columbimorphae, *Aegotheles*, Gruiformes, Charadriiformes, Phaethontimorphae, *Leptosomus, Coracias*, Accipitrimorphae, Strigiformes, and Australaves); W24.

Most of these characters cannot be assessed from published descriptions of the oldest known well-corroborated members of Pelecanimorphae (Smith and Ksepka 2015), *Limnofregata* (Olson 1977; Olson and Matsuoka 2005) and *Rhynchaeites* (Peters 1983; Mayr 2002b; Mayr and Bertelli 2011; Mayr and Kitchener 2023c), though Olson (1977) noted that the quill knobs of *Limnofregata* are “not nearly as well developed as” in *Fregata*, and the acromial processes on the furcula of *Rhynchaeites* appear to be more pointed than in crown threskiornithids (Fig. 7 in Mayr and Kitchener 2023c).

Compared to many other members of Pelecanimorphae, the infra-acrocoracoid recess is modestly developed in *Leptoptilos* and apparently other storks (Ciconiidae), including the Miocene *Grallavis* (Fig. 2 in De Pietri and Mayr 2014). A deep infra-acrocoracoid recess may therefore be a synapomorphy of a less inclusive clade, such as Pelecanes.

#### Pelecanes

Three character states were optimized as potential synapomorphies of Pelecanes, of which one was consistently inferred as such across all alternative molecular topologies studied.

- Internal lip of coracoid expanded along entire width (char. 89: 1 > 0, reversed in *Leucocarbo* and *Scopus* + *Pelecanus*, also found in Galloanserae, Columbimorphae, *Aramus*, Charadriiformes, *Gavia, Pagodroma*, and Telluraves); S24, W24.
- Scapular acromion extends distinctly craniad of coracoid tubercle (Fig. 28, char. 91: 0 > 1, reversed in *Tigrisoma*; also found in most non-aequornithean birds); J14, P15, K21, S24, W24.
- Moderate ventral curvature of scapula (char. 94: 1 > 0, reversed in *Tigrisoma*; also found in *Phaethon, Gavia, Phoebastria*, and most birds outside of Gruiformes, *Opisthocomus*, and some Strisores and Telluraves); S24.

Both *Limnofregata* (Olson 1977) and *Rhynchaeites* (Mayr and Kitchener 2023c) exhibit an acromion process that extends cranial to the coracoid tubercle on the scapula, though this is expressed to a lesser extent in the former compared to most extant members of Pelecanes other than herons (Ardeidae) (Olson 1977). Previously proposed morphological synapomorphies for Pelecanes concern features of the hindlimbs (Sangster et al. 2022).

#### Suliformes

Six character states were optimized as potential synapomorphies of Suliformes, of which five characters were consistently inferred as such across all alternative molecular topologies studied.

**Fig. 31:**
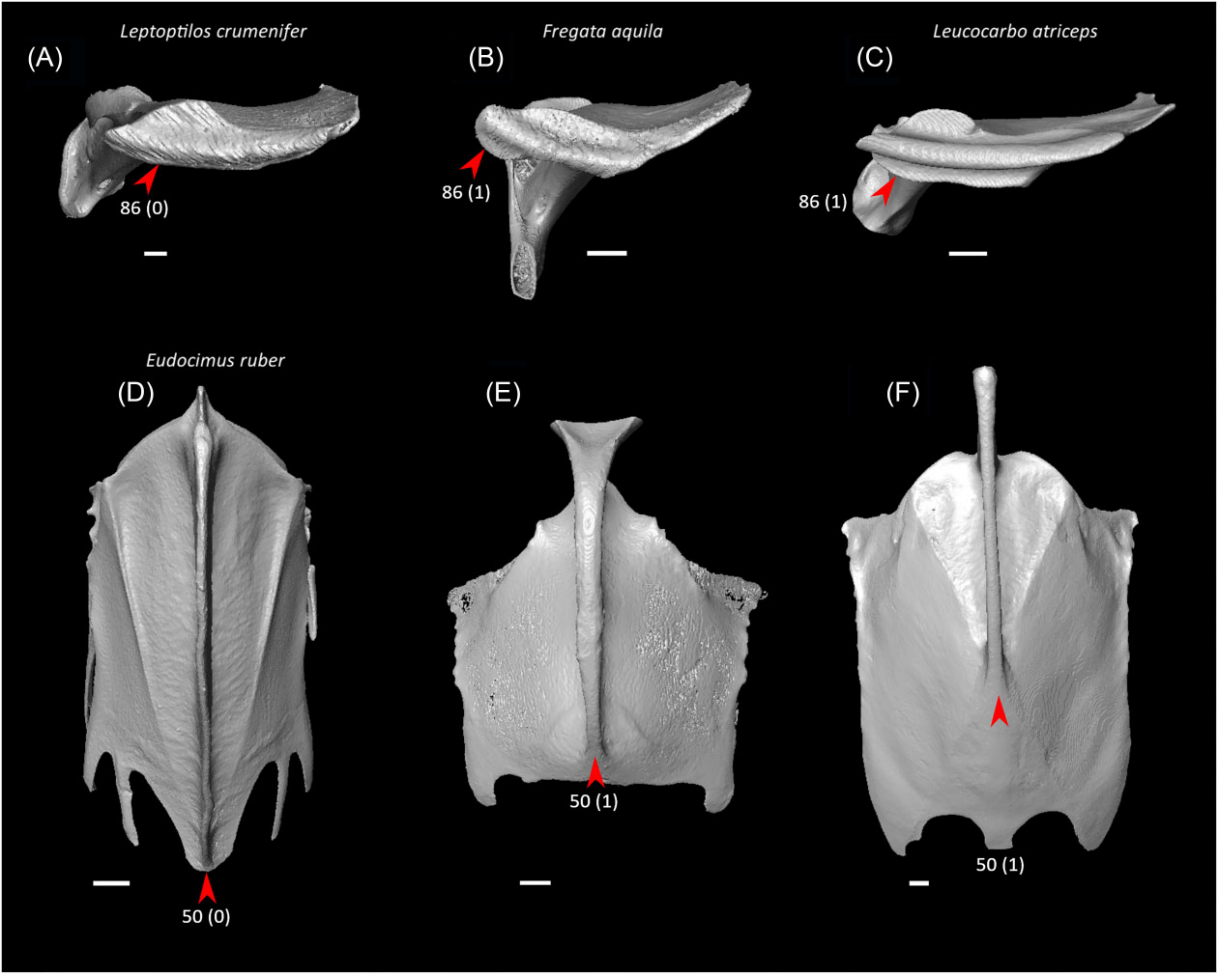
Coracoids of *Leptoptilos crumenifer* (**A**, NHMUK S1952.3.182, left element, mirrored), *Fregata aquila* (**B**, NHMUK 1890.11.3.3, right element), and *Leucocarbo atriceps* (**C**, NHMUK S2012.36, right element) in sternal view and sterna of *Eudocimus ruber* (**D**, NHMUK S1999.8.1), *F. aquila* (**E**, NHMUK 1890.11.3.3), and *L. atriceps* (**F**, NHMUK S2012.36) in ventral view. Scale bars = 5 mm. The furcula of *F. aquila*, which is fused to the sternum and coracoid, has been digitally removed. Arrows indicate the external lip of the coracoid (**A–C**) and the caudal termination of the sternal keel (**D–F**). *Leptoptilos* exhibits state 0 for character 86 (little ventral expansion of external lip of coracoid), whereas *Fregata* and *Leucocarbo* exhibit state 1 (prominent ventral expansion of external lip of coracoid, optimized as a synapomorphy of Suliformes). *Eudocimus* exhibits state 0 for character 50 (sternal keel extending to caudal terminus of median trabecula), whereas *Fregata* and *Leucocarbo* exhibit state 1 (sternal keel ending cranial to caudal terminus of median trabecula, optimized as a synapomorphy of Suliformes).

- Sternal keel ends caudally before termination of median trabecula (Fig. 31, char. 50: 0 > 1, also found in *Gansus*, Anseriformes, *Phoenicopterus, Pterocles, Corythaeola, Balearica, Alca, Phaethon, Gavia, Phoebastria, Pelecanus, Elanus, Psilopogon, Acanthisitta*, and *Menura*); J14, P15, K21, S24, W24.
- Median trabecula of sternum squared off at caudal termination (char. 61: 0 > 1, reversed in *Sula*; also found in *Phaethon, Tigrisoma*, and many non-phaethoquornithean birds); J14, P15, K21, S24, W24.
- Caudolateral trabeculae of sternum extend caudal to median trabecula (char. 62: 0 > 1, also found in *Gansus, Ichthyornis, Dendrocygna*, Mirandornithes, Strisores, Heliornithes, *Phaethon, Spheniscus, Phoebastria, Scopus, Pelecanus, Pandion* + *Elanus, Tyto*, Pici, *Micrastur*, and *Nestor*); J14, P15, K21, S24, W24.
- External lip of coracoid greatly expanded (Fig. 31, char. 86: 0 > 1, also found in Columbimorphae, *Ardeotis, Tapera*, Grues, *Rostratula, Alca, Sterna, Oceanites, Scopus, Pelecanus, Coragyps, Pandion, Leptosomus, Upupa*, Pici, and *Nestor*); J14, P15, K21, S24, W24.
- Transverse sulcus on humerus long (char. 115: 1 > 2, also found in *Limosa, Sterna*, and *Phaethon*); J14, P15, K21, S24, W24.
- Olecranon fossa on humerus deep (char. 127: 0 > 1, also found in Phaethontimorphae, Procellariimorphae, *Scopus* + *Pelecanus*, and most non-phaethoquornithean neognaths); W24.

Traditionally, members of Suliformes were thought to be closely related to pelicans (Pelecanidae) based on anatomical similarities (e.g., Cracraft 1985; Mayr 2003b; Livezey and Zusi 2007; Smith 2010), and indeed three of the above traits are also found in *Pelecanus*, emphasizing the morphological convergence between these two separate lineages within Pelecanes. *Phaethon*, which as discussed previously was also once hypothesized to form a clade with *Pelecanus* and members of Suliformes, shares four of these traits.

Of these characters, *Limnofregata* has been reported to possess caudolateral trabeculae that extend caudal to the median trabecula of the sternum and an expanded external lip on the coracoid (though to a lesser extent than in *Fregata*) (Olson 1977). Furthermore, though Olson (1977) considered the sternal keel of *Limnofregata* and *Fregata* to traverse the entire length of the sternum, a flattened region caudal to the keel is nonetheless visible on the ventral surface of the sternum in these taxa.

Of note is that the extinct clade Plotopteridae, a group of flightless marine neoavians that have been alternatively considered either members of Suliformes (Olson 1980; Smith 2010; Mayr et al. 2015, 2021, 2025) or closely related to spheniscids (Mayr 2005b; Mayr et al. 2015), exhibit at least two of the potential suliform synapomorphies listed above that are absent in spheniscids, namely a sternal keel that does not extend to the caudal termination of the median trabecula (Olson 1980) and a greatly expanded external lip of the coracoid (Mayr et al. 2021). In addition, plotopterids possess an elongated acromion process of the scapula (Olson 1980; Ando and Fukata 2018; Mayr et al. 2021), here optimized as a synapomorphy of Pelecanes (though Mayr et al. 2021 reported that some stem-spheniscids display an enlarged acromion compared to that of extant spheniscids, the acromion in these specimens still does not extend as far cranially as it does in most members of Pelecanes besides ardeids). These observations may therefore add to the growing support for a placement of plotopterids within Suliformes and further suggest that their similarities to total-group spheniscids represent a remarkable example of convergent evolution driven by specialization toward a wing-propelled diving lifestyle (Olson 1980; Smith 2010; Mayr et al. 2021).

Smith (2010) additionally noted a distinct proximal ridge extending from the ventral supracondylar tubercle on the humerus as well as major and minor metacarpals being subequal in length as potential synapomorphies of Suliformes. However, the latter character state (char. 193: 0) is recovered here as plesiomorphic for neognaths. Mayr (2011c) recovered the absence of a supracoracoid nerve foramen as a suliform synapomorphy, but this character state (char. 72: 0) is inferred to be plesiomorphic for crown birds in this study. Using the phylogenetic matrix of Livezey and Zusi (2006), Ericson and Qu (2025) inferred a caudally inflated flexor process on the humerus and the presence of an interclavicle dorsal process on the furcula (char. 7: 1) as potential synapomorphies of Suliformes, though the latter character state was not optimized as such in the present study as it was only observed in *Leucocarbo* among the suliform taxa sampled here.

#### Pelecaniformes

Two character states were optimized as potential synapomorphies of Pelecaniformes, both of which were consistently inferred as such across all alternative molecular topologies studied.

- Coracoid sulci on sternum crossed (char. 25: 1 > 2, reversed in *Pelecanus*; also found in *Ichthyornis, Phoenicopterus, Corythaeola*, Grues, *Burhinus*, Phaethontimorphae, Procellariimorphae, *Elanus, Leptosomus*, and *Micrastur*); J14, P15, K21, S24, W24.
- Procoracoid process of coracoid moderately prominent (char. 70: 2 > 1, also found in Anseres, *Phoenicopterus, Ardeotis, Nyctibius, Streptoprocne, Sarothrura, Rallus, Eurypyga, Gavia, Phoebastria*, Accipitrimorphae, *Trogon*, and *Nestor*); J14, P15, K21, S24, W24.

However, *Rhynchaeites* exhibits a more elongate procoracoid process than extant threskiornithids do, which is here optimized as a plesiomorphic trait for Phaethoquornithes, and lacks crossed coracoid sulci on the sternum (Mayr and Kitchener 2023c). Similarly, the putative stem-threskiornithid *Vadaravis* has an elongated procoracoid process, though conversely crossed coracoid sulci are present in this taxon (Smith et al. 2013). Also observable in *Vadaravis* are a foramen in the median sulcus of the sternum near its cranial margin and a tubercle at the insertion point of humerocarpal ligament on the pisiform (Smith et al. 2013), suggesting that a phylogenetic placement of this genus within Pelecanimorphae is plausible. Unlike most extant members of Pelecanes other than ardeids, however, *Vadaravis* has a short acromion process of the scapula (Smith et al. 2013).

#### Telluraves

Three character states were optimized as potential synapomorphies of Telluraves, but none of these were consistently inferred as such across all alternative molecular topologies studied.

**Fig. 32:**
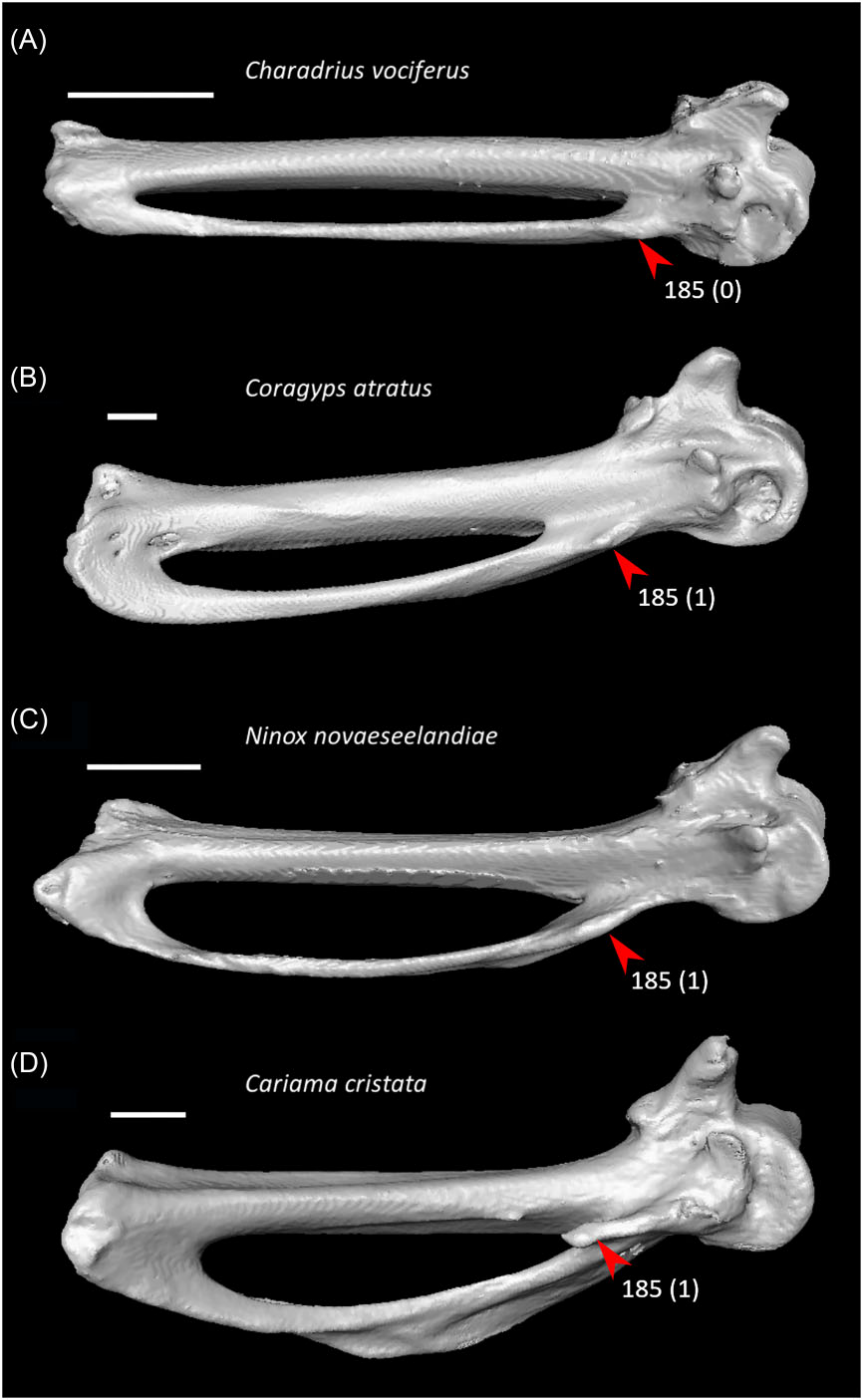
Carpometacarpi of *Charadrius vociferus* (**A**, FMNH 470173, left element, mirrored), *Coragyps atratus* (**B**, UMMZ 71891, right element), *Ninox novaeseelandiae* (**C**, UMZC 497.B, left element, mirrored), and *Cariama cristata* (**D**, FMNH 105653, right element) in ventral view. Scale bars = 5 mm. Arrows indicate the tubercle at the base of the minor metacarpal. *Charadrius* exhibits state 0 for character 185 (tubercle on minor metacarpal small or absent), whereas *Coragyps, Ninox*, and *Cariama* exhibit state 1 (tubercle on minor metacarpal prominent, optimized as a synapomorphy of Telluraves).

- Protruding articular facets for acrocoracoids on furcula prominent (char. 3: 0 > 1, reversed in *Tyto, Ninox* [though present in most other Strigidae, Mayr 2005c; Mayr and Kitchener 2023d], *Trogon, Upupa, Coracias*, and *Nestor*; also found in *Columba, Corythaeola, Nyctibius*, Daedalornithes, *Alca, Sterna, Spheniscus, Oceanites*, Suloidea, *Scopus*, and *Pelecanus*); J14, K21, S24, W24.
- Keel low, less than two-thirds total height of sternum (char. 49: 1 > 0, reversed in *Upupa*, Psittaciformes, and *Menura*; also found in *Chauna, Anseranas*, Mirandornithes, *Tapera*, Vanescaves, *Opisthocomus, Psophia*, Charadrii, Limosa, and Phaethoquornithes), W24.
- Prominent tubercle on minor metacarpal distal to proximal synostosis of metacarpals (Fig. 32, char. 185: 0 > 1, reversed in *Pandion, Urocolius*, Eucavitaves [regained in total-group Coracioidea, Mayr and Mourer-Chauviré 2000; Mayr et al. 2004; Bourdon et al. 2016; Mayr 2022b], and Psittacopasseres; also found in *Ichthyornis, Eudromia, Rollulus, Psophia, Burhinus, Turnix, Sterna*, and *Phaethon*); J14, P15, K21.

Probably owing to the extensive morphological disparity of this clade, few anatomical synapomorphies of Telluraves have previously been identified (Mayr 2011a; Sangster et al. 2022). Even so, potential support for some of the proposed synapomorphies above may be found in the fossil record. The presence of a prominent ventral tubercle on the minor metacarpal has been documented in the stem-mousebird (stem-Coliidae) Sandcoleidae (Houde and Olson 1992), the putative stem-courol (stem-Leptosomidae) *Waltonavis* (Mayr and Kitchener 2023e), and many stem-seriemas (stem-Cariamidae) (Mourer-Chauviré 1983; Mayr 2016b).

Prominent articular facets for the acrocoracoid are present on the furcula of the stem-passeriform (Mayr 2004c, 2008b, 2015b; Ksepka et al. 2019; Mayr and Kitchener 2023e) clade Zygodactylidae (Mayr 1998b, 2008b; Mayr and Kitchener 2023f), but lacking in the stem-owl (stem-Strigiformes) *Ypresiglaux* (Mayr and Kitchener 2023d), sandcoleids (Houde and Olson 1992), *Waltonavis* and the stem-leptosomid *Plesiocathartes* (Mayr and Kitchener 2023e), the stem-falcon (stem-Falconidae) clade Masillaraptoridae (Mayr and Kitchener 2021), and the putative stem-psittacopasserean (Mayr 2015b; Ksepka et al. 2019; Mayr and Kitchener 2023f) clades Messelasturidae (Mayr 2005c; Mayr and Kitchener 2023f) and Halcyornithidae (Ksepka et al. 2011; Mayr and Kitchener 2024d), suggesting that this character state may have evolved multiple times within Telluraves.

#### Accipitrimorphae

Seven character states were optimized as potential synapomorphies of Accipitrimorphae, of which four were consistently inferred as such across all alternative molecular topologies studied.

- Acromial processes on furcula strongly caudally inflected (char. 6: 0 > 1, also found in *Chauna, Rollulus, Pterocles, Florisuga, Rostratula, Alca, Gavia, Spheniscus, Fregata, Leucocarbo, Pelecanus, Ninox, Urocolius, Upupa, Bucco*, and *Psilopogon*); P15.
- Median trabecula of sternum rounded at caudal termination (char. 61: 1 > 0, also found in non-neognath birds, *Ortalis* + *Rollulus, Monias, Columba, Florisuga*, Gruiformes, *Turnix, Alca, Eurypyga*, Aequornithes, *Tyto, Urocolius, Bucorvus, Bucco*, Psittaciformes, and *Menura*); P15.
- Procoracoid process of coracoid moderately prominent (char. 70: 2 > 1, also found in Anseres, *Phoenicopterus, Ardeotis, Nyctibius, Streptoprocne, Sarothrura, Rallus, Eurypyga, Gavia, Phoebastria*, Pelecaniformes, *Trogon*, and *Nestor*); J14, P15, K21, S24, W24.
- Supracoracoid nerve foramen in coracoid present (char. 72: 1 > 2, also found in *Ichthyornis, Anseranas, Phoenicopterus, Corythaeola*, Daedalornithes, Gruiformes, Charadrii, *Alca, Sterna, Phaethon*, Procellariiformes, *Leptoptilos, Eudocimus, Pelecanus*, Strigiformes, *Leptosomus*, and *Micrastur*); P15.
- Dorsal tubercle of humerus pointed (char. 139: 0 > 1, also found in *Dendrocygna*, Mirandornithes, *Podargus, Aegotheles, Balearica, Charadrius, Alca*, Procellariiformes, *Leptoptilos*, Suloidea, *Scopus* + *Pelecanus, Leptosomus, Trogon, Coracias, Micrastur, Psittacus*, and Eupasseres); J14, P15, K21, S24, W24.
- Radial incisure on ulna prominent (char. 155: 0 > 1, also found in *Ichthyornis, Eudromia, Chauna, Anseranas*, Mirandornithes, *Ardeotis, Nyctibius*, Charadriiformes, Grues, *Phaethon, Phoebastria, Pagodroma, Leptoptilos, Fregata, Sula, Scopus* + *Pelecanus*, and Psittaciformes); J14, P15, K21, S24, W24.
- Quill knobs on ulna strongly developed (char. 160: 0 > 1, also found in *Anseranas, Podilymbus, Monias, Columba, Corythaeola, Ardeotis, Tapera, Caprimulgus, Aramus, Charadrius*, Scolopaci + Lari, *Oceanites*, Pelecanimorphae, Picocoraciades, and Passeriformes); J14, P15, K21, S24, W24.

Using the phylogenetic matrix of Livezey and Zusi (2006), Ericson and Qu (2025) also inferred the presence of an impression for the m. coracobrachialis caudalis placed within an impression for the m. supracoracoideus on the sternum as a potential synapomorphy of this clade. Few fossils that bear on the ancestral morphology of Accipitrimorphae have been described, though *Horusornis*, which may have close affinities to this clade, exhibits a moderately prominent procoracoid process and a supracoracoid nerve foramen in the coracoid (Mourer-Chauviré 1991). These features are also found in the Oligocene hawk (Accipitridae) *Archaehierax*, which additionally exhibits a prominent radial incisure on the ulna and ulnar quill knobs comparable to those of extant accipitrids (Mather et al. 2022).

#### Coraciimorphae

Seven character states were optimized as potential synapomorphies of Coraciimorphae, of which two were consistently inferred as such across all alternative molecular topologies studied.

**Fig. 33:**
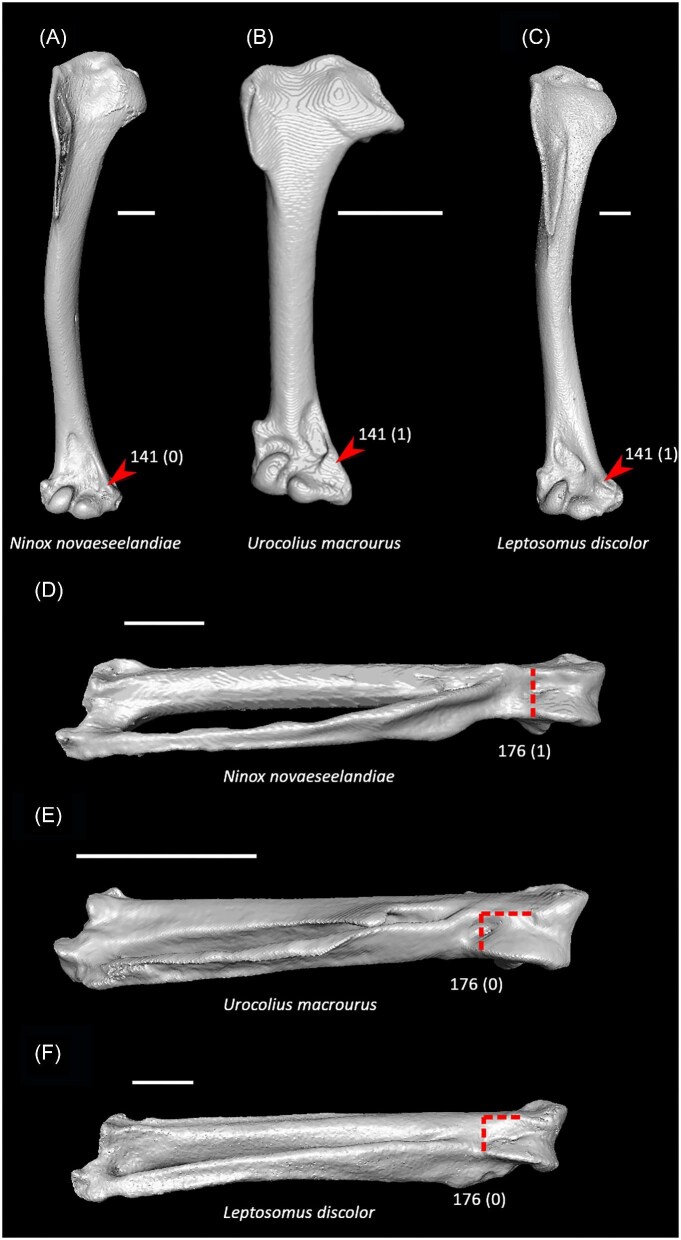
Humeri of *Ninox novaeseelandiae* (**A**, UMZC 497.B, right element), *Urocolius macrourus* (**B**, FMNH 368959, left element, mirrored), and *Leptosomus discolor* (**C**, NHMUK A1968.30.38, left element, mirrored) in cranial view and carpometacarpi of *N. novaeseelandiae* (**D**, UMZC 497.B, left element), *U. macrourus* (**E**, FMNH 368959, right element, mirrored), and *L. discolor* (**F**, NHMUK A1968.30.38, left element) in caudal view. Scale bars = 5 mm. Arrows indicate the ventral supracondylar tubercle (**A–C**) and dotted lines indicate the distal extent of the ventral rim of the carpal trochlea relative to that of the dorsal rim (**D–F**). *Ninox* exhibits state 0 for character 141 (ventral supracondylar tubercle moderately developed), whereas *Urocolius* and *Leptosomus* exhibit state 1 (ventral supracondylar tubercle expanded, forming large triangular platform approximately equal in width to ventral condyle and in dorsal extent to dorsal condyle, optimized as a synapomorphy of Coraciimorphae). *Ninox* also exhibits state 1 for character 176 (dorsal rim of carpal trochlea subequal in distal extent to that of ventral rim), whereas *Urocolius* and *Leptosomus* exhibit state 0 (dorsal rim of carpal trochlea falls considerably short of ventral rim in distal extent, optimized as a synapomorphy of Coraciimorphae).

- Ventromedial intermuscular line on sternum absent or weak (char. 39: 1 > 0, reversed in *Upupa, Coracias, Alcedo*, and Pici; also found in *Gansus, Ichthyornis, Chauna, Corythaeola, Ardeotis, Tapera*, Strisores, *Opisthocomus, Rallus, Turnix*, Phaethontimorphae, *Gavia, Pelecanus, Elanus, Ninox*, and Passeriformes); S24, W24.
- Cranial margin of sternal keel not recurved (char. 44: 1 > 0, reversed in *Trogon, Bucorvus, Alcedo*, and *Jynx*; also found in *Chauna, Dendrocygna, Monias, Corythaeola, Sarothrura, Scopus* + *Pelecanus, Pandion, Tyto*, and Psittaciformes); S24, W24.
- Longitudinal concavity of scapula prominent (char. 95: 0 > 1, reversed in Picocoraciades; also found in *Gansus, Anseranas*, Galliformes, Pteroclimesites, *Tapera, Opisthocomus, Aramus*, Heliornithes, *Eudocimus, Cariama, Micrastur*, and Tyranni); J14, K21.
- Ventral supracondylar tubercle on humerus expanded (Fig. 33, char. 141: 0 > 1, reversed in Picocoraciades; also found in *Ichthyornis, Chauna*, Galliformes, *Columba, Tapera, Limosa, Turnix, Gavia, Oceanites, Fregata, Leucocarbo*, and *Climacteris*); J14, P15, K21, S24, W24.
- Ventral collateral ligamental tubercle on ulna strongly developed (char. 157: 0 > 1, reversed in *Bucorvus* and *Merops*; also found in *Ichthyornis, Ortalis, Corythaeola, Tapera*, Strisores, *Psophia*, Charadriiformes, Procellariiformes, *Fregata, Pelecanus, Elanus*, Strigiformes, *Nestor*, and Passeriformes); W24.
- Distal extent of dorsal rim of carpal trochlea falls considerably short of ventral rim (Fig. 33, char. 176: 1 > 0, reversed in *Trogon*; also found in *Eudromia*, Galliformes, *Monias, Corythaeola, Psophia, Sarothrura, Rallus, Burhinus, Gavia, Spheniscus, Fregata, Cariama*, and Passeriformes); J14, P15, K21, S24, W24.
- Minor metacarpal projecting substantially distally beyond major metacarpal (char. 193: 0 > 1, reversed in *Coracias*; also found in *Eudromia, Alectura, Ortalis, Monias, Corythaeola, Caprimulgus, Florisuga, Psophia, Spheniscus, Elanus, Ninox, Micrastur*, and Passeriformes); S24, W24.

The status of these character states is largely unclear in fossil specimens that bear on the ancestral morphology of Coraciimorphae, such as *Plesiocathartes* (Mayr 2002c; Weidig 2006; Mayr and Kitchener 2023e). However, an expanded ventral supracondylar tubercle on the humerus is present in sandcoleids (Houde and Olson 1992; Mayr and Peters 1998; Ksepka et al. 2017), *Waltonavis* (Fig. 9g in Mayr and Kitchener 2023e), and stem-trogons (stem-Trogonidae) (Mayr et al. 2023b).

#### Cavitaves

Six character states were optimized as potential synapomorphies of Cavitaves, but none of these were consistently inferred as such across all alternative molecular topologies studied.

- Keel apex on sternum cranial to main body of sternum (char. 43: 0 > 1, reversed in *Bucco*; also found in *Cariama*, Psittaciformes, Eupasseres, and many non-telluravian birds); J14, P15, K21.
- Acrocoracoid process on coracoid forms pronounced hook (char. 65: 0 > 1, reversed in *Psilopogon*; also found in *Ichthyornis, Eudromia, Alectura, Rollulus, Podilymbus, Pterocles, Columba, Corythaeola, Tapera, Aegotheles, Streptoprocne*, Heliornithes, Charadriiformes, Phaethontimorphae, Procellariimorphae, *Cariama*, Psittaciformes, and Eupasseres); J14, P15, K21.
- Deltopectoral crest well-developed, extending at least one third the total length of the humerus (char. 122: 1 > 0, reversed in Coraciiformes; also found in *Gansus, Ichthyornis, Corythaeola, Ardeotis, Nyctibius, Podargus, Spheniscus, Leptoptilos, Leucocarbo, Eudocimus, Scopus*, Accipitrimorphae, Strigiformes, *Micrastur*, and Passeri); W24.
- Humerus narrowest near midshaft point (char. 125: 0 > 2, reversed in *Merops, Alcedo*, and *Bucco*; also found in *Ichthyornis, Alectura, Rollulus, Pterocles, Columba, Corythaeola, Tapera*, Strisores, *Opisthocomus, Podica*, Charadrii, *Rostratula, Sterna, Eurypyga, Spheniscus, Oceanites, Leptoptilos, Pelecanus* + *Tigrisoma, Pandion, Cariama*, Psittaciformes, and Eupasseres); J14, P15, K21.
- Radius craniocaudally curved (char. 144: 0 > 1, reversed in *Coracias*; also found in Anseriformes, *Phoenicopterus, Ardeotis*, Strisores, Grues, Charadrii, Feraequornithes, *Coragyps, Pandion*, Strigiformes, *Micrastur*, Tyranni, and *Climacteris*); W24.
- Ventral interosseal sulcus on carpometacarpus forms prominent groove (char. 195: 0 > 1, reversed in *Bucorvus, Coracias*, and *Alcedo*; also found in *Sarothrura, Gavia, Fregata, Scopus*, Strigiformes, *Psittacus, Erythropitta*, and Passeri); S24, W24.

A hooked acrocoracoid process on the coracoid has been reported in *Waltonavis* (Mayr and Kitchener 2023e), *Plesiocathartes* (Mayr 2002c), and stem-trogonids (Mayr 1999b, 2009; Mayr et al. 2023b). This character state is also found in *Ypresiglaux* (Mayr and Kitchener 2023d) and sandcoleids (Houde and Olson 1992; Ksepka et al. 2017), however, which suggests that it could be a synapomorphy of a more inclusive clade. *Waltonavis* additionally has a narrow humeral midshaft (Fig. 9g in Mayr and Kitchener 2023e), but the remaining characters listed above are otherwise difficult to assess in relevant fossil taxa.

Although not optimized as an unambiguous synapomorphy of Cavitaves, a bifurcated acromion process of the scapula (char. 90: 1) may also be synapomorphic for this group, as it is found in all cavitavians sampled in the present study except for *Leptosomus, Bucorvus*, and *Bucco*. A slightly bifurcated acromion process has been reported in *Waltonavis* (Mayr and Kitchener 2023e); the stem-trogonid *Eotrogon* (Mayr et al. 2023b); a potential stem member of the hoopoe + woodhoopoe clade (Upupides), *Messelirrisor* (Mayr 2000c); stem members of the roller + ground roller clade (Coracioidea), *Septencoracias* (Mayr and Kitchener 2024e; *contra*  Bourdon et al. 2016) and *Geranopterus* (Mayr and Mourer-Chauviré 2000); the putative stem-piciform *Gracilitarsus* (Mayr 2005d); and the stem-Pici taxon *Rupelramphastoides* (Mayr 2005e). However, this character is also present in *Ypresiglaux* (Mayr and Kitchener 2023d) and the stem-coliid *Ypresicolius* (Mayr and Kitchener 2024f). If Strigiformes and Coraciimorphae are extant sister taxa, as recovered by Jarvis et al. (2014), Prum et al. (2015), and Kuhl et al. (2021), this may hint at multiple evolutionary acquisitions and losses of this feature within the clade uniting both.

#### Eucavitaves

Five character states were optimized as potential synapomorphies of Eucavitaves, of which four were consistently inferred as such across all alternative molecular topologies studied.

- Notch in medial margin of sternal end of coracoid (char. 77: 0 > 1, reversed in *Bucorvus* and *Merops*; also found in *Rollulus, Tapera, Podica, Burhinus, Alca, Spheniscus*, and Passeriformes); J14, P15, K21, S24, W24.
- Deltopectoral crest rounded dorsally (char. 121: 1 > 0, reversed in *Upupa, Merops*, and *Bucco*; also found in *Gansus, Chauna, Anseranas*, Mirandornithes, *Ardeotis, Aegotheles, Opisthocomus*, Gruiformes, *Charadrius, Rostratula, Alca, Eudocimus*, and Australaves); J14, P15, K21, S24, W24.
- Dorsal supracondylar process on humerus extremely small (char. 136: 1 > 0, reversed in *Upupa, Coracias*, and *Jynx*; also found in *Podilymbus, Aegotheles, Opisthocomus, Balearica, Spheniscus, Sula*, and Psittaciformes); J14, P15, K21, S24, W24.
- Dorsal epicondyle on humerus prominent (char. 138: 0 > 1, reversed in *Coracias*; also found in *Dendrocygna*, Galliformes, Daedalornithes, *Psophia, Phaethon, Eudocimus, Pelecanus*, and Passeriformes); J14, P15, K21, S24, W24.
- Ventral aponeurosis tubercle on radius pointed (char. 148: 0 > 1, reversed in *Merops* and Piciformes; also found in *Coragyps, Pandion, Ninox, Psittacus, Climacteris*, and many non-telluravian birds); P15.

A notched medial margin on the sternal end of the coracoid was previously considered a synapomorphy of Piciformes by Mayr et al. (2003), though as noted by Mayr (2005d), this character also occurs in other birds, including many taxa now recognized as members of Eucavitaves. Among fossil eucavitavians, it has been reported in *Eotrogon* (Mayr et al. 2023b), putative stem-upupideans (Mayr 2000c, 2006b; Mayr et al. 2020b), *Gracilitarsus* (Mayr 2005d), the putative stem-piciform (Duhamel et al. 2020; Mayr 2022a) *Pristineanis kistneri* (Houde and Olson 1989), and *Rupelramphastoides* (Mayr 2005e). However, this feature is not as pronounced in stem-trogonids and stem-upupideans as it is in their respective crown groups (Mayr 2006b; Mayr et al. 2020b, 2023b), and it appears to be absent in the stem-trogonid *Primotrogon* (Mayr 1999b).

A rounded deltopectoral crest is visible on the humerus of *Eotrogon*, though the same element appears to exhibit a more prominent dorsal supracondylar process and a less prominent dorsal epicondyle than found in crown-group trogonids (Fig. 5 in Mayr et al. 2023b). *Messelirrisor* has a prominent dorsal epicondyle on the humerus (Mayr 2000c).

#### Picocoraciades

Ten character states were optimized as potential synapomorphies of Picocoraciades, of which eight were consistently inferred as such across all alternative molecular topologies studied.

- Pneumatic foramen in median sulcus of sternum immediately caudal to cranial margin (char. 29: 0 > 1, reversed in Pici; also found in *Ichthyornis, Dendrocygna, Alectura, Ortalis, Phoenicopterus, Pterocles, Ardeotis, Tapera*, Strisores, *Opisthocomus*, Grues, *Podica, Phaethon*, Pelecanimorphae, *Coragyps*, Strigiformes, *Micrastur*, Psittaciformes, *Neopelma*, and *Menura*); J14, P15, K21, S24, W24.
- Internal lip of coracoid expanded in lateral portion (char. 89: 0 > 2, reversed in *Coracias* and *Psilopogon*; also found in *Aegotheles* and *Oceanites*); J14, S24, W24.
- Longitudinal concavity of scapula absent or weak (char. 95: 1 > 0, reversed in Pici; also found in Accipitrimorphae, Strigiformes, Psittacopasseres, and many non-telluravian birds); J14, P15, K21, S24, W24.
- Cranial surface of bicipital crest planar or slightly concave (char. 118: 0 > 1, reversed in *Bucco*; also found in *Turnix, Phoebastria, Urocolius, Psittacus, Acanthisitta*, and Tyranni); J14, P15, K21, S24, W24.
- Ventral supracondylar tubercle on humerus moderately developed (char. 141: 1 > 0, reversed in Pici; also found in many non-coraciimorph birds); J14, P15, K21, S24, W24.
- Olecranon on ulna narrow and sharply projected (char. 158: 0 > 1, also found in *Chauna, Caprimulgus*, Apodiformes, *Alca, Sula*, and Passeriformes); J14, P15, K21, S24, W24.
- Quill knobs on ulna strongly developed (char. 160: 0 > 1, reversed in *Bucco*; also found in *Anseranas, Podilymbus, Monias, Columba, Corythaeola, Ardeotis, Tapera, Caprimulgus, Aramus, Charadrius*, Scolopaci + Lari, *Oceanites*, Pelecanimorphae, Accipitrimorphae, and Passeriformes); J14, P15, K21, S24, W24.
- Sulcus for tendon of m. extensor longus alulae on scapholunare well-defined (char. 163: 0 > 1); J14, P15, K21, S24, W24.
- Tendinal sulcus on carpometacarpus wraps around cranial surface of bone (char. 171: 0 > 1, reversed in *Bucco*; also found in *Nyctibius, Florisuga, Balearica, Coragyps, Elanus, Tyto, Micrastur*, and Passeriformes); S24, W24.
- Dorsal fossa on phalanx 1 of major digit indistinct (char. 202: 1 > 2, reversed in *Coracias* and *Merops*; also found in *Spheniscus, Pelecanus, Urocolius, Acanthisitta*, and *Climacteris*); J14, P15, K21, S24, W24.

Mayr (2014a) previously identified a well-defined sulcus for the tendon of m. extensor longus alulae on the scapholunare as a potential synapomorphy of this clade. The stem-upupideans *Waltonirrisor* (Mayr and Kitchener 2024e) and *Laurillardia* (Mayr et al. 2020b), the stem-coracioid *Eocoracias* (Mayr and Mourer-Chauviré 2000), the potential stem-piciform *Sylphornis* (Mourer-Chauviré 1988b), and *Rupelramphastoides* (Mayr 2005e) have a well-developed olecranon on the ulna, though this character is absent in the early coraciiform *Quasisyndactylus* (Mayr 1998b), *Gracilitarsus* (Mayr 2001), and *Jacamatia*, a putative stem-member of the jacamar + puffbird clade (Galbulae) (Duhamel et al. 2020). Quill knobs similar to those of extant Upupides have been reported in *Messelirrisor* (Mayr 2000c) and are markedly developed in *Rupelramphastoides* (Mayr 2005e, 2006c), but are poorly developed or absent in *Laurillardia* (Mayr et al. 2020b), the stem-coracioid *Primobucco* (Ksepka and Clarke 2010), and *Jacamatia* (Duhamel et al. 2020). *Primobucco* also lacks dorsal pneumatic foramina in the sternum (Ksepka and Clarke 2010).

#### Picodynastornithes

Two character states were optimized as potential synapomorphies of Picodynastornithes, both of which were consistently inferred as such across all alternative molecular topologies studied.

**Fig. 34:**
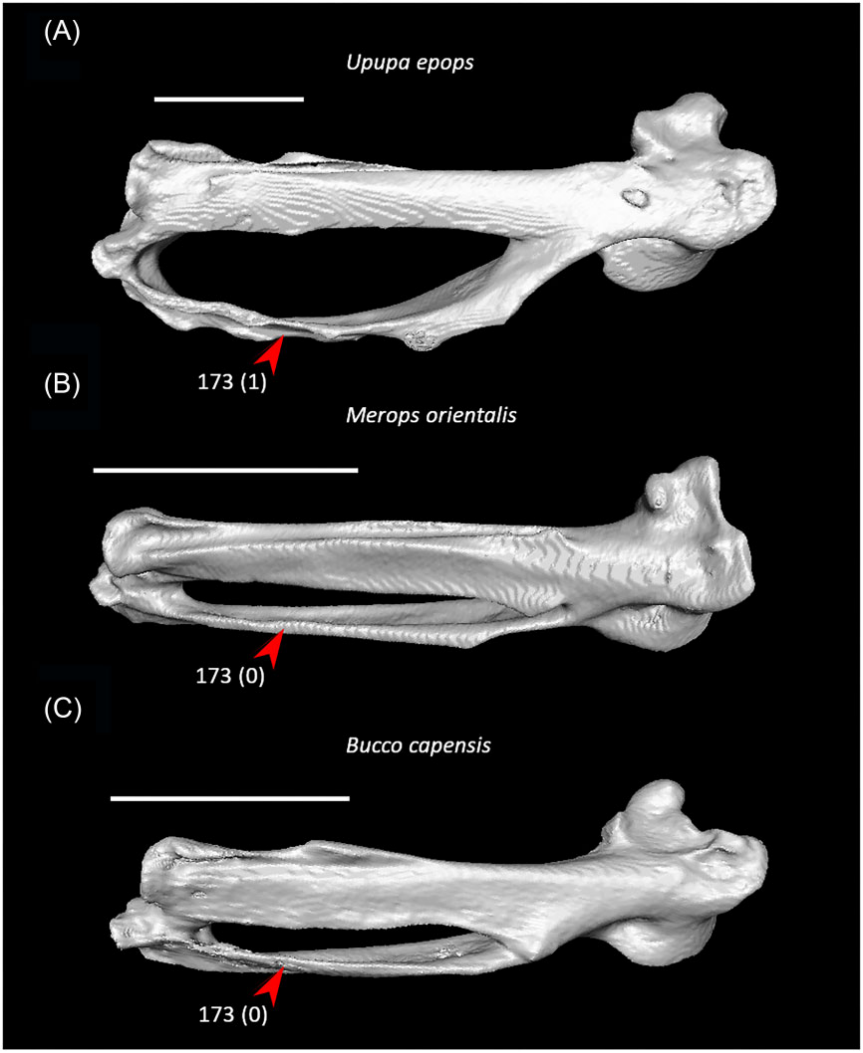
Carpometacarpi of *Upupa epops* (**A**, FMNH 352821, left element), *Merops orientalis* (**B**, UMMZ 216577, right element, mirrored), and *Bucco capensis* (**C**, FMNH 330305, left element) in dorsal view. Scale bars = 5 mm. Arrows indicate the minor metacarpal. *Upupa* exhibits state 1 for character 173 (minor metacarpal strongly bowed), whereas *Merops* and *Bucco* exhibit state 0 (minor metacarpal weakly bowed, optimized as a synapomorphy of Picodynastornithes).

- Minor metacarpal weakly bowed (Fig. 34, char. 173: 1 > 0, reversed in *Jynx*; also found in *Gansus, Ichthyornis, Dendrocygna*, Mirandornithes, Apodiformes, Charadriiformes, *Phaethon, Gavia*, Procellariimorphae, *Fregata, Eudocimus, Pandion*, and Psittacopasseres); J14, P15, K21, S24, W24.
- Intermetacarpal process on carpometacarpus well developed (char. 182: 0 > 1, reversed in *Merops*, also found in *Rollulus. Florisuga, Urocolius*, and Passeriformes); J14, P15, K21, S24, W24.

The minor metacarpal is weakly bowed in *Eocoracias* (Mayr and Mourer-Chauviré 2000), the stem-coracioid *Paracoracias* (Clarke et al. 2009), *Gracilitarsus* (Mayr 2001; Mayr 2005d), and *Sylphornis* (Duhamel et al. 2020), though it is more strongly bowed in *Septencoracias* (Mayr 2022b; Mayr and Kitchener 2024e) and especially so in *Jacamatia* (Duhamel et al. 2020). A well-developed intermetacarpal process on the carpometacarpus is also found in *Waltonirrisor* (Mayr and Kitchener 2024e) and *Messelirrisor* (Mayr 2006b) and may thus represent a synapomorphy of a more inclusive clade. This character appears to have been secondarily reacquired in Coracioidea, as it is absent in stem-coracioids (Mayr and Mourer-Chauviré 2000; Mayr et al. 2004; Clarke et al. 2009; Bourdon et al. 2016; Mayr 2022b; Mayr and Kitchener 2024e).

The above character states, along with some potential synapomorphies of several more inclusive clades like Picocoraciades, Eucavitaves, and Cavitaves, are convergently present in at least some members of Passeriformes, which may have contributed to some members of these groups being hypothesized as close relatives of passeriforms in previous morphological studies (e.g., Höfling and Alvarenga 2001; Livezey and Zusi 2007).

#### Australaves

Ten character states were optimized as potential synapomorphies of Australaves, of which three were consistently inferred as such across all alternative molecular topologies studied.

**Fig. 35:**
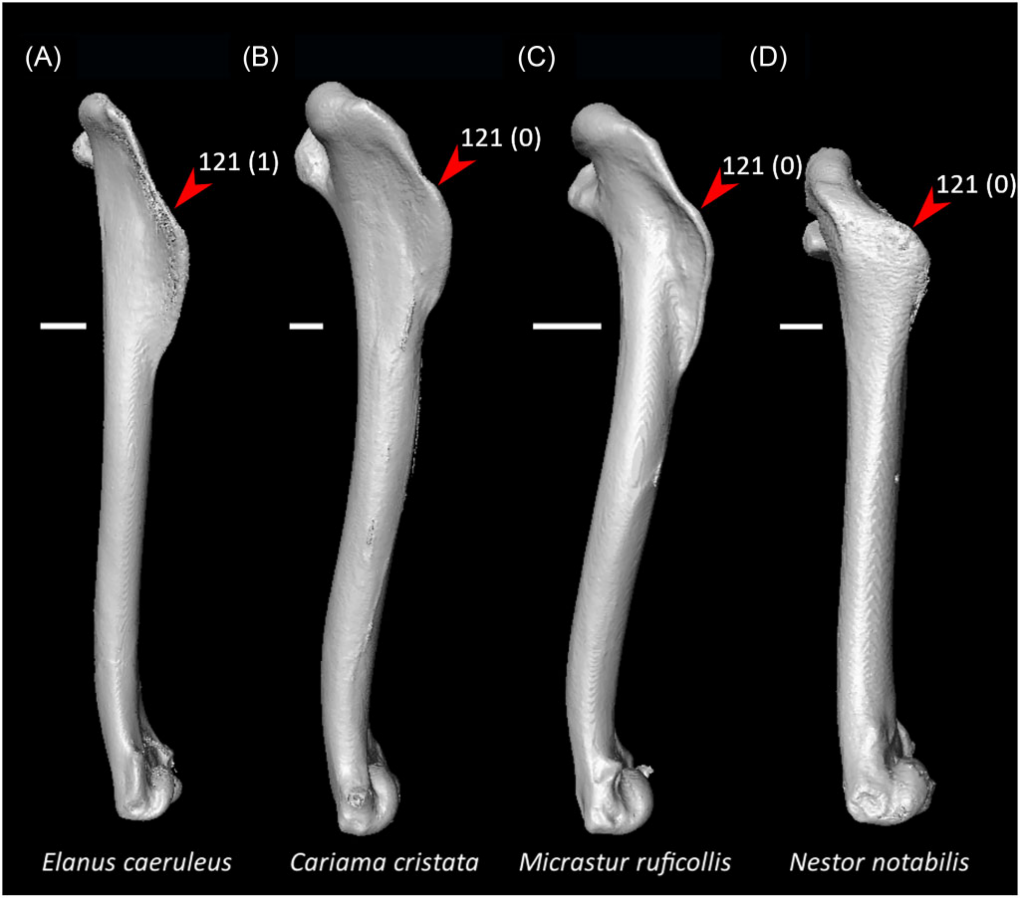
Humeri of *Elanus caeruleus* (**A**, NHMUK 1850.8.15.159, right element), *Cariama cristata* (**B**, FMNH 105653, right element), *Micrastur ruficollis* (**C**, FMNH 330226, right element), and *Nestor notabilis* (**D**, FMNH 23530, right element) in dorsal view. Scale bars = 5 mm. Arrows indicate the proximal dorsal margin of the deltopectoral crest. *Elanus* exhibits state 1 for character 121 (proximal dorsal margin of deltopectoral crest essentially straight), whereas *Cariama, Micrastur*, and *Nestor* exhibit state 0 (proximal dorsal margin of deltopectoral crest forming rounded bulge, optimized as a synapomorphy of Australaves).

- Pneumatic pores along cranial margin of dorsal sternum (char. 32: 0 > 1, reversed in Passeriformes; also found in Anseriformes, *Ortalis, Phoenicopterus, Monias, Columba, Ardeotis, Tapera, Caprimulgus*, Apodiformes, *Opisthocomus*, Gruoidea, *Podica, Phaethon, Leptoptilos, Sula, Eudocimus, Scopus, Pelecanus, Pandion*, Strigiformes, *Trogon, Coracias*, and *Merops*); K21, S24.
- Single pair of caudal fenestrae in sternum (char. 51: 2 > 1, reversed in *Acanthisitta*; also found in *Eudromia*, Anseres, Galliformes, Mirandornithes, *Monias, Caprimulgus, Balearica*, Ralloidea, *Turnix*, Aequornithes, *Pandion* + *Elanus, Tyto*, and Bucerotiformes); J14, S24.
- Pneumatic foramen in cranial end of scapula (char. 92: 0 > 1, reversed in *Nestor* and *Acanthisitta*; also found in *Chauna, Anseranas, Corythaeola, Florisuga, Opisthocomus*, Gruoidea, *Sula, Ninox*, Bucerotiformes, *Coracias*, and *Merops*); J14, P15, K21, S24, W24.
- Deltopectoral crest rounded dorsally (Fig. 35, char. 121: 1 > 0, reversed in *Psittacus* and *Climacteris*; also found in *Gansus, Chauna, Anseranas*, Mirandornithes, *Ardeotis, Aegotheles, Opisthocomus*, Gruiformes, *Charadrius, Rostratula, Alca, Eudocimus*, and Eucavitaves); J14, P15, K21, S24, W24.
- Tendinal sulcus of m. scapulotricipitalis on humerus shallow (char. 129: 1 > 0, reversed in Psittaciformes and *Neopelma*; also found in Galloanserae, *Phoenicopterus, Monias, Corythaeola, Opisthocomus, Psophia, Podica, Rallus, Eurypyga, Gavia, Leptoptilos, Elanus, Leptosomus, Bucorvus*, and Picodynastornithes); P15.
- Flexor process on humerus strongly developed (char. 131: 0 > 1, reversed in Psittaciformes; also found in *Monias, Corythaeola, Tapera*, Heliornithes, *Gavia, Spheniscus, Urocolius*, Bucerotiformes, *Alcedo*, and *Jynx*); J14, P15, K21, S24, W24.
- Scar of m. flexor carpi ulnaris on flexor process of humerus present as two scars (char. 142: 1 > 2, reversed in *Acanthisitta* and *Erythropitta*; also found in *Pandion, Leptosomus, Psilopogon*, and many non-telluravian birds); P15.
- Caudal scar for m. flexor carpi ulnaris on flexor process of humerus shallower than cranial scar (char. 143: 0 > 1, reversed in Psittaciformes; also found in *Chauna, Anseranas, Podilymbus, Ardeotis, Podargus, Aegotheles, Balearica, Burhinus, Alca, Sterna, Gavia, Pagodroma, Sula, Eudocimus, Tigrisoma*, and *Leptosomus*); J14, K21, S24, W24.
- Ventral ramus of pisiform subequal in cranial extent to dorsal ramus (char. 165: 0 > 1, reversed in *Psittacus* and *Climacteris*; also found in *Anseranas*, Galliformes, *Phoenicopterus, Tapera, Opisthocomus, Gavia, Oceanites* + *Pagodroma, Leptoptilos, Eudocimus, Ninox, Urocolius, Upupa*, and Picodynastornithes); J14, K21, S24.
- Internal index on phalanx 1 of major digit poorly developed (char. 200: 1 > 0, reversed in Psittaciformes and Tyranni; also found in *Gansus, Eudromia, Chauna, Alectura, Rollulus, Podilymbus, Monias, Corythaeola, Opisthocomus*, Gruiformes, *Rostratula, Turnix, Spheniscus, Tigrisoma, Ninox, Urocolius, Bucorvus, Merops*, and Piciformes); J14, K21.

A proximodorsally rounded deltopectoral crest has been reported in masillaraptorids (Mayr and Kitchener 2021), messelasturids (Mayr 2000d, 2011d; Mayr and Kitchener 2023f), halcyornithids (Mayr 1998c; Fig. 4 in Ksepka et al. 2011; Fig. 3 in Mayr and Kitchener 2024d), the potentially stem-passeriform (Mayr 2015b; Ksepka et al. 2019; Mayr and Kitchener 2023f; Mayr and Kitchener 2023g; Ksepka et al. 2025) clades Psittacopedidae (Mayr 2020) and Morsoravidae (Mayr 1999c; Ksepka et al. 2025), and zygodactylids (Mayr and Kitchener 2023g). Other character states in the above list have a more variable distribution among fossil taxa with proposed australavian affinities. A shallow scapulotricipital sulcus on the humerus is found in messelasturids (Mayr 2000d, 2005c, 2011d), halcyornithids (Mayr 2002d; Ksepka et al. 2011), and morsoravids (Mayr 1999c; Mayr and Kitchener 2023h), but this sulcus is deep in the stem-cariamid *Bathornis* (Mayr 2016b) and the putative psittacopedid *Eofringillirostrum* (Ksepka et al. 2019). A well-developed flexor process on the humerus is seen in psittacopedids (Mayr and Daniels 1998; Mayr 2020), morsoravids (Mayr 1999c; Mayr and Kitchener 2023h; Ksepka et al. 2025), and zygodactylids (Mayr 1998b; Mayr 2008b; Smith et al. 2018; Mayr and Kitchener 2023g), and a shorter but still distinct process is present in stem-cariamids (Mayr 2016b), *Masillaraptor* (Mayr and Kitchener 2021), and halcyornithids (Mayr 2002d; Ksepka et al. 2011). In contrast, the humeral flexor process is short in the masillaraptorid *Danielsraptor* (Mayr and Kitchener 2021), the putative stem-psittacopasserean (Mayr and Kitchener 2023f) *Vastanavis* (Mayr et al. 2013), and messelasturids (Mayr 2000d, 2005c, 2011d; Mayr and Kitchener 2023f), like in extant parrots (Psittaciformes). A poorly developed internal index process on phalanx 1 of the major digit has been found in halcyornithids (Mayr 2002d; Ksepka et al. 2011), psittacopedids (Mayr and Daniels 1998; Fig. 5 in Mayr 2020; Fig 5 in Mayr and Kitchener 2023g), morsoravids (Mayr 1999c), and zygodactylids (Mayr and Kitchener 2023g), whereas this structure is more strongly developed in *Masillaraptor* (Mayr and Kitchener 2021) and *Vastanavis* (Fig. 1o in Mayr et al. 2013).

The presence of a pneumatic foramen in the cranial end of the scapula is difficult to confirm from fossil material, but this foramen is absent in *Bathornis* (Mayr 2016b) and apparently in the halcyornithid *Cyrilavis* (Ksepka et al. 2011). Contrary to the condition in most extant members of the clade, many fossil australavians have two pairs of sternal incisions, as seen in masillaraptorids (Mayr and Kitchener 2021), messelasturids (Mayr 2021c), halcyornithids (Mayr 1998c, 2000e, 2007b; Ksepka et al. 2011; Mayr and Kitchener 2024d), psittacopedids (Mayr and Daniels 1998), morsoravids (Mayr 1999c; Ksepka et al. 2025), and zygodactylids (Mayr 1998b; Weidig 2010; Smith et al. 2018; Hieronymus et al. 2019).

As has been noted by previous authors, the putative stem-cariamid *Elaphrocnemus* exhibits several features atypical of total-group cariamids (Mourer-Chauviré 1983; Mayr 2016b), such as a deltopectoral crest with an essentially straight proximodorsal margin. Together with the absence of a tubercle on the ventral surface of the minor metacarpal (which was optimized as a potential synapomorphy of Telluraves in the present study), these features may hint at a placement of this taxon outside total-group Cariamidae, or even Telluraves, with closer affinities to *Opisthocomus* suggested by Mayr (2016b).

#### Eufalconimorphae

No unambiguous synapomorphies were optimized for Eufalconimorphae in this study.

#### Psittacopasseres

Six character states were optimized as potential synapomorphies of Psittacopasseres, of which four were consistently inferred as such across all alternative molecular topologies studied.

**Fig. 36:**
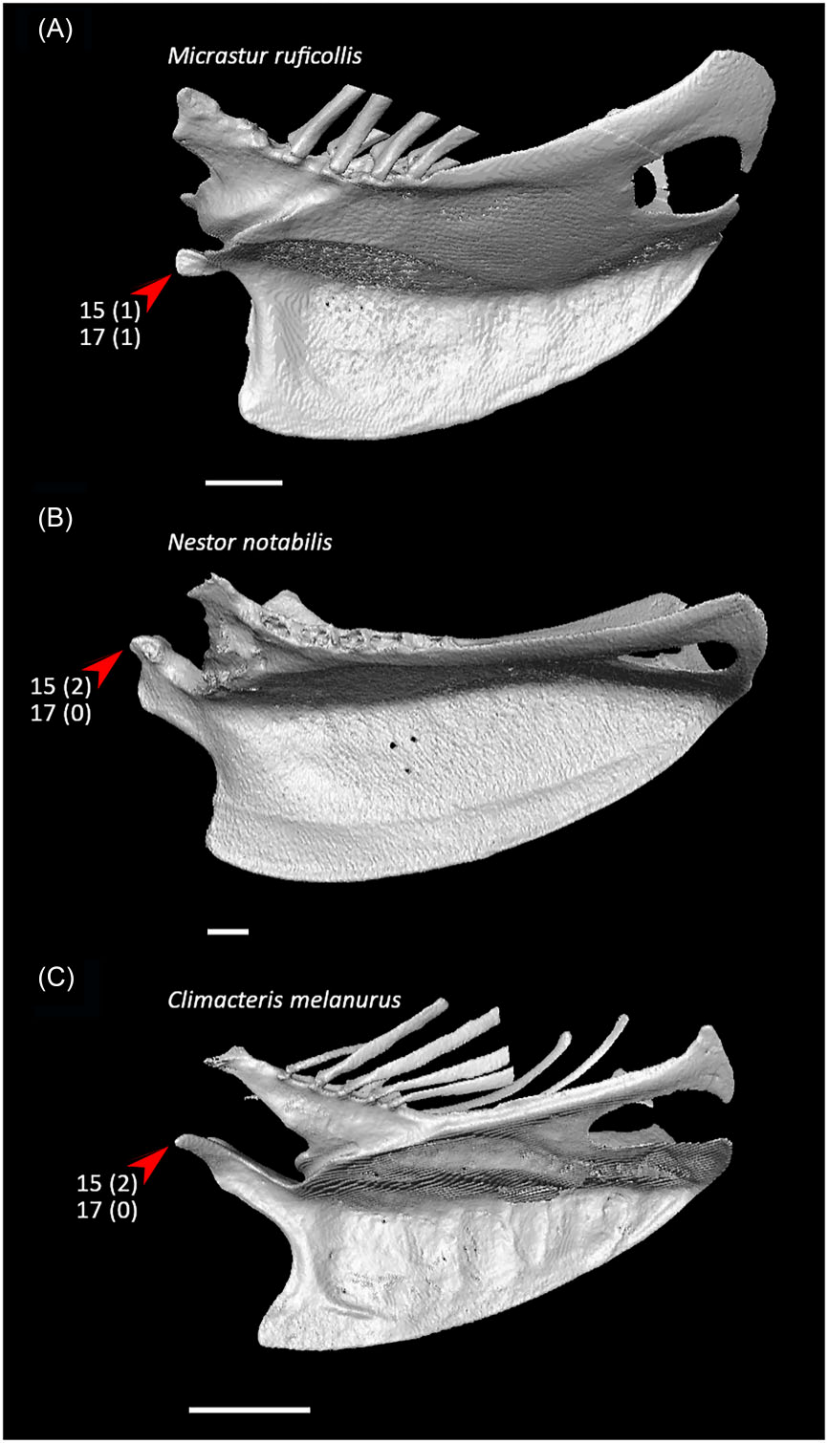
Sterna of *Micrastur ruficollis* (**A**, FMNH 330226), *Nestor notabilis* (**B**, FMNH 23530), and *Climacteris melanurus* (**C**, UMMZ 214303) in left (**A, C**) or right (**B**, mirrored) lateral view. Scale bars = 5 mm. Arrows indicate the external spine. *Micrastur* exhibits state 1 for character 15 (external spine 5–10% of total sternum length), whereas *Nestor* and *Climacteris* exhibit state 2 (external spine 10–20% of total sternum length, optimized as a synapomorphy of Psittacopasseres). *Micrastur* also exhibits state 1 for character 17 (external spine cranially oriented), whereas *Nestor* and *Climacteris* exhibit state 0 (external spine dorsally oriented, optimized as a synapomorphy of Psittacopasseres).

- External spine on sternum 10–20% of total sternum length (Fig. 36, char. 15: 1 > 2, also found in *Ortalis* + *Rollulus, Phoenicopterus, Corythaeola, Tapera, Turnix, Oceanites*, and Eucavitaves); J14, P15, K21, S24, W24.
- External spine of sternum dorsally oriented (Fig. 36, char. 17: 1 > 0, also found in *Monias, Columba*, Charadrii, *Turnix, Alca, Gavia, Ninox, Urocolius, Trogon*, Coraciiformes, and Pici); J14, P15, K21, S24, W24.
- Impression for acrocoracohumeral ligament on coracoid deep (char. 66: 0 > 1, also found in *Eudromia, Chauna, Dendrocygna*, Mirandornithes, *Streptoprocne, Rallus, Rostratula, Turnix, Pagodroma, Leptoptilos*, Suloidea, *Eudocimus, Pelecanus*, Accipitrimorphae, Strigiformes, *Urocolius*, and Eucavitaves); J14, K21, S24.
- Scapulotricipital impression on ulna deep (char. 156: 0 > 1, also found in Mirandornithes, Columbimorphae, Strisores, *Rallus*, Charadriiformes, *Phaethon, Gavia*, Procellariiformes, Suliformes, *Eudocimus, Scopus, Pandion*, Strigiformes, *Trogon, Bucco*, and *Psilopogon*); J14, P15, K21, S24, W24.
- Minor metacarpal weakly bowed (char. 173: 1 > 0, reversed in *Menura*; also found in *Gansus, Ichthyornis, Dendrocygna*, Mirandornithes, Apodiformes, Charadriiformes, *Phaethon, Gavia*, Procellariimorphae, *Fregata, Eudocimus, Pandion*, and Picodynastornithes); J14, P15, K21, S24, W24.
- Small tubercle on minor metacarpal distal to proximal synostosis of metacarpals absent (char. 185: 1 > 0, also found in *Pandion, Urocolius*, Eucavitaves, and most non-telluravian neognaths); J14, P15, K21, W24.

A marked impression for the acrocoracohumeral ligament is visible on the coracoid of *Vastanavis* (Fig. 1 in Mayr et al. 2007; Fig. 1b in Mayr et al. 2013), despite having been described as “shallow” in this taxon (Mayr et al. 2007), as well as that of the stem-psittaciform *Quercypsitta* (Pl. 2 in Mourer-Chauviré 1992b). An elongated, dorsally oriented external spine of the sternum is found in halcyornithids (albeit not as dorsally inflected as in crown-group Psittaciformes, Mayr 2007b; Fig. 3 in Mayr and Kitchener 2024d), psittacopedids (Mayr and Daniels 1998; Mayr 2015b, 2020), morsoravids (Mayr and Kitchener 2023h; Ksepka et al. 2025), zygodactylids (Mayr 1998b, 2008b; Mayr and Kitchener 2023g), and the stem-passeriform *Minutornis* (Mayr and Kitchener 2023g), though its exact proportions relative to the rest of the sternum are often difficult to determine in fossils due to incomplete preservation. Messelasturids exhibit a shorter external spine (Mayr 2021c; Mayr and Kitchener 2023f), which may suggest a stemward phylogenetic placement compared to the aforementioned taxa, as found by Mayr (2021c) and Mayr and Kitchener (2023f). A deep scapuoltricipital impression on the ulna has been reported in *Messelastur* (Mayr 2011d).

The presence of a weakly bowed minor metacarpal in messelasturids (Mayr 2000d, 2005c), halcyornithids (Mayr 1998c; Ksepka et al. 2011), *Eofringillirostrum* (Ksepka et al. 2019), *Psittacopes* (Mayr and Daniels 1998; Mayr and Kitchener 2023g), morsoravids (Mayr 1999c; Ksepka et al. 2025), zygodactylids (Fig. 26 in Mayr 1998b; Mayr and Kitchener 2023g), and *Minutornis* (Fig. 6o in Mayr and Kitchener 2023g) is consistent with affinities of these taxa to total-group Psittacopasseres within Telluraves. However, a more strongly bowed minor metacarpal is present in *Vastanavis* (Mayr et al. 2013) and the putative psittacopedids *Psittacomimus* and *Parapsittacopes* (Mayr and Kitchener 2023g), which may support a relatively stemward placement of *Vastanavis* in total-group Psittacopasseres, as found by Mayr and Kitchener (2023f).

Although not optimized as an unambiguous synapomorphy of Psittacopasseres in the present study, another pectoral character worthy of consideration as such is a bifurcated external spine on the sternum (char. 18: 1), given that this feature is thought to be plesiomorphic for both crown-group Psittaciformes (Mayr 2010c) and Passeriformes (Mayr and Kitchener 2023g). Among fossil pan-psittacopassereans, the external spine is weakly bifurcated in messelasturids (Mayr 2021c), morsoravids (Mayr and Kitchener 2023h), and the zygodactylid *Primoscens* (Mayr and Kitchener 2023g), and strongly bifurcated in *Minutornis* (Mayr and Kitchener 2023g), but not bifurcated in halcyornithids (Mayr 2007b), psittacopedids (Mayr and Daniels 1998; Mayr 2020), and other zygodactylids (Mayr 1998b, 2008b; Smith et al. 2018). If the assignment of all these taxa to total-group Psittacopasseres is correct, this may indicate that a bifurcated external spine has been secondarily lost or convergently evolved multiple times within this lineage. It is also possible that this trait has deeper phylogenetic origins, as a slightly bifurcated external spine has been reported in the type specimen of *Danielsraptor*, though it is not present in a tentatively referred second specimen (Mayr and Kitchener 2021).

### Character support for alternative phylogenetic topologies

#### Deep divergences within Neoaves

The phylogenetic relationships among Neoaves inferred from recent phylogenomic studies notably conflict with each other in several respects. For example, the extant sister group to all other neoavians has been variously recovered as Mirandornithes (the noncoding tree of Braun and Kimball 2021; Kuhl et al. 2021; Stiller et al. 2024), a clade uniting Mirandornithes and Columbimorphae (Jarvis et al. 2014; Reddy et al. 2017; Houde et al. 2019; the coding exon tree of Braun and Kimball 2021 following RY coding; the combined retroelement and sequenced-based gene tree of Gatesy and Springer 2022), or Strisores (Prum et al. 2015; the retroelement tree of Gatesy and Springer 2022).

Using the present dataset, seven osteological character states were optimized as potential synapomorphies of a clade containing all neoavians except for Mirandornithes, of which four were inferred as such under both the Kuhl et al. (2021) and Stiller et al. (2024) topologies.

- Ventrolateral sulcus on sternum distinct (char. 34: 0 > 1, reversed in *Caprimulgus, Aegotheles, Sterna*, Gruoidea, *Eurypyga, Phoebastria, Leptoptilos*, Pelecaniformes, *Bucorvus*, and *Nestor*; also found in *Rollulus*); K21, S24.
- Two pairs of caudal fenestrae in sternum (char. 51: 1 > 2, reversed in *Monias, Caprimulgus*, Gruiformes, *Turnix*, Aequornithes, *Pandion* + *Elanus, Tyto*, Bucerotiformes, and Australaves; also found in *Gansus* and *Ichthyornis*), S24.
- Coracoid compressed and keeled at ventromedial margin of supracoracoid sulcus (char. 71: 0 > 1, reversed in *Pterocles, Ardeotis, Tapera, Aegotheles, Streptoprocne*, Gruiformes, Aequornithes, *Bucorvus, Nestor*, and *Acanthisitta*; also found in *Dendrocygna, Ortalis*, and *Rollulus*); K21, S24.
- Ridge ventral to impression of m. pectoralis on humerus prominent (char. 114: 0 > 1, reversed in *Monias, Tapera*, Apodiformes, *Charadrius, Limosa, Alca, Sarothrura, Rallus, Gavia, Oceanites* + *Pagodroma*, Pelecanimorphae, *Urocolius*, Pici, *Pandion* + *Elanus, Nestor*, and *Acanthisitta*; also found in *Anseranas* and *Ortalis*); K21, S24.
- Humerus narrowest near midshaft point (char. 126: 0 > 2, reversed in *Monias, Ardeotis*, Gruiformes, *Limosa, Alca, Phaethon, Gavia, Phoebastria, Pagodroma*, Suliformes, *Eudocimus, Coragyps, Elanus*, Strigiformes, *Urocolius, Merops, Alcedo, Bucco, Micrastur*, and *Acanthisitta*; also found in *Ichthyornis, Alectura*, and *Rollulus*); S24.
- Radial incisure on ulna absent or indistinct (char. 155: 1 > 0, reversed in *Ardeotis, Nyctibius*, Grues, Charadriiformes, *Phaethon, Phoebastria, Pagodroma, Leptoptilos, Fregata, Sula, Scopus* + *Pelecanus*, Accipitrimorphae, and Psittaciformes; also found in *Dendrocygna* and Galliformes); K21, S24.
- Distal synostosis of major and minor metacarpals shorter than craniocaudal width (char. 197: 1 > 0, reversed in *Monias, Psophia*, Ralloidea, *Charadrius*, Scolopaci, *Sterna, Gavia, Eudocimus*, and *Cariama*; also found in *Gansus* and Galliformes); S24.

Two character states were optimized as potential synapomorphies of all neoavians excluding both Mirandornithes and Columbimorphae under the Jarvis et al. (2014) topology.

- Coracobrachial nerve sulcus on humerus prominent (char. 113: 0 > 1, reversed in *Tapera, Podargus, Psophia*, Lari, *Eurypyga, Spheniscus, Oceanites* + *Pagodroma*, Suloidea, *Eudocimus, Scopus, Pandion, Ninox, Leptosomus, Bucorvus, Merops, Alcecdo, Cariama, Nestor, Acanthisitta*, and Tyranni; also found *Anseranas, Alectura, Ortalis, Podilymbus*, and *Pterocles*).
- Brachial fossa on humerus deep (char. 134: 0 > 1, reversed in *Tapera, Sarothrura, Rallus, Rostratula, Turnix, Alca, Phoebastria, Oceanites, Tigrisoma, Elanus, Ninox, Upupa, Alcedo, Cariama, Psittacus*, and *Erythropitta*; also found in *Ichthyornis*, Anseriformes, and *Phoenicopterus*).

One character state was optimized as a potential synapomorphy for a clade containing all neoavians except Strisores under the Prum et al. (2015) topology.

- Radial tendinal sulcus well-defined (char. 147: 0 > 1, reversed in *Corythaeola, Psophia, Leptoptilos, Eudocimus, Phoenicopterus*, Charadrii, *Turnix, Pandion, Merops, Bucco, Nestor*, and *Neopelma*; also found in *Ichthyornis, Chauna*, and Letornithes).

Contrasting with other phylogenomic studies, Wu et al. (2024b) recovered the split at the root of Neoaves between Telluraves and a clade containing all other neoavians, referred to as Aquaterraves. However, no unambiguous synapomorphies were found for Aquaterraves using the present dataset.

It is perhaps noteworthy that a greater number of characters in our dataset potentially exclude Mirandornithes from other neoavians compared to those supporting competing topologies. However, two of these characters are also absent in *Caprimulgus*, a representative of Caprimulgidae, which is likely the extant sister group to the rest of Strisores (Prum et al. 2015; Chen et al. 2019; White and Braun 2019; Kuhl et al. 2021; Stiller et al. 2024; Wu et al. 2024). As such, they do not offer unambiguous support against Strisores representing the extant sister group to other neoavians. Conversely, the character potentially excluding Strisores from other neoavians is absent in at least some Mirandornithes and thus does not provide evidence against Mirandornithes as the extant sister group of other neoavians. The characters potentially excluding Mirandornithes and Columbimorphae from other neoavians are also found in multiple non-neoavian taxa and some mirandornitheans, raising the possibility that they are instead plesiomorphic for crown birds.

Two character states were optimized as potential synapomorphies for a clade uniting Mirandornithes and Columbimorphae to the exclusion of other neoavians under the Jarvis et al. (2014) topology.

- Humeral shaft straight (char. 125: 0 > 1, reversed in *Monias*; also found in Apodiformes, *Opisthocomus*, Charadriiformes, *Phaethon*, Procellariimorphae, *Fregata, Leucocarbo, Urocolius, Psilopogon, Psittacus, Neopelma*, and Passeri).
- Scapulotricipital impression on ulna deep (char. 156: 0 > 1, also found in Strisores, *Rallus*, Charadriiformes, *Phaethon, Gavia*, Procellariiformes, Suliformes, *Eudocimus, Scopus, Pandion*, Strigiformes, *Trogon, Bucco, Psilopogon*, and Psittacopasseres).

If Mirandornithes is not the extant sister group to other neoavians (either by itself or alongside Columbimorphae), one alternative hypothesis for its affinities is a position close to Charadriiformes (Prum et al. 2015), members of which also share both of the traits above. One of these character states was optimized as a potential synapomorphy for a clade uniting Mirandornithes and Charadriiformes to the exclusion of other neoavians under the Prum et al. (2015) topology.

- Humeral shaft straight (char. 125: 0 > 1, reversed in *Burhinus*; also found in *Pterocles, Columba*, Apodiformes, *Opisthocomus, Phaethon*, Procellariimorphae, *Fregata, Leucocarbo, Urocolius, Psilopogon, Psittacus, Neopelma*, and Passeri).

In the results of Wu et al. (2024b), Mirandornithes is recovered as the sister group to a clade uniting *Opisthocomus* and Phaethoquornithes. Three character states were optimized as potential synapomorphies supporting this relationship.

- Keel low, less than two-thirds total height of sternum (char. 49: 1 > 0, reversed in *Eudocimus*; also found in *Chauna, Anseranas, Tapera*, Vanescaves, *Psophia*, Charadrii, *Limosa*, and Telluraves).
- Olecranon fossa on humerus shallow (char. 127: 1 > 0, reversed in Phaethontimorphae, Procellariimorphae, Suliformes, and *Scopus* + *Pelecanus*; also found in non-neognath birds, *Anseranas, Monias, Ardeotis, Nyctibius*, Grues, and *Alca*).
- Extensor process on carpometacarpus surpasses distal articular facet for alular digit by less than width of facet (char. 181: 2 > 1, reversed in *Eurypyga*, Procellariiformes, and Pelecanimorphae; also found in non-neognath birds, *Monias, Corythaeola, Charadrius*, Bucerotiformes, *Nestor*, and *Climacteris*).

All three of these character states are also found in non-neognath birds, raising the possibility that they are plesiomorphic for Neoaves. Character support from the present dataset therefore does not strongly favor any of the aforementioned alternatives for the affinities of Mirandornithes. However, reanalyses of the Prum et al. (2015) dataset by Braun and Kimball (2021) suggest that the close relationship between Mirandornithes and Charadriiformes may be an artifact driven by data-type effects. Furthermore, Mirarab et al. (2024) presented evidence that the proposed sister-group relationship between Mirandornithes and Columbimorphae is likewise a probable analytical artifact caused by a period of sequence polymorphism early in the evolutionary history of Neoaves. Our identification of multiple potential synapomorphies excluding Mirandornithes from a clade containing all or most other neoavians may thus add to a growing consensus for a sister-group relationship between Mirandornithes and the remainder of Neoaves as the most likely divergence at the root of crown-group Neoaves (Jarvis et al. 2014; Reddy et al. 2017; Braun and Kimball 2021; Kuhl et al. 2021; Gatesy and Springer 2022; Stiller et al. 2024).

Both Jarvis et al. (2014) and Prum et al. (2015) recovered a clade within Neoaves that consists of *Opisthocomus*, Gruiformes, Charadriiformes, Phaethoquornithes, and Telluraves while excluding Columbimorphae, Otidimorphae, and Strisores. Under the Prum et al. (2015) topology, this clade also includes Mirandornithes (see previous paragraph for discussion on the phylogenetic position of Mirandornithes). Four character states were optimized as potential synapomorphies of this clade using the present dataset, though only one was inferred as such under both topologies.

- Costal margin 25–75% of total sternum length (char. 35: 0 > 1, reversed in Mirandornithes [if considered members of this clade], *Sarothrura, Burhinus, Rostratula, Turnix, Tigrisoma, Ninox, Urocolius*, Eucavitaves, and Eupasseres; also found in *Ichthyornis*, Anseriformes, *Corythaeola*, and *Streptoprocne*); J14, P15.
- Five or more articular facets on costal margin of sternum (char. 36: 1 > 2, reversed in *Tigrisoma, Turnix*, Strigiformes, *Urocolius*, Eucavitaves, and Tyranni; also found in *Gansus, Ichthyornis*, Anseriformes, *Podargus*, Apodiformes, *Corythaeola*, and *Monias*); P15.
- Dorsal pneumotricipital fossa narrower than ventral pneumotricipital fossa (char. 112: 1 > 0, reversed in *Sterna, Trogon, Alcedo*, and *Acanthisitta*; also found in *Rollulus*); P15.
- Ventral epicondyle on humerus equal or distal to ventral condyle (char. 130: 0 > 1, reversed in *Balearica, Podica, Rallus, Burhinus, Turnix, Sterna, Phaethon, Oceanites, Leptoptilos*, Suloidea, *Eudocimus, Coragyps, Pandion, Tyto*, and *Nestor*; also found in Galliformes, *Phoenicopterus*, Pteroclimesites, *Tapera*, and Letornithes); J14.

Characters 35 (1) and 130 (1) were also optimized as potential synapomorphies for a similar neoavian clade recovered by Stiller et al. (2024), which excludes Mirandornithes, Columbimorphae, and Otidimorphae, but not Strisores. Under this topology, these character states experienced reversals in Strisores.

In contrast, Kuhl et al. (2021) recovered a clade consisting of Gruiformes, Charadriiformes, Otidimorphae, Columbimorphae, *Opisthocomus*, and Strisores. However, no unambiguous synapomorphies were optimized for this clade using the present dataset.

*Opisthocomus*, Gruiformes, Charadriiformes, Phaethoquornithes, and Strisores form an exclusive clade called Elementaves in the results of Stiller et al. (2024). One character state was optimized as a potential synapomorphy for this clade.

- Pronounced ventral curvature of scapula (char. 94: 0 > 1, reversed in *Podargus, Aegotheles, Streptoprocne, Balearica*, Charadriiformes, *Phaethon, Gavia, Phoebastria*, and Pelecanes; also found in *Coragyps, Pandion, Tyto, Merops, Alcedo, Psilopogon, Nestor, Acanthisitta*, and Tyranni).

Within Elementaves, Stiller et al. (2024) recovered Phaethoquornithes and Strisores as extant sister taxa. Four character states were optimized as potential synapomorphies for this clade.

- Hypocleideum less than 5% the total length of the furcula (char. 9: 1 > 0, reversed in *Streptoprocne* and *Oceanites*; also found in *Podilymbus*, Scolopaci, *Alca, Coragyps, Bucorvus, Alcedo, Cariama*, and *Menura*).
- Internal lip of coracoid expanded in medial portion (char. 89: 0 > 1, reversed in *Gavia, Pagodroma*, and Pelecanes; also found in *Phoenicopterus, Corythaeola, Ardeotis, Tapera, Balearica, Elanus, Leptosomus, Bucorvus*, and *Cariama*).
- Transverse sulcus on humerus intermediate in length (char. 115: 0 > 1, reversed in *Podargus, Eurypyga, Spheniscus*, and *Phoebastria*; also found in *Anseranas, Balearica, Charadrius, Coragyps, Elanus, Tyto, Coracias*, and Psittaciformes).
- Scapulotricipital impression on ulna deep (char. 156: 0 > 1, reversed in Daedalornithes, *Spheniscus, Leptoptilos, Pelecanus*, and *Tigrisoma*; also found in Mirandornithes, Columbimorphae, *Rallus*, Charadriiformes, *Pandion*, Strigiformes, *Trogon, Bucco, Psilopogon*, and Psittacopasseres).

A sister-group relationship between Gruiformes and Charadriiformes, forming a clade called Cursorimorphae, was found by Jarvis et al. (2014), Kuhl et al. (2021), Stiller et al. (2024), and Wu et al. (2024b). No character states in the present dataset were optimized as unambiguous synapomorphies of this clade under the Jarvis et al. (2014) or Kuhl et al. (2021) topologies, though two potential synapomorphies were recovered under the Stiller et al. (2024) and Wu et al. (2024b) topologies, one of which was inferred as such under both.

- Supracoracoid nerve foramen or incisure in coracoid present (char. 72: 0 > 1, reversed in *Rostratula* and *Turnix*; also found in *Ichthyornis, Anseranas, Dendrocygna, Phoenicopterus, Pterocles, Corythaeola*, Daedalornithes, *Phaethon, Gavia*, Procellariiformes, *Leptoptilos, Eudocimus, Pelecanus*, Accipitrimorphae, Strigiformes, *Leptosomus*, and *Micrastur*); S24, W24.
- Minor metacarpal prominently narrows distally in caudal view (char. 179: 0 > 1, reversed in *Psophia*; also found in *Ichthyornis*, Anseres, Mirandornithes, Columbimorphae, *Aegotheles*, Phaethontimorphae, Pelecanimorphae, *Leptosomus, Coracias*, Accipitrimorphae, Strigiformes, and Australaves); W24.

A close relationship between members of Gruiformes and Charadriiformes has long been hypothesized based on anatomical data, especially cranial characteristics such as the presence of occipital fontanelles, supraorbital salt glands, and schizorhinal nostrils (e.g., Beddard 1898; Olson 1979, 1985; Livezey and Zusi 2007), but support for this hypothesis from the pectoral girdle and forelimb skeleton seems to be limited.

Cursorimorphae was recovered as the extant sister group to Strisores by Wu et al. (2024b), forming a clade called Litusilvanae. One character state was optimized as a potential synapomorphy for this clade.

- Dorsoventral orientation of acrocoracoid process essentially coplanar with main craniocaudal axis of coracoid (char. 64: 0 > 1, reversed in Grues, *Rallus, Limosa*, and *Alca*; also found in Galliformes, *Pterocles, Columba, Urocolius*, Eucavitaves, Tyranni, and *Climacteris*).

An alternative phylogenetic position for Charadriiformes close to Mirandornithes and Phaethoquornithes as part of an “extended waterbird clade” (i.e., Aequorlitornithes) was recovered by Prum et al. (2015). Four character states were optimized as potential synapomorphies of Aequorlitornithes using the present dataset.

- Pointed acromial processes on furcula (char. 5: 0 > 1, reversed in *Podilymbus, Phaethon*, and Pelecanimorphae; also found in *Ichthyornis*, Anseriformes, *Nyctibius*, Apodiformes, *Pterocles, Sarothrura, Coragyps, Pandion, Urocolius*, and *Acanthisitta*).
- Mound-shaped tuberosity on lateral surface of scapula (char. 96: 0 > 1, reversed in *Podilymbus, Turnix, Sterna, Phaethon*, Procellariimorphae, *Scopus*, and *Tigrisoma*; also found in *Ardeotis* and Bucerotiformes).
- Scapulotricipital impression on ulna deep (char. 156: 0 > 1, reversed in *Charadrius, Eurypyga, Spheniscus, Leptoptilos, Pelecanus*, and *Tigrisoma*; also found in Columbimorphae, Strisores, *Rallus, Pandion*, Strigiformes, *Trogon, Bucco, Psilopogon*, and Psittacopasseres).
- Minor metacarpal weakly bowed (char. 173: 1 > 0, reversed in *Turnix, Eurypyga, Oceanites, Leptoptilos*, Suloidea, and *Pelecanus* + *Tigrisoma*; also found in *Ichthyornis, Dendrocygna*, Apodiformes, Ralloidea, *Pandion*, Picocoraciades, and Psittacopasseres).

It is perhaps noteworthy that half of these character states (chars. 5: 1 and 173: 0) are also found in *Ichthyornis* and anseriforms, raising the possibility that adaptation toward an aquatic lifestyle readily drives their convergent evolutionary acquisition. This would also be consistent with the findings of Braun and Kimball (2021), which suggest that the recovery of Aequorlitornithes is an artifact of data-type effects (see also previous discussion on the phylogenetic position of Mirandornithes).

A single character state was optimized as a potential synapomorphy of a group containing members of Aequorlitornithes, *Opsithocomus*, and Telluraves to the exclusion of other neoavians, another clade recovered by Prum et al. (2015).

- Keel low, less than two-thirds total height of sternum (char. 49: 1 > 0, reversed in *Rostratula*, Lari, *Eudocimus, Upupa*, Psittaciformes, and *Menura*; also found in *Chauna, Anseranas, Tapera*, Vanescaves, and *Psophia*).

Jarvis et al. (2014) and Kuhl et al. (2021), on the other hand, recovered a sister-group relationship between Phaethoquornithes and Telluraves, but no unambiguous synapomorphies were optimized for this clade using the present dataset.

#### Affinities of Musophagiformes, Otidiformes, and Cuculiformes

Another point of contention regarding neoavian phylogeny pertains to the affinities of turacos (Musophagiformes), bustards (Otidiformes), and cuckoos (Cuculiformes). In the results of Jarvis et al. (2014), Prum et al. (2015), Stiller et al. (2024), Wu et al. (2024b), and the combined retroelement and sequence-based gene tree of Gatesy and Springer (2022), these three groups form a clade to the exclusion of other neoavians, known as Otidimorphae. However, in some other analyses, either Cuculiformes (Kuhl et al. 2021) or Musophagiformes (Reddy et al. 2017; the noncoding tree of Braun and Kimball 2021) has been found to be only distantly related to the other two lineages. Four character states were optimized as potential synapomorphies of a clade uniting all three groups using the present dataset, though only one was inferred as such under all four molecular reference topologies supporting the monophyly of Otidimorphae.

- Ventromedial intermuscular line on sternum absent or weak (char. 39: 1 > 0, also found in *Gansus, Ichthyornis, Chauna*, Strisores, *Opisthocomus, Rallus, Turnix*, Phaethontimorphae, *Gavia, Pelecanus, Elanus, Ninox*, Coraciimorphae, and Passeriformes); P15, S24, W24.
- Internal lip of coracoid expanded in medial portion (char. 89: 0 > 1, also found in *Phoenicopterus*, Strisores, *Balearica*, Phaethontimorphae, *Spheniscus, Phoebastria, Leptoptilos, Leucocarbo, Scopus* + *Pelecanus, Elanus, Leptosomus, Bucorvus*, and *Cariama*); S24.
- Bicipital crest on humerus inconspicuous (char. 117: 1 > 0, also found in *Chauna, Anseranas, Alectura, Opisthocomus, Balearica, Eurypyga, Leptoptilos, Fregata, Scopus, Tigrisoma, Tyto, Leptosomus, Bucorvus, Cariama*, and *Micrastur*); J14, P15, S24, W24.
- Ventral ramus of pisiform subequal in cranial extent to dorsal ramus or longer (char. 165: 0 > 1, also found in *Anseranas*, Galliformes, Mirandornithes, *Opisthocomus, Rostratula, Gavia, Oceanites* + *Pagodroma, Leptoptilos, Eudocimus, Ninox, Urocolius, Upupa*, Picocoraciades, and Australaves); J14, P15, S24.

In general, these characters may be suggestive of close affinities among Musophagiformes, Otidiformes, and Cuculiformes, and a close relationship between at least Musophagiformes and Cuculiformes has long been hypothesized based on comparative anatomy (e.g., Pycraft 1903; Mayr and Clarke 2003; Livezey and Zusi 2007; Mayr 2011a). However, all of these character states are also found in clades that have been recovered as potential close relatives of these three groups by recent phylogenomic studies, including members of Gruiformes and Aequornithes (Reddy et al. 2017; the non-coding tree of Braun and Kimball 2021). Consequently, the monophyly of a group uniting Musophagiformes, Otidiformes, and Cuculiformes remains to be further tested by future studies.

Possible comparisons to fossil forms are limited, given that the fossil record of Musophagiformes, Otidiformes, and Cuculiformes is scant (Mayr 2017b, 2022a), though the Eocene *Foro* is known from a nearly complete skeleton and has been identified as a potential stem-musophagid (Field and Hsiang 2018), and similarities to otidids have been noted in the enigmatic Eocene *Perplexicervix* (Mayr 2010d; Mayr et al. 2023a). A small bicipital crest is found in *Perplexicervix* (Mayr et al. 2023a), whereas *Foro* has been noted to have a more prominent bicipital crest on the humerus than in extant musophagiforms and cuculiforms, approaching the condition in accipitriforms (Olson 1992). If *Foro* is a stem-musophagid, and musophagiforms indeed form an exclusive clade with cuculiforms and/or otidiforms, it may represent an apomorphic reversal of a reduced bicipital crest within this clade.

An alternative topology in which Cuculiformes were recovered as the extant sister group of pigeons (Columbiformes) within Columbimorphae was found by Kuhl et al. (2021). Four character states were optimized as potential synapomorphies of this clade using the present dataset.

- Ventral supracondylar tubercle on humerus expanded (char. 141: 0 > 1, also found in *Ichthyornis, Chauna*, Galliformes, *Limosa, Turnix, Gavia, Oceanites, Fregata, Leucocarbo*, Coraciimorphae, and *Climacteris*).
- Carpal tubercle on ulna projects more than half the craniocaudal width of dorsal and ventral condyles (char. 162: 0 > 1, also found in *Ortalis, Caprimulgus, Florisuga, Opisthocomus, Psophia*, Charadrii, Scolopaci, *Sterna, Phaethon, Gavia*, Procellariiformes, *Fregata, Urocolius, Trogon, Upupa, Coracias, Psilopogon*, and Passeriformes).
- Prominent ligamental groove on ventral ramus of pisiform (char. 167: 0 > 1, also found in *Ichthyornis, Dendrocygna, Nyctibius, Streptoprocne*, Grues, Ralloidea, *Charadrius*, Scolopaci, *Sterna*, Phaethoquornithes, *Pandion*, Strigiformes, *Upupa, Coracias, Alcedo, Psittacus, Erythropitta*, and Passeri).
- Ridge linking ventral rim of carpal trochlea and pisiform process forms sharp drop-off relative to ventral surface of extensor process (char. 191: 0 > 1, also found in *Anseranas*, Galliformes, *Florisuga, Opisthocomus*, Grues, Heliornithes, *Turnix, Eurypyga, Leucocarbo, Eudocimus, Tigrisoma, Coragyps*, Strigiformes, *Urocolius, Psittacus*, and Tyranni).

Two additional character states present in *Tapera* were optimized as synapomorphies of Columbimorphae under this topology (see previous discussion on potential synapomorphies of Columbimorphae).

Even if Musophagiformes, Otidiformes, and Cuculiformes represent each other's closest living relatives, recent studies have found conflicting results regarding the internal topology of this clade. In Jarvis et al. (2014), Musophagiformes and Otidiformes were found forming a clade to the exclusion of Cuculiformes. A sister-group relationship between these two clades was also recovered by Kuhl et al. (2021), Luo et al. (2023), and Wu et al. (2024b), and this grouping was named Musophagotides by Sangster et al. (2022). Six character states were optimized as potential synapomorphies of Musophagotides using the present dataset, three of which were inferred as such all three molecular reference topologies supporting this group.

- Sternal keel maintains thickness ventrally (char. 48: 0 > 1, also found in *Podilymbus, Monias, Opisthocomus, Psophia, Balearica, Phaethon, Gavia, Spheniscus, Pagodroma, Fregata, Leucocarbo, Pelecanus, Pandion* + *Elanus, Leptosomus, Alcedo, Jynx*, and *Menura*); J14, K21, W24.
- Caudolateral trabeculae on sternum turn medially (char. 55: 1 > 0, also found in *Gansus, Eudromia, Ortalis, Podilymbus*, Columbimorphae, *Charadrius*, Lari, *Gavia, Spheniscus, Pelecanus, Coragyps, Bucorvus*, and *Bucco*); J14.
- Bicipital crest on humerus inconspicuous (char. 117: 1 > 0, also found in *Chauna, Anseranas, Alectura, Tapera, Opisthocomus, Balearica, Eurypyga, Leptoptilos, Fregata, Scopus, Tigrisoma, Tyto, Leptosomus, Bucorvus, Cariama*, and *Micrastur*); K21.
- Deltopectoral crest well-developed, extending at least one third the total length of the humerus (char. 122: 1 > 0, also found in *Gansus, Ichthyornis, Nyctibius, Podargus, Spheniscus, Leptoptilos, Leucocarbo, Eudocimus, Scopus*, Accipitrimorphae, Strigiformes, Cavitaves, *Micrastur*, and Passeri); J14, K21, W24.
- Humeroulnar trochlea on ulna absent or shallow (char. 153: 1 > 0, also found in *Ichthyornis*, Galloanserae, Columbimorphae, *Podargus, Aegotheles, Opisthocomus, Podica, Charadrius, Limosa, Turnix, Phaethon, Spheniscus, Leptoptilos, Fregata, Tigrisoma, Pandion, Leptosomus*, Bucerotiformes, *Coracias*, and *Alcedo*); J14.
- Impression of m. brachialis on ulna deep (char. 154: 0 > 1, also found in *Gansus, Ichthyornis, Eudromia, Chauna, Dendrocygna, Ortalis, Phoenicopterus, Aegotheles, Streptoprocne, Balearica, Podica, Charadrius, Limosa, Sterna, Phaethon, Phoebastria*, Suloidea, *Pelecanus*, Accipitrimorphae, Strigiformes, Cavitaves, *Psittacus, Acanthisitta*, and Passeri); J14, K21, W24.

Most of these characters cannot be assessed from published descriptions of *Foro*, though as noted previously it differs from extant musophagids in having a more prominent bicipital crest on the humerus. However, *Foro* does exhibit a well-developed deltopectoral crest (Olson 1992). An elongated deltopectoral crest also appears to be present in *Perplexicervix* (Mayr 2010d).

Prum et al. (2015) and Stiller et al. (2024) instead recovered a clade uniting Otidiformes and Cuculiformes to the exclusion of Musophagiformes, for which five character states were optimized as potential synapomorphies using the present dataset under both molecular topologies.

- Pneumatic foramen in median sulcus of sternum immediately caudal to cranial margin (char. 29: 0 > 1, also found in *Ichthyornis, Dendrocygna, Alectura, Ortalis, Phoenicopterus, Pterocles*, Strisores, *Opisthocomus*, Grues, *Podica, Phaethon*, Pelecanimorphae, *Coragyps*, Strigiformes, Picocoraciades, *Micrastur*, Psittaciformes, *Neopelma*, and *Menura*); P15, S24.
- Keel apex pointed in lateral view (char. 45: 0 > 1, also found in *Alectura, Podilymbus, Podargus, Aegotheles, Balearica*, Ralloidea, *Charadrius, Rostratula, Turnix, Alca, Leptoptilos*, Pelecaniformes, *Leptosomus, Bucorvus, Coracias, Alcedo, Acanthisitta*, and *Erythropitta*); P15, S24.
- Coracoid rounded and relatively thick at ventromedial margin of supracoracoid sulcus (char. 71: 1 > 0, also found in non-neoavian birds, Mirandornithes, *Pterocles, Aegotheles, Streptoprocne*, Gruiformes, Aequornithes, *Bucorvus, Nestor*, and *Acanthisitta*); P15, S24.
- Lateral process of coracoid sharply hooked (char. 82: 0 > 1, also found in *Gansus, Ichthyornis, Anseranas, Rollulus*, Strisores, Grues, *Burhinus, Sterna, Phaethon*, Procellariiformes, *Leptoptilos, Fregata, Leucocarbo, Scopus* + *Pelecanus, Pandion, Trogon, Bucorvus, Coracias, Bucco*, and *Psittacus*); P15, S24.
- Radial bicipital tubercle prominent (char. 146: 0 > 1, also found in *Aegotheles, Florisuga*, Grues, *Charadrius, Phoebastria, Leucocarbo, Scopus, Tigrisoma, Elanus, Tyto, Leptosomus*, Bucerotiformes, *Merops*, Piciformes, and Passeriformes); P15, S24.

In the absence of increased taxon sampling and more completely known fossil representatives of Musophagiformes, Otidiformes, and Cuculiformes to further clarify the polarity and distribution of these potential synapomorphies, skeletal characters from the pectoral girdle and forelimb do not seem to clearly favor any of the recent alternative hypotheses regarding the interrelationships of these clades.

With respect to the phylogenetic relationships of Musophagiformes, Otidiformes, and Cuculiformes to other neoavians, Jarvis et al. (2014) recovered Otidimorphae as the extant sister group to Strisores. Five character states were optimized as potential synapomorphies uniting Otidimorphae and Strisores to the exclusion of other neoavians using the present dataset.

- Pneumatic foramen in median sulcus of sternum immediately caudal to cranial margin (char. 29: 0 > 1, reversed in *Corythaeola* and *Nyctibius*; also found in *Ichthyornis, Dendrocygna, Alectura, Ortalis, Phoenicopterus, Pterocles, Opisthocomus*, Grues, *Podica, Phaethon*, Pelecanimorphae, *Coragyps*, Strigiformes, Picocoraciades, *Micrastur*, Psittaciformes, *Neopelma*, and *Menura*).
- Dorsolateral intermuscular line on sternum absent or weak (char. 38: 1 > 0, reversed in *Ardeotis, Podargus*, and Apodiformes; also found in *Sarothrura, Rallus, Phoebastria, Fregata, Sula, Pelecanus*, and *Bucorvus*).
- Lateral process of coracoid strongly hooked (char. 82: 0 > 1, reversed in *Corythaeola* and Daedalornithes; also found in *Gansus, Ichthyornis, Anseranas, Rollulus*, Grues, *Burhinus, Sterna, Phaethon*, Procellariiformes, *Leptoptilos, Fregata, Leucocarbo, Scopus* + *Pelecanus, Pandion, Trogon, Bucorvus, Coracias, Bucco*, and *Psittacus*).
- Internal lip of coracoid expanded in medial portion (char. 89: 0 > 1, also found in *Phoenicopterus, Balearica*, Phaethontimorphae, *Spheniscus, Phoebastria, Leptoptilos, Leucocarbo, Scopus* + *Pelecanus, Elanus, Leptosomus, Bucorvus*, and *Cariama*).
- Ventral collateral ligamental tubercle on ulna strongly developed (char. 157: 0 > 1, reversed in *Ardeotis*; also found in *Ichthyornis, Ortalis, Psophia*, Charadriiformes, Procellariiformes, *Fregata, Pelecanus, Elanus*, Strigiformes, Coraciimorphae, *Nestor*, and Passeriformes).

In contrast, Prum et al. (2015), Kuhl et al. (2021), Stiller et al. (2024), and Wu et al. (2024b) recovered Otidimorphae as most closely related to Columbimorphae (with Cuculiformes being nested within the latter group in the results of Kuhl et al. 2021), forming a clade called Columbaves. Five character states were optimized as potential synapomorphies of Columbaves, one of which was inferred as such all four molecular reference topologies supporting this group.

- Pneumatic pores along cranial margin of dorsal sternum (char. 32: 0 > 1, reversed in *Pterocles* and *Corythaeola*; also found in Anseriformes, *Ortalis, Phoenicopterus, Tapera, Caprimulgus*, Apodiformes, *Opisthocomus*, Gruoidea, *Podica, Phaethon, Leptoptilos, Sula, Eudocimus, Scopus, Pelecanus, Pandion*, Strigiformes, *Trogon, Coracias, Merops*, and Australaves); W24.
- Caudolateral trabeculae on sternum turn medially (char. 55: 1 > 0, reversed in *Tapera*; also found in *Gansus, Eudromia, Ortalis, Podilymbus, Charadrius*, Lari, *Gavia, Spheniscus, Pelecanus, Coragyps, Bucorvus*, and *Bucco*); P15, K21.
- Lateral extent of lateral process on coracoid half the width of sternal facet or greater (char. 81: 0 > 1, reversed in *Ardeotis*; also found in *Caprimulgus, Podargus, Phoebastria, Pagodroma, Fregata, Sula, Scopus* + *Pelecanus, Charadrius*, Scolopaci + Lari, *Elanus, Tyto, Leptosomus, Upupa, Coracias* + *Merops*, and *Bucco*); P15, S24, W24.
- Quill knobs on ulna strongly developed (char. 160: 0 > 1, reversed in *Pterocles*; also found in *Anseranas, Podilymbus, Caprimulgus, Aramus, Charadrius*, Scolopaci + Lari, *Oceanites*, Pelecanimorphae, Accipitrimorphae, Picocoraciades, and Passeriformes); P15, K21, S24, W24.
- Cranial projection on distal end of major metacarpal weak (char. 196: 1 > 0, reversed in *Ardeotis*; also found in *Gansus, Ortalis* + *Rollulus, Opisthocomus, Psophia, Aramus*, Heliornithes, *Rostratula, Turnix, Spheniscus, Tigrisoma*, Strigiformes, *Upupa, Merops, Bucco, Cariama*, and Passeriformes); S24.

Three of the above character states can also be found in *Caprimulgus*, and therefore do not necessarily contradict the possibility of an Otidimorphae + Strisores clade. One of these characters, the presence of prominent quill knobs, is lacking in *Foro* (Olson 1992).

#### Interrelationships within Telluraves

Within Telluraves, Jarvis et al. (2014), Kuhl et al. (2021), and Stiller et al. (2024) recovered a clade containing Accipitrimorphae, Strigiformes, and Coraciimorphae, forming a group called Afroaves. Seven character states were optimized as potential synapomorphies of Afroaves, of which two were inferred as such under all three topologies.

- Keel apex on sternum caudal to main body of sternum (char. 43: 1 > 0, reversed in Cavitaves; also found in *Eudromia*, Galliformes, *Monias, Opisthocomus, Aramus, Sarothrura, Rallus, Charadrius*, Scolopaci, *Turnix, Pagodroma, Fregata, Micrastur*, and *Acanthisitta*); J14, K21.
- Acrocoracoid process on coracoid straight (char. 65: 1 > 0, reversed in Cavitaves; also found in *Gansus*, Anseriformes, *Ortalis, Phoenicopterus, Monias, Ardeotis*, Strisores, *Opisthocomus*, Gruiformes, *Gavia, Pagodroma*, Pelecanimorphae, *Micrastur*, and *Acanthisitta*); K21.
- Impression for acrocoracohumeral ligament on coracoid deep (char. 66: 0 > 1, reversed in *Leptosomus, Bucorvus*, and *Bucco*; also found in *Eudromia, Chauna, Dendrocygna*, Mirandornithes, *Streptoprocne, Rallus, Rostratula, Turnix, Pagodroma, Leptoptilos*, Suloidea, *Eudocimus, Pelecanus*, and Psittacopasseres); J14, K21, S24.
- Deltopectoral crest well-developed, extending at least one third the total length of the humerus (char. 122: 1 > 0, reversed in *Urocolius* and Coraciiformes; also found in *Gansus, Ichthyornis, Corythaeola, Ardeotis, Nyctibius, Podargus, Spheniscus, Leptoptilos, Leucocarbo, Eudocimus, Scopus, Micrastur*, and Passeri); J14, K21.
- Scar of m. flexor carpi ulnaris on flexor process of humerus forms single large scar (char. 142: 2 > 1, reversed in *Pandion, Leptosomus*, and *Psilopogon*; also found in *Eudromia*, Galliformes, *Tapera, Streptoprocne, Opisthocomus, Psophia, Aramus, Rallus, Eurypyga, Acanthisitta*, and *Erythropitta*); J14, K21, S24.
- Radius craniocaudally curved (char. 144: 0 > 1, reversed in *Elanus, Urocolius*, and *Coracias*; also found in Anseriformes, *Phoenicopterus, Ardeotis*, Strisores, Grues, Charadrii, Feraequornithes, *Micrastur*, Tyranni, and *Climacteris*); J14, K21.
- Impression of m. brachialis on ulna deep (char. 154: 0 > 1, reversed in *Urocolius, Alcedo*, and Pici; also found in *Gansus, Ichthyornis, Eudromia, Chauna, Dendrocygna, Ortalis, Phoenicopterus, Corythaeola, Ardeotis, Aegotheles, Streptoprocne, Balearica, Podica, Charadrius, Limosa, Sterna, Phaethon, Phoebastria*, Suloidea, *Pelecanus, Psittacus, Acanthisitta*, and Passeri); J14, K21.

Prum et al. (2015) instead found a Strigiformes + Coraciimorphae clade as sister to Australaves, forming a group called Eutelluraves. Two character states were optimized as potential synapomorphies of Eutelluraves.

- Humeral articular facet on coracoid flat or convex (char. 69: 0 > 1, reversed in Bucerotiformes, Psittaciformes, *Acanthisitta*, and *Neopelma*; also found in *Ortalis* + *Rollulus, Phoenicopterus, Monias, Columba, Corythaeola, Ardeotis*, Strisores, *Psophia, Rostratula*, Lari, *Eurypyga, Pagodroma*, and *Elanus*).
- Prominent caudal groove on caudal surface of minor metacarpal distal to synostosis with major metacarpal (char. 180: 0 > 2, reversed in *Bucorvus, Bucco*, and Psittaciformes; also found in *Ortalis* + *Rollulus, Tapera*, and *Podica*).

The Strigiformes + Coraciimorphae clade was recovered by Jarvis et al. (2014), Prum et al. (2015), and Kuhl et al. (2021). Two character states were optimized as potential synapomorphies of this clade, both of which were inferred as such under all three molecular topologies.

- Four facets for sternal ribs on costal margin of sternum (char. 36: 2 > 1, reversed in *Leptosomus, Upupa, Coracias*, and Pici; also found in *Eudromia, Alectura, Ortalis, Pterocles, Columba, Ardeotis, Caprimulgus, Nyctibius, Aegotheles*, and Tyranni); J14, P15, K21.
- Ventral collateral ligamental tubercle on ulna strongly developed (char. 157: 0 > 1, reversed in *Bucorvus* and *Merops*; also found in *Ichthyornis, Ortalis, Corythaeola, Tapera*, Strisores, *Psophia*, Charadriiformes, Procellariiformes, *Fregata, Pelecanus, Elanus, Nestor*, and Passeriformes); J14, P15, K21.

These characters are difficult to evaluate in potential stem-strigiform specimens (Mourer-Chauviré 1987; Peters 1992; Mayr et al. 2020c), though *Ypresiglaux* exhibits five costal processes on the sternum instead of four (Mayr and Kitchener 2023d), which may suggest that a reduction to four occurred independently in strigiforms and coraciimorphs. Sandcoleids (Pl. I in Houde and Olson 1992) and *Waltonavis* (Fig. 9i in Mayr and Kitchener 2023e) appear to have a strongly developed ventral collateral ligamental tubercle on the ulna.

Stiller et al. (2024) and Wu et al. (2024b) instead recovered Accipitrimorphae and Strigiformes as sister groups, forming a clade called Hieraves. Six character states were optimized as potential synapomorphies of this clade, though only one was inferred as such under both topologies.

- Keel apex on sternum caudal to main body of sternum (char. 43: 1 > 0, also found in *Eudromia*, Galliformes, *Monias, Opisthocomus, Aramus, Sarothrura, Rallus, Charadrius*, Scolopaci, *Turnix, Pagodroma, Fregata, Urocolius, Bucco, Micrastur*, and *Acanthisitta*); W24.
- Acrocoracoid process on coracoid straight (char. 65: 1 > 0, also found in *Gansus*, Anseriformes, *Ortalis, Phoenicopterus, Monias, Ardeotis*, Strisores, *Opisthocomus*, Gruiformes, *Gavia, Pagodroma*, Pelecanimorphae, *Urocolius, Psilopogon, Micrastur*, and *Acanthisitta*); W24.
- Supracoracoid nerve foramen in coracoid present (char. 72: 1 > 2, also found in *Ichthyornis, Anseranas, Phoenicopterus, Corythaeola*, Daedalornithes, Gruiformes, Charadrii, *Alca, Sterna, Phaethon*, Procellariiformes, *Leptoptilos, Eudocimus, Pelecanus, Leptosomus*, and *Micrastur*); S24, W24.
- Deltopectoral crest well developed, extending at least one third the total length of the humerus (char. 122: 1 > 0, also found in *Gansus, Ichthyornis, Corythaeola, Ardeotis, Nyctibius, Podargus, Spheniscus, Leptoptilos, Leucocarbo, Eudocimus, Scopus*, Cavitaves, *Micrastur*, and Passeri); W24.
- Radius craniocaudally curved (char. 144: 0 > 1, reversed in *Elanus*; also found in Anseriformes, *Phoenicopterus, Ardeotis*, Strisores, Grues, Charadrii, Feraequornithes, Cavitaves, *Micrastur*, Tyranni, and *Climacteris*); W24.
- Tendinal sulcus on carpometacarpus wraps around cranial surface of bone (char. 171: 0 > 1, reversed in *Pandion*; also found in *Nyctibius, Florisuga, Balearica*, Picocoraciades, *Micrastur*, and Passeriformes); S24.

A supracoracoid nerve foramen is present in *Ypresiglaux* (Mayr and Kitchener 2023d) and the stem-strigiform *Primoptynx* (Mayr et al. 2020c), but has also been reported in sandcoleids (Houde and Olson 1992; Ksepka et al. 2017; Mayr 2018), *Ypresicolius* (Mayr and Kitchener 2024f) and total-group leptosomids (Weidig 2006; Mayr 2008c; Mayr and Kitchener 2023e), and may therefore be ancestral for a more inclusive clade.

Whereas Stiller et al. (2024) placed Hieraves within Afroaves, Wu et al. (2024b) recovered it as the sister group to Australaves. Three character states were optimized as potential synapomorphies of this clade.

- Costal margin 25–75% of total sternum length (char. 35: 0 > 1, reversed in *Ninox* and Eupasseres; also found in *Ichthyornis*, Anseriformes, *Corythaeola, Streptoprocne*, and *Leptosomus*).
- Minor metacarpal prominently narrows distally in caudal view (char. 179: 0 > 1, also found in *Ichthyornis*, Anseres, Mirandornithes, Columbimorphae, *Aegotheles*, Gruiformes, Charadriiformes, Phaethontimorphae, Pelecanimorphae, *Leptosomus*, and *Coracias*).
- Prominent tubercle on minor metacarpal distal to proximal synostosis of metacarpals (char. 185: 0 > 1, reversed in *Pandion* and Psittacopasseres; also found in *Ichthyornis, Eudromia, Rollulus, Psophia, Burhinus, Turnix, Sterna, Phaethon, Leptosomus*, and *Coracias*).

Overall, a greater number of potential synapomorphies in this dataset support the Afroaves topology than the Eutelluraves or the Hieraves + Australaves topologies, though the presence of several potential afroavian synapomorphies in certain australavians (especially the falconiform *Micrastur*) raises the possibility that these characters may be telluravian symplesiomorphies. Similarly, a seemingly large number of characters supports Hieraves, but all of these traits are also found in members of Coraciimorphae and Australaves, rendering their inferences as hieravian synapomorphies questionable.

#### Affinities of *Opisthocomus*

One of the most contentious subjects in avian phylogenetics concerns the position of *Opisthocomus*. Jarvis et al. (2014) and Stiller et al. (2024) recovered it as the extant sister taxon of Cursorimorphae. Three character states were optimized as potential synapomorphies of this clade, all of which were inferred as such under both molecular topologies.

- Keel apex on sternum caudal to main body of sternum (char. 43: 1 > 0, reversed in *Psophia, Balearica, Podica, Burhinus, Alca*, and *Sterna*; also found in *Eudromia*, Galliformes, *Monias, Pagodroma, Fregata*, Accipitrimorphae, Strigiformes, *Urocolius, Bucco, Micrastur*, and *Acanthisitta*); J14, S24.
- Impression of m. sternocoracoidei on coracoid deep (char. 78: 0 > 1, reversed in *Psophia, Podica, Burhinus*, Scolopaci, and *Alca*; also found in *Gansus, Ichthyornis, Chauna, Phoenicopterus, Aegotheles, Spheniscus, Phoebastria, Coragyps, Coracias*, and *Cariama*); J14, S24.
- Tendinal sulcus on carpometacarpus barely perceptible (char. 169: 1 > 0, reversed in Gruoidea, Scolopaci, and *Sterna*; also found in *Podilymbus, Gavia, Spheniscus, Leucocarbo*, and *Urocolius*); J14, S24.

In contrast, Prum et al. (2015) recovered a clade uniting *Opisthocomus* and Telluraves to the exclusion of other neoavians, called Inopinaves. One character state was optimized as a potential synapomorphy of Inopinaves.

- Keel apex on sternum caudal to main body of sternum (char. 43: 1 > 0, reversed in Cavitaves, Psittaciformes, and Eupasseres; also found in *Eudromia*, Galliformes, *Monias, Aramus, Sarothrura, Rallus, Charadrius*, Scolopaci, *Turnix, Pagodroma*, and *Fregata*).

Kuhl et al. (2021) recovered *Opisthocomus* as the extant sister group of Strisores. Four character states were optimized as potential synapomorphies of a clade uniting *Opisthocomus* and Strisores to the exclusion of other neoavians.

- Acrocoracoid process on coracoid straight (char. 65: 1 > 0, reversed in *Aegotheles* and *Streptoprocne*; also found in *Gansus*, Anseriformes, *Ortalis, Phoenicopterus, Monias, Ardeotis*, Gruiformes, *Gavia, Pagodroma*, Pelecanimorphae, Accipitrimorphae, Strigiformes, *Urocolius, Micrastur*, and *Acanthisitta*).
- Pronounced ventral curvature of scapula (char. 94: 0 > 1, reversed in Letornithes; also found in Gruiformes, *Eurypyga, Spheniscus, Oceanites* + *Pagodroma, Leptoptilos, Tigrisoma, Coragyps, Pandion, Tyto, Merops, Alcedo, Psilopogon, Nestor, Acanthisitta*, and Tyranni).
- Apex of dorsal cotylar process of ulna approximately coplanar with dorsal surface of ulnar main body (char. 149: 0 > 1, reversed in *Aegotheles* and *Streptoprocne*; also found in *Ichthyornis*, Anseriformes, *Podilymbus, Corythaeola*, Charadriiformes, *Urocolius*, Eucavitaves, *Nestor*, and Passeriformes).
- Pneumatic foramen in infratrochlear fossa on carpometacarpus absent (char. 190: 1 > 0, reversed in *Streptoprocne*; also found in Mirandornithes, Pteroclimesites, *Rallus*, Scolopaci + Lari, Procellariimorphae, *Leucocarbo, Eudocimus, Tigrisoma, Ninox, Urocolius, Merops, Jynx, Pandion, Nestor*, and Passeriformes).

Wu et al. (2024b) recovered *Opisthocomus* as the extant sister group of Phaethoquornithes. Two character states were optimized as potential synapomorphies of a clade uniting *Opisthocomus* and Phaethoquornithes to the exclusion of other neoavians.

- Costal margin 25–75% of total sternum length (char. 35: 0 > 1, reversed in *Tigrisoma*; also found in *Ichthyornis*, Anseriformes, *Corythaeola, Streptoprocne*, Gruiformes, *Charadrius, Limosa, Alca* + *Sterna*, Accipitrimorphae, *Tyto, Leptosomus*, and Australaves).
- Ventromedial intermuscular line on sternum absent or weak (char. 39: 1 > 0, reversed in Feraequornithes; also found in *Gansus, Ichthyornis, Chauna*, Strisores, *Corythaeola, Ardeotis, Tapera, Rallus, Turnix, Elanus, Ninox*, Coraciimorphae, and Passeriformes).

Well-corroborated fossils of stem-opisthocomids are mostly based on fairly incomplete remains (Mayr et al. 2011; Mayr and De Pietri 2014), though a straight acrocoracoid process can be seen in *Namibiavis* and *Hoazinavis* (Mayr et al. 2011), and a ventrally curved scapula is evidenced in *Protoazin* (Mayr and De Pietri  2014). The pectoral girdle and forelimb skeleton provides limited support for preferring any of the three aforementioned alternative hypotheses for the phylogenetic position of *Opisthocomus*, given that essentially all of the potential synapomorphies listed above are found in members of more than one group that has been proposed to be its extant sister taxon. For example, the lone character state optimized as a potential synapomorphy of *Opisthocomus* + Telluraves is also present in members of Gruiformes and Charadriiformes, whereas the character states recovered as potential synapomorphies of *Opisthocomus* + Strisores or *Opisthocomus* + Phaethoquornithes are also present in some members of Gruiformes, Charadriiformes, or Telluraves. However, the presence of a barely perceptible tendinal sulcus on the carpometacarpus as a feature possibly linking *Opisthocomus* to Gruiformes and Charadriiformes is intriguing, considering that this character is otherwise primarily found in specialized diving taxa within Mirandornithes and Aequornithes.

In general, pectoral and forelimb skeletal characters appear to provide little unambiguous support for selecting among alternative molecular topologies. However, qualitative assessment of potential synapomorphies using the current dataset may weakly favor Mirandornithes as the extant sister group to all other neoavians (as opposed to being most closely related to Columbimorphae or Charadriiformes) and Accipitrimorphae as being more closely related to Strigiformes and Coraciimorphae than to Australaves (forming Afroaves, as opposed to being the extant sister group to all other telluravians). Although congruence between molecular and morphological sources of data can help increase confidence in specific phylogenetic hypotheses (Mayr 2008d; Lee and Camens 2009; Mayr 2011a; Giribet 2015; Lee and Palci 2015; Neumann et al. 2021; Hunt et al. 2025), the conclusions here must still be treated with caution. Homoplasy is evidently pervasive in the avian pectoral girdle and forelimb skeleton, and furthermore, the present study did not identify potential synapomorphies for at least one clade (Eufalconimorphae) strongly supported by phylogenomic studies.

That the morphological characters compiled in the present study generally do not strongly favor specific molecular topologies is further evidenced by the fact that no significant differences in RHI values resulted from mapping these characters onto alternative molecular topologies. Tree-distance metrics relative to topologies derived from analyzing the morphological dataset also did not differ appreciably among the molecular topologies examined here. Of the three molecular topologies studied, tree-distance metrics indicate that, on average, the Kuhl et al. (2021) topology exhibits the least similarity to most of the topologies derived from analyzing the morphological dataset (though the Jarvis et al. 2014 and Wu et al. 2024b topologies produce slightly longer tree length when enforced under the present dataset). The Stiller et al. (2024) topology exhibits the lowest average RF distances and shortest tree length. Along with the Prum et al. (2015) topology, it also produces the highest RI and CI (when ordered characters are considered) relative to the other molecular topologies, and exhibits the second lowest RHI after the Prum et al. (2015) tree. Stiller et al. (2024) reported that their phylogenomic topology was more congruent with the taxonomic distribution of nine continuous anatomical traits than that of Prum et al. (2015). The relatively high congruence of the Stiller et al. (2024) topology with morphological characters is upheld by our findings, despite extensive homoplasy in the present dataset.

In terms of RF distances, the results of analyzing the morphological dataset tended to exhibit the greatest similarity to molecular trees when constrained to the consensus tree of Braun and Kimball (2021). Although these results lead to the self-evident conclusion that increasing topological constraints informed by molecular topologies lead to greater congruence between morphological and molecular trees, they also highlight the challenge of identifying phylogenetic signal in avian pectoral and forelimb skeletal characters. This difficulty may partly be due to rampant homoplasy, but a mosaic distribution of morphological characters might also reflect the existence of a developmental zone of variability (*sensu*  Bever et al. 2011) in the early evolutionary history of birds. Accentuated effects of these phenomena would furthermore be consistent with recent hypotheses that the early radiation of neoavians was characterized by rapid, near-simultaneous diversification (Jarvis et al. 2014; Prum et al. 2015; Suh 2016; Houde et al. 2019; Kimball et al. 2019; Braun and Kimball 2021; Brocklehurst and Field 2024; Stiller et al. 2024), incomplete lineage sorting (Suh et al. 2015; Houde et al. 2020), and introgression (Braun and Kimball 2021; Gatesy and Springer 2022) among major lineages, and might not be best approximated by a strictly bifurcating branching pattern.

### Utility of pectoral and forelimb osteological characters in avian phylogenetics

The widespread distribution of most pectoral and forelimb characters optimized as synapomorphies of major avian clades, the limited congruence between results derived from unconstrained analyses of the morphological data and those of phylogenomic analyses, and heatmap visualizations of the phylogenetic distribution of morphological characters suggest that homoplasy is prevalent across the avian pectoral girdle and forelimb skeleton. Low phylogenetic signal in this anatomical region is further evidenced by generally low statistical support for deep nodes recovered by analyses of the present dataset (Figs. 8–15). This is consistent with studies that have concluded that body size and functional specializations, particularly those related to locomotion, exert substantial control on the skeletal morphology of the avian pectoral girdle and forelimb (Stegmann 1963; Stegmann 1964; Feduccia 1972; Hui 2002; Nudds et al. 2007; Habib and Ruff 2008; Simons 2010; Wang et al. 2011; Close and Rayfield 2012; Voeten et al. 2018; Serrano et al. 2020; Feneck et al. 2021; Karoullas and Nudds 2021; Lowi-Merri et al. 2021; Mayr et al. 2021; Watanabe et al. 2021; Mayr 2021a; Holmes et al. 2022; Kotnour et al. 2022; Smith et al. 2022; Akeda and Fujiwara 2023; Eliason et al. 2023; Gündemir et al. 2023; Haidr 2023; Lowi-Merri et al. 2023; Shatkovska and Ghazali 2023; De Mendoza et al. 2024; Orkney and Hedrick 2024; Segesdi et al. 2024; Watanabe 2025; Weeks et al. 2025). This trend may be especially evident in the results of our unconstrained analyses, which recovered, for example, one group containing the burst-flying *Eudromia*, Galliformes, *Monias*, and *Turnix*, and another uniting the diving *Podilymbus, Alca, Gavia*, and *Spheniscus*. Such observations furthermore align with previously reported evidence for strong evolutionary constraints on the morphological disparity (Wang and Zhou 2023), complexity (Brinkworth et al. 2023), and modularity (Orkney and Hedrick 2024; Nebreda et al. 2025) of the forelimb skeleton in birds and their close extinct relatives (though see Navalón et al. 2022 and Nebreda et al. 2025 regarding complex intra-skeletal and intra-clade variability in the establishment of these evolutionary patterns). Considerable variation with little obvious correlation with either phylogeny or function has notably been previously reported in the sternal morphology of passeriforms (Heimerdinger and Ames 1967; Webster 1992). Our results also broadly parallel those of Ericson and Qu (2025), who found evidence of pervasive homoplasy and few unambiguous synapomorphies characterizing deep avian divergences captured in the morphological dataset of Livezey and Zusi (2006), especially among postcranial osteological characters.

These findings echo previous recommendations for caution in assessing the phylogenetic placement of avian or avian-like fossil specimens known only from isolated pectoral and forelimb elements, especially those potentially belonging to stem-group lineages for which morphological variation is poorly understood (Longrich et al. 2011; Mohr et al. 2021; Wilson et al. 2025). Nonetheless, even if subject to homoplastic gains and losses, some of the characters identified in the present study seem to represent plausible synapomorphies of clades that have hitherto been difficult to characterize morphologically (such as Phaethontimorphae and Telluraves), and are thus potentially informative for understanding morphological evolution across Neornithes and identifying fossil representatives of these groups. Therefore, the optimization of thoroughly vetted anatomical characters onto molecular phylogenetic topologies may prove fruitful for diagnosing recently recognized avian clades, as has been previously suggested (Mayr 2011a; Mayr 2014c; Steell et al. 2023). Among the individual elements of the pectoral girdle and forelimb skeleton, the humerus and carpometacarpus may be relatively reliable for assessing phylogenetic relationships according to our heatmap visualizations (though see Steell et al. 2023 regarding widespread homoplasy in carpometacarpal characters within Passeriformes), potentially increasing confidence in the identification of fossil bird taxa based on these bones.

However, the absence of fossil crown bird taxa in the present dataset should be kept in mind as a notable limitation. Fossils provide the only direct evidence of transitional ancestral morphologies that have been lost or modified in extant organisms, and as a result have the potential to dramatically affect inferences about phylogenetic topology and character polarity (e.g., Gauthier et al. 1988; Donoghue et al. 1989; Mayr and Knopf 2007; Mayr 2007a, 2008d, 2014c; Hsiang et al. 2015; Puttick 2016; Chen et al. 2019; Field et al. 2020; Mongiardino Koch and Parry 2020; Mongiardino Koch et al. 2021; Asher and Smith 2022; Beck et al. 2022; Kuo et al. 2023; Phillips et al. 2023). This effect is particularly noticeable in the present study regarding the optimization of pectoral and forelimb synapomorphies for crown birds and neognaths. The presence of two burst-flying groups (Tinamidae and Galliformes) among the extant outgroups to Neoaves likely leads to the reconstruction of some adaptations related to burst flight as ancestral to crown birds, even though these features are lacking in potential stem-paleognaths and early stem-galliforms (see previous discussion on potential synapomorphies of Neornithes and Neognathae). Our heatmap visualizations further indicate that crown galliforms (especially the phasianid *Rollulus*) do not closely resemble crownward stem-birds in their overall pectoral girdle and forelimb osteology (Fig. 20). Instead, the two crownward stem-birds included in our dataset (*Gansus* and *Ichthyornis*) appear to exhibit greater general similarity to members of Anseriformes, Mirandornithes, and Charadriiformes. This may suggest that the pectoral girdle and forelimb anatomy of these groups is more reminiscent of plesiomorphic states in the avian crown group, recalling the recent conclusion that elements of the palate in extant anseriforms may approximate the crown bird ancestral condition (Benito et al. 2022b). Alternatively, morphological similarities among these taxa may derive from repeated adaptation to aquatic lifestyles, which have been inferred for both of the stem-bird taxa in our dataset (You et al. 2006; Li et al. 2011; Nudds et al. 2013; Benito et al. 2022a; Lowi-Merri et al. 2023). It is simultaneously possible that the last common ancestor of crown birds was littoral or otherwise associated with aquatic environments, as hypothesized by Feduccia (1995) and supported by the morphology and paleoenvironments of the oldest known crown bird fossils (Clarke et al. 2016; Agnolín et al. 2017; Field et al. 2020; Acosta Hospitaleche and Worthy 2021; Field et al. 2024; Torres et al. 2025), but that homoplastic evolution complicates this signal in reconstructions based on extant taxa alone. Regardless of the exact evolutionary drivers underpinning these resemblances, the bones comprising the pectoral girdle and forelimb apparatus in putative stem-anseriforms share noticeable similarities with those of putative stem-mirandornitheans, charadriiforms, and some Cretaceous avialans, as has been noted by previous authors (Feduccia 1976; Olson and Feduccia 1980b; Ericson 1997; Ericson 1999; Zelenkov 2021; Houde et al. 2023). Careful reconsideration of Mesozoic fossils that have been assigned to total-group Anseriformes and Charadriiformes based on elements from these anatomical regions (e.g., Olson and Feduccia 1980a; Hope 2002; Kurochkin et al. 2002; De Pietri et al. 2016; Novas et al. 2019; Acosta Hospitaleche et al. 2023) may therefore be warranted.

Whether the addition of characters from other anatomical regions and the incorporation of information from a representative sample of fossil avian taxa will increase congruence between the results of morphological and molecular phylogenetic analyses for birds remains to be seen, especially given that recent research has indicated that different anatomical regions may favor different phylogenetic topologies in macroevolutionary studies of some vertebrate taxa (Benson 2012; Mounce et al. 2016; Sansom and Wills 2017; Sansom et al. 2017; Li et al. 2020; Callender-Crowe and Sansom 2022; Ericson and Qu 2025). Although large-scale morphological phylogenetic datasets have been criticized on the grounds that they may contain relatively little phylogenetic signal (Mayr 2008a; Yu et al. 2021), increased dataset size is an inevitable outcome of expanding taxonomic and character scope (Laing et al. 2018). Even if incongruent with phylogenomic studies when analyzed on their own, large morphological datasets may yet prove useful for identifying synapomorphies of major clades, placing fossils into phylogenetic context, and studying rates of character evolution.

On top of increasing taxon and character sampling, various methodological techniques have been suggested to improve the accuracy of morphological phylogenetic investigation. These include Bayesian analyses (Wright and Hillis 2014; O'Reilly et al. 2016; Puttick et al. 2017; O'Reilly et al. 2018; Puttick et al. 2019; Smith 2019; Keating et al. 2020; Vernygora et al. 2020; Simões et al. 2023; Barbosa et al. 2024), especially the implementation of tip dating (Mongiardino Koch et al. 2021; Barido-Sottani et al. 2023; Mongiardino Koch et al. 2023; Lian et al. 2024); implied weights parsimony analyses (Goloboff et al. 2018; Smith 2019; Rio and Mannion 2021); the use of continuous characters (Parins-Fukuchi 2018; Rio and Mannion 2021; Tagliacollo et al. 2025); the selective removal of homoplasy-prone characters (Dávalos et al. 2014; Zou and Zhang 2016); the inclusion of hypothetical ancestral morphologies as distinct operational taxonomic units (Asher et al. 2019); and correcting for ecological signal in morphological datasets (Phillips et al. 2023). However, the value of some of these techniques remains contested, and probably no single method on its own will emerge as a panacea for increasing congruence between morphological and molecular phylogenies (Dávalos et al. 2012; Congreve and Lamsdell 2016; Sookias 2020; Brady and Springer 2021; Asher and Smith 2022; Keating et al. 2023; Phillips et al. 2023; Holvast et al. 2024). In the present study, Bayesian analyses tended to produce trees slightly more congruent with molecular topologies than equal weights parsimony analysis under unconstrained topologies. However, under partial topological constraints, our implied weights parsimony and Bayesian analyses did not consistently recover results substantially closer to molecular topologies compared to equal weights parsimony analyses. Irrespective of dataset size and method choice, clear character construction and repeatability of character scoring is essential to all morphological phylogenetic analyses (Simões et al. 2017; Sookias 2020; Simões et al. 2023; Khakurel et al. 2024).

## Conclusion

The present study represents part of an initiative to produce a comprehensive morphological phylogenetic dataset for crown-group birds. By analyzing a novel dataset that extensively samples characters from the pectoral girdle and forelimb skeleton of a phylogenetically diverse set of extant birds, we found that this anatomical region does appear to exhibit substantial signal conflict with molecular sources of data, perhaps in part as a result of homoplasy driven by functional convergence. However, optimizing patterns of character acquisition onto molecular topologies recovered plausible synapomorphies for several clades for which morphological support had been largely unrecognized. Future work sourcing characters from other anatomical regions, incorporating key fossil taxa, and experimenting with additional phylogenetic methods may further clarify controversial aspects of avian systematics and patterns of morphological evolution during the early evolutionary history of crown birds.

## Supplementary Material

obaf029_Supplemental_Files

## Data Availability

The data underlying this article are available in the article and in its online supplementary material. Volumetric scans of specimens used in this research are available on Morphosource (project IDs 00000C420 and 000405009).

## References

[bib1] Acosta Hospitaleche C, O'Gorman JP, Panzeri KM. 2023. A new Cretaceous bird from the Maastrichtian La Colonia Formation (Patagonia, Argentina). Cretaceous Res 150: 105595. 10.1016/j.cretres.2023.105595

[bib2] Acosta Hospitaleche C, Worthy TH. 2021. New data on the *Vegavis iaai* holotype from the Maastrichtian of Antarctica. Cretaceous Res 124: 104818. 10.1016/j.cretres.2021.104818

[bib3] Agnolín FL, Brissón Egli F, Chatterjee S, García Marsà JA, Novas FE. 2017. Vegaviidae, a new clade of southern diving birds that survived the K/T boundary. Sci Nat 104: 87. 10.1007/s00114-017-1508-y 28988276

[bib4] Akeda T, Fujiwara S. 2023. Coracoid strength as an indicator of wing-beat propulsion in birds. J Anat 242: 436–46. 10.1111/joa.13788 36380603 PMC9919476

[bib5] Ando T, Fukata K. 2018. A well-preserved partial scapula from Japan and the reconstruction of the triosseal canal of plotopterids. PeerJ 6: e5391. 10.7717/peerj.5391 30155348 PMC6112113

[bib6] Asher RJ, Smith MR. 2022. Phylogenetic signal and bias in paleontology. Syst Biol 71: 986–1008. 10.1093/sysbio/syab072 34469583 PMC9248965

[bib7] Asher RJ, Smith MR, Rankin A, Emry RJ. 2019. Congruence, fossils and the evolutionary tree of rodents and lagomorphs. R Soc Open Sci 6: 190387. 10.1098/rsos.190387 31417738 PMC6689570

[bib8] Barbosa FF, Mermudes JRM, Russo CAM. 2024. Performance of tree-building methods using a morphological dataset and a well-supported Hexapoda phylogeny. PeerJ 12: e16706. 10.7717/peerj.16706 38213769 PMC10782957

[bib9] Barido-Sottani J, Pohle A, De Baets K, Murdock D, Warnock RCM. 2023. Putting the F into FBD analysis: tree constraints or morphological data? Palaeontology 66: e12679. 10.1111/pala.12679

[bib10] Baumel JJ, Witmer LM. 1993. Osteologia. Pp. 45–132, in Baumel J.J., King A.S., Breazile J.E., Evans H.E., Berge J.C. (eds.), in Handbook of Avian Anatomy: Nomina Anatomica Avium. 2nd Edition. Publications of the Nuttall Ornithological Club 23. Cambridge, MA: Nuttall Ornithological Club.

[bib11] Beck RMD, Voss RS, Jansa SA. 2022. Craniodental morphology and phylogeny of marsupials. Bull Am Museum Natl Hist 457: 1–352. 10.1206/0003-0090.457.1.1

[bib12] Beddard FE . 1898. The Structure and Classification of Birds. London: Longmans, Green, and Co. 548 pp.

[bib13] Benito J, Chen A, Wilson LE, Bhullar B-AS, Burnham D, Field DJ. 2022a. Forty new specimens of *Ichthyornis* provide unprecedented insight into the postcranial morphology of crownward stem group birds. PeerJ 10: e13919. 10.7717/peerj.13919 36545383 PMC9762251

[bib14] Benito J, Kuo P-C, Widrig KE, Jagt JWM, Field DJ. 2022b. Cretaceous ornithurine supports a neognathous crown bird ancestor. Nature 612: 100–5. 10.1038/s41586-022-05445-y 36450906

[bib15] Benson RBJ . 2012. Interrelationships of basal synapsids: cranial and postcranial morphological partitions suggest different topologies. J Syst Paleontol 10: 601–24. 10.1080/14772019.2011.631042

[bib16] Benson RBJ, Choiniere JN. 2013. Rates of dinosaur limb evolution provide evidence for exceptional radiation in Mesozoic birds. Proc R Soc B 280: 20131780. 10.1098/rspb.2013.1780 PMC3757991 23945695

[bib17] Bertelli S, Chiappe LM, Mayr G. 2014. Phylogenetic interrelationships of living and extinct Tinamidae, volant palaeognathous birds from the New World. Zool J Linn Soc 172: 145–84. 10.1111/zoj.12156

[bib18] Bertelli S, Lindow BEK, Dyke GJ, Mayr G. 2013. Another charadriiform-like bird from the lower Eocene of Denmark. Paleontol J 47: 1282–301. 10.1134/S0031030113110026

[bib19] Bever GS, Gauthier JA, Wagner GP. 2011. Finding the frame shift: digit loss, developmental variability, and the origin of the avian hand. Evol Dev 13: 269–79. 10.1111/j.1525-142X.2011.00478.x 21535465

[bib20] Bjarnason A, Benson R. 2021. A 3D geometric morphometric dataset quantifying skeletal variation in birds. M3 7: e125. 10.18563/journal.m3.125

[bib21] Blokland JC, Reid CM, Worthy TH, Tennyson AJD, Clarke JA, Scofield RP. 2019. Chatham Island Paleocene fossils provide insight into the palaeobiology, evolution, and diversity of early penguins (Aves, Sphenisciformes). Palaeontol Electron 22: 78. 10.26879/1009

[bib22] Bochenski Z, Bochenski ZM. 2008. An old world hummingbird from the oligocene: a new fossil from Polish Carpathians. J Ornithol 149: 211–6. 10.1007/s10336-007-0261-y

[bib23] Botelho JF, Ossa-Fuentes L, Soto-Acuña S, Smith-Paredes D, Nuñez-León D, Salinas-Saavedra M, Ruiz-Flores M, Vargas AO. 2014. New developmental evidence clarifies the evolution of wrist bones in the dinosaur–bird transition. PLoS Biol 12: e1001957. 10.1371/journal.pbio.1001957 25268520 PMC4181957

[bib24] Bourdon E, Kristoffersen AV, Bonde N. 2016. A roller-like bird (Coracii) from the Early Eocene of Denmark. Sci Rep 6: 34050. 10.1038/srep34050 27670387 PMC5037458

[bib25] Bourdon E, Lindow B. 2015. A redescription of *Lithornis vulturinus* (Aves, Palaeognathae) from the Early Eocene Fur Formation of Denmark. Zootaxa 4032: 493–514. 10.11646/zootaxa.4032.5.2 26624382

[bib26] Bourdon E, Mourer-Chauviré C, Amaghzaz M, Bouya B. 2008. New specimens of *Lithoptila abdounensis* (Aves, Prophaethontidae) from the lower Paleogene of Morocco. J Vertebr Paleontol 28: 751–61. 10.1671/0272-4634(2008)28[751:NSOLAA]2.0.CO;2

[bib27] Brady PL, Springer MS. 2021. The effects of fossil taxa, hypothetical predicted ancestors, and a molecular scaffold on pseudoextinction analyses of extant placental orders. PLoS One 16: e0257338. 10.1371/journal.pone.0257338 34534236 PMC8448315

[bib28] Braun EL, Cracraft J, Houde P. 2019. Resolving the avian tree of life from top to bottom: the promise and potential boundaries of the phylogenomic era. Pp. 151–210, in Kraus R.H.S. (ed.), Avian Genomics in Ecology and Evolution. Cham: Springer.

[bib29] Braun EL, Kimball RT. 2021. Data types and the phylogeny of Neoaves. Birds 2: 1–22. 10.3390/birds2010001

[bib30] Bravo GA, Schmitt CJ, Edwards SV. 2021. What have we learned from the first 500 avian genomes? Annu Rev Ecol Evol Syst 52: 611–39. 10.1146/annurev-ecolsys-012121-085928

[bib31] Brinkworth A, Green E, Li Y, Oyston J, Ruta M, Wills MA. 2023. Bird clades with less complex appendicular skeletons tend to have higher species richness. Nat Commun 14: 5817. 10.1038/s41467-023-41415-2 37726273 PMC10509246

[bib32] Brocklehurst N, Field DJ. 2024. Tip dating and Bayes factors provide insight into the divergences of crown bird clades across the end-Cretaceous mass extinction. Proc R Soc B 291: 20232618. 10.1098/rspb.2023.2618 PMC10865003 38351798

[bib33] Brocklehurst N, Upchurch P, Mannion PD, O'Connor J. 2012. The completeness of the fossil record of Mesozoic birds: implications for early avian evolution. PLoS One 7: e39056. 10.1371/journal.pone.0039056 22761723 PMC3382576

[bib34] Burton MGP, Benson RBJ, Field DJ. 2023. Direct quantification of skeletal pneumaticity illuminates ecological drivers of a key avian trait. Proc R Soc B 290: 20230160. 10.1098/rspb.2023.0160 PMC10015330 36919426

[bib35] Callender-Crowe LM, Sansom RS. 2022. Osteological characters of birds and reptiles are more congruent with molecular phylogenies than soft characters are. Zool J Linnean Soc 194: 1–13. 10.1093/zoolinnean/zlaa136

[bib36] Cau A, Beyrand V, Barsbold R, Tsogtbaatar K, Godefroit P. 2021. Unusual pectoral apparatus in a predatory dinosaur resolves avian wishbone homology. Sci Rep 11: 14722. 10.1038/s41598-021-94285-3 34282248 PMC8289867

[bib37] Chen A, Field DJ. 2020. Phylogenetic definitions for *Caprimulgimorphae* (*Aves*) and major constituent clades under the International Code of Phylogenetic Nomenclature. Vertebrate Zool 70: 571–85. 10.26049/VZ70-4-2020-03

[bib38] Chen A, White ND, Benson RBJ, Braun MJ, Field DJ. 2019. Total-evidence framework reveals complex morphological evolution in nightbirds (Strisores). Diversity 11: 143. 10.3390/d11090143

[bib39] Chen G, Xie Y, Zhang G. 2025. Phylogenomics and comparative genomic perspective on the avian radiation. Nat Rev Biodivers 1: 439–60. 10.1038/s44358-025-00062-9

[bib40] Cheneval J . 1984. Les oiseaux aquatiques (Gaviiformes à Anseriformes) du gisement Aquitanien de Saint-Gerand-le-Puy (Allier, France): révision systématique. Palaeovertebrata 14: 33–115.

[bib41] Clarke JA . 2004. Morphology, phylogenetic taxonomy, and systematics of *Ichthyornis* and *Apatornis* (Avialae, Ornithurae). Bull Am Museum Natl Hist 286: 1–179. 10.1206/0003-0090(2004)286<0001:MPTASO>2.0.CO;2

[bib42] Clarke JA, Chatterjee S, Li Z, Riede T, Agnolín F, Goller F, Isasi MP, Martinioni DR, Mussel FJ, Novas FE. 2016. Fossil evidence of the avian vocal organ from the Mesozoic. Nature 538: 502–5. 10.1038/nature19852 27732575

[bib43] Clarke JA, Ksepka DT, Smith NA, Norell MA. 2009. Combined phylogenetic analysis of a new North American fossil species confirms widespread Eocene distribution for stem rollers (Aves, Coracii). Zool J Linnean Soc 157: 586–611. 10.1111/j.1096-3642.2009.00550.x

[bib44] Clarke JA, Zhou Z, Zhang F. 2006. Insight into the evolution of avian flight from a new clade of Early Cretaceous ornithurines from China and the morphology of *Yixianornis grabaui*. J Anat 208: 287–308. 10.1111/j.1469-7580.2006.00534.x 16533313 PMC2100246

[bib45] Close RA, Rayfield EJ. 2012. Functional morphometric analysis of the furcula in Mesozoic birds. PLoS One 7: e36664. 10.1371/journal.pone.0036664 22666324 PMC3364262

[bib46] Congreve CR, Lamsdell JC. 2016. Implied weighting and its utility in palaeontological datasets: a study using modelled phylogenetic matrices. Palaeontology 59: 447–62. 10.1111/pala.12236

[bib47] Cracraft J . 1985. Monophyly and phylogenetic relationships of the Pelecaniformes: a numerical cladistic analysis. Auk 102: 834–53. 10.1093/auk/102.4.834

[bib48] Cracraft J . 1988. The major clades of birds. Pp. 339–61, in Benton M.J. (ed.), The Phylogeny and Classification of the Tetrapods Volume 1: Amphibians, Reptiles, Birds. Oxford: Oxford University Press.

[bib49] Cracraft J, Clarke J. 2001. The basal clades of modern birds. Pp. 143–56, in Gauthier J., Gall L.F. (eds.), New Perspectives on the Origin and Early Evolution of Birds. New Haven, CT: Yale Peabody Museum.

[bib50] Crane A, Benito J, Chen A, Musser G, Torres CR, Clarke JA, Lautenschlager S, Ksepka DT, Field DJ. 2025. Taphonomic damage obfuscates interpretation of the retroarticular region of the *Asteriornis* mandible. Geobios 90: 31–43. 10.1016/j.geobios.2024.03.003

[bib51] Dávalos LM, Cirranello AL, Geisler JH, Simmons NB. 2012. Understanding phylogenetic incongruence: lessons from phyllostomid bats. Biol Rev 87: 991–1024. 10.1111/j.1469-185X.2012.00240.x 22891620 PMC3573643

[bib52] Dávalos LM, Velazco PM, Warsi OM, Smits PD, Simmons NB. 2014. Integrating incomplete fossils by isolating conflicting signal in saturated and non-independent morphological characters. Syst Biol 63: 582–600. 10.1093/sysbio/syu022 24817532

[bib53] Dececchi TA, Larsson HCE. 2009. Patristic evolutionary rates suggest a punctuated pattern in forelimb evolution before and after the origin of birds. Paleobiology 35: 1–12. 10.1666/07079.1

[bib54] De Mendoza RS, Carril J, Degrange FJ, Tambussi CP. 2024. Specialized diving traits in the generalist morphology of *Fulica* (Aves, Rallidae). Sci Rep 14: 13966. 10.1038/s41598-024-64853-4 38886412 PMC11183161

[bib55] De Pietri VL, Berger J-P, Pirkenseer C, Scherler L, Mayr G. 2010. New skeleton from the early Oligocene of Germany indicates a stem-group position of diomedeoidid birds. Acta Palaeontol Polonica 55: 23–34. 10.4202/app.2009.0069

[bib56] De Pietri VL, Mayr G. 2014. The phylogenetic relationships of the Early Miocene stork *Grallavis edwardsi*, with comments on the interrelationships of living Ciconiidae (Aves). Zool Scripta 43: 576–85. 10.1111/zsc.12074

[bib57] De Pietri VL, Scofield RP, Zelenkov N, Boles WE, Worthy TH. 2016. The unexpected survival of an ancient lineage of anseriform birds into the Neogene of Australia: the youngest record of Presbyornithidae. R Soc Open Sci 3: 150635. 10.1098/rsos.150635 26998335 PMC4785986

[bib58] Donoghue MJ, Doyle JA, Gauthier J, Kluge AG, Rowe T. 1989. The importance of fossils in phylogeny reconstruction. Annu Rev Ecol Syst 20: 431–60. 10.1146/annurev.es.20.110189.002243

[bib59] Duhamel A, Balme C, Legal S, Riamon S, Louchart A. 2020. An early Oligocene stem Galbulae (jacamars and puffbirds) from southern France, and the position of the Paleogene family Sylphornithidae. Auk 137: ukaa023. 10.1093/auk/ukaa023

[bib60] Eliason CM, Proffitt JV, Clarke JA. 2023. Early diversification of avian limb morphology and the role of modularity in the locomotor evolution of crown birds. Evolution 77: 342–54. 10.1093/evolut/qpac039 36611286

[bib61] Ericson PGP . 1997. Systematic relationships of the Palaeogene family Presbyornithidae (Aves: Anseriformes). Zool J Linnean Soc 121: 429–83. 10.1111/j.1096-3642.1997.tb01286.x

[bib62] Ericson PGP . 1999. New material of *Juncitarsus* (Phoenicopteriformes), with a guide for differentiating that genus from the Presbyornithidae (Anseriformes). Pp. 245–51, in Olson S.L. (ed.), Avian Paleontology at the Close of the 20th Century: Proceedings of the 4th International Meeting of the Society of Avian Paleontology and Evolution, Washington DC, 4-7 June 1996. Smithsonian Contributions to Paleobiology 89. Washington, D.C.: Smithsonian Institution Press.

[bib63] Ericson PGP . 2000. Systematic revision, skeletal anatomy, and paleoecology of the New World early Tertiary Presbyornithidae (Aves: Anseriformes). PaleoBios 20: 1–23.

[bib64] Ericson PGP, Qu Y. 2025. An evaluation of the usefulness of morphological characters to infer higher-level relationships in birds by mapping them to a molecular phylogeny. Biol J Linn Soc 145: blae070. 10.1093/biolinnean/blae070

[bib65] Evers SW, Benson RBJ. 2019. A new phylogenetic hypothesis of turtles with implications for the timing and number of evolutionary transitions to marine lifestyles in the group. Palaeontology 62: 93–134. 10.1111/pala.12384

[bib66] Farris JS . 1989. The retention index and the rescaled consistency index. Cladistics 5: 417–9. 10.1111/j.1096-0031.1989.tb00573.x 34933481

[bib67] Feduccia A . 1972. Variation in the posterior border of the sternum in some tree-trunk foraging birds. Wilson Bull 84: 315–28.

[bib68] Feduccia A . 1976. Osteological evidence for shorebird affinities of the flamingos. Auk 93: 587–601. 10.1093/auk/93.3.587

[bib69] Feduccia A . 1986. The scapulocoracoid of flightless birds: a primitive avian character similar to that of theropods. Ibis 128: 128–32. 10.1111/j.1474-919X.1986.tb02099.x

[bib70] Feduccia A . 1995. Explosive evolution in Tertiary birds and mammals. Science 267: 637–8. 10.1126/science.267.5198.637 17745839

[bib71] Feneck EM, Bickley SRB, Logan MPO. 2021. Embryonic development of the avian sternum and its morphological adaptations for optimizing locomotion. Diversity 13: 481. 10.3390/d13100481

[bib72] Field DJ, Benito J, Chen A, Jagt JWM, Ksepka DT. 2020. Late Cretaceous neornithine from Europe illuminates the origins of crown birds. Nature 579: 397–401. 10.1038/s41586-020-2096-0 32188952

[bib73] Field DJ, Benito J, Werning S, Chen A, Kuo P-C, Crane A, Widrig KE, Ksepka DT, Jagt JWM. 2024. Remarkable insights into modern bird origins from the Maastrichtian type area (north-east Belgium, south-east Netherlands). Neth J Geosci 103: e15. 10.1017/njg.2024.11

[bib74] Field DJ, Burton MG, Benito J, Plateau O, Navalón G. 2025. Whence the birds: 200 years of dinosaurs, avian antecedents. Biol Lett 21: 20240500. 10.1098/rsbl.2024.0500 39837495 PMC11750382

[bib75] Field DJ, Hsiang AY. 2018. A North American stem turaco, and the complex biogeographic history of modern birds. BMC Evol Biol 18: 102. 10.1186/s12862-018-1212-3 29936914 PMC6016133

[bib76] Fürbringer M . 1888. Untersuchungen zur Morphologie und Systematik der Vögel, zugleich ein Beitrag zur Anatomie der Stütz- und Bewegungsorgane. Amsterdam: Van Holkema. 1751 pp.

[bib77] Gatesy J, Springer MS. 2022. Phylogenomic coalescent analyses of avian retroelements infer zero-length branches at the base of Neoaves, emergent support for controversial clades, and ancient introgressive hybridization in Afroaves. Genes 13: 1167. 10.3390/genes13071167 35885951 PMC9324441

[bib78] Gauthier J, Kluge AG, Rowe T. 1988. Amniote phylogeny and the importance of fossils. Cladistics 4: 105–209. 10.1111/j.1096-0031.1988.tb00514.x 34949076

[bib79] Gill F, Donsker D, Rasmussen P. 2024. IOC World Bird List (v14.1). https://www.worldbirdnames.org/ (accessed on February 8, 2024)

[bib80] Giribet G . 2015. Morphology should not be forgotten in the era of genomics–a phylogenetic perspective. Zoologischer Anzeiger J Compar Zool 256: 96–103. 10.1016/j.jcz.2015.01.003

[bib81] Gishlick AD . 2001. The functional morphology of the manus and forelimb of *Deinonychus antirrhopus* and its importance for the origin of avian flight. Pp. 301–18, in Gauthier J., Gall L.F. (eds.), New Perspectives on the Origin and Early Evolution of Birds. New Haven, CT: Yale Peabody Museum.

[bib82] Goloboff PA, Catalano SA. 2016. TNT version 1.5, including a full implementation of phylogenetic morphometrics. Cladistics 32: 221–38. 10.1111/cla.12160 34727670

[bib83] Goloboff PA, Mattoni CI, Quinteros AS. 2006. Continuous characters analyzed as such. Cladistics 22: 589–601. 10.1111/j.1096-0031.2006.00122.x 34892898

[bib84] Goloboff PA, Torres A, Arias JS. 2018. Weighted parsimony outperforms other methods of phylogenetic inference under models appropriate for morphology. Cladistics 34: 407–37. 10.1111/cla.12205 34649370

[bib85] Gündemir MG, Szara T, Spataru C, Demircioglu I, Turek B, Petrovas G, Spataru MC. 2023. Shape differences of the *Carina sterni* in birds of various locomotion types. Anat Histol Embryol 52: 190–6. 10.1111/ahe.12870 36181376

[bib86] Habib MB, Ruff CB. 2008. The effects of locomotion on the structural characteristics of avian limb bones. Zool J Linnean Soc 153: 601–24. 10.1111/j.1096-3642.2008.00402.x

[bib87] Haidr NS . 2023. Ecomorphological variation of the penguin wing. J Morphol 284: e21588. 10.1002/jmor.21588. 37183492

[bib88] Harrison CJO . 1984. A revision of the fossil swifts (Vertebrata, Aves, Suborder Apodi), with descriptions of three new genera and two new species. Mededelingen van de Werkgroep voor Tertiaire en Kwartaire Geologie 21: 157–77.

[bib89] Hartman S, Mortimer M, Wahl WR, Lomax DR, Lippincott J, Lovelace DM. 2019. A new paravian dinosaur from the Late Jurassic of North America supports a late acquisition of avian flight. PeerJ 7: e7247. 10.7717/peerj.7247 31333906 PMC6626525

[bib90] Hawkins JA, Hughes CE, Scotland RW. 1997. Primary homology assessment, characters and character states. Cladistics 13: 275–83. 10.1111/j.1096-0031.1997.tb00320.x 34911232

[bib91] Heers AM . 2016. New perspectives on the ontogeny and evolution of avian locomotion. Integr Comp Biol 56: 428–41. 10.1093/icb/icw065 27371381

[bib92] Heers AM, Baier DB, Jackson BE, Dial KP. 2016. Flapping before flight: high resolution, three-dimensional skeletal kinematics of wings and legs during avian development. PLoS One 11: e0153446. 10.1371/journal.pone.0153446 27100994 PMC4872793

[bib93] Heers AM, Dial KP. 2012. From extant to extinct: locomotor ontogeny and the evolution of avian flight. Trends Ecol Evol 27: 296–305. 10.1016/j.tree.2011.12.003 22304966

[bib94] Heers AM, Varghese SL, Hatier LK, Cabrera JJ. 2021. Multiple functional solutions during flightless to flight-capable transitions. Front Ecol Evol 8: 573411. 10.3389/fevo.2020.573411

[bib95] Heimerdinger MA, Ames PL. 1967. Variation in the sternal notches of suboscine passeriform birds. Postilla 105: 1–44.

[bib96] Heingård M, Musser G, Hall SA, Clarke JA. 2021. New remains of *Scandiavis mikkelseni* inform avian phylogenetic relationships and brain evolution. Diversity 13: 651. 10.3390/d13120651

[bib97] Hesse A . 1988. Die Messelornithidae—eine neue Familie der Kranichartigen (Aves: Gruiformes: Rhynocheti) aus dem Tertiär Europas und Nordamerikas. J Ornithol 129: 83–95. 10.1007/BF01641534

[bib98] Hesse A . 1990. Die Beschreibung der Messelornithidae (Aves: Gruiformes: Rhynocheti) aus dem Alttertiär Europas und Nordamerikas. Courier Forschungsinstitut Senckenberg 128: 1–176.

[bib99] Hesse A . 1992. A new species of *Messelornis* (Aves: Gruiformes: Messelornithidae) from the middle Eocene Green River Formation. Pp. 171–8, in Campbell K.E. (ed.), Papers in Avian Paleontology Honoring Pierce Brodkorb. Natural History Museum of Los Angeles County, Science Series 36. Los Angeles, CA: Natural History Museum of Los Angeles County.

[bib100] Hieronymus TL, Waugh DA, Clarke JA. 2019. A new zygodactylid species indicates the persistence of stem passerines into the early Oligocene in North America. BMC Evol Biol 19: 3. 10.1186/s12862-018-1319-6 30611195 PMC6321701

[bib101] Höfling E, Alvarenga HMF. 2001. Osteology of the shoulder girdle in the Piciformes, Passeriformes and related groups of birds. Zoologischer Anzeiger J Compar Zool 240: 196–208. 10.1078/0044-5231-00016

[bib102] Holmes J, Sustaita D, Hertel F. 2022. Geometric morphometric analysis of the humerus in New and Old World vultures. J Morphol 283: 379–94. 10.1002/jmor.21449 35038183

[bib103] Holvast EJ, Celik MA, Phillips MJ, Wilson LAB. 2024. Do morphometric data improve phylogenetic reconstruction? A systematic review and assessment. BMC Ecol Evo 24: 127. 10.1186/s12862-024-02313-3 PMC11487705 39425066

[bib104] Hood SC, Torres CR, Norell MA, Clarke JA. 2019. New fossil birds from the earliest Eocene of Mongolia. Am Museum Novitates 2019: 1–22. 10.1206/3934.1

[bib105] Hope S . 2002. The Mesozoic radiation of Neornithes. Pp. 339–88, in Chiappe L.M., Witmer L.M. (eds.), Mesozoic Birds: Above the Heads of Dinosaurs. Berkeley and Los Angeles, CA: University of California Press.

[bib106] Houde P, Braun EL, Narula N, Minjares U, Mirarab S. 2019. Phylogenetic signal of indels and the neoavian radiation. Diversity 11: 108. 10.3390/d11070108

[bib107] Houde P, Braun EL, Zhou L. 2020. Deep-time demographic inference suggests ecological release as driver of neoavian adaptive radiation. Diversity 12: 164. 10.3390/d12040164

[bib108] Houde P, Dickson M, Camarena D. 2023. Basal Anseriformes from the early Paleogene of North America and Europe. Diversity 15: 233. 10.3390/d15020233

[bib109] Houde P, Olson SL. 1989. Small arboreal nonpasserine birds from the early Tertiary of western North America. Pp. 2030–6, in Ouellet H. (ed.), Acta XIX Congressus Internationalis Ornithologici. Ottawa: University of Ottawa Press.

[bib110] Houde P, Olson SL. 1992. A radiation of coly-like birds from the early Eocene of North America (Aves: Sandcoleiformes new order). Pp. 137–60, in Campbell K.E. (ed.), Papers in Avian Paleontology Honoring Pierce Brodkorb. Natural History Museum of Los Angeles County, Science Series 36. Los Angeles, CA: Natural History Museum of Los Angeles County.

[bib111] Houde PW . 1988. Paleognathous birds from the early Tertiary of the Northern Hemisphere. Publ Nuttall Ornithol Club 22: 1–148.

[bib112] Howard H . 1955. A new wading bird from the Eocene of Patagonia. Am Museum Novitates 1710: 1–25.

[bib113] Hsiang AY, Field DJ, Webster TH, Behlke ADB, Davis MB, Racicot RA, Gauthier JA. 2015. The origin of snakes: revealing the ecology, behavior, and evolutionary history of early snakes using genomics, phenomics, and the fossil record. BMC Evol Biol 15: 87. 10.1186/s12862-015-0358-5 25989795 PMC4438441

[bib114] Hui CA . 2002. Avian furcula morphology may indicate relationships of flight requirements among birds. J Morphol 251: 284–93. 10.1002/jmor.1089 11835365

[bib115] Hunt GR . 1996. Family Rhynochetidae (kagu). Pp. 218–25, in del Hoyo J., Elliott A., Sargatal J. (eds.), Handbook of the Birds of the World. Volume 3. Barcelona: Lynx Edicions.

[bib116] Hunt R, Reyes-Hernández JL, Shaw JJ, Solodovnikov A, Pedersen KS. 2025. Integrating deep learning derived morphological traits and molecular data for total-evidence phylogenetics: lessons from digitized collections. Syst Biol 74: 453–68. 10.1093/sysbio/syae072 39826140 PMC12162172

[bib117] Hurley C . 2019. gclus: clustering Graphics. R package version 1.3.2. https://CRAN.R-project.org/package=gclus

[bib118] Jadwiszczak P, Hospitaleche CA, Reguero M. 2013. Redescription of *Crossvallia unienwillia*: the only Paleocene Antarctic penguin. Ameghiniana 50: 545–53. 10.5710/AMGH.09.10.2013.1058

[bib119] Jarvis ED, Mirarab S, Aberer AJ, Li B, Houde P, Li C, Ho SYW, Faircloth BC, Nabholz B, Howard JT et al. 2014. Whole-genome analyses resolve early branches in the tree of life of modern birds. Science 346: 1320–31. 10.1126/science.1253451 25504713 PMC4405904

[bib120] Jenkins FA Jr. 1993. The evolution of the avian shoulder joint. Am J Sci 293: 253–67. 10.2475/ajs.293.A.253

[bib121] Karhu AA . 1999. A new genus and species of the family Jungornithidae (Apodiformes) from the late Eocene of the northern Caucasus, with comments on the ancestry of hummingbirds. Pp. 207–16, in Olson S.L. (ed.), Avian Paleontology at the Close of the 20th Century: Proceedings of the 4th International Meeting of the Society of Avian Paleontology and Evolution, Washington DC, 4-7 June 1996. Smithsonian Contributions to Paleobiology 89. Washington, D.C.: Smithsonian Institution Press.

[bib122] Karoullas C, Nudds RL. 2021. The link between avian brachial index, flight capability and the neornithine evolutionary radiation. J Morphol 282: 1698–707. 10.1002/jmor.21414 34570390

[bib123] Keating JN, Garwood RJ, Sansom RS. 2023. Phylogenetic congruence, conflict and consilience between molecular and morphological data. BMC Ecol Evo 23: 30. 10.1186/s12862-023-02131-z PMC10321016 37403037

[bib124] Keating JN, Sansom RS, Sutton MD, Knight CG, Garwood RJ. 2020. Morphological phylogenetics evaluated using novel evolutionary simulations. Syst Biol 69: 897–912. 10.1093/sysbio/syaa012 32073641 PMC7440746

[bib125] Khakurel B, Grigsby C, Tran TD, Zariwala J, Höhna S, Wright AM. 2024. The fundamental role of character coding in Bayesian morphological phylogenetics. Syst Biol 73: 861–71. 10.1093/sysbio/syae033 38963801 PMC13396948

[bib126] Kimball RT, Hosner PA, Braun EL. 2021. A phylogenomic supermatrix of Galliformes (landfowl) reveals biased branch lengths. Mol Phylogenet Evol 158: 107091. 10.1016/j.ympev.2021.107091 33545275

[bib127] Kimball RT, Oliveros CH, Wang N, White ND, Barker FK, Field DJ, Ksepka DT, Chesser RT, Moyle RG, Braun MJ et al. 2019. A phylogenomic (super) tree of birds (with lessons for other organisms). Diversity 11: 109. 10.3390/d11070109

[bib128] Kluge AG, Farris JS. 1969. Quantitative phyletics and the evolution of anurans. Syst Biol 18: 1–32. 10.1093/sysbio/18.1.1

[bib128a] Kotnour J, McPeek SJ, Wedig H, Dominguez J, Wright NA. 2022. Relative forelimb–hindlimb investment is associated with flight style, foraging strategy, and nestling period, but not nest type. Ornithology 139: ukab084. 10.1093/ornithology/ukab084

[bib129] Ksepka DT, Bertelli S, Balanoff AM, Grande L. 2025. A new species of Morsoravidae sheds light on beak and limb morphology in stem passerines. J Vertebr Paleontol 10.1080/02724634.2025.2514121

[bib130] Ksepka DT, Clarke JA. 2010. *Primobucco mcgrewi* (Aves: Coracii) from the Eocene Green River Formation: new anatomical data from the earliest constrained record of stem rollers. J Vertebr Paleontol 30: 215–25. 10.1080/02724630903412414

[bib131] Ksepka DT, Clarke JA, Grande L. 2011. Stem parrots (Aves, Halcyornithidae) from the Green River Formation and a combined phylogeny of Pan-Psittaciformes. J Paleontol 85: 835–52. 10.1666/10-108.1

[bib132] Ksepka DT, Clarke JA, Nesbitt SJ, Kulp FB, Grande L. 2013. Fossil evidence of wing shape in a stem relative of swifts and hummingbirds (Aves, Pan-Apodiformes). Proc R Soc B 280: 20130580. 10.1098/rspb.2013.0580 PMC3652446 23760643

[bib133] Ksepka DT, Early CM, Dzikiewicz K, Balanoff AM. 2023a. Osteology and neuroanatomy of a phasianid (Aves: Galliformes) from the Miocene of Nebraska. J Paleontol 97: 223–42. 10.1017/jpa.2022.80

[bib134] Ksepka DT, Field DJ, Heath TA, Pett W, Thomas DB, Giovanardi S, Tennyson AJD. 2023b. Largest-known fossil penguin provides insight into the early evolution of sphenisciform body size and flipper anatomy. J Paleontol 97: 434–53. 10.1017/jpa.2022.88

[bib135] Ksepka DT, Grande L, Mayr G. 2019. Oldest finch-beaked birds reveal parallel ecological radiations in the earliest evolution of passerines. Curr Biol 29: 657–663.e1. 10.1016/j.cub.2018.12.040 30744971

[bib136] Ksepka DT, Stidham TA, Williamson TE. 2017. Early Paleocene landbird supports rapid phylogenetic and morphological diversification of crown birds after the K–Pg mass extinction. Proc Natl Acad Sci USA 114: 8047–52. 10.1073/pnas.1700188114 28696285 PMC5544281

[bib137] Kuhl H, Frankl-Vilches C, Bakker A, Mayr G, Nikolaus G, Boerno ST, Klages S, Timmermann B, Gahr M. 2021. An unbiased molecular approach using 3’UTRs resolves the avian family-level tree of life. Mol Biol Evol 38: 108–27. 10.1093/molbev/msaa191 32781465 PMC7783168

[bib138] Kuo P-C, Benson RBJ, Field DJ. 2023. The influence of fossils in macroevolutionary analyses of 3D geometric morphometric data: a case study of galloanseran quadrates. J Morphol 284: e21594. 10.1002/jmor.21594 37183494

[bib139] Kurochkin EN, Dyke GJ, Karhu AA. 2002. A new presbyornithid bird (Aves, Anseriformes) from the Late Cretaceous of southern Mongolia. Am Museum Novitates 3386: 1–11. 10.1206/0003-0082(2002)386<0001:ANPBAA>2.0.CO;2

[bib140] Laing AM, Doyle S, Gold MEL, Nesbitt SJ, O'Leary MA, Turner AH, Wilberg EW, Poole KE. 2018. Giant taxon-character matrices: the future of morphological systematics. Cladistics 34: 333–5. 10.1111/cla.12197 34645074

[bib141] Lee MSY, Camens AB. 2009. Strong morphological support for the molecular evolutionary tree of placental mammals. J Evol Biol 22: 2243–57. 10.1111/j.1420-9101.2009.01843.x 19780874

[bib142] Lee MSY, Palci A. 2015. Morphological phylogenetics in the genomic age. Curr Biol 25: R922–9. 10.1016/j.cub.2015.07.009 26439355

[bib143] Lewis PO . 2001. A likelihood approach to estimating phylogeny from discrete morphological character data. Syst Biol 50: 913–25. 10.1080/106351501753462876 12116640

[bib144] Li Y, Ruta M, Wills MA. 2020. Craniodental and postcranial characters of non-avian Dinosauria often imply different trees. Syst Biol 69: 638–59. 10.1093/sysbio/syz077 31769837 PMC7302058

[bib145] Li Y, Zhang Y-G, Zhou Z-H, Li Z-H, Liu D, Wang X-L. 2011. New material of *Gansus* and a discussion on its habit. Vertebrata PalAsiatica 49: 435–45.

[bib146] Lian L, Peng H-W, Erst AS, Ortiz RC, Jabbour F, Chen Z-D, Wang W. 2024. Bayesian tip-dated phylogeny and biogeography of Cissampelideae (Menispermaceae): mitigating the effects of homoplastic morphological characters. Cladistics 40: 391–410. 10.1111/cla.12573 38469932

[bib147] Livezey BC, Zusi RL. 2006. Phylogeny of Neornithes. Bull Carnegie Museum Natl Hist 37: 1–544. 10.2992/0145-9058(2006)37[1:PON]2.0.CO;2

[bib148] Livezey BC, Zusi RL. 2007. Higher-order phylogeny of modern birds (Theropoda, Aves: Neornithes) based on comparative anatomy. II. Analysis and discussion. Zool J Linnean Soc 149: 1–95. 10.1111/j.1096-3642.2006.00293.x PMC2517308 18784798

[bib149] Lo Coco GE, Motta MJ, Agnolín FL, Novas FE. 2022. Wing osteology, myology, and function of *Rhea americana* (Aves, Rheidae). J Morphol 283: 1015–47. 10.1002/jmor.21486 35673834

[bib150] Longrich N . 2009. An ornithurine-dominated avifauna from the Belly River Group (Campanian, Upper Cretaceous) of Alberta, Canada. Cretaceous Res 30: 161–77. 10.1016/j.cretres.2008.06.007

[bib151] Longrich NR, Tokaryk T, Field DJ. 2011. Mass extinction of birds at the Cretaceous–Paleogene (K–Pg) boundary. Proc Natl Acad Sci USA 108: 15253–7. 10.1073/pnas.1110395108 21914849 PMC3174646

[bib152] Lowi-Merri TM, Benson RBJ, Claramunt S, Evans DC. 2021. The relationship between sternum variation and mode of locomotion in birds. BMC Biol 19: 165. 10.1186/s12915-021-01105-1 34412636 PMC8377870

[bib153] Lowi-Merri TM, Demuth OE, Benito J, Field DJ, Benson RBJ, Claramunt S, Evans DC. 2023. Reconstructing locomotor ecology of extinct avialans: a case study of *Ichthyornis* comparing sternum morphology and skeletal proportions. Proc R Soc B 290: 20222020. 10.1098/rspb.2022.2020 PMC9993061 36883281

[bib154] Luo H, Jiang X, Li B, Wu J, Shen J, Xu Z, Zhou X, Hou M, Huang Z, Ou X et al. 2023. A high-quality genome assembly highlights the evolutionary history of the great bustard (*Otis tarda*, Otidiformes). Commun Biol 6: 746. 10.1038/s42003-023-05137-x 37463976 PMC10354230

[bib155] Manegold A . 2006. Two additional synapomorphies of grebes Podicipedidae and flamingos Phoenicopteridae. Acta Ornithol 41: 79–82. 10.3161/068.041.0113

[bib156] Mather EK, Lee MSY, Camens AB, Worthy TH. 2022. An exceptional partial skeleton of a new basal raptor (Aves: Accipitridae) from the late Oligocene Namba Formation, South Australia. Hist Biol 34: 1175–207. 10.1080/08912963.2021.1966777

[bib157] Mayr G . 1998a. Ein Archaeotrogon (Aves: Archaeotrogonidae) aus dem Mittel-Eozän der Grube Messel (Hessen, Deutschland)? J Ornithol 139: 121–9. 10.1007/BF01651221

[bib158] Mayr G . 1998b. “Coraciiforme” und “piciforme” Kleinvögel aus dem Mittel-Eozän der Grube Messel (Hessen, Deutschland). Courier Forschungsinstitut Senckenberg 205: 1–101.

[bib159] Mayr G . 1998c. A new family of Eocene zygodactyl birds. Senckenbergiana lethaea 78: 199–209. 10.1007/BF03042769

[bib160] Mayr G . 1999a. Caprimulgiform birds from the middle Eocene of Messel (Hessen, Germany). J Vertebr Paleontol 19: 521–32. 10.1080/02724634.1999.10011162

[bib161] Mayr G . 1999b. A new trogon from the middle Oligocene of Céreste, France. Auk 116: 427–34. 10.2307/4089376

[bib162] Mayr G . 1999c. *Pumiliornis tessellatus* n. gen. n. sp., a new enigmatic bird from the middle Eocene of Grube Messel (Hessen, Germany). Courier Forschungsinstitut Senckenberg 216: 75–83.

[bib163] Mayr G . 2000a. A new basal galliform bird from the middle Eocene of Messel (Hessen, Germany). Senckenbergiana Lethaea 80: 45–57.

[bib164] Mayr G . 2000b. Charadriiform birds from the early Oligocene of Céreste (France) and the middle Eocene of Messel (Hessen, Germany). Geobios 33: 625–36.

[bib165] Mayr G . 2000c. Tiny hoopoe-like birds from the middle Eocene of Messel (Germany). Auk 117: 964–70. 10.1093/auk/117.4.964

[bib166] Mayr G . 2000d. A new raptor-like bird from the lower Eocene of North America and Europe. Senckenbergiana Lethaea 80: 59–65.

[bib167] Mayr G . 2000e. New or previously unrecorded avian taxa from the middle Eocene of Messel (Hessen, Germany). Fossil Rec 3: 207–19. 10.1002/mmng.20000030110

[bib168] Mayr G . 2001. A new specimen of the tiny middle Eocene bird *Gracilitarsus mirabilis* (new family: Gracilitarsidae). Condor 103: 78–84. 10.1093/condor/103.1.78

[bib169] Mayr G . 2002a. Osteological evidence for paraphyly of the avian order Caprimulgiformes (nightjars and allies). J Ornithol 143: 82–97 10.1007/BF02465461

[bib170] Mayr G . 2002b. A contribution to the osteology of the middle Eocene ibis *Rhynchaeites messelensis* (Aves: Threskiornithidae: Rhynchaeitinae nov. subfam.). Neues Jahrbuch für Geologie und Paläontologie Monatshefte, 2002: 501–12.

[bib171] Mayr G . 2002c. A new species of *Plesiocathartes* (Aves: ?Leptosomidae) from the middle Eocene of Messel, Germany. PaleoBios 22: 10–20.

[bib172] Mayr G . 2002d. On the osteology and phylogenetic affinities of the Pseudasturidae—lower Eocene stem-group representatives of parrots (Aves, Psittaciformes). Zool J Linnean Soc 136: 715–29. 10.1046/j.1096-3642.2002.00042.x

[bib173] Mayr G . 2003a. A new Eocene swift-like bird with a peculiar feathering. Ibis 145: 382–91. 10.1046/j.1474-919X.2003.00168.x

[bib174] Mayr G . 2003b. The phylogenetic affinities of the Shoebill (*Balaeniceps rex*). J Ornithol 144: 157–75. 10.1007/BF02465644

[bib175] Mayr G . 2004a. Morphological evidence for sister group relationship between flamingos (Aves: Phoenicopteridae) and grebes (Podicipedidae). Zool J Linnean Soc 140: 157–69. 10.1111/j.1096-3642.2003.00094.x

[bib176] Mayr G . 2004b. Phylogenetic relationships of the early Tertiary Messel rails (Aves, Messelornithidae). Senckenbergiana Lethaea 84: 317–22. 10.1007/BF03043474

[bib177] Mayr G . 2004c. The phylogenetic relationships of the early Tertiary Primoscenidae and Sylphornithidae and the sister taxon of crown group piciform birds. J Ornithol 145: 188–98. 10.1007/s10336-003-0018-1

[bib178] Mayr G . 2005a. The Palaeogene Old World potoo *Paraprefica* Mayr, 1999 (Aves, Nyctibiidae): its osteology and affinities to the New World Preficinae Olson, 1987. J Syst Paleontol 3: 359–70. 10.1017/S1477201905001653

[bib179] Mayr G . 2005b. Tertiary plotopterids (Aves, Plotopteridae) and a novel hypothesis on the phylogenetic relationships of penguins (Spheniscidae). J Zool Syst 43: 61–71. 10.1111/j.1439-0469.2004.00291.x

[bib180] Mayr G . 2005c. The postcranial osteology and phylogenetic position of the Middle Eocene *Messelastur gratulator* Peters, 1994—a morphological link between owls (Strigiformes) and falconiform birds? J Vertebr Paleontol 25: 635–45. 10.1671/0272-4634(2005)025[0635:TPOAPP]2.0.CO;2

[bib181] Mayr G . 2005d. Phylogenetic affinities and composition of the early Eocene Gracilitarsidae (Aves, ?Piciformes). Neues Jahrbuch für Geologie und Paläontologie Monatshefte 2005: 1–16. 10.1127/njgpm/2005/2005/1

[bib182] Mayr G . 2005e. A tiny barbet-like bird from the lower Oligocene of Germany: the smallest species and earliest substantial fossil record of the Pici (woodpeckers and allies). Auk 122: 1055–63. 10.1093/auk/122.4.1055

[bib183] Mayr G . 2006a. New specimens of the early Eocene stem group galliform *Paraortygoides* (Gallinuloididae), with comments on the evolution of a crop in the stem lineage of Galliformes. J Ornithol 147: 31–7. 10.1007/s10336-005-0006-8

[bib184] Mayr G . 2006b. New specimens of the Eocene Messelirrisoridae (Aves: Bucerotes), with comments on the preservation of uropygial gland waxes in fossil birds from Messel and the phylogenetic affinities of Bucerotes. Paläontol Z 80: 390–405. 10.1007/BF02990211

[bib185] Mayr G . 2006c. First fossil skull of a Palaeogene representative of the Pici (woodpeckers and allies) and its evolutionary implications. Ibis 148: 824–7. 10.1111/j.1474-919X.2006.00584.x

[bib186] Mayr G . 2007a. The renaissance of avian paleontology and its bearing on the higher-level phylogeny of birds. J Ornithol 148: 455–8. 10.1007/s10336-007-0159-8

[bib187] Mayr G . 2007b. New specimens of Eocene stem-group psittaciform birds may shed light on the affinities of the first named fossil bird, *Halcyornis toliapicus* Koenig, 1825. Neues Jahrbuch für Geologie und Paläontologie Abhandlungen 244: 207–13. 10.1127/0077-7749/2007/0244-0207

[bib188] Mayr G . 2008a. Avian higher-level phylogeny: well-supported clades and what we can learn from a phylogenetic analysis of 2954 morphological characters. J Zool Syst 46: 63–72. 10.1111/j.1439-0469.2007.00433.x

[bib189] Mayr G . 2008b. Phylogenetic affinities of the enigmatic avian taxon *Zygodactylus* based on new material from the early Oligocene of France. J Syst Paleontol 6: 333–44. 10.1017/S1477201907002398

[bib190] Mayr G . 2008c. The Madagascan “Cuckoo-roller” (Aves: Leptosomidae) is not a roller—notes on the phylogenetic affinities and evolutionary history of a “living fossil.” Acta Ornithol 43: 226–30. 10.3161/000164508X395360

[bib191] Mayr G . 2008d. The higher-level phylogeny of birds—when morphology, molecules, and fossils coincide. Oryctos 7: 67–73.

[bib192] Mayr G . 2009. A well-preserved second trogon skeleton (Aves, Trogonidae) from the middle Eocene of Messel, Germany. Palaeobio Palaeoenv 89: 1–6. 10.1007/s12549-009-0001-9

[bib193] Mayr G . 2010a. Reappraisal of *Eocypselus*—a stem group apodiform from the early Eocene of Northern Europe. Palaeobio Palaeoenv 90: 395–403. 10.1007/s12549-010-0043-z

[bib194] Mayr G . 2010b. Phylogenetic relationships of the paraphyletic ‘caprimulgiform’ birds (nightjars and allies). J Zool Systemat Evol Res 48: 126–37 10.1111/j.1439-0469.2009.00552.x

[bib195] Mayr G . 2010c. Parrot interrelationships—morphology and the new molecular phylogenies. Emu Austral Ornithol 110: 348–57. 10.1071/MU10035

[bib196] Mayr G . 2010d. A new avian species with tubercle-bearing cervical vertebrae from the Middle Eocene of Messel (Germany). Rec Aust Mus Pp. 21–8 in Boles W.E., Worthy T.H. (eds.), Proceedings of the VII International Meeting of the Society of Avian Paleontology and Evolution. Records of the Australian Museum 62. Sydney: The Australian Museum. 10.3853/j.0067-1975.62.2010.1537

[bib253] Mayr G . 2011a. Metaves, Mirandornithes, Strisores and other novelties—a critical review of the higher-level phylogeny of neornithine birds. J Zool Syst Evol Res 49: 58–76. 10.1111/j.1439-0469.2010.00586.x

[bib197] Mayr G . 2011b. The phylogeny of charadriiform birds (shorebirds and allies)—reassessing the conflict between morphology and molecules. Zool J Linnean Soc 161: 916–34. 10.1111/j.1096-3642.2010.00654.x

[bib198] Mayr G . 2011c. Cenozoic mystery birds—on the phylogenetic affinities of bony-toothed birds (Pelagornithidae). Zoologica Scripta 40: 448–67. 10.1111/j.1463-6409.2011.00484.x

[bib199] Mayr G . 2011d. Well-preserved new skeleton of the Middle Eocene *Messelastur* substantiates sister group relationship between Messelasturidae and Halcyornithidae (Aves, ?Pan-Psittaciformes). J Syst Paleontol 9: 159–71. 10.1080/14772019.2010.505252

[bib200] Mayr G . 2014a. Comparative morphology of the radial carpal bone of neornithine birds and the phylogenetic significance of character variation. Zoomorphology 133: 425–34. 10.1007/s00435-014-0236-5

[bib201] Mayr G . 2014b. The Eocene *Juncitarsus*—its phylogenetic position and significance for the evolution and higher-level affinities of flamingos and grebes. CR Palevol 13: 9–18. 10.1016/j.crpv.2013.07.005

[bib202] Mayr G . 2014c. The origins of crown group birds: molecules and fossils. Palaeontology 57: 231–42. 10.1111/pala.12103

[bib203] Mayr G . 2015a. New remains of the Eocene *Prophaethon* and the early evolution of tropicbirds (Phaethontiformes). Ibis 157: 54–67. 10.1111/ibi.12214

[bib204] Mayr G . 2015b. A reassessment of Eocene parrotlike fossils indicates a previously undetected radiation of zygodactyl stem group representatives of passerines (Passeriformes). Zool Scripta 44: 587–602. 10.1111/zsc.12128

[bib205] Mayr G . 2016a. The world's smallest owl, the earliest unambiguous charadriiform bird, and other avian remains from the early Eocene Nanjemoy Formation of Virginia (USA). PalZ 90: 747–63. 10.1007/s12542-016-0330-8

[bib206] Mayr G . 2016b. Osteology and phylogenetic affinities of the middle Eocene North American *Bathornis grallator*—one of the best represented, albeit least known Paleogene cariamiform birds (seriemas and allies). J Paleontol 90: 357–74. 10.1017/jpa.2016.45

[bib207] Mayr G . 2017a. Pectoral girdle morphology of Mesozoic birds and the evolution of the avian supracoracoideus muscle. J Ornithol 158: 859–67. 10.1007/s10336-017-1451-x

[bib208] Mayr G . 2017b. Avian Evolution: The Fossil Record of Birds and its Paleobiological Significance. Chichester: Wiley-Blackwell. 306 pp.

[bib209] Mayr G . 2018. New data on the anatomy and palaeobiology of sandcoleid mousebirds (Aves, Coliiformes) from the early Eocene of Messel. Palaeobio Palaeoenv 98: 639–51. 10.1007/s12549-018-0328-1

[bib210] Mayr G . 2019a. Hypotarsus morphology of the Ralloidea supports a clade comprising *Sarothrura* and *Mentocrex* to the exclusion of *Canirallus*. Acta Ornithol 54: 51–8. 10.3161/00016454AO2019.54.1.005

[bib211] Mayr G . 2019b. The otic region of the skull of neognathous birds: on the homology and comparative morphology of some neurovascular and muscular foramina and other external skeletal structures. Vertebrate Zool 70: 69–85. 10.26049/VZ70-1-2020-05

[bib212] Mayr G . 2020. A remarkably complete skeleton from the London Clay provides insights into the morphology and diversity of early Eocene zygodactyl near-passerine birds. J Syst Paleontol 18: 1891–906. 10.1080/14772019.2020.1862930

[bib213] Mayr G . 2021a. The coracoscapular joint of neornithine birds—extensive homoplasy in a widely neglected articular surface of the avian pectoral girdle and its possible functional correlates. Zoomorphology 140: 217–28. 10.1007/s00435-021-00528-2

[bib214] Mayr G . 2021b. An early Eocene fossil from the British London Clay elucidates the evolutionary history of the enigmatic Archaeotrogonidae (Aves, Strisores). Papers Palaeontol 7: 2049–64. 10.1002/spp2.1392

[bib215] Mayr G . 2021c. A partial skeleton of a new species of *Tynskya* Mayr, 2000 (Aves, Messelasturidae) from the London Clay highlights the osteological distinctness of a poorly known early Eocene “owl/parrot mosaic.” PalZ 95: 337–57. 10.1007/s12542-020-00541-8

[bib216] Mayr G . 2022a. Paleogene Fossil Birds. 2nd edition. Berlin: Springer. 251 pp.

[bib217] Mayr G . 2022b. A partial skeleton of *Septencoracias* from the early Eocene London Clay reveals derived features of bee-eaters (Meropidae) in a putative stem group roller (Aves, Coracii). Palaeobio Palaeoenv 102: 449–63. 10.1007/s12549-021-00504-0

[bib218] Mayr G, Alvarenga H, Mourer-Chauviré C. 2011. Out of Africa: fossils shed light on the origin of the hoatzin, an iconic Neotropic bird. Naturwissenschaften 98: 961. 10.1007/s00114-011-0849-1 21964974

[bib219] Mayr G, Bertelli S. 2011. A record of *Rhynchaeites* (Aves, Threskiornithidae) from the early Eocene Fur Formation of Denmark, and the affinities of the alleged parrot *Mopsitta*. Palaeobio Palaeoenv 91: 229–36. 10.1007/s12549-011-0050-8

[bib220] Mayr G, Bochenski ZM, Tomek T, Wertz K, Bienkowska-Wasiluk M, Manegold A. 2020b. Skeletons from the early Oligocene of Poland fill a significant temporal gap in the fossil record of upupiform birds (hoopoes and allies). Hist Biol 32: 1163–75. 10.1080/08912963.2019.1570507

[bib221] Mayr G, Carrió V, Kitchener AC. 2023a. On the “screamer-like” birds from the British London Clay: an archaic anseriform-galliform mosaic and a non-galloanserine “barb-necked” species of *Perplexicervix*. Palaeontol Electron 26: 33. 10.26879/1301

[bib222] Mayr G, Clarke J. 2003. The deep divergences of neornithine birds: a phylogenetic analysis of morphological characters. Cladistics 19: 527–53. 10.1111/j.1096-0031.2003.tb00387.x 34905857

[bib223] Mayr G, Daniels M. 1998. Eocene parrots from Messel (Hessen, Germany) and the London Clay of Walton-on-the-Naze (Essex, England). Senckenbergiana Lethaea 78: 157–77.

[bib263] Mayr G, De Pietri VL, Love L, Mannering AA, Bevitt JJ, Scofield RP. 2020a. First complete wing of a stem group sphenisciform from the Paleocene of New Zealand sheds light on the evolution of the penguin flipper. Diversity 12: 46. 10.3390/d12020046

[bib224] Mayr G, De Pietri V, Scofield RP. 2022. New bird remains from the early Eocene Nanjemoy Formation of Virginia (USA), including the first records of the Messelasturidae, Psittacopedidae, and Zygodactylidae from the Fisher/Sullivan site. Hist Biol 34: 322–34. 10.1080/08912963.2021.1910820

[bib225] Mayr G, De Pietri VL. 2014. Earliest and first Northern Hemispheric hoatzin fossils substantiate Old World origin of a “Neotropic endemic.” Naturwissenschaften 101: 143–8. 10.1007/s00114-014-1144-8 24441712

[bib226] Mayr G, De Pietri VL, Kitchener AC. 2023b. Narrow-beaked trogons from the early Eocene London Clay of Walton-on-the-Naze (Essex, UK). J Ornithol 164: 749–64. 10.1007/s10336-023-02071-x

[bib227] Mayr G, De Pietri VL, Love L, Mannering AA, Scofield RP. 2017a. A well-preserved new mid-Paleocene penguin (Aves, Sphenisciformes) from the Waipara Greensand in New Zealand. J Vertebr Paleontol 37: e1398169. 10.1080/02724634.2017.1398169

[bib228] Mayr G, Gingerich PD, Smith T. 2020c. Skeleton of a new owl from the early Eocene of North America (Aves, Strigiformes) with an accipitrid-like foot morphology. J Vertebr Paleontol 40: e1769116. 10.1080/02724634.2020.1769116

[bib229] Mayr G, Goedert JL, De Pietri VL, Scofield RP. 2021. Comparative osteology of the penguin-like mid-Cenozoic Plotopteridae and the earliest true fossil penguins, with comments on the origins of wing-propelled diving. J Zool Syst Evol Res 59: 264–76. 10.1111/jzs.12400

[bib230] Mayr G, Goedert JL, Richter A. 2025. Nearly complete late Eocene skull from the North Pacific elucidates the cranial morphology and affinities of the penguin-like Plotopteridae. Sci Nat 112: 27. 10.1007/s00114-025-01977-1 PMC11926016 40111588

[bib231] Mayr G, Goedert JL, Vogel O. 2015. Oligocene plotopterid skulls from western North America and their bearing on the phylogenetic affinities of these penguin-like seabirds. J Vertebr Paleontol 35: e943764. 10.1080/02724634.2014.943764

[bib232] Mayr G, Kitchener AC. 2021. New fossils from the London Clay show that the Eocene Masillaraptoridae are stem group representatives of falcons (Aves, Falconiformes). J Vertebr Paleontol 41: e2083515. 10.1080/02724634.2021.2083515

[bib233] Mayr G, Kitchener AC. 2022. Oldest fossil loon documents a pronounced ecomorphological shift in the evolution of gaviiform birds. Zool J Linnean Soc 196: 1431–50. 10.1093/zoolinnean/zlac045

[bib234] Mayr G, Kitchener AC. 2023a. The galliform birds from the lower Eocene London Clay of Walton-on-the-Naze (Essex, U.K.): new species suggest faunal connections to Asia. J Vertebr Paleontol 43: e2374305. 10.1080/02724634.2024.2374305

[bib235] Mayr G, Kitchener AC. 2023b. Early Eocene fossils elucidate the evolutionary history of the Charadriiformes (shorebirds and allies). J Paleontol 97: 941–55. 10.1017/jpa.2023.51

[bib236] Mayr G, Kitchener AC. 2023c. Multiple skeletons of *Rhynchaeites* from the London Clay reveal the osteology of early Eocene ibises (Aves, Threskiornithidae). PalZ 97: 425–42. 10.1007/s12542-022-00647-1

[bib237] Mayr G, Kitchener AC. 2023d. Early Eocene fossil illuminates the ancestral (diurnal) ecomorphology of owls and documents a mosaic evolution of the strigiform body plan. Ibis 165: 231–47. 10.1111/ibi.13125

[bib238] Mayr G, Kitchener AC. 2023e. New species from the early Eocene London Clay suggest an undetected early Eocene diversity of the Leptosomiformes, an avian clade that includes a living fossil from Madagascar. Palaeobio Palaeoenv 103: 585–608. 10.1007/s12549-022-00560-0

[bib239] Mayr G, Kitchener AC. 2023f. The Vastanavidae and Messelasturidae (Aves) from the early Eocene London Clay of Walton-on-the-Naze (Essex, UK). Neues Jahrbuch für Geologie und Paläontologie Abhandlungen 307: 113–39. 10.1127/njgpa/2023/1119

[bib240] Mayr G, Kitchener AC. 2023g. Psittacopedids and zygodactylids: the diverse and species-rich psittacopasserine birds from the early Eocene London Clay of Walton-on-the-Naze (Essex, UK). Hist Biol 35: 2372–95. 10.1080/08912963.2022.2141629

[bib241] Mayr G, Kitchener AC. 2023h. A new fossil from the London Clay documents the convergent origin of a “mousebird-like” tarsometatarsus in an early Eocene near-passerine bird. Acta Palaeontologica Polonica 68: 1–11. 10.4202/app.01049.2022

[bib242] Mayr G, Kitchener AC. 2024a. The non-apodiform Strisores (potoos, nightjars and allied birds) from the early Eocene London Clay of Walton-on-the-Naze. Palaeobio Palaeoenv 10.1007/s12549-024-00610-9

[bib243] Mayr G, Kitchener AC. 2024b. New fossils of *Eocypselus* and *Primapus* from the British London Clay reveal a high taxonomic and ecological diversity of early Eocene swift-like apodiform birds. Ibis 166: 1199–217. 10.1111/ibi.13323

[bib244] Mayr G, Kitchener AC. 2024c. A new species of the Prophaethontidae (Aves, Phaethontiformes) from the early Eocene London Clay. Hist Biol 1–8, 10.1080/08912963.2024.2418895

[bib245] Mayr G, Kitchener AC. 2024d. The Halcyornithidae from the early Eocene London Clay of Walton-on-the-Naze (Essex, UK): a species complex of Paleogene arboreal birds. Geobios 83: 45–60. 10.1016/j.geobios.2023.06.003

[bib246] Mayr G, Kitchener AC. 2024e. The Picocoraciades (hoopoes, rollers, woodpeckers, and allies) from the early Eocene London Clay of Walton-on-the-Naze. PalZ 98: 291–312. 10.1007/s12542-024-00687-9

[bib247] Mayr G, Kitchener AC. 2024f. A new mousebird (Aves, Coliiformes) from the early Eocene London Clay of Walton-on-the-Naze (Essex, United Kingdom) constitutes a morphological link between sandcoleids and coliids. Geodiversitas 46: 979–93. 10.5252/geodiversitas2024v46a20

[bib248] Mayr G, Kitchener AC. 2025a. The Lithornithiformes (Aves) from the early Eocene London Clay of Walton-on-the-Naze (Essex, UK). Papers Palaeontol 11: e1611. 10.1002/spp2.1611

[bib249] Mayr G, Kitchener AC. 2025b. Messelornithids and messelornithid-like birds from the early Eocene London Clay of Walton-on-the-Naze (Essex, UK). Geobios 90: 87–101. 10.1016/j.geobios.2023.12.011

[bib250] Mayr G, Kitchener AC. 2025c. Two new species of larger gruiform and charadriiform birds from the London Clay of Walton-on-the-Naze (Essex, UK). Palaeobio Palaeoenv 10.1007/s12549-025-00653-6

[bib251] Mayr G, Knopf CW. 2007. A stem lineage representative of buttonquails from the Lower Oligocene of Germany—fossil evidence for a charadriiform origin of the Turnicidae. Ibis 149: 774–82. 10.1111/j.1474-919X.2007.00712.x

[bib252] Mayr G, Manegold A, Johansson US. 2003. Monophyletic groups within ‘higher land birds’—comparison of morphological and molecular data. J Zool Syst 41: 233–48. 10.1046/j.1439-0469.2003.00230.x

[bib254] Mayr G, Mourer-Chauviré C. 2000. Rollers (Aves: Coraciiformes s.s.) from the middle Eocene of Messel (Germany) and the upper Eocene of the Quercy (France). J Vertebr Paleontol 20: 533–46. 10.1671/0272-4634(2000)020[0533:RACSSF]2.0.CO;2

[bib255] Mayr G, Mourer-Chauviré C, Weidig I. 2004. Osteology and systematic position of the Eocene Primobucconidae (Aves, Coraciiformes sensu stricto), with first records from Europe. J Syst Paleontol 2: 1–12. 10.1017/S1477201903001093

[bib256] Mayr G, Peters DS. 1998. The mousebirds (Aves: Coliiformes) from the middle Eocene of Grube Messel (Hessen, Germany). Senckenbergiana Lethaea 78: 179–97. 10.1007/BF03042768

[bib257] Mayr G, Peters DS, Rietschel S. 2002. Petrel-like birds with a peculiar foot morphology from the Oligocene of Germany and Belgium (Aves: Procellariiformes). J Vertebr Paleontol 22: 667–76. 10.1671/0272-4634(2002)022[0667:PLBWAP]2.0.CO;2

[bib258] Mayr G, Rana RS, Rose KD, Sahni A, Kumar K, Smith T. 2013. New specimens of the early Eocene bird *Vastanavis* and the interrelationships of stem group Psittaciformes. Paleontol J 47: 1308–14. 10.1134/S0031030113110105

[bib259] Mayr G, Rana RS, Sahni A, Smith T. 2007. Oldest fossil avian remains from the Indian subcontinental plate. Curr Sci 92: 1266–9.

[bib260] Mayr G, Scofield RP, De Pietri VL, Tennyson AJD. 2017b. A Paleocene penguin from New Zealand substantiates multiple origins of gigantism in fossil Sphenisciformes. Nat Commun 8: 1927. 10.1038/s41467-017-01959-6 29233963 PMC5727159

[bib261] Mayr G, Smith T. 2012. Phylogenetic affinities and taxonomy of the Oligocene Diomedeoididae, and the basal divergences amongst extant procellariiform birds. Zool J Linnean Soc 166: 854–75. 10.1111/j.1096-3642.2012.00858.x

[bib262] Mayr G, Weidig I. 2004. The early Eocene bird *Gallinuloides wyomingensis*—a stem group representative of Galliformes. Acta Palaeontol Polonica 49: 211–7.

[bib264] McInerney PL, Lee MSY, Clement AM, Worthy TH. 2019. The phylogenetic significance of the morphology of the syrinx, hyoid and larynx, of the southern cassowary, *Casuarius casuarius* (Aves, Palaeognathae). BMC Evol Biol 19: 233. 10.1186/s12862-019-1544-7 31881941 PMC6935130

[bib265] Middleton KM, Gatesy SM. 2000. Theropod forelimb design and evolution. Zool J Linnean Soc 128: 149–87. 10.1111/j.1096-3642.2000.tb00160.x

[bib266] Miller MA, Pfeiffer W, Schartz T. 2010. Creating the CIPRES Science Gateway for inference of large phylogenetic trees. Pp. 1–8 in Institute of Electrical and Electronics Engineers, Proceedings of the Gateway Computing Environments Workshop (GCE). Piscataway, NJ: IEEE Press.

[bib267] Mindell DP . 2020. Galloanserae. Pp. 1255–7, in de Queiroz K., Cantino P.D., Gauthier J.A. (eds.), Phylonyms: A Companion to the PhyloCode. Boca Raton, FL: CRC Press.

[bib268] Mirarab S, Rivas-González I, Feng S, Stiller J, Fang Q, Mai U, Hickey G, Chen G, Brajuka N, Fedrigo O et al. 2024. A region of suppressed recombination misleads neoavian phylogenomics. Proc Natl Acad Sci USA 121: e2319506121. 10.1073/pnas.2319506121 38557186 PMC11009670

[bib269] Mohr SR, Acorn JH, Funston G, Currie PJ. 2021. An ornithurine bird coracoid from the Late Cretaceous of Alberta, Canada. Can J Earth Sci 58: 134–40. 10.1139/cjes-2019-0202

[bib270] Mongiardino Koch N, Garwood RJ, Parry LA. 2021. Fossils improve phylogenetic analyses of morphological characters. Proc R Soc B 288: 20210044. 10.1098/rspb.2021.0044 PMC8246652 33947239

[bib271] Mongiardino Koch N, Garwood RJ, Parry LA. 2023. Inaccurate fossil placement does not compromise tip-dated divergence times. Palaeontology 66: e12680 10.1111/pala.12680

[bib272] Mongiardino Koch N, Parry LA. 2020. Death is on our side: paleontological data drastically modify phylogenetic hypotheses. Syst Biol 69: 1052–67. 10.1093/sysbio/syaa023 32208492

[bib273] Mounce RCP, Sansom R, Wills MA. 2016. Sampling diverse characters improves phylogenies: craniodental and postcranial characters of vertebrates often imply different trees. Evolution 70: 666–86. 10.1111/evo.12884 26899622

[bib274] Mourer-Chauviré C . 1980. The Archaeotrogonidae of the Eocene and Oligocene Phosphorites du Quercy (France). Pp. 17–31, in Campbell K.E. Jr. (ed.), Papers in Avian Paleontology Honoring Hildegarde Howard. Natural History Museum of Los Angeles County Contributions in Science 330. Los Angeles, CA: Natural History Museum of Los Angeles County.

[bib275] Mourer-Chauviré C . 1983. Les Gruiformes (Aves) des Phosphorites du Quercy (France). 1. Sous-ordre Cariamae (Cariamidae et Phorusrhacidae). Systématique et biostratigraphie. Palaeovertebrata 13: 83–143.

[bib276] Mourer-Chauviré C . 1987. Les Strigiformes (Aves) des Phosphorites du Quercy (France): systématique, Biostratigraphie et Paléobiogéographie. Documents des Laboratoires de Géologie, Lyon 99: 89–135.

[bib277] Mourer-Chauviré C . 1988a. Les Aegialornithidae (Aves: Apodiformes) des Phosphorites du Quercy. Comparaison avec la forme de Messel. Courier Forschungsinstitut Senckenberg 107: 369–81.

[bib278] Mourer-Chauviré C . 1988b. Le gisement du Bretou (Phosphorites du Quercy, Tarn-et-Garonne, France) et sa faune de vertébrés de l'Eocène supérieur. II. Oiseaux. Palaeontograph A 205: 29–50.

[bib279] Mourer-Chauviré C . 1991. The Horusornithidae nov. fam., Accipitriformes (Aves) with a hyperflexible intertarsal joint from the Eocene of Quercy. Geobios 24: 183–92. 10.1016/S0016-6995(66)80023-2

[bib280] Mourer-Chauviré C . 1992a. The Galliformes (Aves) from the Phosphorites du Quercy (France): systematics and biostratigraphy. Pp. 67–95, in Campbell K.E. (ed.), Papers in Avian Paleontology Honoring Pierce Brodkorb. Natural History Museum of Los Angeles County Science Series 36. Los Angeles, CA: Natural History Museum of Los Angeles County.

[bib281] Mourer-Chauviré C . 1992b. Une nouvelle famille de perroquets (Aves, Psittaciformes) dans l’Éocène supérieur des Phosphorites du Quercy. France. Geobios 25: 169–77.

[bib282] Mourer-Chauviré C . 1993. Les gangas (Aves, Columbiformes, Pteroclidae) du Paléogène et du Miocène inférieur de France. Palaeovertebrata 22: 73–98.

[bib283] Mourer-Chauviré C . 1995. The Messelornithidae (Aves: Gruiformes) from the Paleogene of France. Courier Forschungsinstitut Senckenberg 181: 95–105.

[bib284] Mourer-Chauviré C . 2000. A new species of *Ameripodius* (Aves: Galliformes: Quercymegapodiidae) from the lower Miocene of France. Palaeontology 43: 481–93.

[bib285] Musser G, Clarke JA. 2020. An exceptionally preserved specimen from the Green River Formation elucidates complex phenotypic evolution in Gruiformes and Charadriiformes. Front Ecol Evol 8: 559929. 10.3389/fevo.2020.559929

[bib286] Musser GM, Cracraft J. 2019. A new morphological dataset reveals a novel relationship for the adzebills of New Zealand (*Aptornis*) and provides a foundation for total evidence neoavian phylogenetics. Am Museum Novitates 2019: 1–70. 10.1206/3927.1

[bib287] Musser GM, Ksepka DT, Field DJ. 2019. New material of Paleocene-Eocene *Pellornis* (Aves: Gruiformes) clarifies the pattern and timing of the extant gruiform radiation. Diversity 11: 102. 10.3390/d11070102

[bib288] Napoli JG, Fabbri M, Ruebenstahl AA, O'Connor JK, Bhullar B-AS, Norell MA. 2025. Reorganization of the theropod wrist preceded the origin of avian flight. Nature 10.1038/s41586-025-09232-3 40634603

[bib289] Navalón G, Bjarnason A, Griffiths E, Benson RBJ. 2022. Environmental signal in the evolutionary diversification of bird skeletons. Nature 611: 306–11. 10.1038/s41586-022-05372-y 36289328

[bib290] Nebreda SM, Fernández MH, Marugán-Lobón J. 2025. Macroevolutionary integration underlies limb modularity in the origin of avian flight. Biol Lett 21: 20240685. 10.1098/rsbl.2024.0685 40328312 PMC12162096

[bib291] Nebreda SM, Navalón G, Menéndez I, Sigurdsen T, Chiappe LM, Marugán-Lobón J. 2020. Disparity and macroevolutionary transformation of the maniraptoran manus. Pp. 183–203, in Pittman M., Xu X. (eds.), Pennaraptoran Theropod Dinosaurs Past Progress and New Frontiers. Bulletin of the American Museum of Natural History 440. New York, NY: American Museum of Natural History.

[bib292] Nesbitt SJ, Clarke JA. 2016. The anatomy and taxonomy of the exquisitely preserved Green River Formation (early Eocene) lithornithids (Aves) and the relationships of Lithornithidae. Bull Am Museum Natl Hist 406: 1–91.

[bib293] Nesbitt SJ, Ksepka DT, Clarke JA. 2011. Podargiform affinities of the enigmatic *Fluvioviridavis platyrhamphus* and the early diversification of Strisores (“Caprimulgiformes” + Apodiformes). PLoS One 6: e26350. 10.1371/journal.pone.0026350 22140427 PMC3227577

[bib294] Nesbitt SJ, Turner AH, Spaulding M, Conrad JL, Norell MA. 2009. The theropod furcula. J Morphol 270: 856–79. 10.1002/jmor.10724 19206153

[bib295] Neumann JS, Desalle R, Narechania A, Schierwater B, Tessler M. 2021. Morphological characters can strongly influence early animal relationships inferred from phylogenomic data sets. Syst Biol 70: 360–75. 10.1093/sysbio/syaa038 32462193 PMC7875439

[bib296] Nixon KC . 2002. ASADO ver. 1.61. Ithaca, NY: Kevin C. Nixon.

[bib297] Novas FE, Agnolín FL, Brissón Egli F, Lo Coco GE. 2020. Pectoral girdle morphology in early-diverging paravians and living ratites: implications for the origin of flight. Pp. 345–53, in Pittman M., Xu X. (eds.), Pennaraptoran Theropod Dinosaurs Past Progress and New Frontiers. Bulletin of the American Museum of Natural History 440. New York, NY: American Museum of Natural History.

[bib298] Novas FE, Agnolín FL, Rozadilla S, Aranciaga-Rolando AM, Brissón-Egli F, Motta MJ, Cerroni M, Ezcurra MD, Martinelli AG, D'Angelo JS et al. 2019. Paleontological discoveries in the Chorrillo Formation (upper Campanian–lower Maastrichtian, Upper Cretaceous), Santa Cruz Province, Patagonia, Argentina. MACN 21: 217–93. 10.22179/REVMACN.21.655

[bib299] Novas FE, Motta MJ, Agnolín FL, Rozadilla S, Lo Coco GE, Brissón Egli F. 2021. Comments on the morphology of basal paravian shoulder girdle: new data based on unenlagiid theropods and paleognath birds. Front Earth Sci 9: 662167. 10.3389/feart.2021.662167

[bib300] Nudds RL, Atterholt J, Wang X, You H-L, Dyke GJ. 2013. Locomotory abilities and habitat of the Cretaceous bird *Gansus yumenensis* inferred from limb length proportions. J Evol Biol 26: 150–4. 10.1111/jeb.12036 23194019

[bib301] Nudds RL, Dyke GJ, Rayner JMV. 2007. Avian brachial index and wing kinematics: putting movement back into bones. J Zool 272: 218–26. 10.1111/j.1469-7998.2006.00261.x

[bib302] O'Connor JK, Zheng X-T, Sullivan C, Chuong C-M, Wang X-L, Li A, Wang Y, Zhang X-M, Zhou Z-H. 2015. Evolution and functional significance of derived sternal ossification patterns in ornithothoracine birds. J Evol Biol 28: 1550–67. 10.1111/jeb.12675 26079847 PMC5548695

[bib303] Olson SL . 1977. A lower Eocene frigatebird from the Green River Formation of Wyoming (Pelecaniformes: Fregatidae). Smithsonian Contrib Paleobiol 35: 1–33. 10.5479/si.00810266.35.1

[bib304] Olson SL . 1979. Multiple origins of the Ciconiiformes. Proc Colonial Waterbird Group 2: 165–70.

[bib305] Olson SL . 1980. A new genus of penguin-like pelecaniform bird from the Oligocene of Washington (Pelecaniformes: Plotopteridae). Pp. 5l–7, in Campbell, Jr. K.E. (ed.), Papers in Avian Paleontology Honoring Hildegarde Howard. Natural History Museum of Los Angeles County Contributions in Science 330. Los Angeles, CA: Natural History Museum of Los Angeles County.

[bib306] Olson SL . 1985. The fossil record of birds. Pp. 79–238, in Farner D.S., King J.R., Parkes K.C. (eds.), Avian Biology. Volume 8. New York, NY: Academic Press.

[bib307] Olson SL . 1992. A new family of primitive landbirds from the lower Eocene Green River Formation of Wyoming. Pp. 137–60, in Campbell K.E. (ed.), Papers in Avian Paleontology Honoring Pierce Brodkorb. Natural History Museum of Los Angeles County, Science Series 36. Los Angeles, CA: Natural History Museum of Los Angeles County.

[bib308] Olson SL . 1999. The anseriform relationships of *Anatalavis* Olson and Parris (Anseranatidae), with a new species from the lower Eocene London Clay. Pp. 231–43, in Olson S.L. (ed.), Avian Paleontology at the Close of the 20th Century: Proceedings of the 4th International Meeting of the Society of Avian Paleontology and Evolution, Washington D.C., 4-7 June 1996. Smithsonian Contributions to Paleobiology 89. Washington, DC: Smithsonian Institution Press.

[bib309] Olson SL, Feduccia A. 1979. Flight capability and the pectoral girdle of *Archaeopteryx*. Nature 278: 247–8. 10.1038/278247a0

[bib310] Olson SL, Feduccia A. 1980a. Relationships and evolution of flamingos (Aves, Phoenicopteridae). Smithsonian Contrib Zool 316: 1–73. 10.5479/si.00810282.316

[bib311] Olson SL, Feduccia A. 1980b. *Presbyornis* and the origin of the Anseriformes (Aves: Charadriomorphae). Smithsonian Contrib Zool 323: 1–24. 10.5479/si.00810282.323

[bib312] Olson SL, Matsuoka H. 2005. New specimens of the early Eocene frigatebird *Limnofregata* (Pelecaniformes: Fregatidae), with the description of a new species. Zootaxa 1046: 1–15. 10.11646/zootaxa.1046.1.1

[bib313] O'Reilly JE, Puttick MN, Parry L, Tanner AR, Tarver JE, Fleming J, Pisani D, Donoghue PCJ. 2016. Bayesian methods outperform parsimony but at the expense of precision in the estimation of phylogeny from discrete morphological data. Biol Lett 12: 20160081. 10.1098/rsbl.2016.0081 27095266 PMC4881353

[bib314] O'Reilly JE, Puttick MN, Pisani D, Donoghue PCJ. 2018. Probabilistic methods surpass parsimony when assessing clade support in phylogenetic analyses of discrete morphological data. Palaeontology 61: 105–18. 10.1111/pala.12330 29398726 PMC5784394

[bib315] Orkney A, Hedrick BP. 2024. Small body size is associated with increased evolutionary lability of wing skeleton proportions in birds. Nat Commun 15: 4208. 10.1038/s41467-024-48324-y 38806471 PMC11133451

[bib316] Ostrom JH . 1974. *Archaeopteryx* and the origin of flight. Q Rev Biol 49: 27–47. 10.1086/407902

[bib317] Ostrom JH . 1995. Wing biomechanics and the origin of bird flight. NJGPA 195: 253–66. 10.1127/njgpa/195/1995/253

[bib318] Pan H, Cole TL, Bi X, Fang M, Zhou C, Yang Z, Ksepka DT, Hart T, Bouzat JL, Argilla LS et al. 2019. High-coverage genomes to elucidate the evolution of penguins. GigaScience 8: giz117. 10.1093/gigascience/giz117 31531675 PMC6904868

[bib319] Paradis E, Schliep K. 2019. ape 5.0: an environment for modern phylogenetics and evolutionary analyses in R. Bioinformatics 35: 526–8. 10.1093/bioinformatics/bty633 30016406

[bib320] Parins-Fukuchi C . 2018. Use of continuous traits can improve morphological phylogenetics. Syst Biol 67: 328–39. 10.1093/sysbio/syx072 28945906

[bib321] Parker WK . 1869. On the osteology of the Kagu (*Rhinochetus jubatus*). Trans Zool Soc Lond 6: 501–22. 10.1111/j.1096-3642.1869.tb00585.x

[bib322] Peters DS . 1983. Die „Schnepfenralle” *Rhynchaeites messelensis* Wittich 1898 ist ein Ibis. J Ornithol 124: 1–27. 10.1007/BF01650658

[bib323] Peters DS . 1987. *Juncitarsus merkeli* n. sp. stützt die Ableitung der Flamingos von Regenpfeifervögeln (Aves: Charadriiformes: Phoenicopteridae). Courier Forschungsinstitut Senckenberg 97: 141–55.

[bib324] Peters DS . 1992. A new species of owl (Aves: Strigiformes) from the middle Eocene Messel oil shale. Pp. 161–9, in Campbell K.E. (ed.), Papers in Avian Paleontology Honoring Pierce Brodkorb. Natural History Museum of Los Angeles County, Science Series 36. Los Angeles, CA: Natural History Museum of Los Angeles County.

[bib325] Phillips MJ, Celik MA, Beck RMD. 2023. The evolutionary relationships of Diprotodontia and improving the accuracy of phylogenetic inference from morphological data. Alcheringa Austr J Palaeontol 47: 686–98. 10.1080/03115518.2023.2184492

[bib326] Pittman M, Kaye TG, Wang X, Zheng X, Dececchi TA, Hartman SA. 2022. Preserved soft anatomy confirms shoulder-powered upstroke of early theropod flyers, reveals enhanced early pygostylian upstroke, and explains early sternum loss. Proc Natl Acad Sci USA 119: e2205476119. doi:10.1073/pnas.2205476119 36375073 PMC9704744

[bib327] Prum RO, Berv JS, Dornburg A, Field DJ, Townsend JP, Lemmon EM, Lemmon AR. 2015. A comprehensive phylogeny of birds (Aves) using targeted next-generation DNA sequencing. Nature 526: 569–73. 10.1038/nature15697 26444237

[bib328] Puttick MN . 2016. Partially incorrect fossil data augment analyses of discrete trait evolution in living species. Biol Lett 12: 20160392. 10.1098/rsbl.2016.0392 27484647 PMC5014033

[bib329] Puttick MN, O'Reilly JE, Pisani D, Donoghue PCJ. 2019. Probabilistic methods outperform parsimony in the phylogenetic analysis of data simulated without a probabilistic model. Palaeontology 62: 1–17. 10.1111/pala.12388 PMC5784394 29398726

[bib330] Puttick MN, O'Reilly JE, Tanner AR, Fleming JF, Clark J, Holloway L, Lozano-Fernandez J, Parry LA, Tarver JE, Pisani D et al. 2017. Uncertain-tree: discriminating among competing approaches to the phylogenetic analysis of phenotype data. Proc R Soc B 284: 20162290. 10.1098/rspb.2016.2290 PMC5247500 28077778

[bib331] Pycraft WP . 1903. Contributions to the osteology of birds. Part VI. Cuculiformes. Proc Zool Soc Lond 73: 258–91.

[bib332] R Core Team . 2023. R: a language and environment for statistical computing. Vienna, Austria: R Foundation for Statistical Computing.

[bib333] Randle E, Sansom RS. 2017. Exploring phylogenetic relationships of Pteraspidiformes heterostracans (stem-gnathostomes) using continuous and discrete characters. J Syst Paleontol 15: 583–99. 10.1080/14772019.2016.1208293

[bib334] Reddy S, Kimball RT, Pandey A, Hosner PA, Braun MJ, Hackett SJ, Han K-L, Harshman J, Huddleston CJ, Kingston S et al. 2017. Why do phylogenomic data sets yield conflicting trees? Data type influences the avian tree of life more than taxon sampling. Syst Biol 66: 857–79. 10.1093/sysbio/syx041 28369655

[bib335] Revell LJ . 2024. phytools 2.0: an updated R ecosystem for phylogenetic comparative methods (and other things). PeerJ 12: e16505. 10.7717/peerj.16505 38192598 PMC10773453

[bib336] Rio JP, Mannion PD. 2021. Phylogenetic analysis of a new morphological dataset elucidates the evolutionary history of Crocodylia and resolves the long-standing gharial problem. PeerJ 9: e12094. 10.7717/peerj.12094 34567843 PMC8428266

[bib337] Ronquist F, Teslenko M, van der Mark P, Ayres DL, Darling A, Höhna S, Larget B, Liu L, Suchard MA, Huelsenbeck JP. 2012. MRBAYES 3.2: efficient Bayesian phylogenetic inference and model selection across a large model space. Syst Biol 61: 539–42. 10.1093/sysbio/sys029 22357727 PMC3329765

[bib338] Sangster G . 2020a. Mirandornithes. Pp. 1265–7, in de Queiroz K., Cantino P.D., Gauthier J.A. (eds.), Phylonyms: A Companion to the PhyloCode. Boca Raton, FL: CRC Press.

[bib339] Sangster G . 2020b. Charadriiformes. Pp. 1269–72, in de Queiroz K., Cantino P.D., Gauthier J.A. (eds.), Phylonyms: A Companion to the PhyloCode. Boca Raton, FL: CRC Press.

[bib340] Sangster G, Braun EL, Johansson US, Kimball RT, Mayr G, Suh A. 2022. Phylogenetic definitions for 25 higher-level clade names of birds. Avian Res 13: 100027. 10.1016/j.avrs.2022.100027

[bib341] Sangster G, Mayr G. 2021. *Feraequornithes*: a name for the clade formed by *Procellariiformes, Sphenisciformes, Ciconiiformes, Suliformes* and *Pelecaniformes* (*Aves*). Vertebrate Zool 71: 49–53 10.3897/vz.71.e61728

[bib342] Sansom RS, Wills MA. 2017. Differences between hard and soft phylogenetic data. Proc R Soc B 284: 20172150. 10.1098/rspb.2017.2150 PMC5745416 29237859

[bib343] Sansom RS, Wills MA, Williams T. 2017. Dental data perform relatively poorly in reconstructing mammal phylogenies: morphological partitions evaluated with molecular benchmarks. Syst Biol 66: 813–22. 10.1093/sysbio/syw116 28003534 PMC5790133

[bib344] Schliep KP . 2011. phangorn: phylogenetic analysis in R. Bioinformatics 27: 592–3. 10.1093/bioinformatics/btq706 21169378 PMC3035803

[bib345] Segesdi M, Brabant D, Cornette R, Houssaye A. 2024. How does the shape of the wing and hindlimb bones of aquatic birds relate to their locomotor abilities? Anat Rec 307: 3801–29. 10.1002/ar.25512 38803316

[bib346] Senter P . 2006. Scapular orientation in theropods and basal birds, and the origin of flapping flight. Acta Palaeontol Polonica 51: 305–13.

[bib347] Sereno PC . 2007. Logical basis for morphological characters in phylogenetics. Cladistics 23: 565–87. 10.1111/j.1096-0031.2007.00161.x 34905871

[bib348] Serrano FJ, Costa-Pérez M, Navalón G, Martín-Serra A. 2020. Morphological disparity of the humerus in modern birds. Diversity 12: 173. 10.3390/d12050173

[bib349] Shatkovska OV, Ghazali M. 2017. Relationship between developmental modes, flight styles, and wing morphology in birds. Eur Zool J 84: 390–401. 10.1080/24750263.2017.1346151

[bib350] Shatkovska OV, Ghazali M. 2023. Covariation in shapes between the sternum and pelvis in aquatic birds with different locomotor modes. Zoodiversity 57: 251–66. 10.15407/zoo2023.03.251

[bib351] Shimizu N, Anezaki T. 2024. Estimating the taxonomic group of a bird based on the shape of the sternum using specimens in Gunma Museum of Natural History. Bull Gunma Museum Natl Hist 28: 247–51.

[bib352] Simões TR, Caldwell MW, Palci A, Nydam RL. 2017. Giant taxon-character matrices: quality of character constructions remains critical regardless of size. Cladistics 33: 198–219. 10.1111/cla.12163 34710972

[bib353] Simões TR, Vernygora OV, de Medeiros BAS, Wright AM. 2023. Handling logical character dependency in phylogenetic inference: extensive performance testing of assumptions and solutions using simulated and empirical data. Syst Biol 72: 662–80. 10.1093/sysbio/syad006 36773019 PMC10276625

[bib354] Simons ELR . 2010. Forelimb skeletal morphology and flight mode evolution in pelecaniform birds. Zoology 113: 39–46. 10.1016/j.zool.2009.05.002 20071157

[bib354a] Slack KE, Jones CM, Ando T, Harrison GL(A), Fordyce RE, Arnason U, Penny D. 2006. Early penguin fossils, plus mitochondrial genomes, calibrate avian evolution. Mol Biol Evol 23: 1144–55. 10.1093/molbev/msj124 16533822

[bib355] Smith MR . 2019. Bayesian and parsimony approaches reconstruct informative trees from simulated morphological datasets. Biol Lett 15: 20180632. 10.1098/rsbl.2018.0632 30958126 PMC6405459

[bib354ab] Smith NA, DeBee AM, Clarke JA. 2018. Systematics and phylogeny of the Zygodactylidae (Aves, Neognathae) with description of a new species from the early Eocene of Wyoming, USA. PeerJ 6: e4950. 10.7717/peerj.4950 29967716 PMC6022727

[bib356] Smith NA, Koeller KL, Clarke JA, Ksepka DT, Mitchell JS, Nabavizadeh A, Ridgley RC, Witmer LM. 2022. Convergent evolution in dippers (Aves, Cinclidae): the only wing-propelled diving songbirds. Anat Rec 305: 1563–91. 10.1002/ar.24820 PMC9298897 34813153

[bib357] Smith ND . 2010. Phylogenetic analysis of Pelecaniformes (Aves) based on osteological data: implications for waterbird phylogeny and fossil calibration studies. PLoS One 5: e13354. 10.1371/journal.pone.0013354 20976229 PMC2954798

[bib358] Smith ND, Grande L, Clarke JA. 2013. A new species of Threskiornithidae-like bird (Aves, Ciconiiformes) from the Green River Formation (Eocene) of Wyoming. J Vertebr Paleontol 33: 363–81. 10.1080/02724634.2012.722898

[bib359] Smith ND, Ksepka DT. 2015. Five well-supported fossil calibrations within the “waterbird” assemblage (Tetrapoda, Aves). Palaeontol Electron 18: 1.7FC. 10.26879/483

[bib360] Sookias RB . 2020. Exploring the effects of character construction and choice, outgroups and analytical method on phylogenetic inference from discrete characters in extant crocodilians. Zool J Linnean Soc 189: 670–99. 10.1093/zoolinnean/zlz015

[bib361] Steell EM, Hsiang AY, Field DJ. 2025. Revealing patterns of homoplasy in discrete phylogenetic datasets with a cross-comparable index. Zool J Linnean Soc 204: zlaf024. 10.1093/zoolinnean/zlaf024

[bib362] Steell EM, Nguyen JMT, Benson RBJ, Field DJ. 2023. Comparative anatomy of the passerine carpometacarpus helps illuminate the early fossil record of crown Passeriformes. J Anat 242: 495–509. 10.1111/joa.13761 36070480 PMC9919509

[bib363] Stegmann B . 1963. Der Processus internus indicis im Skelett des Vogelflügels. J Ornithol 104: 413–23. 10.1007/BF01671057

[bib364] Stegmann B . 1964. Die funktionelle Bedeutung des Schlüsselbeines bei den Vögeln. J Ornithol 105: 450–63. 10.1007/BF01671621

[bib365] Stiller J, Feng S, Chowdhury A-A, Rivas-González I, Duchêne DA, Fang Q, Deng Y, Kozlov A, Stamatakis A, Claramunt S et al. 2024. Complexity of avian evolution revealed by family-level genomes. Nature 629: 851–60. 10.1038/s41586-024-07323-1 38560995 PMC11111414

[bib366] Storer RW . 1956. The fossil loon, *Colymboides minutus*. Condor 58: 413–26. 10.2307/1365096

[bib367] Suh A . 2016. The phylogenomic forest of bird trees contains a hard polytomy at the root of Neoaves. Zool Scripta 45: 50–62. 10.1111/zsc.12213

[bib368] Suh A, Smeds L, Ellegren H. 2015. The dynamics of incomplete lineage sorting across the ancient adaptive radiation of neoavian birds. PLoS Biol 13: e1002224. 10.1371/journal.pbio.1002224 26284513 PMC4540587

[bib369] Sullivan C, Hone DWE, Xu X, Zhang F. 2010. The asymmetry of the carpal joint and the evolution of wing folding in maniraptoran theropod dinosaurs. Proc R Soc B 277: 2027–33. 10.1098/rspb.2009.2281 PMC2880093 20200032

[bib370] Suzuki D, Chiba K, VanBuren CS, Ohashi T. 2014. The appendicular anatomy of the elegant crested tinamou (*Eudromia elegans*). Bull Kitakyushu Museum Natl Hist Hum Hist Ser A 12: 1–48.

[bib371] Tagliacollo VA, de Pinna M, Chuctaya J, Datovo A. 2025. Accuracy of phylogenetic reconstructions from continuous characters analysed under parsimony and its parametric correlates. Cladistics 41: 212–22. 10.1111/cla.12606 39915925

[bib372] Tambussi CP, Degrange FJ, De Mendoza RS, Sferco E, Santillana S. 2019. A stem anseriform from the early Palaeocene of Antarctica provides new key evidence in the early evolution of waterfowl. Zool J Linnean Soc 186: 673–700. 10.1093/zoolinnean/zly085

[bib373] Torres CR, Clarke JA, Groenke JR, Lamanna MC, MacPhee RDE, Musser GM, Roberts EM, O'Connor PM. 2025. Cretaceous Antarctic bird skull elucidates early avian ecological diversity. Nature 638: 146–51. 10.1038/s41586-024-08390-0 39910387

[bib374] Turner AH, Makovicky PJ, Norell M. 2012. A review of dromaeosaurid systematics and paravian phylogeny. Bull Am Museum Natl Hist 371: 1–206. 10.1206/748.1

[bib375] Vernygora OV, Simões TR, Campbell EO. 2020. Evaluating the performance of probabilistic algorithms for phylogenetic analysis of big morphological datasets: a simulation study. Syst Biol 69: 1088–105. 10.1093/sysbio/syaa020 32191335

[bib376] Vianna JA, Fernandes FAN, Frugone MJ, Figueiró HV, Pertierra LR, Noll D, Bi K, Wang-Claypool CY, Lowther A, Parker P et al. 2020. Genome-wide analyses reveal drivers of penguin diversification. Proc Natl Acad Sci USA 117: 22303–10. 10.1073/pnas.2006659117 32817535 PMC7486704

[bib377] Voeten DFAE, Cubo J, de Margerie E, Röper M, Beyrand V, Bureš S, Tafforeau P, Sanchez S. 2018. Wing bone geometry reveals active flight in *Archaeopteryx*. Nat Commun 9: 923. 10.1038/s41467-018-03296-8 29535376 PMC5849612

[bib378] Wang M, Mayr G, Zhang J, Zhou Z. 2012. Two new skeletons of the enigmatic, rail-like avian taxon *Songzia* Hou, 1990 (Songziidae) from the early Eocene of China. Alcheringa Austral J Palaeontol 36: 487–99. 10.1080/03115518.2012.673302

[bib379] Wang M, Zhou Z. 2023. Low morphological disparity and decelerated rate of limb size evolution close to the origin of birds. Nat Ecol Evol 7: 1257–66. 10.1038/s41559-023-02091-z 37277496

[bib380] Wang N, Braun EL, Liang B, Cracraft J, Smith SA. 2022. Categorical edge-based analyses of phylogenomic data reveal conflicting signals for difficult relationships in the avian tree. Mol Phylogenet Evol 174: 107550. 10.1016/j.ympev.2022.107550 35691570

[bib381] Wang S, Ma Y, Wu Q, Wang M, Hu D, Sullivan C, Xu X. 2022. Digital restoration of the pectoral girdles of two Early Cretaceous birds and implications for early-flight evolution. eLife 11: e76086. 10.7554/eLife.76086 35356889 PMC9023055

[bib382] Wang X, Clarke JA. 2014. Phylogeny and forelimb disparity in waterbirds. Evolution 68: 2847–60. 10.1111/evo.12486 24989899

[bib383] Wang X, McGowan AJ, Dyke GJ. 2011. Avian wing proportions and flight styles: first step towards predicting the flight modes of Mesozoic birds. PLoS One 6: e28672. 10.1371/journal.pone.0028672 22163324 PMC3233598

[bib384] Wang Y-M, O'Connor JK, Li D-Q, You H-L. 2016. New information on postcranial skeleton of the Early Cretaceous *Gansus yumenensis* (Aves: Ornithuromorpha). Hist Biol 28: 666–79. 10.1080/08912963.2015.1006217

[bib385] Watanabe J . 2025. Aspects of diversity, paleobiology, and morphology of wing-propelled diving birds. Geobios 90: 143–61. 10.1016/j.geobios.2024.11.005

[bib386] Watanabe J, Field DJ, Matsuoka H. 2021. Wing musculature reconstruction in extinct flightless auks (*Pinguinus* and *Mancalla*) reveals incomplete convergence with penguins (Spheniscidae) due to differing ancestral states. Integr Org Biol 3: obaa040. 10.1093/iob/obaa040 34258512 PMC8271220

[bib387] Webster JD . 1992. The manubrium–sternum bridge in songbirds (oscines). Proc Indiana Acad Sci 101: 299–308.

[bib388] Weeks BC, Harvey C, Tobias JA, Sheard C, Zhou Z, Fouhey DF. 2025. Longer wing bones in warmer climates suggest a role of thermoregulation in bird wing evolution. Global Ecol Biogeogr 34: e70033. 10.1111/geb.70033

[bib389] Weidig I . 2006. The first New World occurrence of the Eocene bird *Plesiocathartes* (Aves: ?Leptosomidae). Paläontol Z 80: 230–7. 10.1007/BF02988439

[bib390] Weidig I . 2010. New birds from the lower Eocene Green River Formation, North America. Rec Aust Mus Pp. 29–44, in Boles W.E., Worthy T.H. (eds.), Proceedings of the VII International Meeting of the Society of Avian Paleontology and Evolution. Records of the Australian Museum 62. Sydney: The Australian Museum. 10.3853/j.0067-1975.62.2010.1544

[bib391] White ND, Braun MJ. 2019. Extracting phylogenetic signal from phylogenomic data: higher-level relationships of the nightbirds (Strisores). Mol Phylogenet Evol 141: 106611. 10.1016/j.ympev.2019.106611 31520780

[bib392] Widrig KE, Bhullar B-AS, Field DJ. 2023. 3D atlas of tinamou (Neornithes: Tinamidae) pectoral morphology: implications for reconstructing the ancestral neornithine flight apparatus. J Anat 243: 729–57. 10.1111/joa.13919 37358291 PMC10557402

[bib393] Wilson LN, Ksepka DT, Wilson JP, Gardner JD, Erickson GM, Brinkman D, Brown CM, Eberle JJ, Organ CL, Druckenmiller PS. 2025. Arctic bird nesting traces back to the Cretaceous. Science 388: 974–8. 10.1126/science.adt5189 40440391

[bib394] Worthy TH, Degrange FJ, Handley WD, Lee MSY. 2017. The evolution of giant flightless birds and novel phylogenetic relationships for extinct fowl (Aves, Galloanseres). R Soc Open Sci 4: 170975. 10.1098/rsos.170975 29134094 PMC5666277

[bib395] Worthy TH, De Pietri VL, Scofield RP, Hand SJ. 2023. A new Eocene species of presbyornithid (Aves, Anseriformes) from Murgon, Australia. Alcheringa Austral J Palaeontol 47: 416–30. 10.1080/03115518.2023.2184491

[bib396] Worthy TH, Scofield RP. 2012. Twenty-first century advances in knowledge of the biology of moa (Aves: Dinornithiformes): a new morphological analysis and moa diagnoses revised. NZ J Zool 39: 87–153. 10.1080/03014223.2012.665060

[bib397] Wright AM, Hillis DM. 2014. Bayesian analysis using a simple likelihood model outperforms parsimony for estimation of phylogeny from discrete morphological data. PLoS One 9: e109210. 10.1371/journal.pone.0109210 25279853 PMC4184849

[bib398] Wu Q, O'Connor JK, Wang S, Zhou Z. 2024a. Transformation of the pectoral girdle in pennaraptorans: critical steps in the formation of the modern avian shoulder joint. PeerJ 12: e16960. 10.7717/peerj.16960 38436017 PMC10909347

[bib399] Wu Q, Zhou Z, Li Z. 2025. A secondary endochondral ossification centre in the furcula of extant birds and its significance for the evolution of the neornithine acrocoracoclavicular joint. Hist Biol 37: 1299–308. 10.1080/08912963.2024.2364337

[bib400] Wu S, Rheindt FE, Zhang J, Wang J, Zhang L, Quan C, Li Z, Wang M, Wu F, Qu Y et al. 2024b. Genomes, fossils, and the concurrent rise of modern birds and flowering plants in the Late Cretaceous. Proc Natl Acad Sci USA 121: e2319696121. 10.1073/pnas.2319696121 38346181 PMC10895254

[bib401] Xu X, Han F, Zhao Q. 2014. Homologies and homeotic transformation of the theropod ‘semilunate’ carpal. Sci Rep 4: 6042. 10.1038/srep06042 25116378 PMC4131224

[bib402] You H, Lamanna MC, Harris JD, Chiappe LM, O'Connor J, Ji S, Lü J, Yuan C, Li D, Zhang X et al. 2006. A nearly modern amphibious bird from the Early Cretaceous of northwestern China. Science 312: 1640–3. 10.1126/science.1126377 16778053

[bib403] Yu C, Jiangzuo Q, Tschopp E, Wang H, Norell M. 2021. Information in morphological characters. Ecol Evol 11: 11689–99. 10.1002/ece3.7874 34522333 PMC8427622

[bib404] Zelenkov NV . 2021. A revision of the Palaeocene–Eocene Mongolian Presbyornithidae (Aves: anseriformes). Paleontol J 55: 323–30. 10.1134/S0031030121030138

[bib405] Zheng X, O'Connor J, Wang X, Wang M, Zhang X, Zhou Z. 2014. On the absence of sternal elements in *Anchiornis* (Paraves) and *Sapeornis* (Aves) and the complex early evolution of the avian sternum. Proc Natl Acad Sci USA 111: 13900–5. 10.1073/pnas.1411070111 25201982 PMC4183337

[bib406] Zheng X, Wang X, O'Connor J, Zhou Z. 2012. Insight into the early evolution of the avian sternum from juvenile enantiornithines. Nat Commun 3: 1116. 10.1038/ncomms2104 23047674

[bib407] Zou Z, Zhang J. 2016. Morphological and molecular convergences in mammalian phylogenetics. Nat Commun 7: 12758. 10.1038/ncomms12758 27585543 PMC5025827

